# Competitive Inhibition as a Tool to Modulate and Predict Dynamic Hydrogel Mechanics

**DOI:** 10.1021/acscentsci.5c02130

**Published:** 2026-02-17

**Authors:** Alexander D. Claiborne, Sirilak Mekcham, Owen A. Lee, Megan R. Hill

**Affiliations:** Department of Chemistry, 3447 Colorado State University, 301 W Pitkin St., Fort Collins, Colorado 80521, United States

## Abstract

Dynamic hydrogels are powerful biomaterials whose performance in drug delivery, tissue engineering, and related applications depends on mechanical properties that remain difficult to predict. We introduce a simple, quantitative framework for tuning hydrogel mechanics through competitive inhibition, where small-molecule competitors reversibly disrupt cross-linking. Inspired by Michaelis–Menten kinetics, the model defines an apparent cross-link association constant, *K*_a,app_, that decreases as a function of competitor concentration and binding affinity. Incorporating *K*_a,app_ into traditional network theory enabled quantitative prediction of modulus. When using boronate ester networks and small-molecule competitors bearing diol motifs spanning 4 orders of magnitude in affinity, the predicted and measured moduli agreed within 10% relative error. A Langmuir-type decay function further captured stress-relaxation behavior by accounting for changes in effective cross-link density. Extending the approach to hydrazone-cross-linked gels confirmed its generality across distinct dynamic chemistries and exchange mechanisms. Finally, we demonstrate practical relevance by transforming a nonextrudable gel into a hand-injectable material through competitor addition. This framework establishes competitive inhibition as a universal and predictive strategy for designing adaptive soft materials.

## Graphical abstract

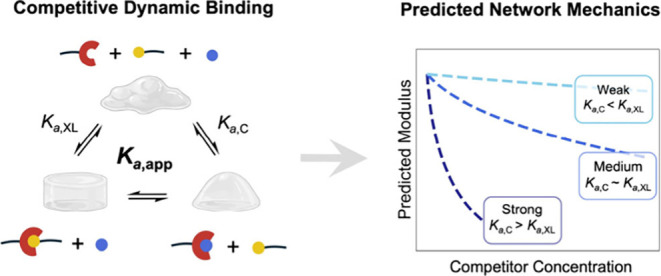

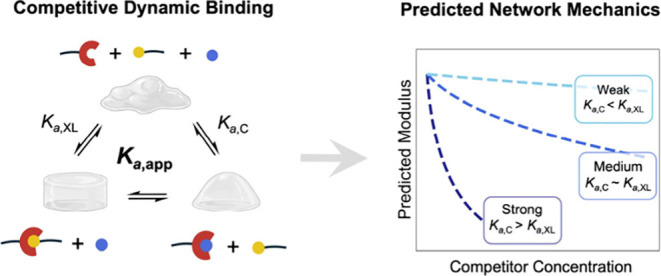

## Introduction

Synthetic hydrogels are water-swollen polymer networks valued for their softness, permeability, and biocompatibility. These attributes have enabled their widespread use in applications such as tissue scaffolds,^1,2^ drug delivery systems,^3–5^ and wound dressings.^6^ The macroscopic properties that underpin this broad utility arise from the chemistry and connectivity of cross-links within the hydrogel (Figure 1A). While traditional hydrogels use static covalent cross-links to provide mechanical stability, dynamic hydrogels incorporate reversible cross-links that can repeatedly break and reform, enabling self-healing, stress-induced flow, and responsiveness to chemical or mechanical cues (Figure 1B).^7–9^ In the context of biomaterials, these behaviors bring hydrogels even closer to the flexibility and functionality of living tissues.

**Figure 1:**
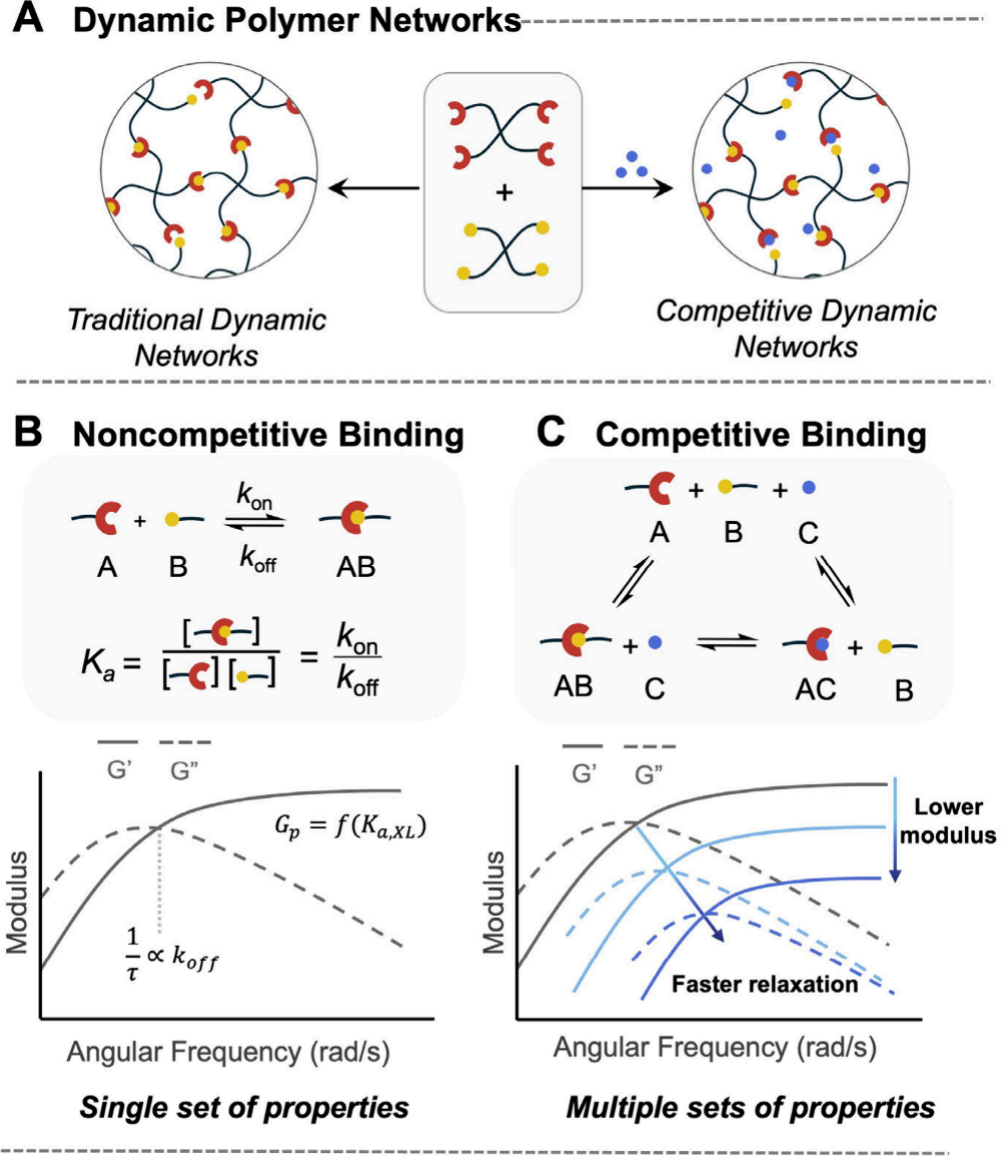
Conceptual framework for competitor-modulated hydrogels formed with ideal 4-arm macromer association. (A) In this work we alter the association strength and kinetics of traditional dynamic cross-links by adding competitors to the cross-link. (B) In the noncompetitive case, a single set of properties is defined by the cross-link association constant (*K*_a_) and dissociation rate (*k*_off_), determining modulus (*G*_p_), and relaxation time (τ). (C) In the competitive inhibition case, competitors with varying concentrations and affinities modulate cross-link thermodynamics through an apparent equilibrium constant (*K*_a,app_) enabling access to multiple sets of properties without changing the polymer composition.

Despite their promise, achieving precise and predictive control over the viscoelastic properties of dynamic hydrogels remains a central challenge. While classical network models can predict the elastic and viscoelastic response of individual hydrogel formulations,^10–15^ they do not provide a general strategy for continuously tuning these properties to reach a desired target. This limitation is especially important in biological settings, where small changes in stiffness and viscoelasticity can lead to divergent functional outcomes. For example, stem cells exhibit distinct gene expression profiles and lineage outcomes when cultured in environments of different stiffness or viscoelasticity.^16–18^ In drug delivery and tissue engineering contexts, control of cross-link density allows quantitative control over diffusivity^19,20^ or cellular mechanosensing.^21,22^ Other practical aspects of material design such as injectability for clinical application can be related to the mechanics of hydrogels.^23,24^ Thus, the ability to regulate and predict mechanical properties of hydrogels is of central importance in the field.

The mechanics of dynamic hydrogels can largely be described in terms of the network shear modulus (*G*_p_) and relaxation time (τ). *G*_p_ determines the stiffness of the material, while τ describes how quickly networks can rearrange under an applied stress. For dynamic hydrogels with cross-links in reversible equilibrium, it is well established that *G*_p_ is set by the equilibrium association constant (*K*_a_), while τ is governed by the dissociation rate constant (*k*_off_) of the cross-linking motif.^10,11^ Consequently, researchers have often sought to tune hydrogel properties by modifying cross-link chemistry^25,26^ –a strategy that typically requires synthesizing a new polymer network and results in dynamics that remain difficult to predict. Cross-links with a stimuli-response have been introduced to impart additional states, such as controlled phase changes or hydrogel stiffness.^27–30^ However, these approaches generally provide only one or two additional states. As a result, realizing continuum-type hydrogel mechanics often relies on slow, empirical tuning with limited generalizability and small design space.

A key parameter in governing the properties of dynamic networks is the number of *active* cross-links at a given time, as it directly determines *G*_p_ and τ.^31^ In reversible networks, this quantity is set by *K*_a_, such that stronger binding increases the fraction of engaged crosslinks. ().^10,11^ However, the ability to reversibly modulate how many cross-links simultaneously are engaged provides a powerful lever for tuning mechanics and flow without resynthesis. Such control would, for example, enable direct regulation over key functional outcomes, such as the rate of drug delivery, the diffusion of biomolecules, or stem-cell differentiation pathways governed by matrix stiffness and relaxation. One common approach to altering active cross-linking in dynamic networks is to adjust the concentration of polymer in solution, where decreasing polymer concentration reduces the number of active cross-links and lowers the stiffness of the material (). Other recent efforts have leveraged external stimuli to tune dynamic networks, expanding the accessible range of mechanical properties without polymer redesign. For instance, pH can strongly influence reversible reactions and dramatically alter network *G*_p_ and τ.^10^ While broadly tunable, this approach is limited in environments where pH is tightly regulated, such as the body.

A more general strategy is the addition of small molecules that compete with the cross-linking interaction (Figure 1A). Competitors are well-known to displace reversible cross-links and dissolve networks,^32–38^ and they have also been reported to modulate viscoelasticity,^34^ gelation time,^33,37^ and extrudability.^39^ One recent report from Heilshorn et al. demonstrated this strategy by showing that the sol–gel transitions in hydrazone-based networks could be predicted by incorporating the relative binding strength of the competitor and cross-link into a modified percolation theory.^37^ This model successfully captured how competitive inhibition governs network formation and also probed changes in hydrogel stiffness, competitor diffusion, and gelation time. However, this framework did not provide quantitative predictions of elasticity under competitive inhibition, or extend to dynamic properties such as τ. The limited predictive power reflects, in part, the complexity of introducing a third component, which creates a convoluted equilibria and binding behavior that existing network models do not capture.

While existing hydrogel models struggle with this complexity, other fields have long used simplified frameworks to describe multiequilibrium binding. For example, enzyme kinetics treats competitive inhibition through modified equilibrium constants,^40^ and gas adsorption is captured by Langmuir-type isotherms,^41^ both of which reduce complex binding behavior to tractable mathematical forms. Inspired by these precedents, we proposed a description for dynamic hydrogels in which an ‘apparent’ equilibrium constant (*K*_a,app_), analogous to that in enzyme kinetics, provides a direct and quantitative predictor of *G*_p_ under competitive inhibition by balancing the cross-link equilibrium constant (*K*_a,XL_) with that of the competitor (*K*_a,C_). Additionally, we fit τ using our model combined with a Langmuir-type decay function as an initial approximation of its dependence on competitor concentration. These descriptors can then be incorporated into network elasticity and stress relaxation models to enable predictive insight into hydrogel mechanics in the presence of competitors (Figure 1C). Moreover, the decoupling of dynamic bond association strength from polymer composition via competitive inhibition enables continuum-type changes to gel mechanics (Figure S1).

To validate this model experimentally, we first employed ideal boronate ester hydrogels based on tetra-arm PEG macromers and introduced a series of 1,2- and 1,3-diols with varying association strengths. This hydrogel system is well characterized, biocompatible, and clinically relevant, making it an ideal platform for model validation.^10,11,42^ Binding interactions between the cross-links and competitors were quantified using isothermal titration calorimetry (ITC), and material properties were characterized by oscillatory shear rheology. By incorporating thermodynamic data with mechanical measurements, we successfully modeled experimental values of *G*_p_ with an inhibition-modified network elasticity model and captured τ by a Langmuir-type model. We next applied the analysis to hydrazone-cross-linked hydrogels to show that competitive inhibition principles extend across distinct dynamic chemistries. Finally, we used the model to rationalize injectability, illustrating how competitor binding can transform gels from nonextrudable to hand-injectable. These results establish our model as a broadly applicable strategy for linking molecular competitive inhibition to hydrogel mechanics and function.

## Results and Discussion

### Competitive Inhibition Model for Predicted Network Modulus

To assess whether *K*_a,app_ can be applied to predict hydrogel properties under competitive inhibition, we began from the established principle that the *K*_a_ of the cross-link in dynamic networks directly influences elastic properties.^10,11^ This is because *K*_a_ determines the fraction of cross-links formed at equilibrium; a higher *K*_a_ increases the probability that two binding partners associate, producing more cross-links and thus a higher density of elastically active chains. To describe how competitor alters this equilibrium, we drew inspiration from Michaelis–Menten enzyme inhibition models, in which competitive binding reduces the apparent affinity of the substrate.^40^ We therefore define *K*_a,app_, for cross-link formation in the presence of a competitor as

1 $$K_{a,app}=\frac{K_{a,XL}}{1+K_{a,C}\left[C\right]}$$

where *K*_a,XL_ is the intrinsic equilibrium constant for the cross-link formation, *K*_a,C_ is the binding constant between the competitor and binding partner, and [*C*] is the competitor concentration (for full derivation, see eqs S1–S10). It should be noted that when [*C*] or *K*_a,C_ approaches zero, *K*_a,app_ equals *K*_a,XL_, and the original binding affinity of the cross-link is recovered.

This equilibrium determines the fraction of cross-links formed, and thus a higher *K*_a,app_ increases the probability that two binding partners associate, producing a greater conversion of cross-links (*p*) as

2 $$p=\left(1+\frac{1}{2N_{XL}K_{a,app}}\right)-{\left[{\left(1+\frac{1}{2N_{XL}K_{a,app}}\right)}^{2}-1\right]}^{1/2}$$

where *N*_XL_ is the concentration of active cross-linking species in solution.

The value of *p* can be used to calculate the probability of forming a macromer with 3 or 4 arms connected to the percolated network (as those with 0, 1, or 2 connected arms do not contribute to network elasticity). This yields the average quantity of elastically active chains through a mean field approximation (eqs S13–S17, Figure S2), which is used to calculate the *G*_p_ of a polymer network. In an affine network this may be given as

3 $$G_{p}=\upsilon_{e}k_{B}T$$

where υ_e_, is the number density of elastically active chains (chains that form 3 or 4 cross-links, eq S19), *k*_B_ is the Boltzmann constant, and *T* is the absolute temperature.

However, for more dilute systems (such as those presented here), it is more appropriate to use a phantom model for network elasticity, which accounts for the movement of strand junctions. Here, *G*_p_ is reduced according to the number density of elastically active junctions (μ, eq S18):

4 $$G_{p}=\left(\upsilon_{e}-\mu \right)k_{B}T$$

In summary, *K*_a,app_ determines *p*, *p* determines υ_e_, and υ_e_ determines *G*_p_ through eqs 3 or 4. (For a full derivation, see section “Rubber Elasticity Models for Competitively Inhibited Gels” in the SI). Thus, if we know (or can measure) *K*_a,XL_, *K*_a,C_, and [*C*], the stiffness of a competitive network can be predicted. Importantly, varying *K*_a,C_ and [*C*] unlocks a broad and continuous range of accessible moduli within a single polymer network formulation. This provides a powerful tool to both predictive control and purposeful design of materials that respond to specific molecular cues.

Using this model necessarily involves assumptions regarding network architecture and elasticity (see section “Model Assumptions” in the SI, Figure S2). While we employ the phantom network model here, the *K*_a,app_ approximation can be incorporated into alternative elasticity frameworks that account for different polymer architecture or network defects.^43–46^

### Measuring Cross-link Competitor Thermodynamics and Kinetics for Boronate Ester Linked Gels

To evaluate the framework’s ability to quantitatively capture competitive inhibition in dynamic hydrogels, we turned to a well-studied boronate ester cross-linking system and measured the equilibrium constants (*K*_a,XL_ and *K*_a,C_) between the boronic acid and diol containing competitors that served as inputs to the model.^47^ We selected a chemically and functionally diverse library of small-molecule competitors bearing one or more diol motifs, including a simple sugar (glucose, **1**),^48^ a neurotransmitter (dopamine, **6**),^49^ and pharmaceutical drugs such as dyphylline (**2**)^50^ and capecitabine (**4**).^51^ These competitors were intentionally chosen to represent molecules that are either naturally present *in vivo* (e.g., glucose and dopamine) or therapeutically relevant, enabling us to demonstrate how this strategy can be applied to biologically responsive materials and drug responsive hydrogels. In addition, we synthesized a small-molecule analogue of the cross-link (**5**, Figure S6) to serve as a control competitor with similar dynamics to the cross-link itself.

To measure the relevant binding constants, we synthesized monofunctional PEG analogues (mPEG) bearing 3-fluorophenylboronic acid (mPEG-3FPBA) and gluconic acid (mPEG-GA) to mimic the local chemical environment of the network (Figures S5, S9, S10). Binding measurements were carried out using ITC, in which mPEG-3FPBA was titrated into solutions of either mPEG-GA (to determine *K*_a,XL_) or small-molecule containing diol (to determine *K*_a,C_) (Figure 2A). Data were analyzed with AFFINImeter software using the kinITC method,^52^ which extracts both *K*_a_ as well as dissociation rates (*k*_off_) from the equilibrium profiles.^10,53,54,55^ The resulting *K*_a_ and *k*_off_ values are summarized in Table S8. The boronate ester cross-link exhibited a *K*_a,XL_ of 2,200 ± 100 M^–1^ and a *k*_off_ of 0.26 ± 0.11 s^–1^. anned a wide range of binding affinities and dissociation rates, with *K*_a,C_ values from 6.2 M^–1^ to 6,700 M^–1^ and *k*_off_ values 0.06 s^–1^ to 238 s^–1^, consistent with prior reports (Figure 2B).^42,47,56,57^ While dissociation rates of the cross-link have previously been used to predict network relaxation times in dissociative systems, the *k*_off_ values of the competitors could not be incorporated into our stress-relaxation model in a physically meaninful way. We tehrefore report these values for completeness, as they may be useful for future studies. All ITC experiments were performed in triplicate, and reported values represent the mean and standard deviation (SI Figures S17–S38, Tables S1–S8).

**Figure 2:**
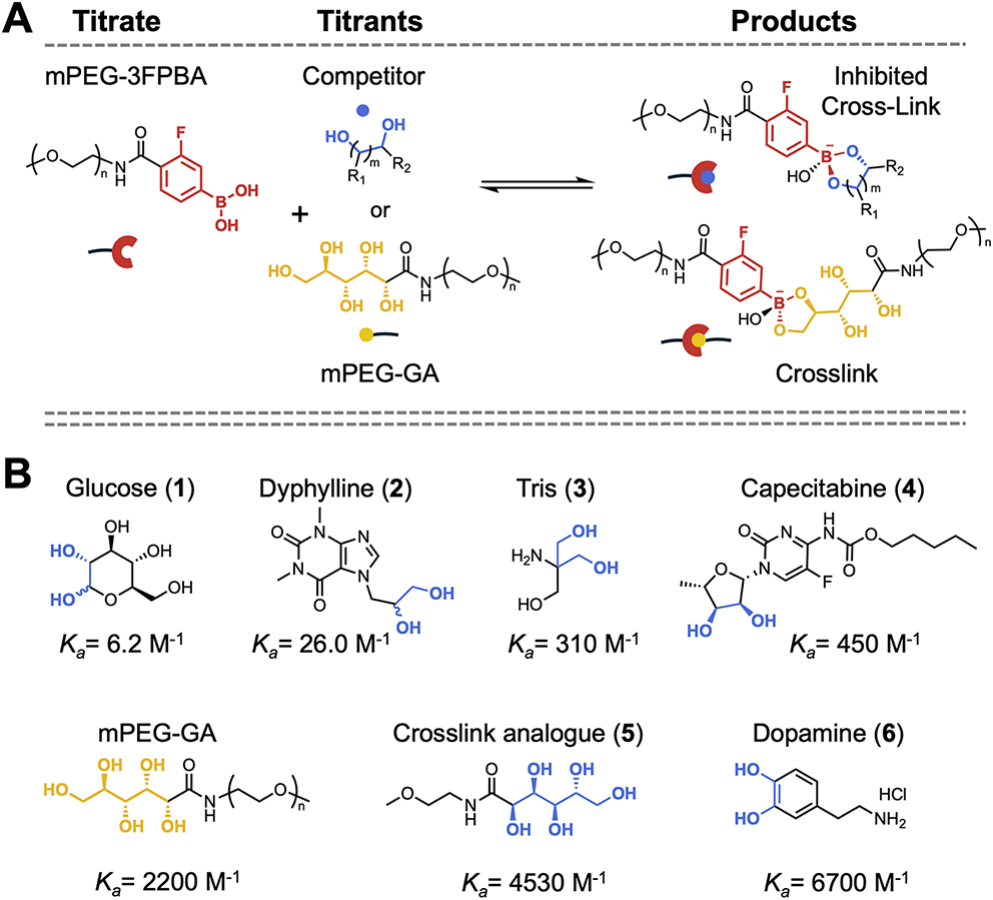
Boronate ester cross-link and competitor thermodynamics. (A) Schematic of mPEG-3FPBA forming reversible complexes with competitors (where *m* = 1, 2), and mPEG-GA cross-links for characterization by ITC. (B) Diol containing small molecules studied in this work, with association constants (*K*_a,C_) spanning 6.2–6700 M^–1^ and cross-link mPEG-GA (*K*_a,XL_) = 2200 M^–1^.

#### Formation and Characterization of Boronate Ester Network Under Competitive Inhibition

With the molecular thermodynamic parameters established, we next characterized how competitive inhibition alters the mechanical properties of bulk hydrogels. End-functionalized tetra-arm PEG (tetraPEG, 5 kDa) bearing either 3FPBA (tetraPEG-3FPBA) or GA (tetraPEG-GA) were synthesized following established literature procedures (SI Figures S3–S4, S7–S8).^58,59^ Mixing 10% w/v solutions of each polymer (corresponding to ≈76 mM reactive end groups) prepared in 0.1 M HEPES buffer (7.4 ± 0.1 pH) yielded boronate ester cross-linked hydrogels, with an effective concentration of ≈38 mM, equivalent to the variable *N*_XL_, of each complementary end group after gelation. To introduce competitive inhibition, small-molecule diol solutions were prepared and used to dissolve the diol-functionalized polymer prior to gelation (Figure 3A).

**Figure 3:**
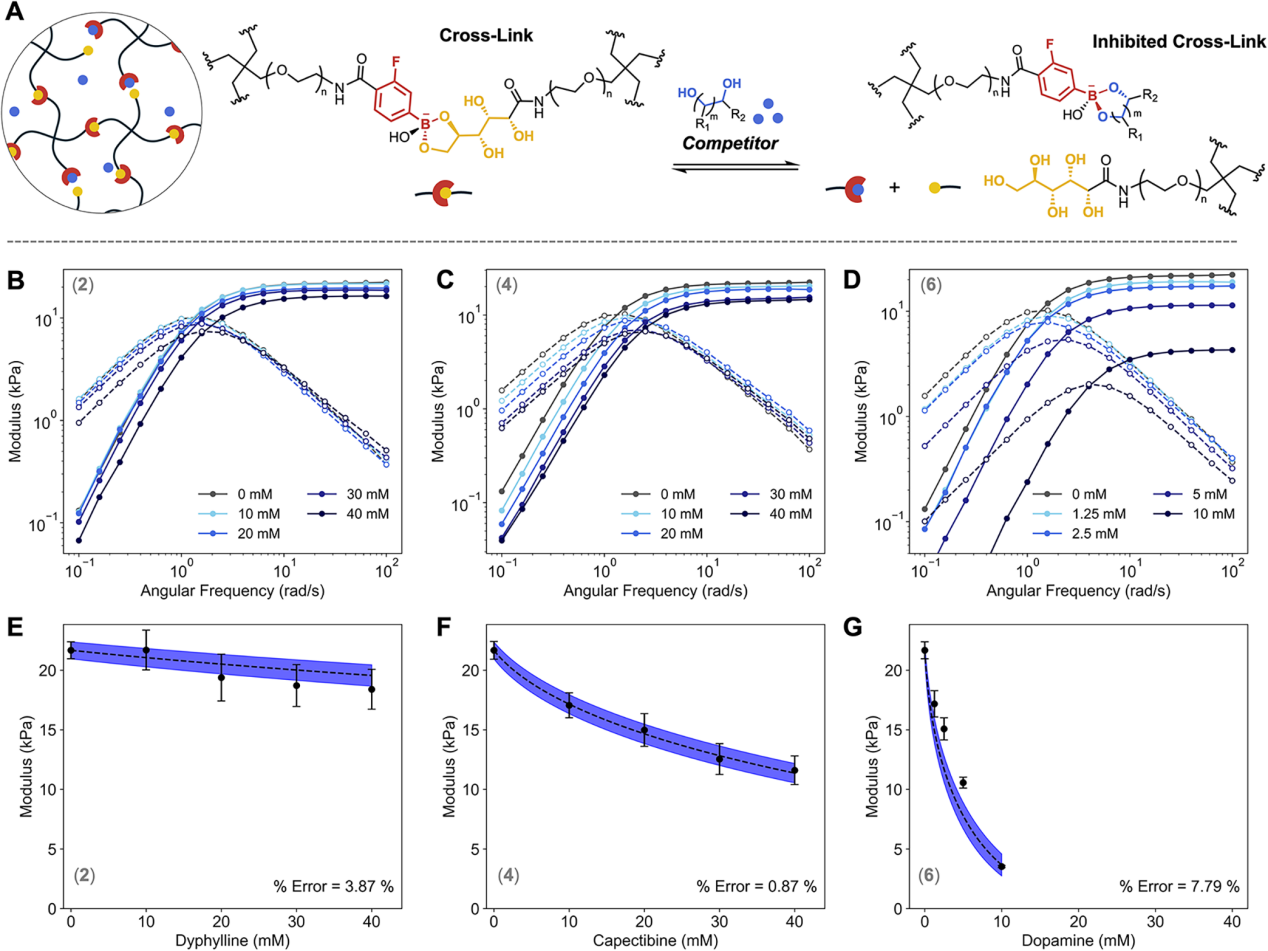
Experimental measurements and model predictions of the competitive inhibition framework for hydrogel stiffness. (A) Schematic of the two states (cross-linked and inhibited) in equilibrium after network formation. (B–D) Representative frequency sweeps showing storage (solid) and loss (dashed) moduli for uninhibited gels (gray) and gels with increasing concentrations of a weak (**B, competitor 2**), medium (**C, competitor 4**), and strong (**D, competitor 6**) competitor at various concentrations. (E–G) Comparison of experimental storage moduli (points) with prediction from the competitive inhibition model (blue region, eq S20) with ranged propagated from ITC error from competitor **2 (E)**, competitor **4** (F), and competitor **6 (G)**. The reported % error corresponds to the mean absolute deviation between experiment and model, normalized by the uninhibited *G*_p_.

The mechanics of uninhibited and inhibited gels were evaluated by small-amplitude oscillatory shear rheology. The uninhibited gel exhibited an average *G*_p_ of 21.7 ± 1.0 kPa with a crossover frequency (ω_*c*_) of 1.38 ± 0.06 rad/s. Introduction of competitors led to systematic decreases in *G*_p_ and increases in ω_*c*_, consistent with progressive disruption of cross-linking.

Representative frequency sweep for weak (*K*_a,C_ < *K*_a,XL_, **1, 2**), moderate (*K*_a,C_ ∼ *K*_a,XL_, **3**, **4**), and strong competitors (*K*_a,C_ > *K*_a,XL_ **5**, **6**) are shown in Figure 3B–D, with full triplicate data summarized in the Supporting Information (SI, Figure S40–S45, Table S9–15). While weak competitors produced only minor reductions in *G*_p_, strong competitors caused substantial softening. For example, with just 10 mM of competitor **6** (0.25 equiv. to the functionalized FPBA groups in solution), we observed an 83% decrease in *G*_p_ and a 200% increase in ω_*c*_ (Figure 3D). These results confirm that a wide range of material properties can be achieved simply by introducing small molecule competitors, leading to decreased stiffness and faster dynamics consistent with our prediction and prior observations (Figure S1D).^33,37,38^ Upon addition of competitor, the gels remained optically clear, and, increasing competitor concentration resulted in gels that qualitatively flow faster (Figure S39). Lastly, to test whether competitors could alter material properties postgelation, solid competitor was weighed and placed on top of the gel. Addition of 30 mM of (**6**) led to complete dissolution of the gel within 1 min, while addition of 40 mM of (**4**) resulted in a *G*_p_ equivalent to that obtained when the competitor was added to gelation (Figure S46).

#### Predicting Hydrogel Modulus Under Competitive Inhibition

Motivated by these systematic changes, we evaluated whether our model could quantitatively predict mechanical responses, which has not been addressed by previous studies.^33,37,38^ Using the *K*_a_ values determined by ITC, we calculated *K*_a,app_ according to eq 1. These values were then used to estimate the fraction of elastically active chains in the phantom network model (eq 4), yielding predicted *G*_p_ for each competitor concentration (for full details on the derivation of the model refer to the SI, S2–S3). Values were normalized to the uninhibited case for comparison across conditions.

Comparison of model predictions with experimental rheology (Figures 3E–G, S47) showed strong agreement across weak, medium, and strong competitors, with deviations never exceeding 10% relative error. However, for very weak competitors (**1**, **2**), differences in properties were difficult to resolve above instrumental noise of the rheology measurements. The uncertainty in the initial modulus measurement of the uninhibited gel is approximately 0.9% (e.g., 0.197 kPa/21.7 kPa) and compounds when propagated across multiple competitor concentrations. When combined with manufacturer-reported calibration uncertainties of ± 8%, the observed ∼10% deviation between predicted and experimental moduli is consistent with expected instrumental and sample-to-sample variability. Overall, these results demonstrate that competitive inhibition can be quantitatively captured, enabling predictive tuning of hydrogel stiffness.

In addition to predicting *G*_p_ from known binding constants, a key advantage of our model is that it can also be applied in reverse. If either *K*_a,XL_ or *K*_a,C_ is unknown, experimental *G*_p_ with added competitor can be fit with the same equations to extract the missing binding parameter (Figures S48–S49). This approach was most accurate for moderate and strong competitors, where fitted values (*R* ^2^ > 0.85) were in reasonable agreement with ITC results. For weak competitors, however, the small decreases in *G*_p_ were within the rheometer error, making it difficult to distinguish true effects. We anticipate that this reverse application will be particularly valuable for determining *K*_a,XL_, which has long been challenging to measure reliably using small-molecule analogues. The model, and the reverse calculations for *K*_a,C_ and *K*_a,XL_ are available on our GitHub.^60^

#### Modeling Stress Relaxation Dynamics under Competitive Inhibition

A defining feature of dynamic polymer networks is their ability to undergo terminal relaxation, or flow, at long time scales. τ reflects how long it takes for sufficient dynamic bonds to dissociate instantaneously to break the percolated network architecture, and it plays an important role in processes such as cellular mechanotransduction or adhesion.^61–63^ In rheology, τ can be extracted from stress relaxation fits (Figure 4B) or estimated from the ω_c_ in a frequency sweep.

**Figure 4:**
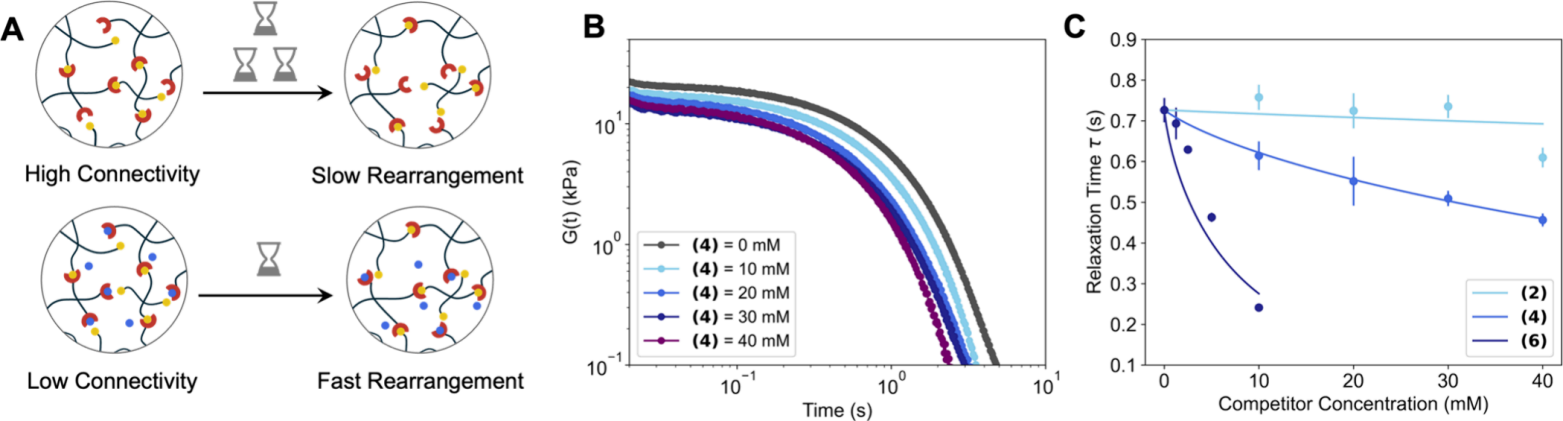
Effect of competitive binding on stress relaxation. (A) Schematic showing that fewer cross-links lead to faster network rearrangement in inhibited polymer networks. (B) Representative stress relaxation curves with increasing concentrations of moderate competitor **(4)**. (C) Relaxation times extracted from ω_c_ data fit to a Langmuir-type decay model, showing systematic acceleration with competitor concentration.

Stress relaxation data collected at varying competitor concentrations were fit to the Kohlrausch–Williams–Watts (KWW)^64,65^ equation:

5 $$\frac{G\left(t\right)}{G_{0}}=\exp\left(-{1\left(\frac{t}{\tau_{kww}}\right)}^{\beta }\right)$$

where $\frac{G\left(t\right)}{G_{0}}$ is the normalized modulus as a function of time, *t* is time, τ_*kww*_ is the characteristic relaxation time obtained from the KWW fit, and β is the stretching exponent ranging from 0 to 1. Note that when β = 1, eq 5 reduces to the single element maxwell model. We compared τ extracted from the crossover frequency, the KWW fits, and the single-element Maxwell fits, and found that all three methods yielded comparable values (Table S16). The β values obtained from KWW fits were close to 1, indicating a narrow distribution of relaxation times and predominately Maxwellian relaxation behavior, as expected for networks dominated by a single dissociative exchange process.^11^ For the subsequent analysis, we used the τ values obtained from the ω_*c*_ to minimize overfitting.

Competitive binding lowers *v*_e_, which reduces *G*_p_ and accelerates relaxation. In other words, fewer cross-links create more pathways for rearrangement, leading to faster stress relaxation (Figure 4A). To capture this relationship, we turned to a Langmuir-type model as an initial descriptor. In the Langmuir adsorption model, a finite number of sites can be occupied, and the extent of adsorption depends on concentration and affinity (Figure S50).^41^ Dynamic hydrogels under competition behave in a similar way: the network has a finite number of reversible cross-linking sites, and the extent of competitor binding reduces the fraction that remain elastically active (*v*_e_). Guided by this analogy and its use in describing competing binding in other contexts,^66^ we fit our data with a decay form of the Langmuir model:

6 $$\tau \left(\left[C\right]\right)=\tau_{0}-\left(\tau_{0}-\tau_{\min}\right)•\left(\frac{2v_{e}}{N_{XL}}\right)$$

where τ­([*C*]) is the relaxation time as a function of competitor concentration, [*C*], τ_0_ is the relaxation time without competitor, τ_min_ is a fitted parameter representing the minimum relaxation time of the network, corresponding to an asymptotic limit as [*C*] → ∞ for each distinct competitor. This model requires only one fitted parameter (τ_min_), with all other terms obtained from experiment or calculation, and it reproduces the observed acceleration of relaxation with increasing competitor (Figure 4C). For strong competitors τ_min_ approaches zero, potentially representing relaxation near the gelation point. It should be noted that eq 6 assumes Maxwellian relaxation, as the τ values are extracted from the crossover frequency, however, we anticipate that the model could be modified for the stretched exponential format, for non-Maxwellian systems.

While eq 6 held across most competitors (Figures 4C, S51), a notable exception was competitor 3 (Tris), which showed increasing relaxation times with competitor concentration, despite the decrease in *G*_p_ (Figure S52). We attribute this inversion to pH effects: competitor 3 raised the pH to 7.93, outside the HEPES buffer range of 7.4 pH. Such increases are known to slow *k*_off_ for boronate ester cross-links, counteracting the expected acceleration.^10^ This result highlights that environmental variables such as pH, ionic strength, or temperature, can independently modulate network dynamics and must be controlled for predictive modeling. We adjusted the formulation for competitor three to have constant pH of 7.4 and constant ion concentration of 80 mM (Figure S53). In this study, these parameters were intentionally held constant to isolate the effects of competitive binding, however, systematic variation of additional physiologically relevant variables represents an important direction for future work.

### Extending Competitive Inhibition Models to Hydrazone Cross-Links

To demonstrate that our framework extends beyond boronate ester gels, we next examined a hydrazone cross-linking system (Figure 5A). Hydrazone cross-linking is a benchmark system in dynamic hydrogel design offering enhanced water stability. The benzyl hydrazone linkage proceeds through a distinct, slower dissociative bond-exchange mechanism compared to boronate esters.^67–69^

**Figure 5:**
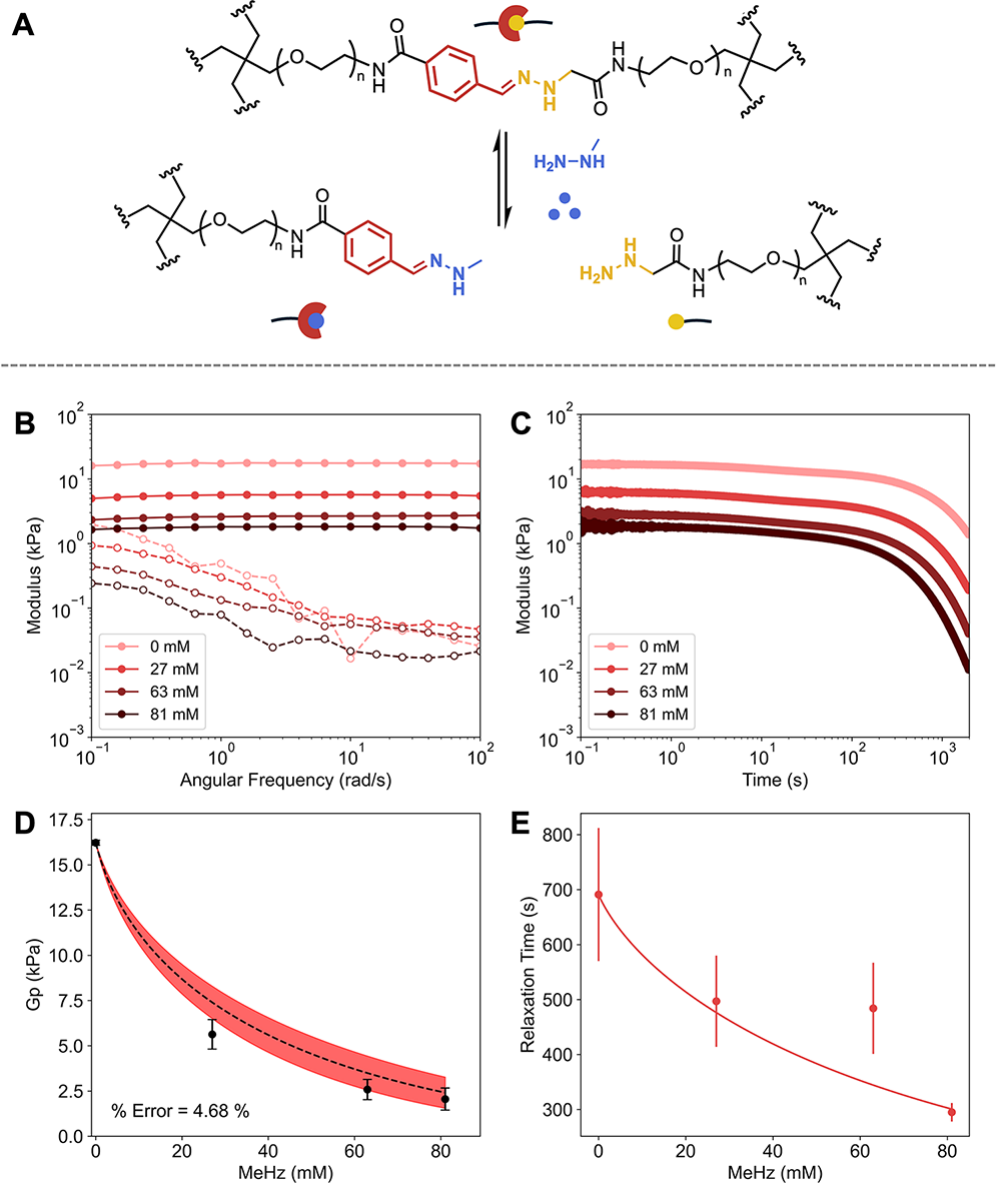
Experimental measurements and model predictions of the competitive inhibition framework for hydrazone cross-linked hydrogels. (A) Schematic of the two states (cross-linked and inhibited) in equilibrium after network formation. (B) Frequency sweeps showing storage (solid) and loss (dashed) moduli for gels with increasing MeHz concentrations. The loss modulus does not exhibit substantial change consistent with prior reports of the slow exchange of the benzyl hydrazone bond.^70^ (C) Stress relaxation curves with increasing concentrations of MeHz. (D) Comparison of experimental storage moduli (points) with prediction from the competitive inhibition model (red region, eq S20) with ranged propagated from ITC error from competitor. (E) Relaxation times extracted from stress relaxation data fit to a Langmuir-type decay model (eq 6).

TetraPEG precursors functionalized with hydrazine and benzaldehyde were synthesized according to previously established methods and mixed at 10% w/v to form hydrazone cross-linked hydrogels (Figures S11, S13, S14), and methyl hydrazine (MeHz) was selected as a competitor. MeHz increased pH values, which in turn slowed τ values (similar to **3** in the boronate ester system). Thus, we carefully held both pH and salt concentrations constant to isolate the effects of competitive inhibition on hydrogel mechanics.^71,72^

mPEG analogues of the macromers were synthesized for measuring the dynamics of the system (S12, S15, S16). We first collected *K*_a_ values for the cross-link (1350 ± 120 M^–1^) and the MeHz (510 ± 60 M^–1^) competitor (Figure S54–S65, Tables S17–S20). We next examined how competitive inhibition altered hydrogel mechanics. Consistent with our prior observations, increasing competitor concentration reduced *G*_p_ (Figure 5B) and accelerated τ (Figure 5C) when pH and salt concentrations were held constant. Importantly, the competitive inhibition model (eq S20) captured the observed *G*_p_ changes with high accuracy (Figure 5D, <5% relative error). Likewise, τ fit well with the same Langmuir-type decay model (eq 6) applied in the boronate ester system (Figure 5E, Table S21).

Beyond our own model, we applied the framework developed by Heilshorn *et. al*, which provides a description of sol–gel transitions in reversible hydrazone networks.^37^ Using this approach, we show that our competitive inhibition formulations follow the same sol–gel phase diagram reported in that work, with formulations forming gels in agreement with the predicted phase boundaries (Figure S66).

These results show that our predictions extend beyond boronate ester gels to dynamic cross-links with different time scales of bond exchange. This underscores our model’s potential as a unifying framework for predicting how binding under competitive inhibition governs the mechanics of diverse dynamic polymer networks.

### Expanding Material Function Through Competitive Inhibition

Cross-link density and relaxation govern not only stiffness and stress relaxation, but also functional properties of dynamic hydrogels, including self-healing, swelling, and injectability. Under competitive inhibition, we observed predictable shifts in these behaviors: self-healing slowed (S70–S71) and equilibrium swelling increased (Figure S67, Table S22), consistent with the reduction in cross-link density. Because swelling provides a pathway for small-molecule diffusion, reversibility of network mechanics through competitor removal was also evaluated. In hydrazone-cross-linked networks, swelling enabled diffusion and removal of the competitor, as confirmed by UV–vis analysis of the surrounding buffer, and rehydration of the lyophilized gel to the original concentration recovered mechanical properties comparable to those of the uninhibited material (Figures S68–S69). While these observations align with expectation, more detailed studies are needed to quantitatively link competitor binding to these properties.

Injectability is a complex yet practically important property across applications ranging from therapeutic delivery to bioprinting and materials manufacturing. In each case, materials flow under applied stress and maintain structure after placement or extrusion. Prior work has shown that the addition of competitors can sometimes improve injectability by softening the network, but these effects have generally been empirical and system-specific.^23,24^

To test whether our framework could connect competitive inhibition to injectability through its impact on *G*_p_, we performed injection tests on syringes loaded with boronate ester gels using an Instron mechanical tester (Figure 6A). During compression, the force increased to a yield point before plateauing at steady-state injection (Figure 6B). While uninhibited gels required ≈76 N of force, well above the 38 N threshold for hand-injectability, adding just 5 mM of competitor **6** reduced the injection force to ≈ 43 N, and 10 mM reduced it further to ≈25 N, converting the gels from nonextrudable to comfortably injectable (Supplementary Video).

**Figure 6:**
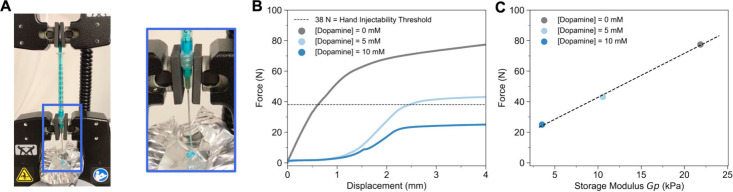
Linking competitive inhibition to hydrogel injectability. (A) Syringe compression set up using an Instron. (B) Injection force profiles of boronate ester gels with increasing concentrations of competitor **6**, showing reduced force with increasing inhibition. (C) Linear best fit between injection force and *G*_p_, enabling prediction of injectability from competitive inhibition inputs.

As an initial approximation, *G*_p_ in our system is linearly correlated to injection force (Figure 6C). While injectability is influenced by τ, *G*_p_, and multiple other factors, prior studies have often simplified it to a positive linear correlation with *G*_p_.^23,73^ Our model gives control over *K*_a,app_ which allows for changes in *G*_p_ which further extends to modulating injection force. This result highlights how molecular-scale competitive binding can be translated into macroscopic material function, expanding the utility of the model beyond mechanical descriptors to clinically and technologically relevant properties. Previous studies have demonstrated cytocompatibility, cell encapsulation, and controlled drug release in related dynamic hydrogel systems, suggesting that this framework is well-positioned for application in these contexts.^21,67,74,75^ However, reducing effective cross-link density to improve injectability necessarily alters nonmechanical properties, such as network mesh size;^76^ which may be relevant for uses in applied contexts such as drug diffusion and delivery. Systematic evaluation of formulations is required to avoid unintended effects.^4,5^

## Conclusion

We proposed and experimentally validated a competitive inhibition framework that quantitatively links molecular binding equilibria of cross-links to the macroscopic mechanics of dynamic polymer networks. Drawing inspiration from biochemical models, we introduced apparent equilibrium constants (*K*_a,app_) to describe competing equilibria and predict how competitive inhibition alters network properties. The model predicted *G*_p_ of various competitors within 10% relative error. Extending the model to hydrazone gels confirmed its generality across distinct dynamic chemistries with different exchange mechanisms and time scales. A limitation of the framework is that network defects, and environmental sensitivities are not yet accounted for we hope to expand on these topics in future work. Finally, we demonstrated functional utility by showing that competitors can transform a gel from nonextrudable to hand-injectable without resynthesis. These results position competitive inhibition as a unifying principle for designing responsive polymer networks, offering a predictive route to tune material mechanics for applications ranging from injectable biomaterials to tissue mimics and drug delivery devices.

## Supplementary Material







## Data Availability

GitHub with model. Tools for predicting hydrogel properties under competition (https://github.com/hill-lab-chem/InhibNet).

## Notes

Discussion of model. Instrumentation, General Procedures, and Methods. Synthetic Methods and Spectra. ITC Traces and Data Tables. Rheology Traces and Data Tables. Modulus Predictions. Representative Stress Relaxation Fits. Tau Predictions. UV–vis Titrations. Rheology of Hydrazone. Swelling Test. Self-healing Test (PDF)
Supplementary Video: Qualitative hand injectability of gels with different [dopamine] (MP4)
Transparent Peer Review report available (PDF)

## Abbreviations

ITC: isothermal titration calorimetry
*G*_p_: plateau modulus
τ: relaxation time
*K*_a_: associative equilibrium constant
*K*_a,XL_: associative equilibrium constant of cross-link formation
*K*_a,C_: associative equilibrium constant of cross-link-competitor complex
[*C*]: concentration of competing small molecule
*N*_XL_: concentration of cross-links in solution
ω_c_: crossover frequency
*K*_a,app_: apparent cross-link association constant
*k*_off_: dissociative rate constant
*v*_e_: elastically active strands
**1**: glucose
**2**: dyphylline
**3**: tris
**4**: capecitabine
**5**: cross-link analogue
**6**: dopamine-HCl
mPEG: methoxy poly­(ethylene glycol)
tetraPEG: tetra-armed poly­(ethylene glycol), MW = 5000 Da
3FPBA: 3-fluorophenylboronic acid
GA: gluconic acid
HEPES: 2-[4-(2-hydroxyethyl)­piperazin-1-yl]­ethane-1-sulfonic acid
