# Supplementary material for: Competitive Inhibition as a Tool to Modulate and Predict Dynamic Hydrogel Mechanics
Source: ACS Cent Sci. 2026 Feb 17;12(2):233–42. doi: 10.1021/acscentsci.5c02130 (PMC12947549; doi:10.1021/acscentsci.5c02130)
Supplement: Supplementary file 2 [file oc5c02130_si_002.pdf]

Supporting Information for

Competitive Inhibition as a Tool to Modulate and Predict Dynamic Hydrogel Mechanics

Alexander D. Claiborne [1], Sirilak Mekcham [1], Owen A. Lee [1], Megan R. Hill [1]
Department of Chemistry, Colorado State University, 301 W Pitkin, Fort Collins, CO 80521-1872, United States

Table of Contents

1. Discussion **3**
- *1.1. Equilibrium Constants Under Competitive Inhibition* *3*
- *1.2. Rubber Elasticity Models for Competitively Inhibited Gels* *4*
- *1.3. Design Space Available in Model* *7*
- *1.4. Model Assumptions* *8*

2. Instrumentation, General Procedures, and Materials **9**

3. Synthetic Methods **11**
- *3.1. Synthesis of 5 kDa 4PEG-GA* *11*
- *3.2. Synthesis of 2 kDa mPEG-GA* *11*
- *3.3. Synthesis of (2R,3S,4R,5R)-2,3,4,5,6-pentahydroxy-N-(2-methoxyethyl)hexanamide (Cl-ana) 11*
- *3.4. Synthesis of 5 kDa 4PEG-3-fluorophenylboronic acid* *12*
- *3.5. Synthesis of 2 kDa mPEG-3-fluorophenylboronic acid* *12*
- *3.6. Synthesis of 5 kDa 4PEG-benzyl aldehyde (5kDa 4PEG-Ar-CHO):* *13*
- *3.7. Synthesis of 2 kDa mPEG-benzyl aldehyde (2kDa mPEG-Ar-CHO):* *13*
- *3.8. Synthesis of 5 kDa 4PEG-hydrazine (5kDa 4PEG-Hz):* *13*
- *3.9. Synthesis of 2 kDa mPEG-hydrazine (2kDa mPEG-Hz):* *15*

4. ITC **23**
- *4.1. ITC Procedures* *23*
- *4.2. Glucose (1)* *23*
- *4.3. Dyphylline (2)* *26*
- *4.4. Tris (3)* *28*
- *4.5. Capecitabine (4)* *30*
- *4.6. mPEG-GA* *32*
- *4.7. Cl-ana (5)* *34*
- *4.8. Dopamine (6)* *36*

4.9. *ITC Data Summary* 38

5. Rheology 39

5.1. *Formation of Hydrogels:* 39

5.2. *Zero Competitor* 40

5.3. *Glucose (1)* 41

5.4. *Dyphylline (2)* 42

5.5. *Tris (3)* 43

5.6. *Capecitabine (4)* 44

5.7. *Crosslink-analogue (cl-ana) (5)* 45

5.8. *Dopamine (6)* 46

5.9. *Addition of Competitive Inhibitor Post-Gelation* 47

6. Modulus Predictions 48

6.1. *Predicting Modulus From K_a Crosslink and K_a of Competitor* 48

6.2. *Fitting for $K_{a,XL}$ From Known $K_{a,C}$* 49

6.3. *Fitting for $K_{a,C}$ From Known $K_{a,XL}$* 50

7. Tau Predictions 51

7.1. *Representative Stress Relaxation Fits* 51

7.2. *Modeling Tau Under Competitive Inhibition* 52

8. Measuring K_a in Hydrazone Crosslinked Hydrogel System 55

8.1. *UV-Vis Titration for MeHz Competitor K_a Determination* 55

8.2. *UV-Vis Titration for Hydrazone Crosslink K_a Determination* 60

9. Rheology Values for Hydrazone Gels 64

10. Swelling Test 65

11. Self-healing Test 68

References 69

1. Discussion

1.1. Equilibrium Constants Under Competitive Inhibition

To model the competitive inhibition of reversible polymer crosslinks, we adopted a framework inspired by competitive binding models commonly used in chemical biology. This section summarizes the assumptions used to derive an effective equilibrium constant for crosslink formation in the presence of competitors.

1.1.1. Definition of Binding Equilibria

We consider a dynamic polymer network in which crosslinks form through the reversible association of two complementary binding partners: $A + B \rightleftarrows [AB]$, where $[AB]$ denotes an active crosslink. The association constant for the active crosslink formation is defined as,

$$K_{a,XL} = \frac{[AB]}{[A][B]} = \frac{k_{on}}{k_{off}} \tag{S1}$$

which can be rearranged to express the concentration of active crosslinks as

$$[AB] = K_{a,XL}[A][B] \tag{S2}$$

We next introduce competitive binding, where a competitor molecule *C* reversibly binds to one half of the crosslink (either A or B), forming an inactive complex $A + C \rightleftarrows AC$. The equilibrium constant for this competing interaction is defined as

$$[AC] = K_{a,C}[A][C] \tag{S3}$$

1.1.2. Mass balance and crosslink concentration

The total concentration of A can be expressed by:

$$[A_{total}] = [A] + N_{XL} + [AC] \tag{S4}$$

Substituting eqs (S2) and (S3) into eq (S4), and simplifying yields the concentration of free A:

$$[A] = \frac{[A_{total}]}{1+K_{a,XL}[B]+K_{a,C}[C]} \tag{S5}$$

Next, substituting eq (S5) into eq (S2) gives the concentration of active crosslinks as

$$[AB] = \frac{K_{a,XL}[A_{total}][B]}{1 + K_{a,XL}[B] + K_{a,C}[C]} \tag{S6}$$

However, eqs (S5) and (S6) are difficult to use practically, as they require knowledge of the instantaneous concentrations of all bound and unbound species in the system, which are not readily accessible experimentally.

1.1.3. Effective Equilibrium Constant Under Competitive Inhibition

Rather than solving eq (S6) explicitly, we adopt an approach commonly used in chemical biology to describe competitive inhibition at equilibrium, which provides an effective equilibrium constant that captures the influence of competitor binding.

In Michaelis–Menten kinetics, the apparent Michaelis constant K_m^{app} changes as a function of competitor concentration ($[C]_0$) and competitor affinity ($K_{d,C}$).[1]

$$K_m^{app} = K_m\left(1 + \frac{[C]_0}{K_{d,C}}\right) \quad \text{(S7)}$$

In enzyme binding, an enzyme (*E)* reversibly binds a substrate (*S)* to form an enzyme–substrate complex (*ES)*, which is subsequently converted to product (*P)*:

$$S + E \underset{k_{off}}{\overset{k_{on}}{\rightleftarrows}} ES \overset{k_{cat}}{\longrightarrow} E + P \quad \text{(S8)}$$

The Michaelis constant is defined as $K_m = (k_{off} + k_{cat})/k_{on}$. In contrast, dynamic networks do not involve product formation, so k_{cat} = 0 and $K_m = k_{off}/k_{on} = K_d$.

Thus, for crosslinking equilibria, the dissociation constant is $K_d = [A][B]/[AB] = k_{off}/k_{on}$ and the corresponding association constant is $K_a = 1/K_d$. Applying the competitive inhibition framework to crosslink formation then gives

$$\frac{1}{K_{a,app}} = \frac{1}{K_{a,XL}}\left(1 + K_{a,C}[C]_0\right) \quad \text{(S9)}$$

Taking the inverse yields a simple expression for the apparent association constant in the presence of competitor:

$$K_{a,app} = \frac{K_{a,XL}}{1 + K_{a,C}[C]_0} \quad \text{(S10)}$$

1.1.4. Practical Implications

Equation (S10) provides a practical means of predicting how competitive inhibition reduces the effective crosslinking strength. Importantly, estimating Ka,app requires only:

(i) The crosslink association constant $K_{a,XL}$
(ii) The competitor association constant $K_{a,C}$
(iii) The initial competitor concentration $[C]_0$

This approach avoids the need to determine the instantaneous distribution of bound and unbound species, enabling direct comparison between experimental conditions and simplifying analysis of competitive, dynamic polymer networks.

1.2. Rubber Elasticity Models for Competitively Inhibited Gels

To predict the plateau shear modulus G_p, several theoretical descriptions of reversible polymer networks have been developed. Zhao *et. al* established a mean-field, Bell-type description for reversible ideal polymer networks that links explicitly links molecular association equilibria to macroscopic viscoelastic response.[2,3] More recently, Spakowitz *et. al,* introduced a *brachiation* model in which network mechanics arise from stochastic association and dissociation events along polymer chains, with bound-state statistics treated using a Poisson framework.[4,5]

In this work, we adopt the Zhao framework as an initial approximation because it provides a direct and analytically tractable connection between equilibrium binding parameters and bulk mechanical properties. This feature makes the model well suited for incorporating the apparent association constant $K_{a,app}$, introduced here to describe competitive inhibition of reversible crosslinks. Importantly, because the competitive inhibition formalism itself is general, $K_{a,app}$ could

be incorporated into a variety of network elasticity models.[2,3] In this work, we demonstrate its implementation for ideal polymer networks assuming either affine or phantom elasticity.

1.2.1. *Rubber Elasticity Models*

In rubber elasticity theory, the plateau shear modulus, G_p, which reflects network stiffness, is proportional to the density of elastically active chains υ_e. For an affine network this relationship is given by:

$$G_p = \upsilon_e k_B T \tag{S11}$$

For a phantom network, which accounts for junction fluctuations, the modulus is instead:

$$G_p = (\upsilon_e - \mu) k_B T \tag{S12}$$

where μ is the concentration of cross-link junctions. The affine model is generally applicable to highly crosslinked systems, whereas the phantom model is more appropriate for relatively dilute networks near the overlap concentration, as is the case for the materials studied here.

Elastically active chains are defined as network strands connected through junctions that participate in load-bearing pathways, corresponding to junctions with three or more independent connections. Estimating υ_e and μ therefore requires determining the equilibrium conversion of reversible crosslinks.

1.2.2. Crosslink Conversion Under Competitive Inhibition

Because crosslinks are reversible, their equilibrium conversion *p* depends on both the intrinsic association constant, $K_{a,XL}$, and the concentration of the reactive chain ends N_{XL}.[2,6] In the absence of competitor, the conversion (p_0) is given by:

$$p_0 = \left(1 + \frac{1}{2N_{XL}K_{a,XL}}\right) - \left[\left(1 + \frac{1}{2N_{XL}K_{a,XL}}\right)^2 - 1\right]^{1/2} \tag{S13}$$

To account for competitive inhibition, $K_{a,XL}$ is replaced by the apparent association constant $K_{a,app}$, yielding the conversion in the presence of competitor.

$$p = \left(1 + \frac{1}{2N_{XL}K_{a,app}}\right) - \left[\left(1 + \frac{1}{2N_{XL}K_{a,app}}\right)^2 - 1\right]^{1/2} \tag{S14}$$

This substitution directly links competitor concentration and binding affinity to the fraction of active crosslinks in the network.

1.2.3. Elastically Active Chains in a Tetra-arm Network

For an ideal network formed from tetra-arm macromers, the density of elastically active chains υ_e can then be related to conversion p through the probability that a macromer forms three or four independent connections to the percolated network (thereby contributing to network elasticity). The probability that a given arm leads to a dangling end P_{out} as a function of conversion is:

$$P_{out} = \left(\frac{1}{p} - \frac{3}{4}\right)^{\frac{1}{2}} - \frac{1}{2} \quad \text{(S15)}$$

Using a mean-field approximation,[7] the probabilities of three-arm (P_3) and four-arm (P_4) junctions are given by:

$$P_3 = 4P_{out}(1 - P_{out})^3 \quad \text{(S16)}$$

$$P_4 = (1 - P_{out})^4 \quad \text{(S17)}$$

A three-arm junction contributes 1.5 elastically active chains, while a four-arm junction contributes 2, reflecting the fact that each network strand is shared between two junctions. Two-arm junctions correspond to loops and do not contribute to elasticity, while one-arm represent dangling ends.

The junction density μ and elastically active chain density v_e are therefore

$$\mu = \frac{N_{XL}}{4}(P_3 + P_4) \quad \text{(S18)}$$

$$v_e = \frac{N_{XL}}{4}\left(\frac{3}{2}P_3 + 2P_4\right) \quad \text{(S19)}$$

1.2.4. Predicted Modulus

Substituting egs (s18) and (S19) into the phantom network expression (eq (S12)) and simplifying (as detailed in reference 5) yields the predicted shear modulus:

$$G_0/k_BT = \frac{N_{XL}}{16}\left(3 - \sqrt{\frac{4}{p} - 3}\right)^3\left(\sqrt{\frac{4}{p} - 3} + 1\right) \quad \text{(S20)}$$

Utilizing equation (20) together with $K_{a,app}$ determined from the Michaelis-Menton-type competitive inhibition approximation, the modulus can be predicted as a function of competitor concentration and binding affinity. Fits to experimental data are shown in Figure 6.

Although the same procedure can be applied using the affine network model (eq (S11)), the phantom model provides better agreement for networks near overlap concentration. Because the affine and phantom models represent limiting cases of rubber elasticity, some deviation from experimental values is expected.

To facilitate use of these models as a predictive design tool, we have made the underlying calculations available via an open-source GitHub repository, including a web-based app (https://inhibnet.streamlit.app/) that allows users to explore the influence of network and binding parameters.[8]

1.3. Design Space Available in Model

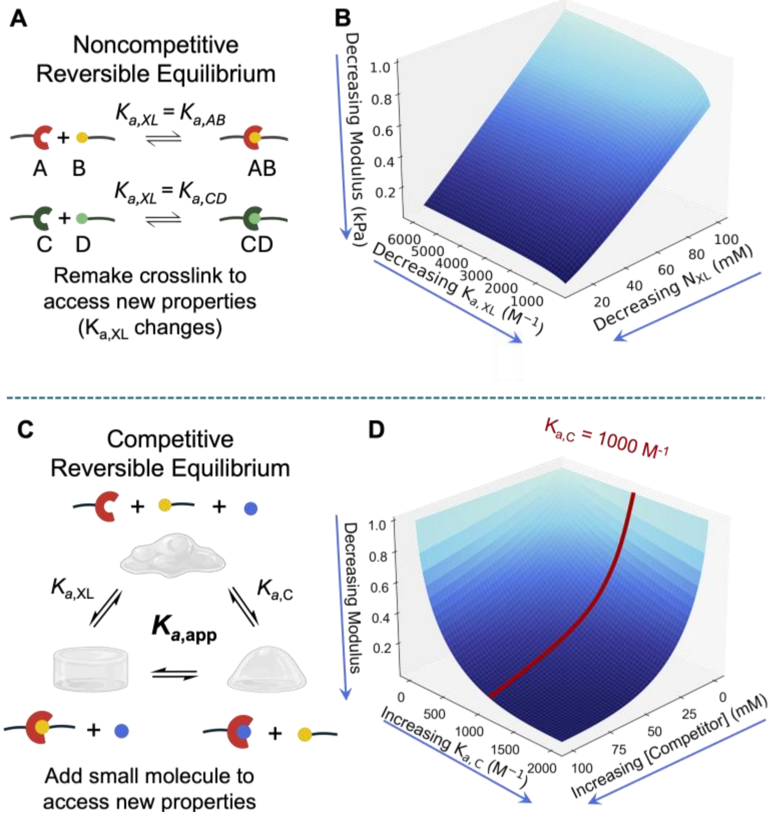

Figure S1. Design space for tuning network mechanics in noncompetitive and competitive reversible polymer networks. **(A)** In a noncompetitive reversible network, material properties are tuned by changing either the crosslink concentration or the crosslinking chemistry. Distinct crosslinker pairs (e.g., AB vs CD) exhibit different crosslink association constants ($K_{a,XL}$), leading to different network stiffnesses, as demonstrated in prior work.[2,3] **(B)** Visualization of the accessible modulus design space for a phantom network in the noncompetitive case. The shear modulus can be tuned by varying either $K_{a,XL}$ or the total crosslink concentration N_{XL}. Changes in $K_{a,XL}$ arise from differences in crosslink identity, whereas changes in N_{XL} can be achieved by preparing the same network formulation at different polymer concentrations. **(C)** In a competitively inhibited reversible network, material properties are tuned by adding competitors that binds to chains. This creates a ternary equilibrium between unbound chain ends, active crosslinks, and competitor-bound inactive complexes. The extent of crosslinking depends on $K_{a,XL}$, the competitor association constant $K_{a,C}$ and the competitor concentration [C] (eq S1 – S10). **(D)** Visualization of the accessible modulus design space for a phantom network in the competitive case, where modulus decreases with increasing $K_{a,C}$, and [C], while the underlying crosslinking chemistry remains unchanged. The red trajectory illustrates how increasing competitor concentration modulates the modulus for a fixed competitor with $K_{a,C}$ = 1000 M^{-1}.

1.4. Model Assumptions

1.4.1. Affine and Phantom Network Models

The gels studied here using the phantom network approximation, which is appropriate for relatively dilute polymer networks near the overlap concentration and accounts for junction fluctuations through a mean-field description of network connectivity as illustrated figure S2.[9,10] For completeness, our published tool also allows use of an affine network model. However we do not capture behavior in-between the phantom and affine network, but we posit more complex network models could incorporate the $K_{a,app}$.

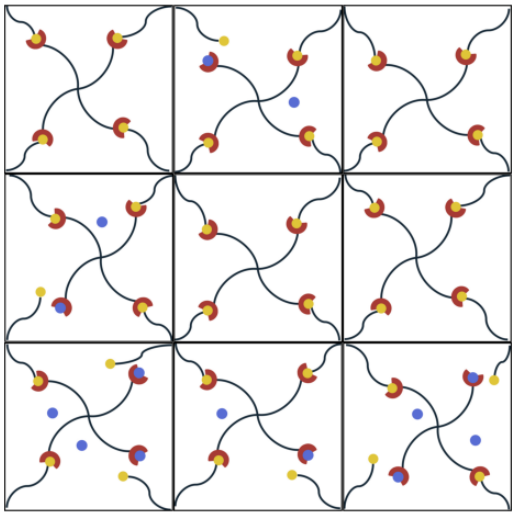

Figure S2. Scheme illustrating the mean field approximation for polymer network under competitive inhibition. The network is split into a mean unit (*e.g.* the unit within a single box). The mean field assumption for dynamic polymer networks calculates the probability of forming different numbers of junctions. We apply the mean field assumptions to the entire polymer network.

1.4.2. K_a is Concentration Independent

We assume that the association constants of both the crosslinks and the competitors remain unchanged across different concentrations. Although the concentrations used to measure crosslink association constants differ from those present in the gel, we assume that these values remain a reasonable approximation of binding behavior in the network environment.

1.4.3. mPEG Binding Approximates 4PEG Binding

Binding measurements in this study are performed using functionalized monofunctional methyl-PEG (mPEG) to probe crosslink–competitor interactions. We assume that these measurements provide a reasonable approximation of the binding behavior of individual strands within the tetra-arm PEG macromers.

2. Instrumentation, General Procedures, and Materials

2.1. Instrumentation

Nuclear Magnetic Resonance spectra spectra were acquired at 25 °C on a Bruker US400 (400 MHz) NMR instrument. Matrix Assisted Laser Desorption/Ionization Time-of-Flight Mass Spectrometry (MALDI-TOF MS) spectra were acquired on a Bruker UltrafleXtreme MALDI-TOF instrument. Rheology spectra were acquired on a Discovery Hybrid Rheometer 20 at 25 °C with either 8 mm or 20 mm parallel plates with a gap height set at 100 mm for the 8 mm plate and 300 mm for the 20 mm plate. Isothermal Titration Calorimetry (ITC) spectra were acquired on a TA Instruments Affinity ITC at 25 °C, with 30 injections of 2 μL. UV-Vis Spectrophotometer (UV/Vis) spectra were acquired on a Cary 3500 Multicell UV-Vis Spectrophotometer. Scans were acquired from 200.00 - 800.00 nm with an average time of 0.020 s. An Instron 68TM-10 Mechanical Tester was used to measure the injectability profiles. The Instron was equipped with a 500 N load cell with 1 kN pneumatic grips with serrated jaw faces. For the samples containing competitors there is an onset period before the force increases. This onset period arises from the initial compression of the gel within the syringe prior to flow through the needle. Once the gel is fully compressed and injection begins, the force reaches a plateau corresponding to the steady-state force required to inject the gel.

2.2. General MALDI Procedure

A 20 mg/mL solution of the polymer product was prepared in THF. A 60 mg/mL solution of DCTB in THF was prepared as a matrix solution. A 15 mg/mL solution of NaTFA in THF was used as the salt solution. To prepare the sample on the plate, 1 µL of sample solution, 20 µL of matrix solution, and 1 µL of salt solution was used. The instrument was set to reflector mode; 1000 shots were fired at a time at 200 Hz.

2.3. General Rheology Procedure

2.3.1. Boronate Ester Crosslinked Gels

10 w/v % 4arm-PEG-FPBA and 10 w/v % 4arm-PEG-GA were prepared by using 10x HEPES buffer. To prepare the samples 100 µL of each PEG solution were combined in a vial. Gels would form within 3 seconds. The samples were then placed on an 8 mm or 20 mm parallel plate geometry. The trim gap was set to 130 mm for 8 mm plates or 330 for 20 mm plates. Excess gel was trimmed, and the geometry was set to 100 mm for the 8 mm plate and 300 mm for the 20 mm plate. Following this mineral oil was applied around the sides of the plates to limit evaporation. The instrument was set to 25 °C. To ensure thermodynamic equilibrium a time sweep was recorded at 10 rad/s, 1% strain, for 300 s. If the modulus of the gel was consistent a frequency sweep was collected from 100 rad/s to 0.1 rad/s at 1% strain. For the stress relaxation experiments, 5% strain steps were measured over 1000 s.

For the rheology experiments, we ensured the material was in equilibrium by initially performing a time sweep at 10 rad/s at 1 % strain for 300 seconds at 25 ºC. Following the time sweep, we performed frequency sweeps from 100 rad/s to 0.1 rad/s at 1% strain, and 25 ºC, collecting 5 points per decade, at varying identify and concentration of competitor. Finally, a stress relaxation experiment was run at 1% strain for 1000 seconds.

Over longer timescales, the materials are bench-stable with or without competitor, remaining in the gel phase for at least six months after formation. However, when placed in excess water, the hydrogels are not water-stable and dissolve within 24 h, consistent with previous work.[11] As a result, reversibility upon competitor removal by dilution is limited under these conditions. However, this behavior may be advantageous in contexts where triggered dissolution or clearance is

desired, such as drug delivery or injectable applications. Long-term stability would require additional network stabilization strategies.

2.3.2. *Hydrazone Crosslinked Gels*

The hydrogels were allowed to set at ambient temperature for at least 12 h. Experiments were performed at a gap of 0.3 mm. The Oscillation time experiment was run at 25 ºC, 1% strain, Angular frequency 10.0 rad/s for 300 s, and a frequency sweep experiment was then performed from 0.1 to 100 rad/s with 1% strain at 25 ºC. After that stress relaxation experiment was performed with 10% strain at 37 ºC for 1000 s.

2.4. *Instron Injectability Test*

10 w/v % 4arm-PEG-FPBA and 10 w/v % 4arm-PEG-GA with varying amounts of competitor were prepared by using 10x HEPES buffer. To prepare the samples 100 µL of each PEG solution were combined in a vial. Gels would form within 3 seconds. The gels were top loaded into 1 mL Air-Tite 2-Part Luer Lock Syringe with a PrecisionGlide 21G x 1" TW needle equipped. The grips were set to be 78 mm apart from each other. The grips were set to compress at a pressure of 15 psi, with one grip on the plunger of the syringe with the other on the screw of the Luer lock. After the syringe was secured in the center of the grips the compression test started at a rate of 0.05 mm/s. The test was set to end after a displacement of 5 mm occurred. The force vs displacement graphs were outputted to excel.

2.5. *Materials*

Triethylamine (TEA) – Fisher (121-44-8)
o-Benzotriazol-1-yl-tetramethyluronium hexafluorophosphate (HBTU) – Chem Impex Intl (94790-37-1)
1-Hydroxybenzotriazole hydrate (HOBt) – Sigma Aldrich (123333-53-9)
Glucono-delta-lactone – Chem Impex Intl (90-80-2)
Tetra-PEG-Amine (5kDa) – JenKem
(3-Formyloxyphenyl)boronic acid – TCI (25487-66-5)
Formylbenzoic acid- Aldrich Chemical (619-66-9)
4-Methylmorpholine- Alfa Aesar (109-02-4)
Tri-Boc-hydrazinoacetic acid- Chem Impex (261380-41-0)
(1-[Bis(dimethylamino)methylene]-1H-1,2,3-triazolo[4,5b]pyridinium3-oxidhexafluorophosphate) (HATU)- Sigma Aldrich (148893-10-1)
Methyl Hydrazine-Aldrich (60-34-4)
Dopamine hydrochloride – Thermo-scientific (62-31-7)
D(+) Glucose anhydrous – Thermo-scientific (50-99-7)
Dyphylline – Thermo-scientific (479-18-5)
Tris – Matrix Scientific (77-86-1)
Capecitabine – Sigma Aldrich (154361-50-9)
2-Methoxyethylamine – Sigma Aldrich (109-85-3)
4-(2-hydroxyethyl)-1-piperazineethanesulfonic acid (HEPES) – Sigma Aldrich (7365-45-9)
PBS Tablets – MP Biomedicals (N/A)
and were used as received unless otherwise noted.

3. Synthetic Methods

3.1. Synthesis of 5 kDa 4PEG-GA

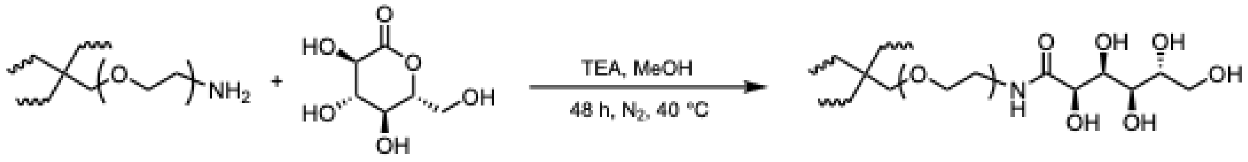

In a procedure adapted from Kang, and Kalow,[12] 5 kDa 4PEG-Amine HCl salt (1.000 g, 0.8000 mmol of amine, 1.000 eq), glucono-delta-lactone (1.143 g, 0.8005 mmol, 8.020 eq), TEA (0.4946 g, 4.888 mmol, 6.11 eq) and a stir bar were placed in a 20 mL scintillation vial filled with 10 mL of methanol. The vial was heated and stirred at 40 °C for 48 hours under nitrogen. The reaction was then precipitated directly into 100 mL of cold diethyl ether. The solution was filtered using a fine fritted funnel and dried under vacuum overnight. After seeing impurities in the ^{1}H NMR, the sample was redissolved in water and dialyzed against water for 3 days,. The sample was lyophilized to yield a white powder (1.012 g, 88.74 %). d = 7.46 (bs, 1H), 4.30 (s, 1H), 4.13 (s, 1H), 3.83 (s, 2H), 3.64 (brs, 114H), 1.25 (s, 1H).

3.2. Synthesis of 2 kDa mPEG-GA

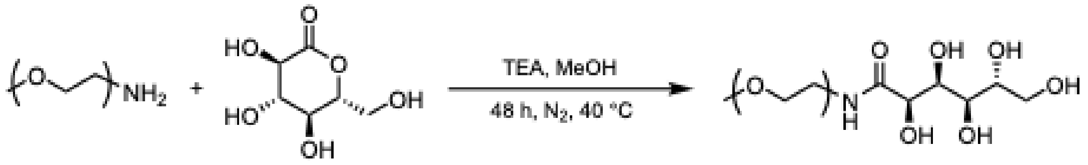

In a procedure adapted from Kang, and Kalow,[12] 2 kDa methoxyPEG-amine HCl salt (0.5000 g, 0.2500 mmol of amine, 1.0 eq), glucono-delta-lactone (0.1781 g, 0.9998 mmol, 3.999 eq), TEA (0.0761 g, 0.7525 mmol, 3.010 eq) and a stir bar were placed in a 20 mL scintillation vial filled with 10 mL of methanol. The vial was heated and stirred at 40 °C for 48 hours under nitrogen. The reaction was then precipitated directly into 100 mL of cold diethyl ether. The solution was filtered using a fine fritted funnel and dried under vacuum overnight. After seeing impurities in the ^{1}H NMR, the sample was redissolved in water and dialyzed against water for 3 days The sample was lyophilized to yield a white powder (0.1834 g, 36.80%). d = 7.46 (bs, 1H), 4.30 (s, 1H), 4.13 (s, 1H), 3.83 (s, 2H), 3.64 (brs, 114H), 1.25 (s, 1H).

3.3. Synthesis of (2R,3S,4R,5R)-2,3,4,5,6-pentahydroxy-N-(2-methoxyethyl)hexanamide (Cl-ana)

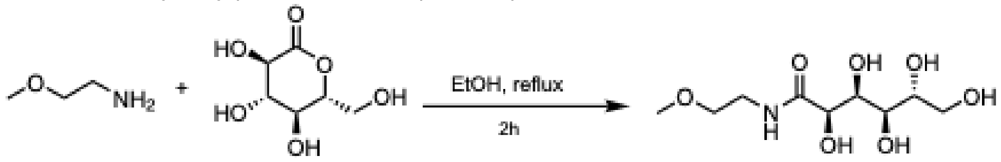

In a procedure adapted from Kang, and Kalow,[12] glucono-delta-lactone (1 eq, 1.00 g, 5.6 mmol) was placed in a 100 mL round bottom flask. 2-Methoxyethylamine (1.1eq, 0.46 g, 6.2 mmol) was added dropwise to the flask. A condenser was attached to the round bottom flask and the ethanol was refluxed for 2 hours. Ethanol was then removed under reduced pressure and the residual white powder was washed with 10 mL of DCM three times. The white powder was then placed in a scintillation vial and dried overnight to yield the final product. The NMR was prepared in DMSO-d6 f(0.52 g, 36 %). ^{1}H-NMR d = 7.58 (t, 1H), 5.41 (d, 1H), 4.53 (brs, 1H), 4.47 (brs, 1H), 4.40 (d, 1H), 4.33 (t, 1H), 4.00(t, 1H), 3.90 (brs, 1H), 3.58 (brs, 1H), 3.47 (brs, 2H), 3.35 (m, 4H), 3.30 (t, 1H), 3.25 (s, 3H).

3.4. *Synthesis of 5 kDa 4PEG-3-fluorophenylboronic acid*

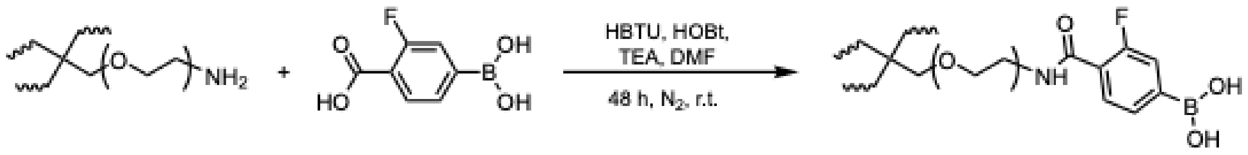

In a procedure adapted from Xiang et. al.,[13] 5kDa 4PEG-amine (2.0 g, 0.4 mmol, 1.0 eq), HOBt (0.2450, 3.2 mmol, 8 eq), 4-carboxy-3-flurophenylboronic acid (0.5886 g, 3.2 mmol, 8 eq) and TEA (0.446 mL, 3.200 mmol, 8.0 eq) were placed in a 100 mL flame dried round bottom flask and dissolved in 20 mL of DMF. The reaction mixture was then stirred for 5 minutes. Following this HBTU (1.2136 g, 3.2 mmol, 8 eq) was added. The reaction was stirred at room temperature for 48 hours under nitrogen. The reaction mixture was then dialyzed against MeOH for 1 day followed by dialysis against DI water for 2 days. The sample was lyophilized to yield a white powder. When the sample was prepared in DMSO-d6 gelation occurred, likely due to formation of a borazine crosslink under dry conditions. One drop of DI water was added therefore added to analyze by NMR. (1.7478 g, 77.15 %). ^{1}H-NMR d = 8.39 (s, 2H), 8.27(brt, 1H), 7.53 – 7.26 (m, 4H), 3.64 (brs, 114H) ^{1}F-NMR d = 116.1 (s, 1F).

3.5. *Synthesis of 2 kDa mPEG-3-fluorophenylboronic acid*

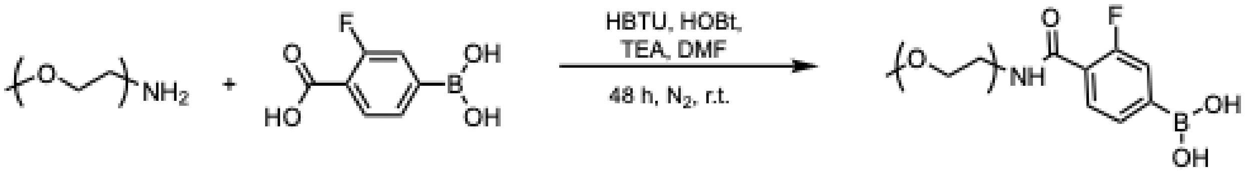

In a procedure adapted from Xiang et. al.,[13] 5kDa 4PEG-amine (2.0 g, 0.4 mmol, 1.0 eq), HOBt (0.2450, 1.6 mmol, 8 eq), 4-carboxy-3-flurophenylboronic acid (0.5886 g, 3.2 mmol, 8 eq) and TEA (0.446 mL, 3.200 mmol, 8.0 eq) were placed in a 100 mL flame dried round bottom flask and dissolved in 20 mL of DMF. The reaction mixture was then stirred for 5 minutes. Following this HBTU (1.2136 g, 3.2 mmol, 8 eq) was added. The reaction was stirred at room temperature for 48 hours under nitrogen. The reaction mixture was then dialyzed against MeOH for 1 day followed by dialysis against DI water for 2 days. The sample was lyophilized to yield a white powder (1.7478 g, 77.15 %). ^{1}H-NMR d = 8.39 (s, 2H), 8.27(brt, 1H), 7.53 – 7.26 (m, 4H), 3.64 (brs, 181H) ^{1}F-NMR d = 116.1 (s, 1F)

3.6. Synthesis of 5 kDa 4PEG-benzyl aldehyde (5kDa 4PEG-Ar-CHO):

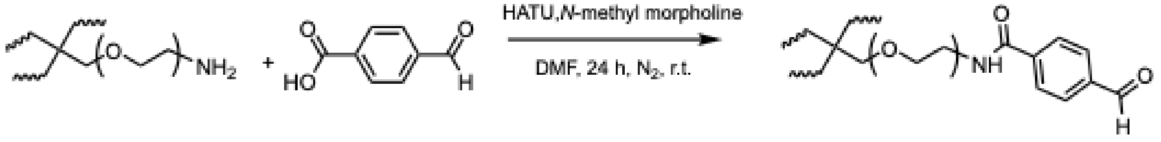

The benzyl PEG aldehyde was synthesized following the reported procedure.[14] 4-formylbenzoic acid (264 mg, 1.76 mmol, 2.20 eq. to NH_2 groups) was activated with HATU (1-[Bis(dimetylamino)methylene]-1H-1,2,3-triazolo[4,5-b]pyridinium 3-oxid hexafluorophosphate) (608 mg, 1.60 mmol, 2.00 eq. to NH_2 groups) and 4-methylmorpholine (0.440 mL, 4.00 mmol, 5.00 eq. to NH_2 groups) in dimethylformamide (DMF) 7.00 mL under N_2 for 10 min. In a separate vessel, 4-methylmorpholine (0.440 mL, 4.00 mmol, 5.00 eq. to NH_2 groups was added to 4-arm PEG-NH_2, HCl salt (M_n 5000 g/mol) (1.00 g, 0.200 mmol, 1.00 eq.) in DMF 3.00 mL. The 4-arm PEG-NH_2, HCl salt containing 4-methylmorpholine was slowly added to the first solution under N_2. The reaction was allowed to proceed at room temperature for 24 h. The reaction mixture was concentrated and precipitated in cold diethyl ether (-25 °C). The crude product was concentrated under reduced pressure, dissolved in DI water, transferred to regenerated cellulose membranes (Spectra/Por) with a molecular weight cut-off of 2,000 g/mol, and dialyzed against DI water for 2 days. The product was lyophilized to give an off-white powder (83 % yield, Figure S6). ^{1}H NMR (400 MHz, $CDCl_3$): δ= 10.1 (s, H, C*H*O), 8.12-7.88 (m, 4H, C_6H_4-), 3.83-3.41 (m, 113.5H, -O-CH_2-CH_2-O-).

3.7. Synthesis of 2 kDa mPEG-benzyl aldehyde (2kDa mPEG-Ar-CHO):

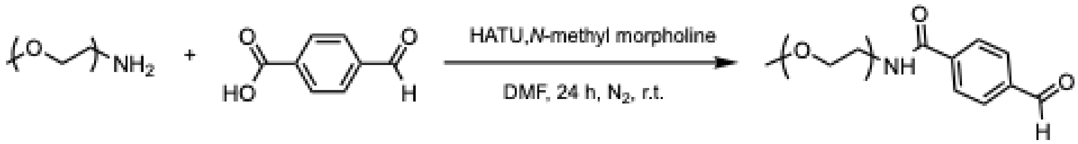

The (mono) functionalization of PEG with an aryl aldehyde was performed following the reported procedure.[14] 4-formylbenzoic acid (430 mg, 1.10 mmol, 2.20 eq. to NH_2 groups) was activated with HATU (1-[Bis(dimetylamino)methylene]-1H-1,2,3-triazolo[4,5-b]pyridinium 3-oxid hexafluorophosphate) (380 mg, 1.00 mmol, 2.00 eq. to NH_2 groups) and 4-methylmorpholine (0.270 mL, 2.50 mmol, 5.00 eq. to NH_2 groups) in dimethylformamide (DMF) 7.00 mL under N_2 for 10 min. In a separate vessel, 4-methylmorpholine (0.270 mL, 2.50 mmol, 5.00 eq. to NH_2 groups was added to poly(ethylene glycol) methyl ether amine (mPEG-NH_2) (M_n 2000 g/mol) (1.00 g, 0.500 mmol, 1.00 eq.) in DMF 5.00 mL. The mPEG-NH_2 containing 4-methylmorpholine was slowly added to the first solution under N_2. The reaction was allowed to proceed at room temperature for 24 h. The reaction mixture was concentrated and precipitated in cold diethyl ether (-25 °C). The crude product was concentrated under reduced pressure, dissolved in DI water, transferred to regenerated cellulose membranes (Spectra/Por) with a molecular weight cut-off of 2,000 g/mol, and dialyzed against DI water for 2 days. The product was lyophilized to give an off-white powder (75 % yield, Figure S7). ^{1}H NMR (400 MHz, $CDCl_3$): δ= 10.1 (s, H, C*H*O), 8.12-7.88 (m, 4H, C_6H_4-), 3.83-3.41 (m, 181H, -O-CH_2-CH_2-O-)

3.8. Synthesis of 5 kDa 4PEG-hydrazine (5kDa 4PEG-Hz):

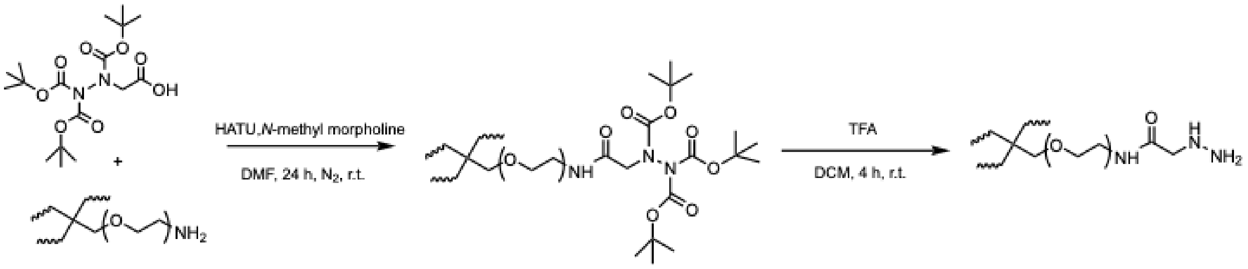

5 kDa 4PEG hydrazine was synthesized following the reported procedure.[15] Tri-boc-hydrazinoacetic acid (1.37 g, 3.52 mmol, 2.20 eq. to NH_2 groups) was activated with 4-methylmorpholine (0.880 mL, 8.00 mmol, 5.00 eq. to NH_2 groups) and HATU (1.22 g, 3.20 mmol, 2.00 eq. to NH_2 groups) in DMF 10.0 mL. In a separate vessel, 4-methylmorpholine (0.88 mL, 8.00 mmol, 5.00 eq. to NH_2 groups was added to 4-arm PEG-NH_2, HCl salt (M_n 5000 g/mol) (2.00 g, 0.400 mmol, 1.00 eq.) in DMF 5.00 mL. The 4-arm PEG-NH_2, HCl salt containing 4-methylmorpholine was slowly added to the first solution under N_2. The reaction was allowed to proceed at room temperature (25 °C) for 24 h. The reaction mixture was concentrated and precipitated in cold diethyl ether (-25 °C), filtered and dried to obtain 5k4APEG-NBoc-NBoc2 (Figure S8). ^{1}H NMR (400 MHz, $CDCl_3$): δ = 3.83-3.41 (m, 113.5H, -O-CH_2-CH_2-O-), 1.55-1.50 (d, 18H, -OC(CH_3)$_3$), δ = 1.48-1.42 (d, 9H, -OC(CH_3)$_3$).

After that, the 5k4PEG-NBoc-NBoc2 was dissolved in a 50:50 mixture of trifluoroacetic acid (TFA) and DCM. The reaction proceeded for 4 h and then precipitated into cold diethyl ether. The crude product was filtered and concentrated under reduced pressure, dissolved in DI water, transferred to regenerated cellulose membranes (Spectra/Por) with a molecular weight cut-off of 2,000 g/mol, and dialyzed against DI water for 1 day. The product was lyophilized to give a yellow powder (42 % yield, Figure S9). ^{1}H NMR (400 MHz, $CDCl_3$): δ= 3.83-3.41 (m, 113.5H, -O-CH_2-CH_2-O-).

3.9. *Synthesis of 2 kDa mPEG-hydrazine (2kDa mPEG-Hz):*

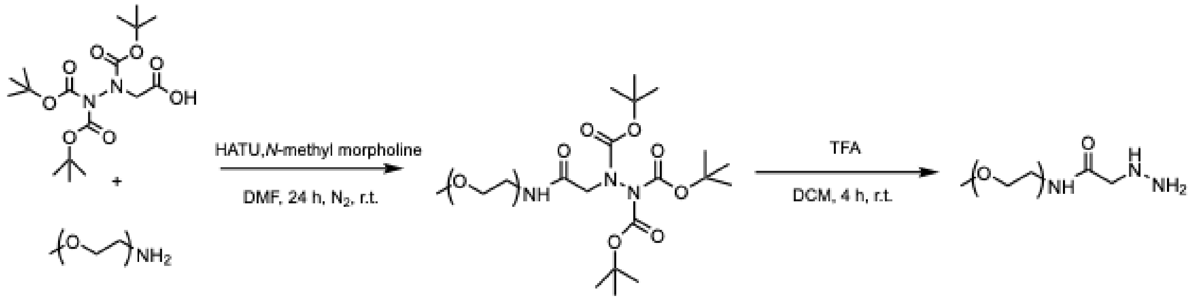

m-PEG hydrazine was synthesized following the reported procedure.[15] Tri-boc-hydrazinoacetic acid (430 mg, 1.10 mmol, 2.20 eq. to NH_2 groups) was activated with 4-methylmorpholine (0.270 mL, 2.50 mmol, 5.00 eq. to NH_2 groups) and HATU (380 mg, 1.0 mmol, 2.00 eq. to NH_2 groups) in DMF 10.0 mL. In a separate vessel, 4-methylmorpholine (0.270 mL, 2.50 mmol, 5.00 eq. to NH_2 groups was added to mPEG-NH_2 (M_n 2000 g/mol) (1.00 g, 0.500 mmol, 1.00 eq.) in DMF 5.00 mL. The mPEG-NH_2 containing 4-methylmorpholine was slowly added to the first solution under N2. The reaction was allowed to proceed at room temperature (25 °C) for 24 h. The reaction mixture was concentrated and precipitated in cold diethyl ether (-25 °C), filtered and dried to obtain 5k4PEG-NBoc-NBoc2 (Figure S10). ^{1}H NMR (400 MHz, $CDCl_3$): δ = 3.84-3.37 (m, 181H, -O-CH_2-CH_2-O-), 1.57-1.48 (d, 18H, -OC(CH_3)$_3$), δ = 1.48-1.41 (d, 9H, -OC(CH_3)$_3$).

After that, the 2 kDa mPEG-NBoc-NBoc2 was then dissolved in a 50:50 mixture of trifluoroacetic acid (TFA) and DCM. The reaction was allowed to proceed for 4 h and then precipitated into cold diethyl ether again. The crude product was filtered and concentrated under reduced pressure, dissolved in DI water, transferred to regenerated cellulose membranes (Spectra/Por) with a molecular weight cut-off of 2,000 g/mol, and dialyzed against DI water for 1 day. The product was lyophilized to give a yellow powder (22 % yield, Figure S11). ^{1}H NMR (400 MHz, $CDCl_3$): δ= 3.84-3.37 (m, 181H, -O-CH_2-CH_2-O-).

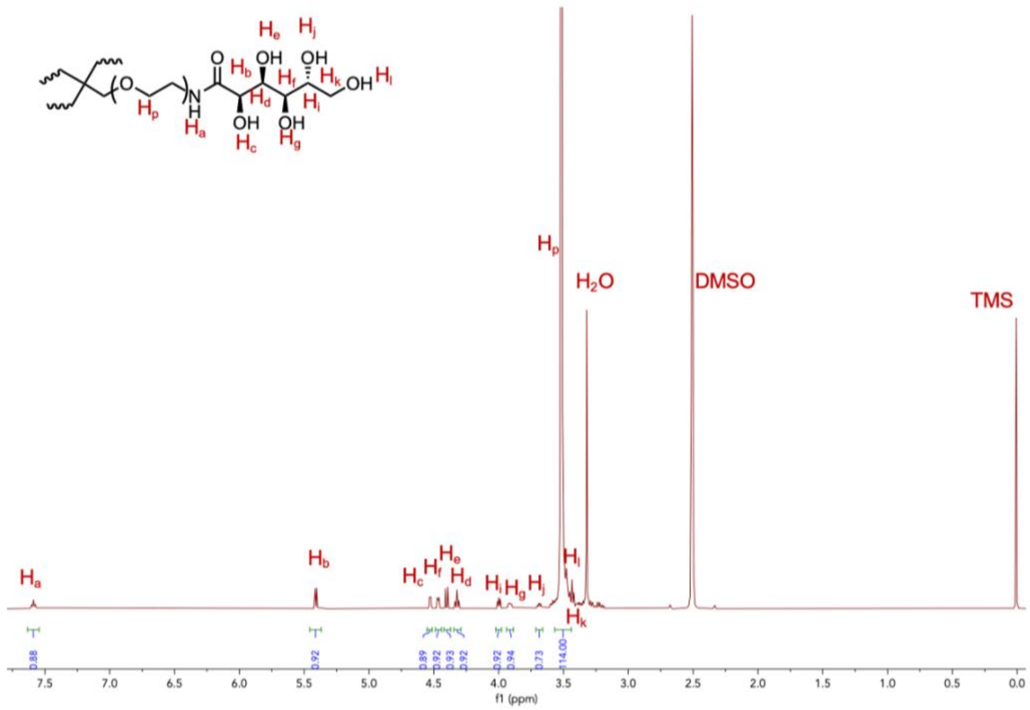

Figure S3. 1^{1}H NMR of 5 kDa 4PEG-GA (400 MHz, DMSO-d6, 25 °C).

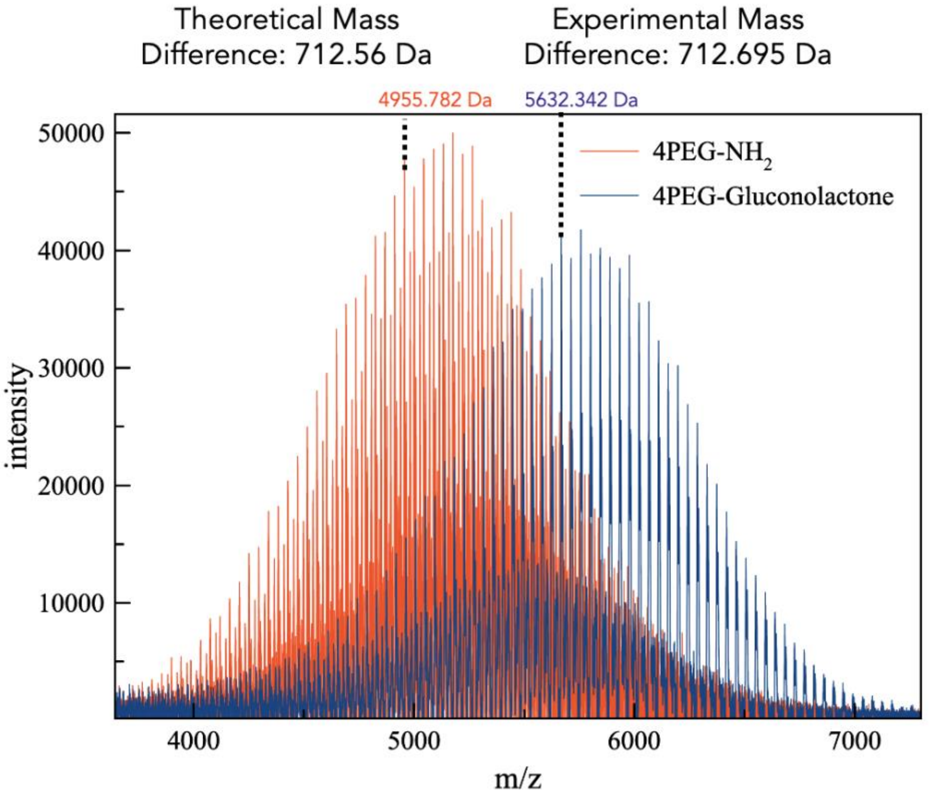

Figure S4. MALDI-TOF Analysis of 5 kDa 4PEG-GA.

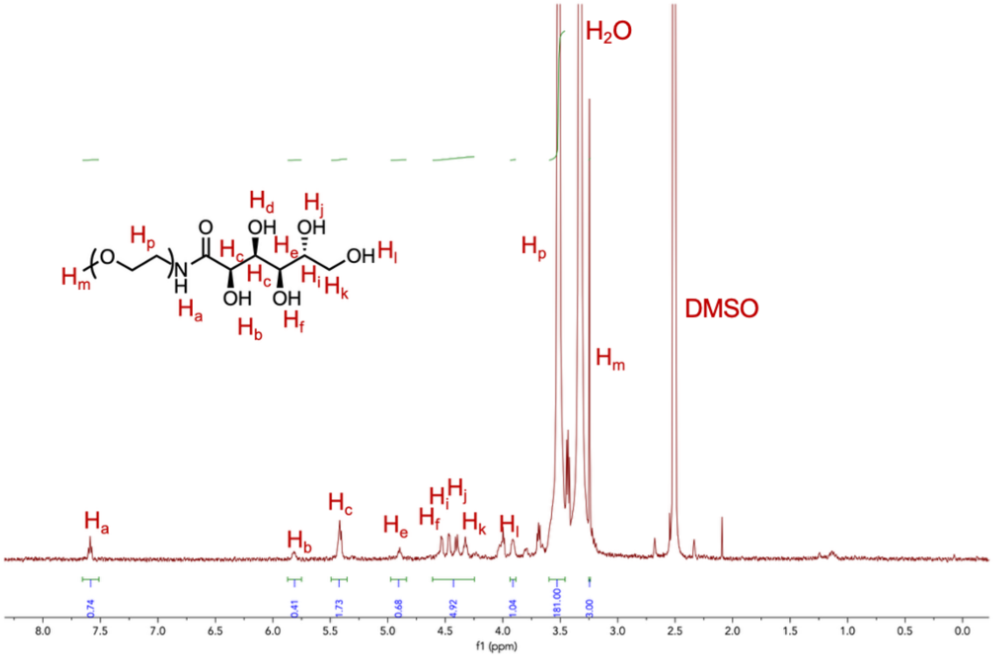

Figure S5. ^{1}H NMR of 2 kDa mPEG-GA (400 MHz, DMSO-d6, 25 °C).

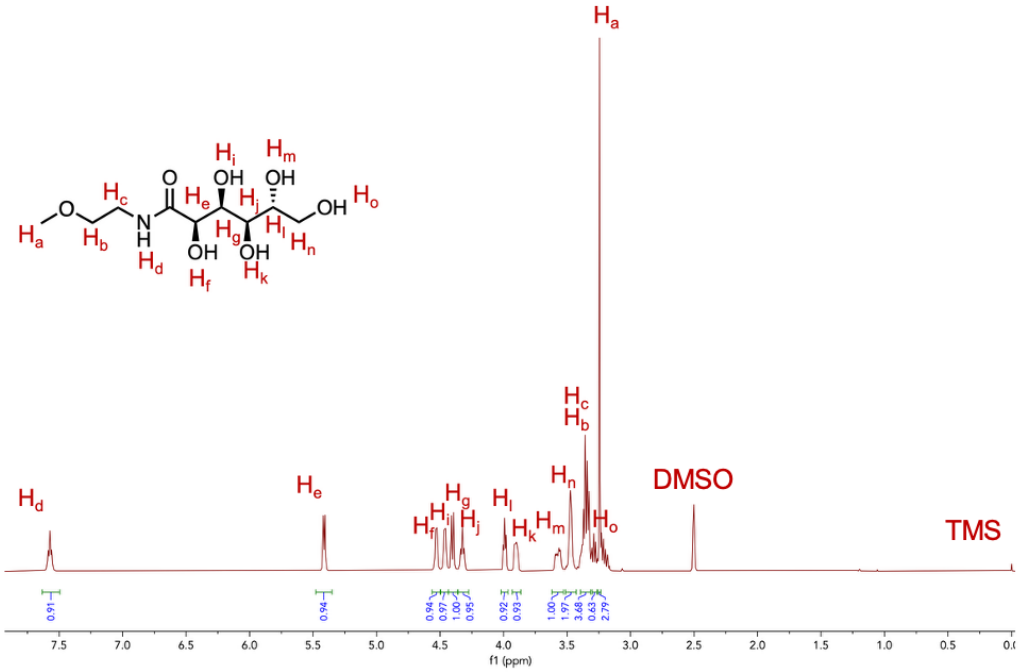

Figure S6. ^{1}H NMR of (2R,3S,4R,5R)-2,3,4,5,6-pentahydroxy-N-(2-methoxyethyl)hexanamide (CI-ana) (400 MHz, DMSO-d6, 25 °C).

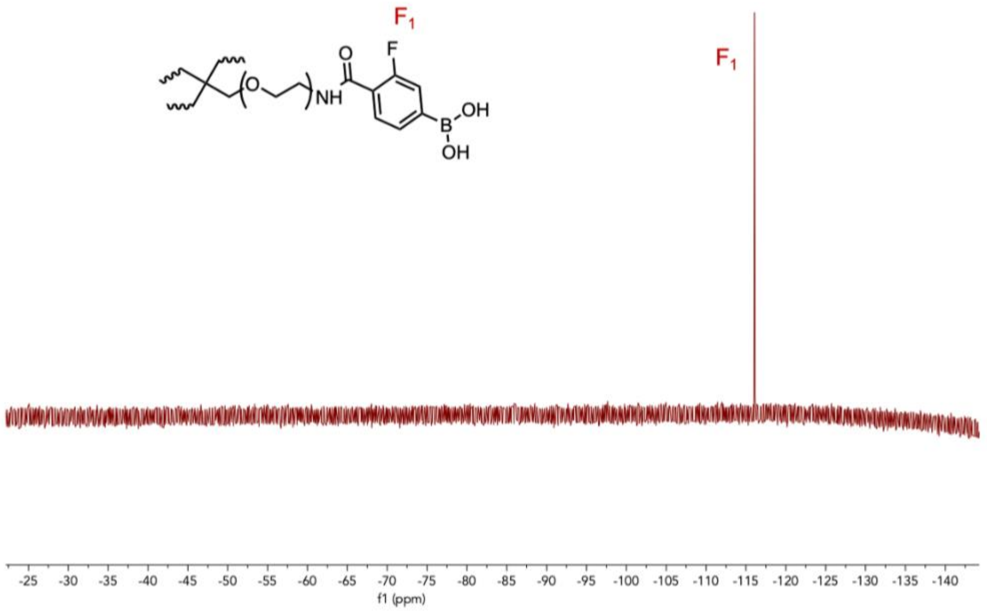

Figure S7. ^{19}F NMR of 5 kDa 4PEG-FPBA (400 MHz, DMSO-d6, 25 °C).

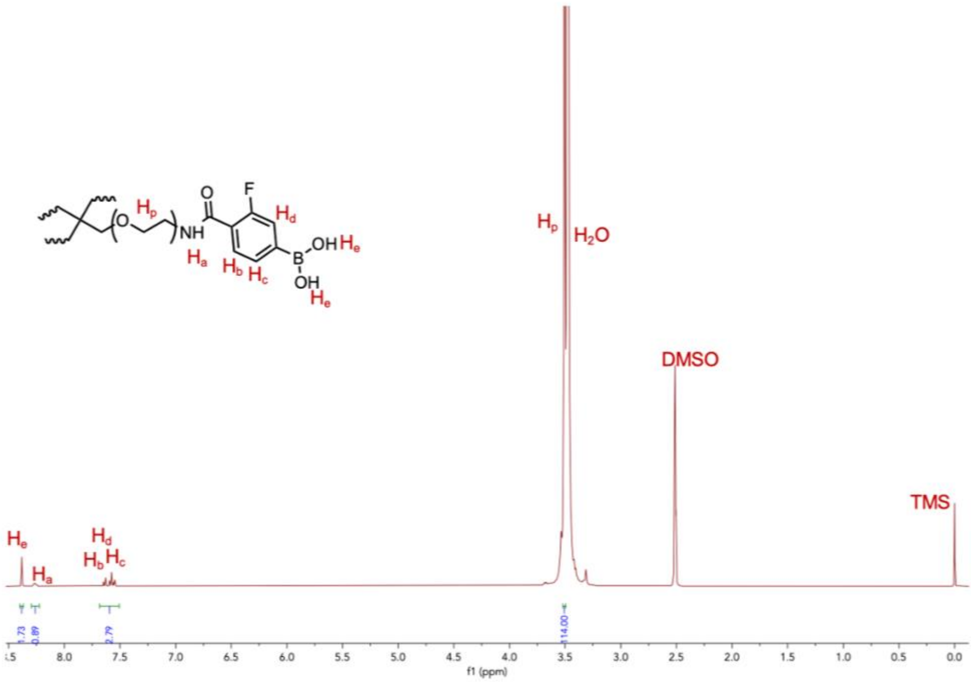

Figure S8. ^{1}H NMR of 5 kDa 4PEG-FPBA (400 MHz, DMSO-d6, 25 °C).

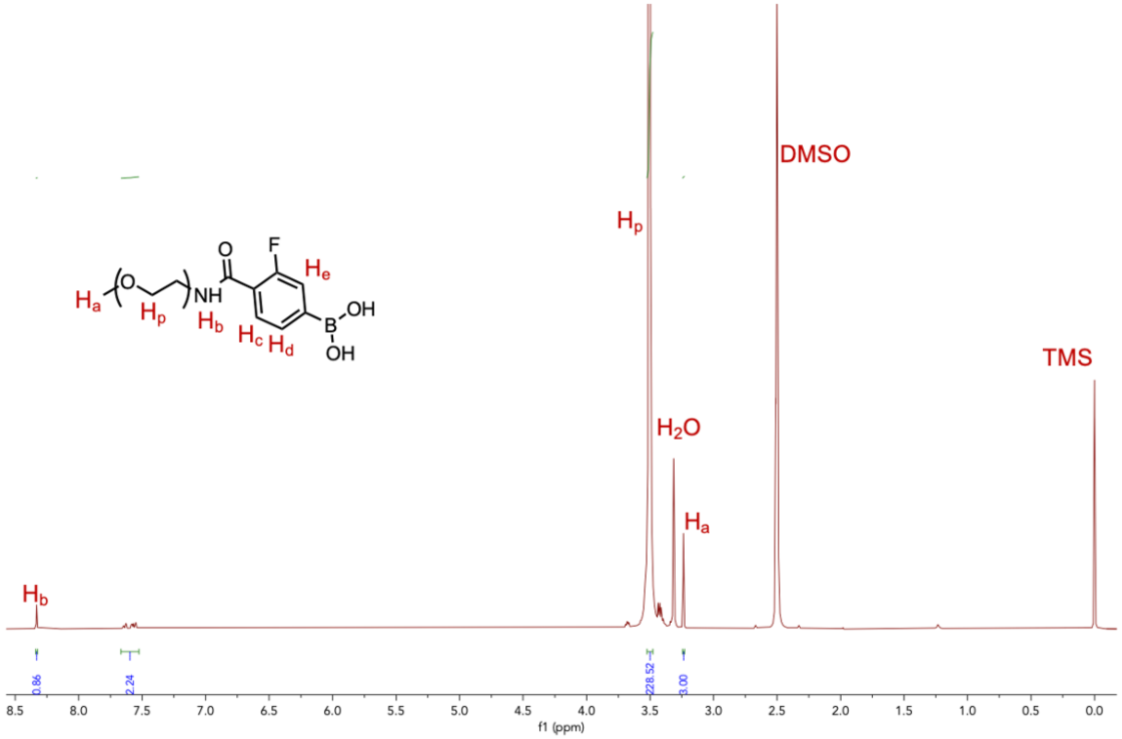

Figure S9. 1D ^{1}H NMR of 2 kDa mPEG-FPBA (400 MHz, DMSO-d6, 25 °C).

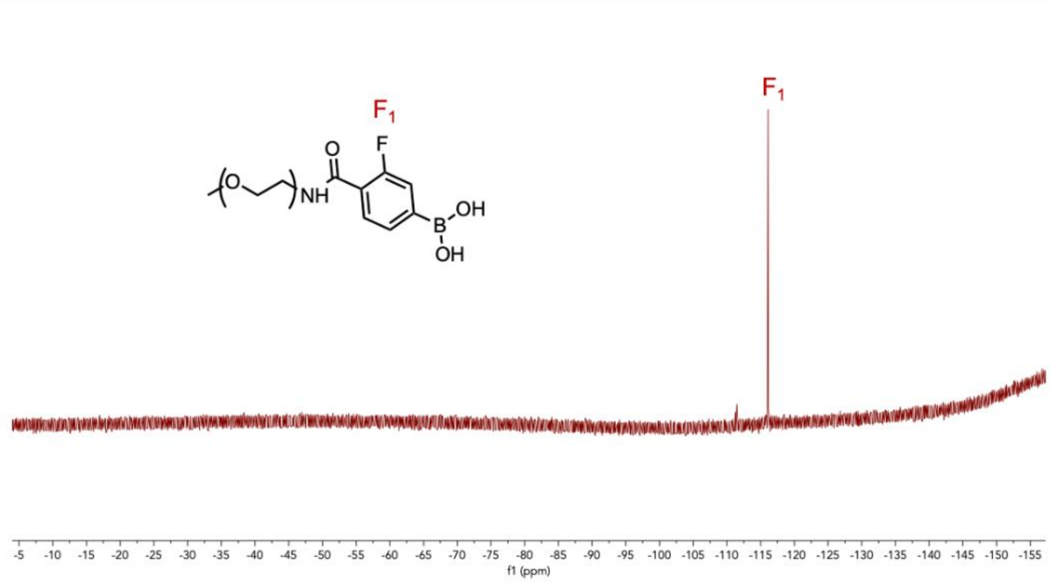

Figure S10. ^{1}H NMR of 2 kDa mPEG-FPBA (400 MHz, DMSO-d6, 25 °C).

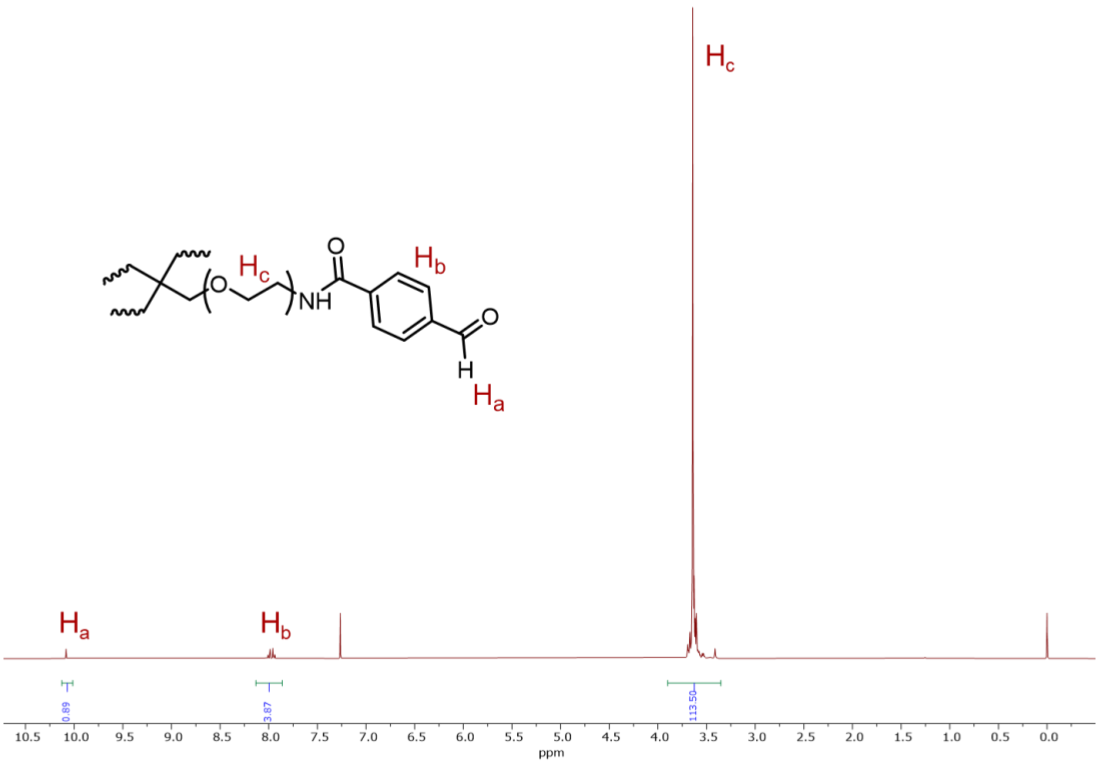

Figure S11. ^{1}H NMR spectrum of 5kDa 4PEG-Ar-CHO (400 MHz, $CDCl_3$, 25 °*C*).

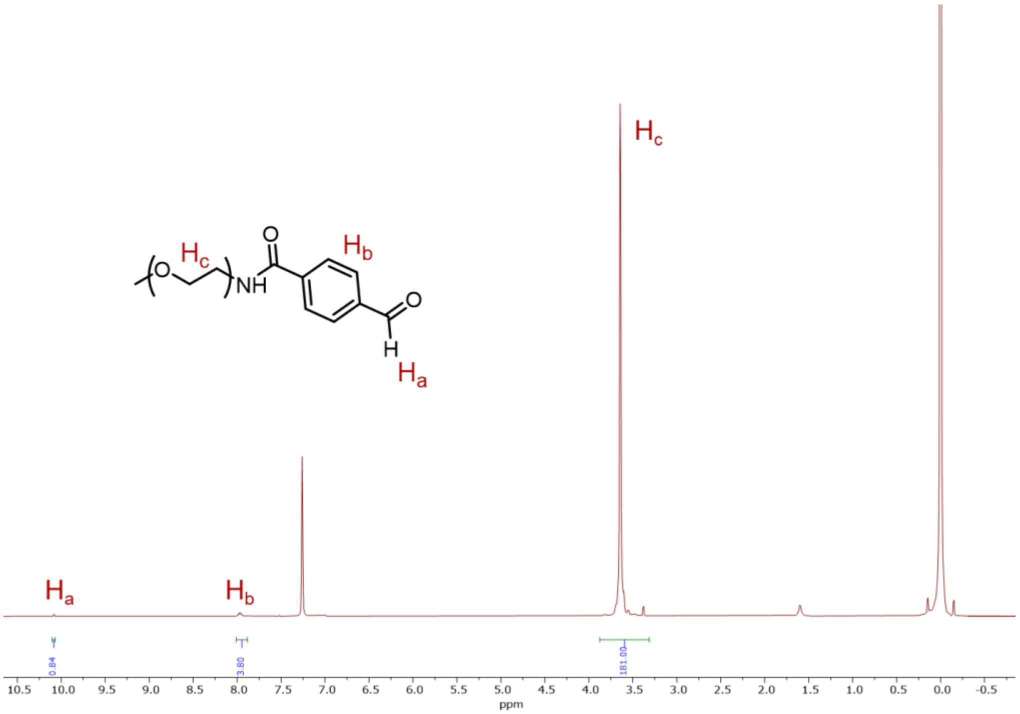

Figure S12. ^{1}H NMR spectrum of 2kDa mPEG-Ar-CHO (400 MHz, $CDCl_3$, 25 °C).

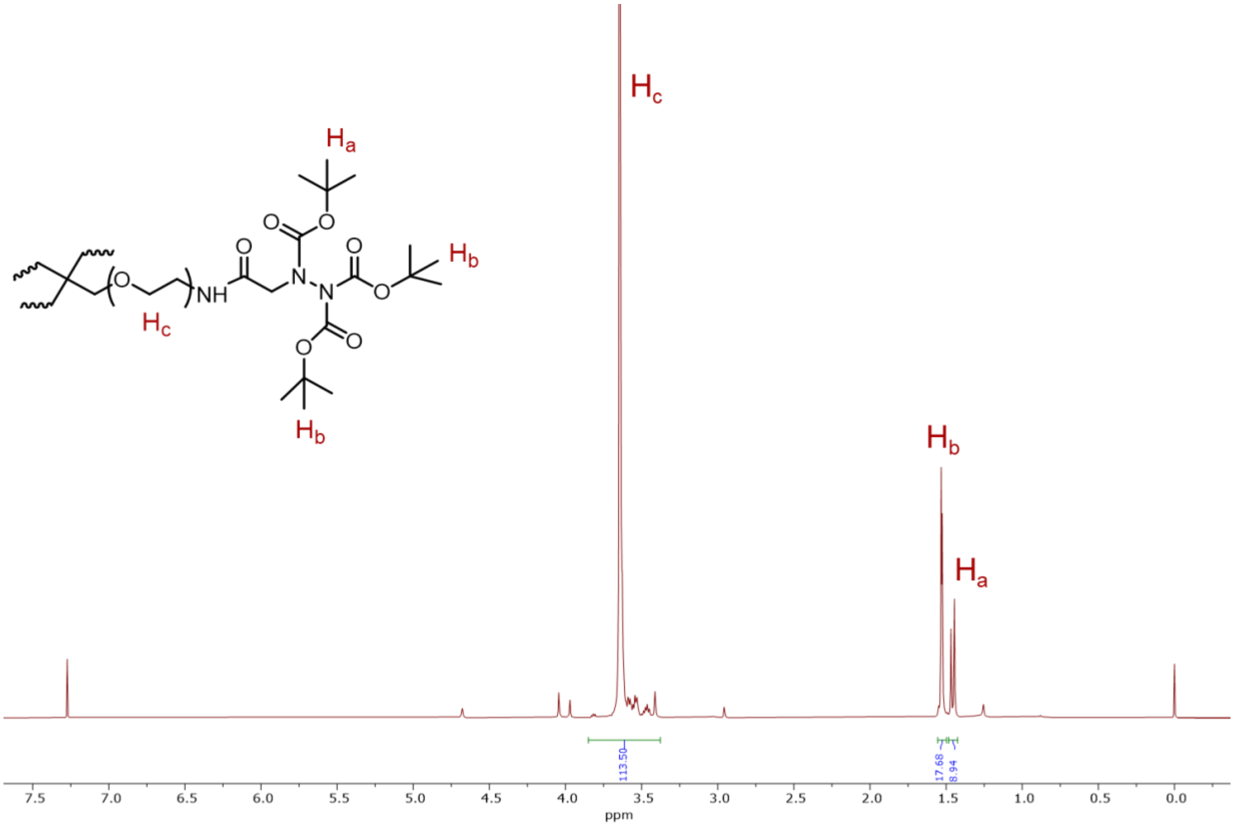

Figure S13. ^{1}H NMR spectrum of 5kDa 4PEG-NBoc-NBoc$_2$ (400 MHz, $CDCl_3$, 25 °C).

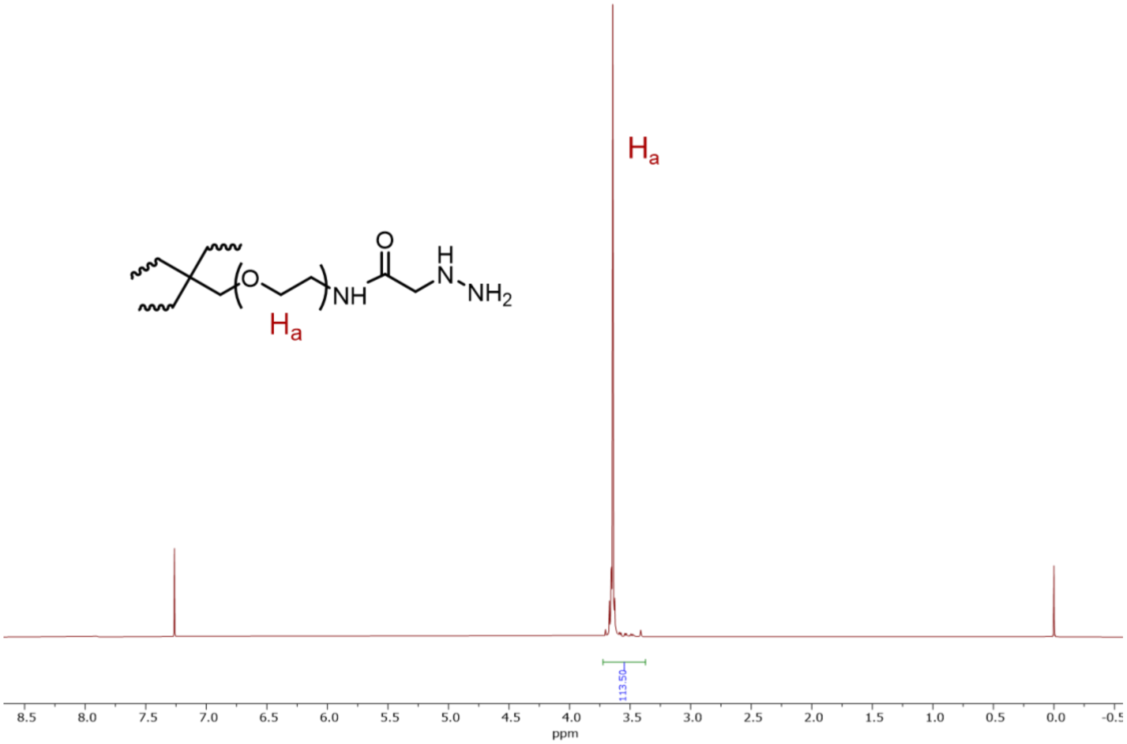

Figure S14. ^{1}H NMR spectrum of 5kDa 4PEG-Hz (400 MHz, $CDCl_3$, 25 °C).

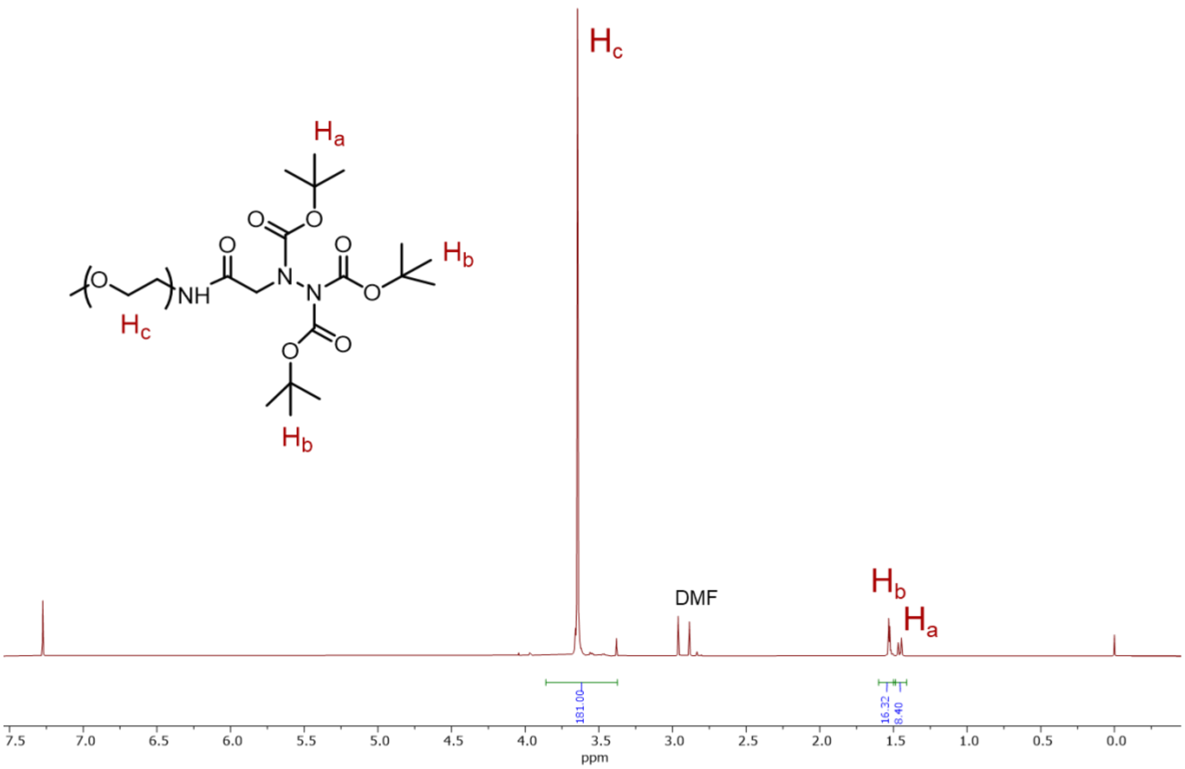

Figure S15. ^{1}H NMR spectrum of 2kDa mPEG-NBoc-NBoc$_2$ (400 MHz, $CDCl_3$, 25 °C).

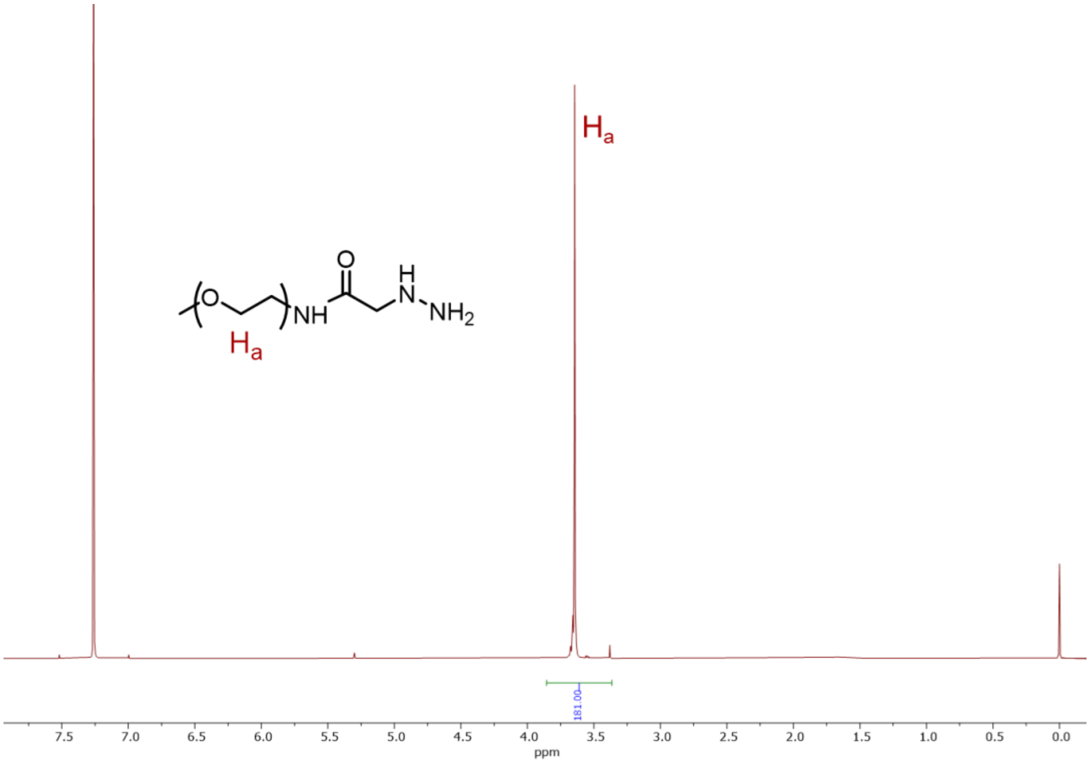

Figure S16. ^{1}H NMR spectrum of 2kDa mPEG-Hz (400 MHz, $CDCl_3$, 25 °C).

4. ITC

4.1. ITC Procedures

For the boronate ester system, ITC experiments were conducted at 25 °C with 30 injections. The syringe contained 27.5 mM mPEG–phenylboronic acid, while the cell was filled with 4 mM of a diol species (either a competitor or the mPEG-based crosslinker). All association constants were determined through the kinITC software.[16]

The heats of injection were plotted as a binding isotherm to determine the K_a values. The k_{off} values were determined by extracting the equilibration time between each injection and fitting these values as a function of molar ratio. When determining the k_{off} values the key assumptions that the software makes are:

(1) The instrument gives a single response time
(2) The time between each injection starting, and returning to the baseline can be extracted, plotted against the molar ratio of the interaction and fit in a parabolic curve to return the rate constants of the binding interaction.

For more details regarding the kinITC process for determining the rate constants of the interactions please refer to the corresponding publications.[16,17]

Triplicate measurements were performed for each competitor, and fit-derived residuals and are reported (K_a std, k_{off} std).

4.2. Glucose (1)

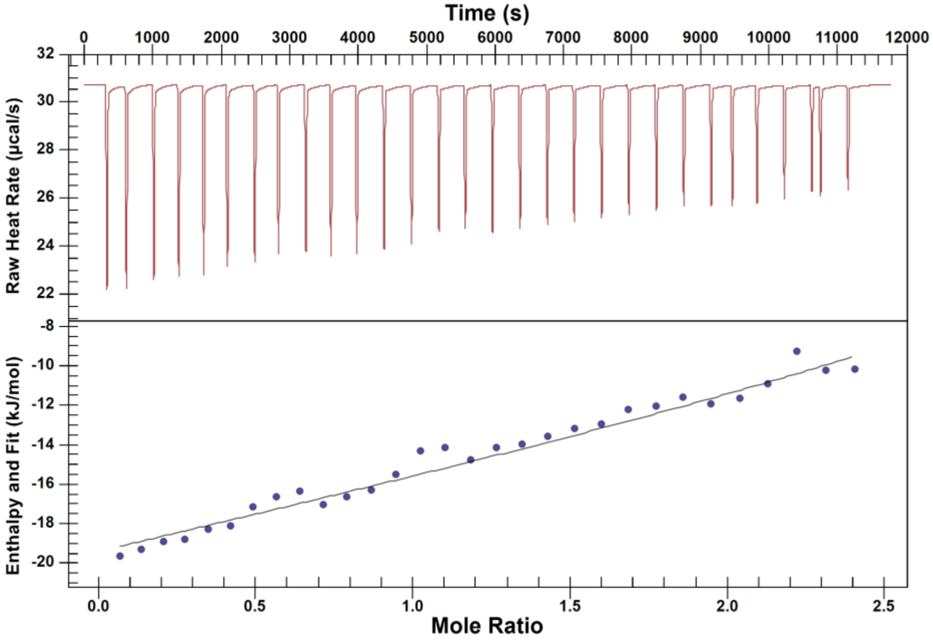

Figure S17. ITC heats of injection and binding isotherm of Glucose titrated with mPEG-FPBA Trial 1 conducted at 25 °C.

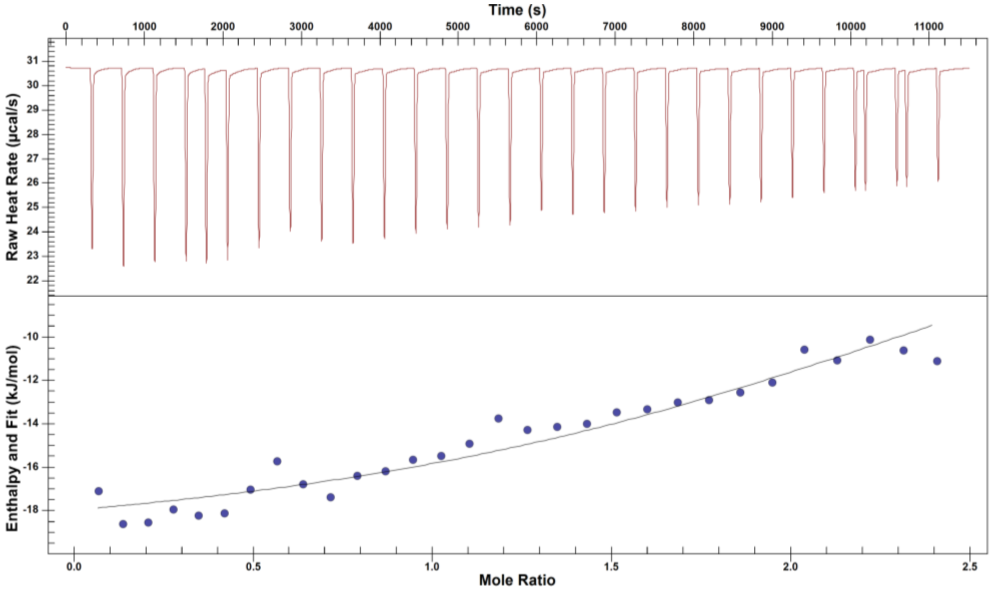

Figure S18. ITC heats of injection and binding isotherm of Glucose titrated with mPEG-FPBA Trial 2 conducted at 25 °C.

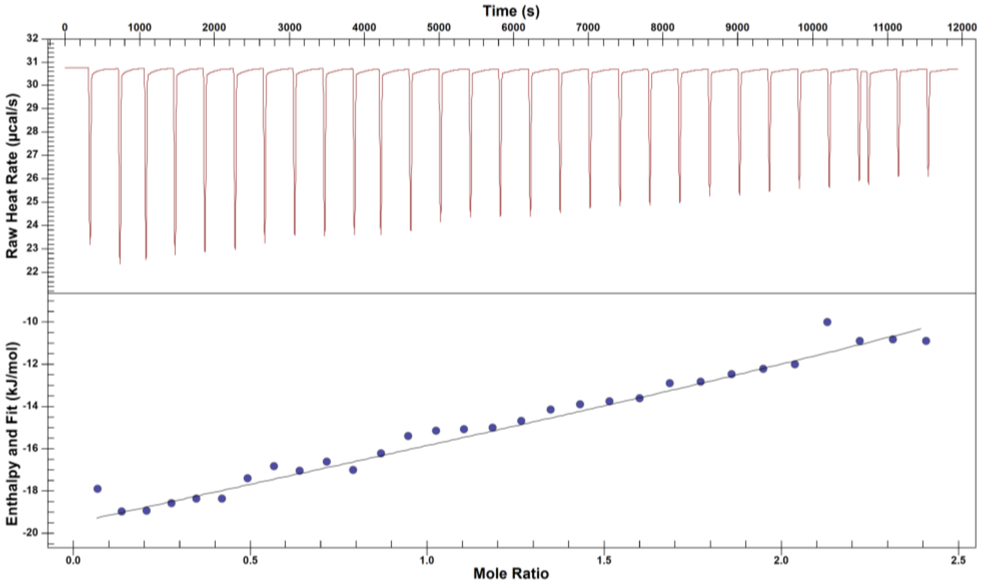

Figure S19. ITC heats of injection and binding isotherm of Glucose titrated with mPEG-FPBA Trial 3 conducted at 25 °C.

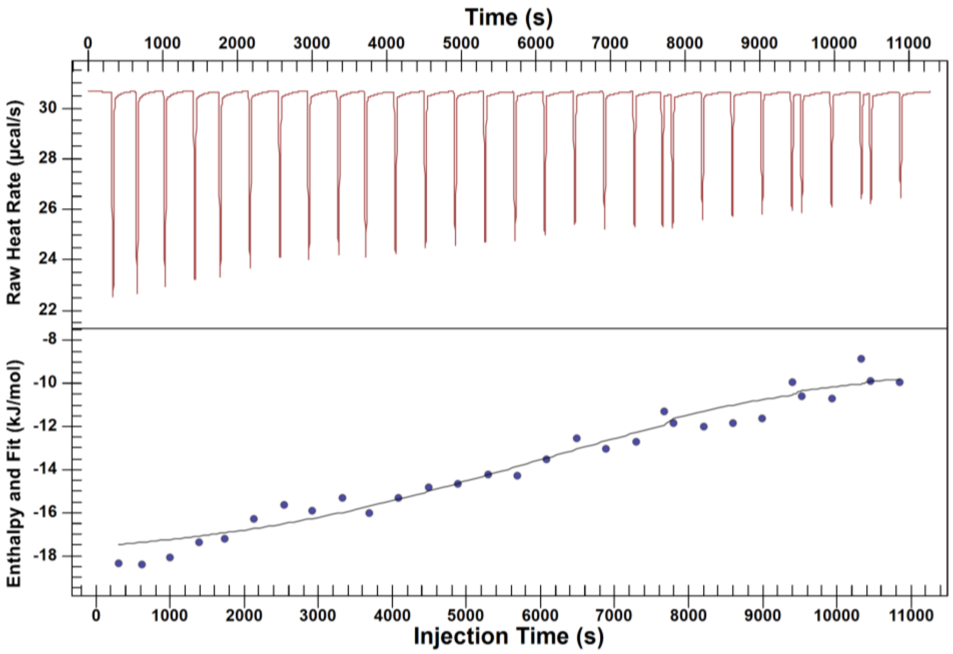

Figure S20. ITC heats of injection and binding isotherm of glucose titrated with mPEG-FPBA Trial 4 conducted at 25 °C.

Table S1. Summary of ITC fit from glucose runs determined by kinITC[3].

KinITC	**Glucose**						
Trial	n	Kd (M)	Ka (M^{-1})	Ka std	koff ($M^{-1}s^{-1}$)	koff std	kon
1	1.01E+0	1.06E-1	9.40E+0	6.57E+0	9.83E-1	1.40 E+0	9.24E+0
2	7.85E-1	1.35E-1	7.40E+0	2.07E+0	1.36E+2	2.49 E+2	1.01E+3
3	1.93E+0	3.34E-1	3.00E+0	9.59E+0	9.54E-1	1.98 E+0	2.86E+0
4	1.05E+0	1.65E-1	6.06E+0	2.35E+0	2.49E+0	2.29 E+0	1.51E+1
avg	1.19E+0	1.85E-1	6.46E+0		3.51E+1		2.59E+2
std	5.03E-1	1.02E-1	2.69E+0		6.73E+1		4.99E+2

4.3. Dyphylline (2)

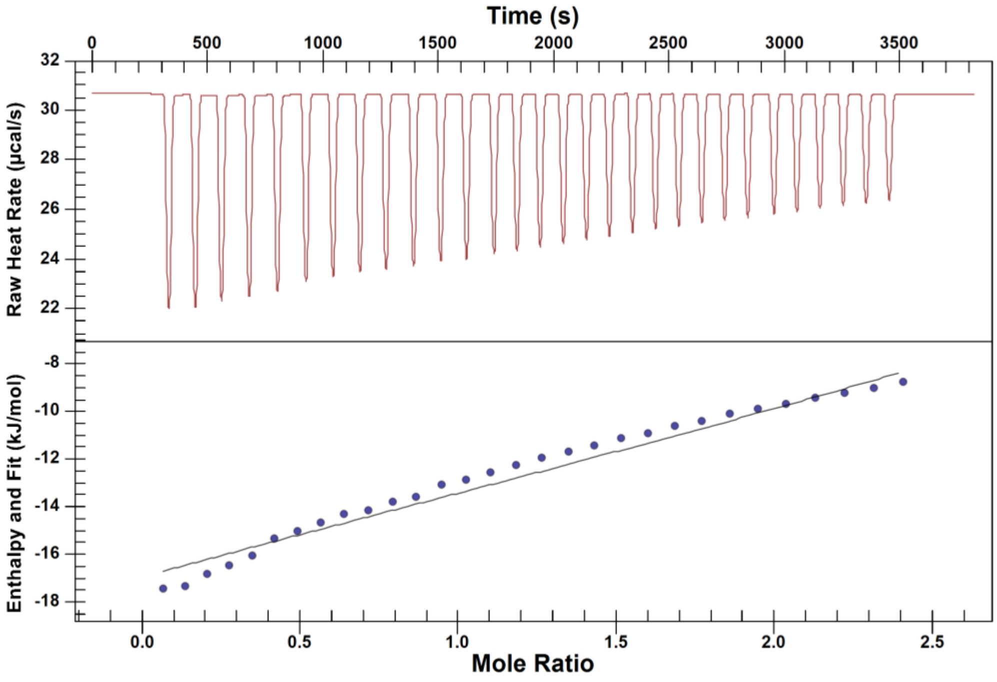

Figure S21. ITC heats of injection and binding isotherm of Dyphylline titrated with mPEG-FPBA Trial 1 conducted at 25 °C.

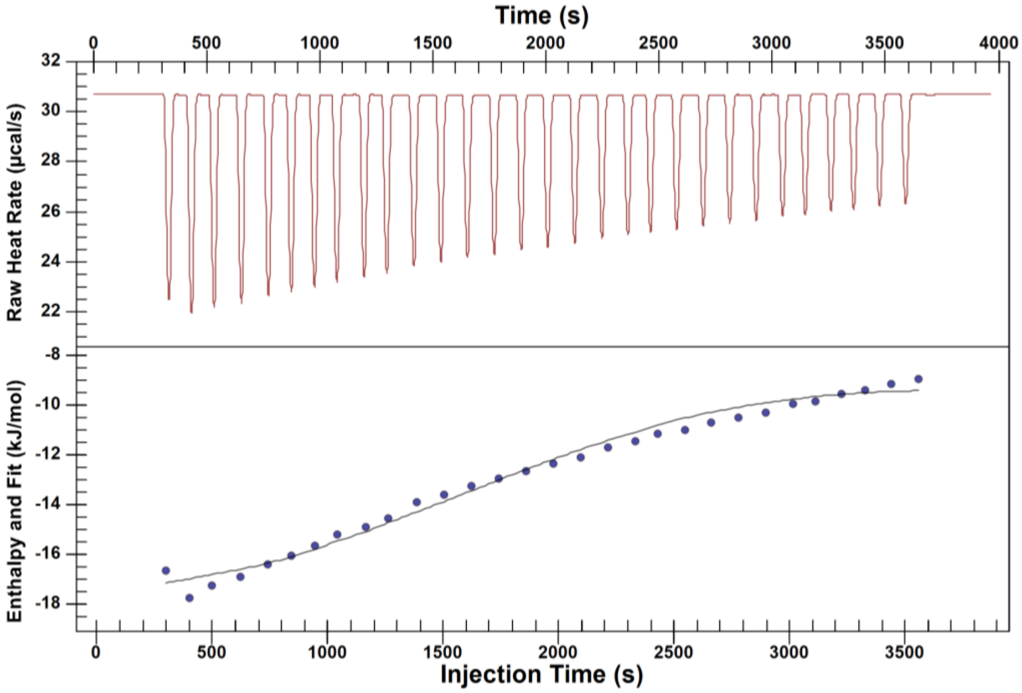

Figure S22. ITC heats of injection and binding isotherm of Dyphylline titrated with mPEG-FPBA Trial 2 conducted at 25 °C.

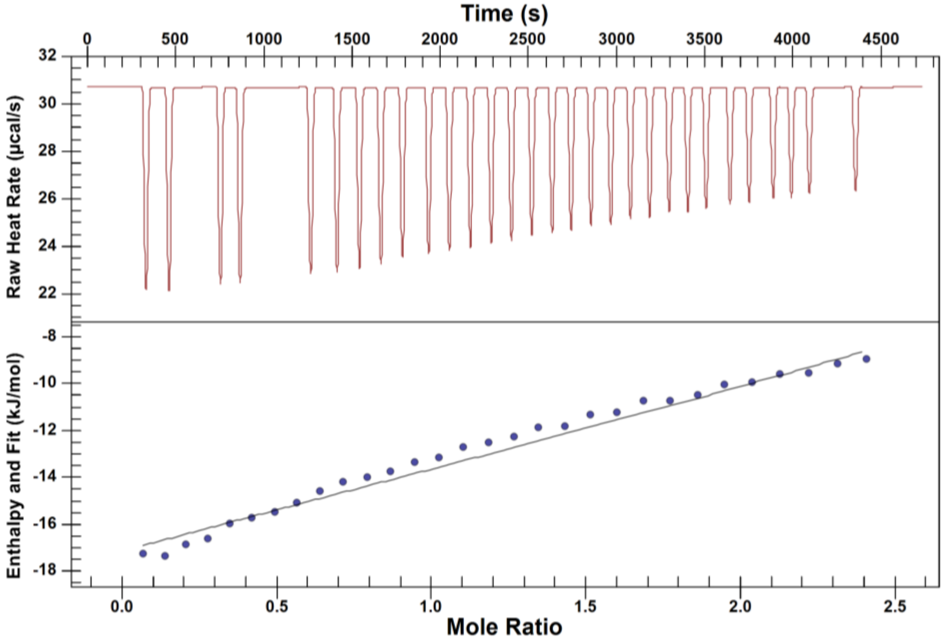

Figure S23. ITC heats of injection and binding isotherm of Dyphylline titrated with mPEG-FPBA Trial 3 conducted at 25 °C.

Table S2. Summary of ITC fits from dyphylline runs determined by kinITC[3].

KinITC	Dyphylline						
Trial	n	Kd (M)	Ka (M^{-1})	Ka std	koff ($M^{-1}s^{-1}$)	koff std	kon
1	9.87E-1	3.53E-2	2.83E+1	3.77E+0	2.69E+2	1.74E+1	7.62E+3
2	7.90E-1	3.55E-2	2.82E+1	3.38E+0	2.94E+2	1.21E+1	8.29E+3
3	1.00E+0	4.62E-2	2.16E+1	3.91E+0	2.33E+2	2.35E+1	5.03E+3
avg	9.26E-1	3.90E-2	2.60E+1		2.65E+2		6.98E+3
std	1.17E-1	6.25E-3	3.82E+0		3.09E+1		1.72E+3

4.4.   Tris (3)

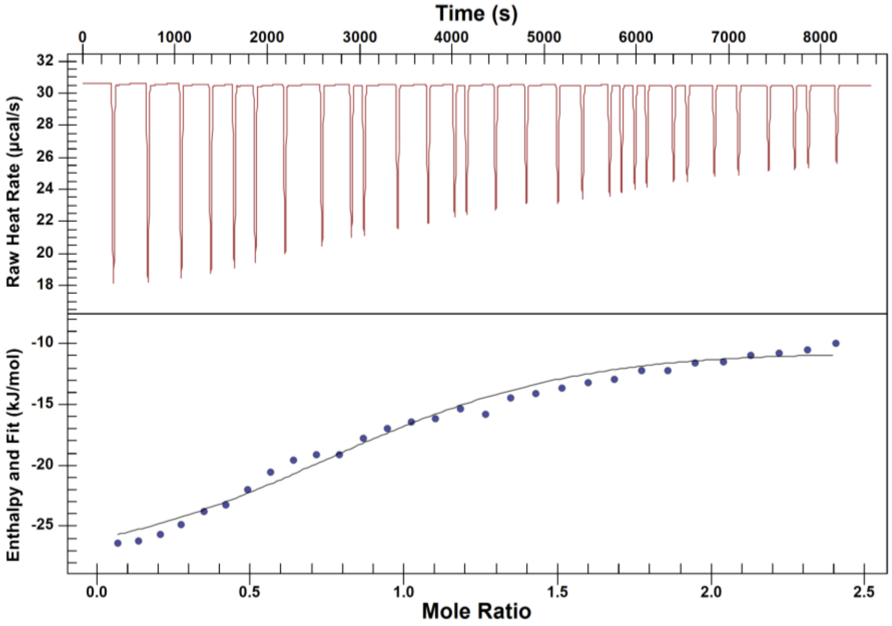

Figure S24. ITC heats of injection and binding isotherm of Tris titrated with mPEG-FPBA Trial 1 conducted at 25 °C.

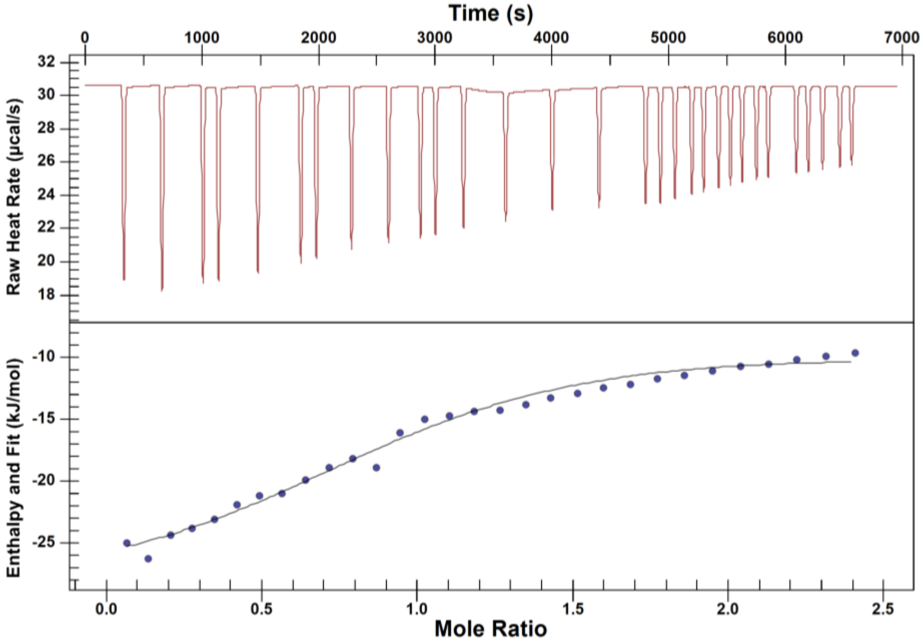

Figure S25. ITC heats of injection and binding isotherm of Tris titrated with mPEG-FPBA Trial 2 conducted at 25 °C.

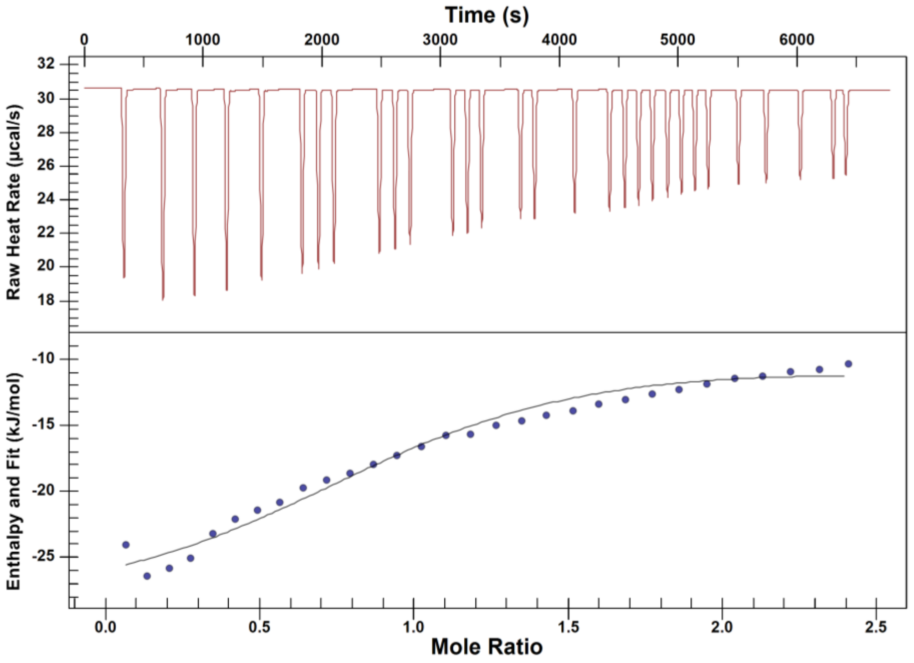

Figure S26. ITC heats of injection and binding isotherm of Tris titrated with mPEG-FPBA Trial 3 conducted at 25 °C.

Table S3. Summary of ITC fits from tris runs determined by kinITC[3].

KinITC	Tris						
Trial	n	Kd (M)	Ka (M^{-1})	Ka std	koff ($M^{-1}s^{-1}$)	koff std	kon
1	7.15E-1	2.65E-3	3.77E+2	5.04E+1	2.07E+2	2.45E+1	1.40E+5
2	9.02E-1	3.81E-3	2.63E+2	4.94E+1	1.55E+2	3.81E+1	4.07E+4
3	1.03E+0	3.18E-3	3.15E+2	3.57E+1	1.31E+2	2.77E+1	3.36E+4
avg	8.81E-1	3.21E-3	3.18E+2		1.64E+2		7.13E+4
std	1.57E-1	5.78E-4	5.72E+1		3.86E+1		5.93E+4

4.5. Capecitabine (4)

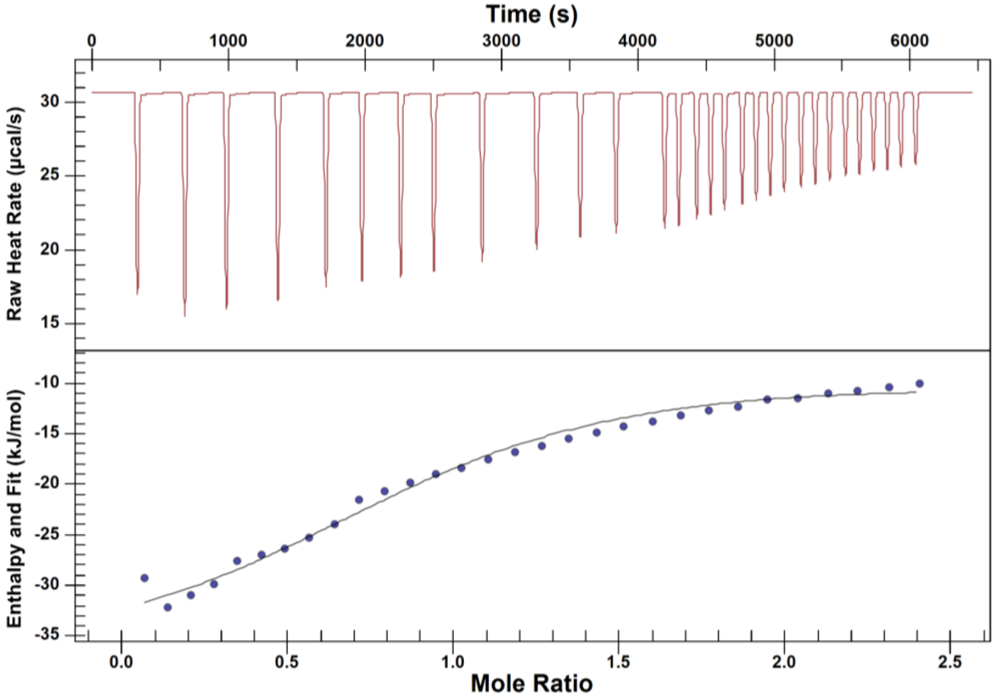

Figure S27. ITC heats of injection and binding isotherm of Capecitabine titrated with mPEG-FPBA Trial 1 conducted at 25 °C.

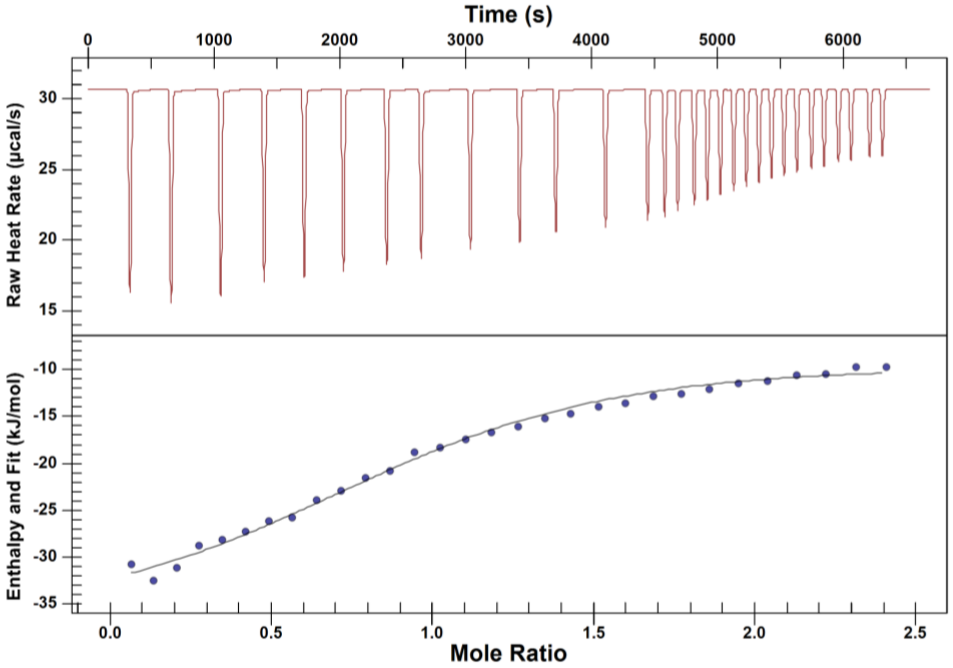

Figure S28. ITC heats of injection and binding isotherm of Capecitabine titrated with mPEG-FPBA Trial 2 conducted at 25 °C.

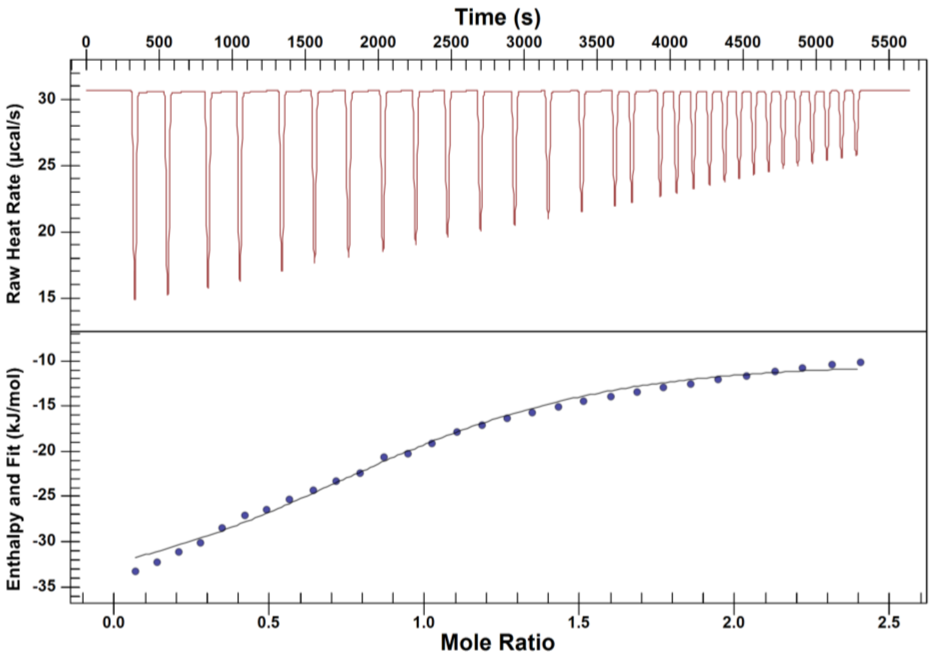

Figure S29. ITC heats of injection and binding isotherm of Capecitabine titrated with mPEG-FPBA Trial 3 conducted at 25 °C.

Table S4. Summary of ITC fits from capecitabine runs determined by kinITC[3].

KinITC	**Capecitabine**						
Trial	n	Kd (M)	Ka (M^{-1})	Ka std	koff ($M^{-1}s^{-1}$)	koff std	kon
1	1.00E+0	2.20E-3	4.55E+2	2.34E+1	1.22E+2	3.23E+1	5.53E+4
2	9.51E-1	2.15E-3	4.64E+2	1.79E+1	1.47E+2	1.76E+1	6.84E+4
3	9.79E-1	2.33E-3	4.28E+2	2.38E+1	1.27E+2	1.87E+1	5.42E+4
avg	9.77E-1	2.23E-3	4.49E+2		1.32E+2		5.93E+4
std	2.54E-2	9.43E-5	1.87E+1		1.37E+1		7.92E+3

4.6. mPEG-GA

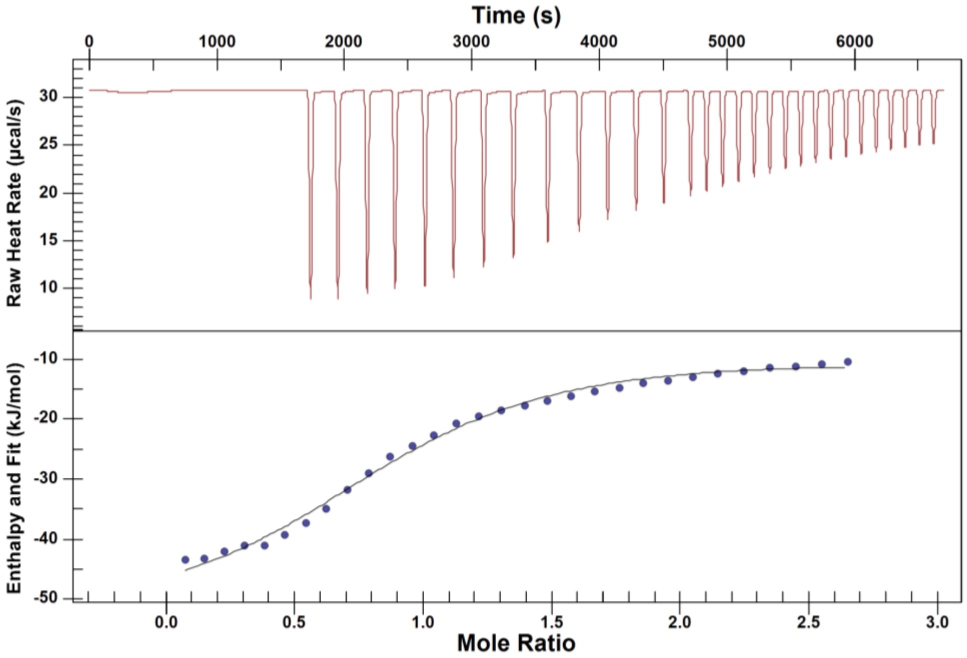

Figure S30. ITC heats of injection and binding isotherm of mPEG-GA titrated with mPEG-FPBA Trial 1 conducted at 25 °C.

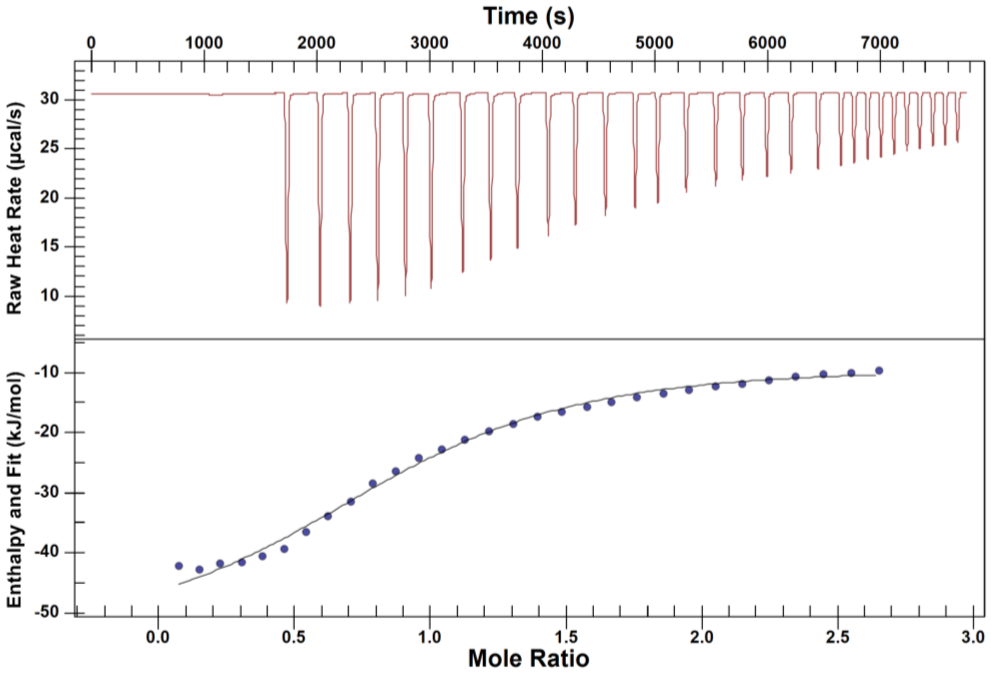

Figure S31. ITC heats of injection and binding isotherm of mPEG-GA titrated with mPEG-FPBA Trial 2 conducted at 25 °C.

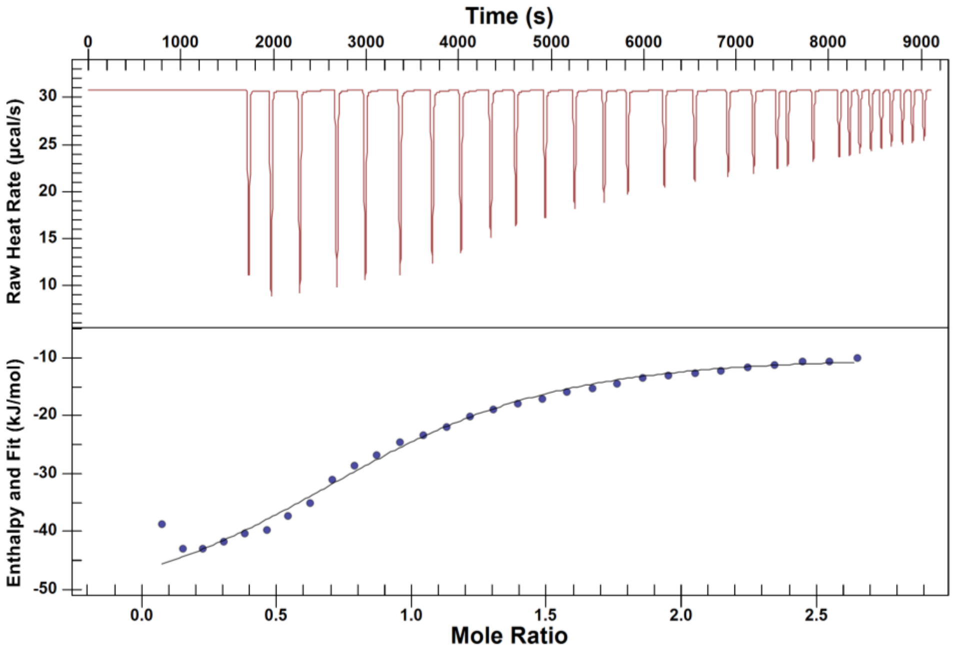

Figure S32. ITC heats of injection and binding isotherm of mPEG-GA titrated with mPEG-FPBA Trial 3 conducted at 25 °C.

Table S5. Summary of ITC fits from mPEG-GA runs determined by kinITC[3].

KinITC	mPEG-*GA*						
Trial	n	Kd (M)	Ka (M^{-1})	Ka std	koff ($M^{-1}s^{-1}$)	koff std	kon
1	8.77E-1	4.67E-4	2.14E+3	1.40E+1	2.04E-1	2.41E-2	4.36E+2
2	8.92E-1	4.35E-4	2.30E+3	1.91E+1	1.86E-1	7.05E-2	4.28E+2
3	1.01E+0	4.73E-4	2.11E+3	3.66E+1	3.85E-1	6.99E-2	8.15E+2
avg	9.25E-1	4.58E-4	2.18E+3		2.58E-1		5.60E+2
std	7.06E-2	2.06E-5	1.01E+2		1.10E-1		2.21E+2

4.7. Cl-ana (5)

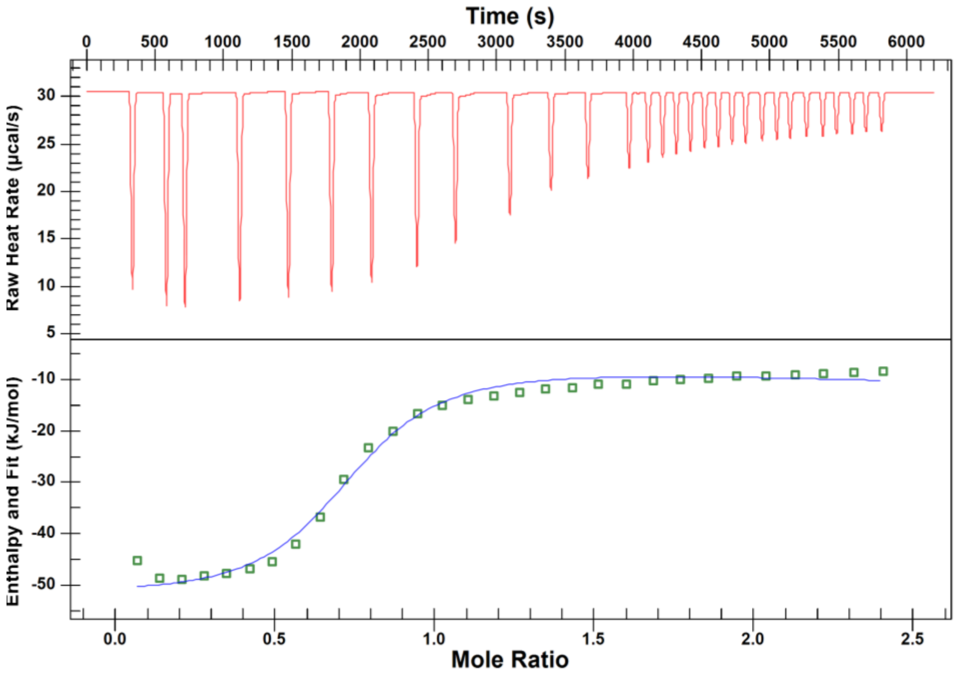

Figure S33. ITC heats of injection and binding isotherm of cl-ana titrated with mPEG-FPBA Trial 1 conducted at 25 °C.

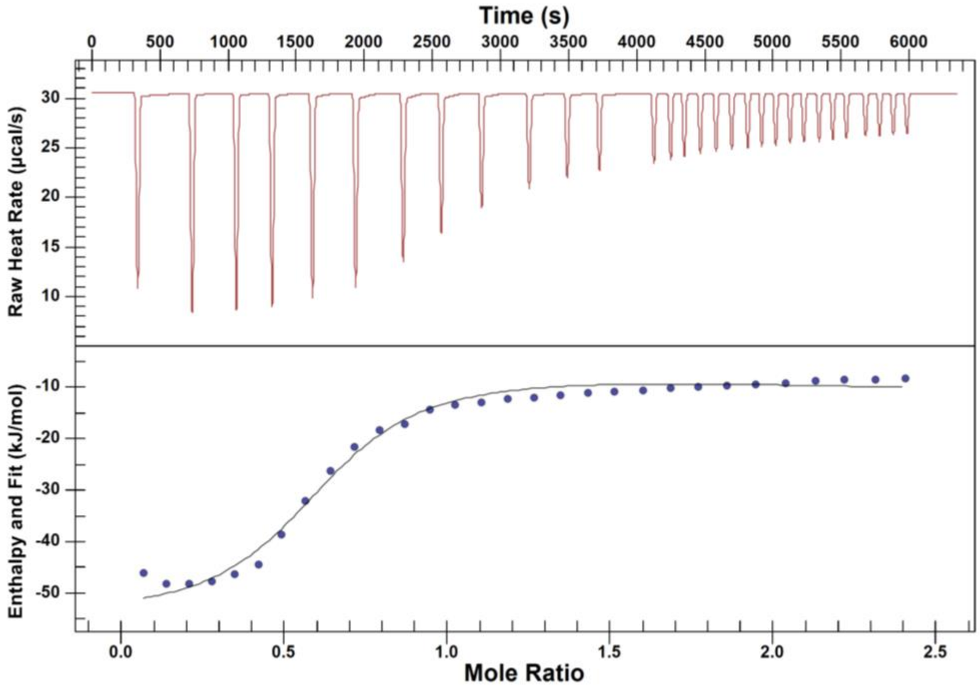

Figure S34. ITC heats of injection and binding isotherm of cl-ana titrated with mPEG-FPBA Trial 2 conducted at 25 °C.

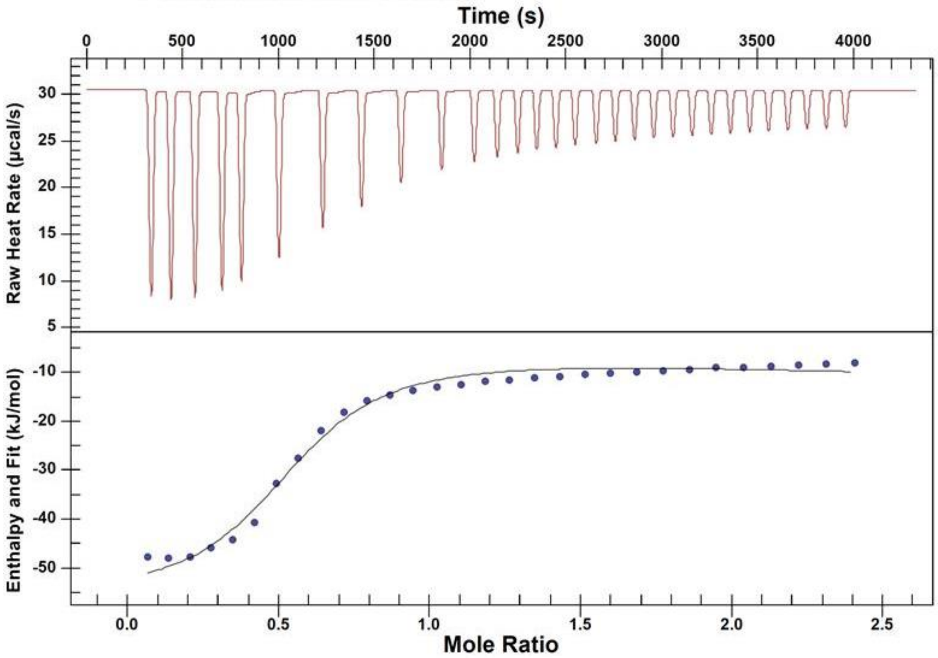

Figure S35. ITC heats of injection and binding isotherm of cl-ana titrated with mPEG-FPBA Trial 3 conducted at 25 °C.

Table S6. Summary of ITC fits from cl-ana runs determined by kinITC[3].

KinITC	*cl-ana*						
Trial	n	Kd (M)	Ka (M^{-1})	Ka std	koff ($M^{-1}s^{-1}$)	koff std	kon
1	7.28E-1	2.74E-4	3.65E+3	4.52E+1	1.11E-1	1.96E-2	4.04E+2
2	5.19E-1	1.96E-4	5.09E+3	9.30E+1	1.04E-1	2.24E-2	5.28E+2
3	6.74E-1	2.07E-4	4.83E+3	7.44E+1	1.01E-1	1.48E-2	4.89E+2
avg	6.40E-1	2.26E-4	4.52E+3		1.05E-1		4.74E+2
std	1.09E-1	4.21E-5	7.69E+2		4.92E-3		6.34E+1

4.8. Dopamine (6)

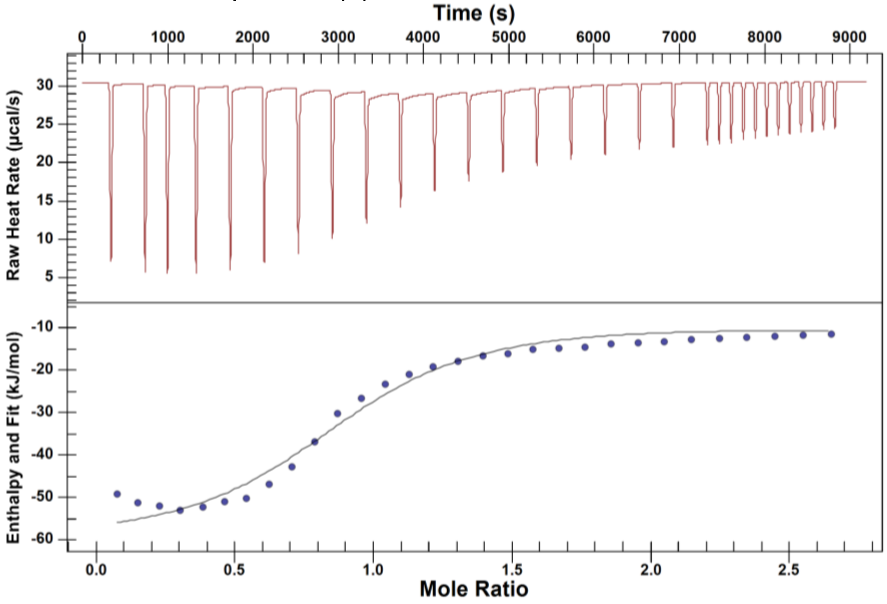

Figure S36. ITC heats of injection and binding isotherm of Dopamine titrated with mPEG-FPBA Trial 1conducted at 25 °C.

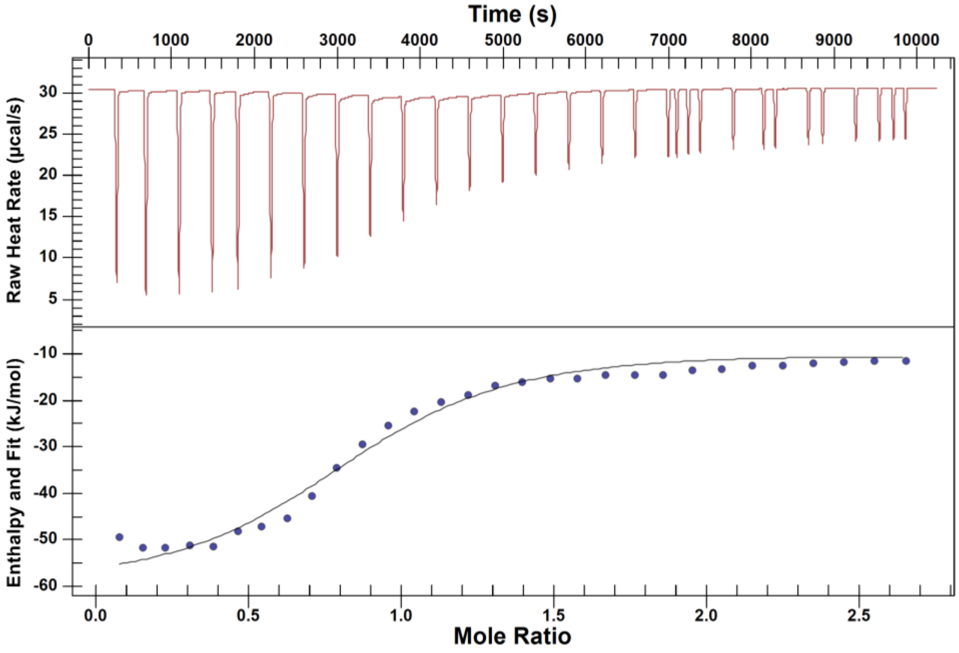

Figure S37. ITC heats of injection and binding isotherm of Dopamine titrated with mPEG-FPBA Trial 2 conducted at 25 °C.

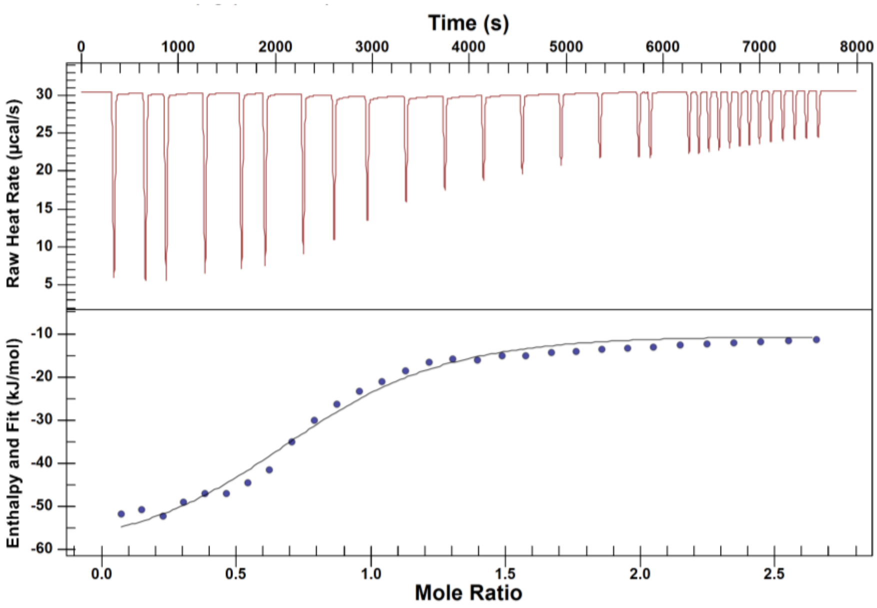

Figure S38. ITC heats of injection and binding isotherm of Dopamine titrated with mPEG-FPBA Trial 3 conducted at 25 °C.

Table S7. Summary of ITC fits from dopamine runs determined by kinITC[3].

KinITC	**Dopamine**						
Trial	n	Kd (M)	Ka (M^{-1})	Ka std	koff ($M^{-1}s^{-1}$)	koff std	kon
1	6.87E-1	1.34E-4	7.47E+3	7.38E+1	7.64E-2	1.05E-2	5.71E+2
2	7.46E-1	1.62E-4	6.17E+3	1.51E+2	6.08E-2	1.81E-2	3.76E+2
3	7.18E-1	1.52E-4	6.58E+3	1.25E+2	4.52E-2	5.88E-3	2.98E+2
avg	7.17E-1	1.49E-4	6.74E+3		6.08E-2		4.15E+2
std	2.93E-2	1.42E-5	6.63E+2		1.56E-2		1.41E+2

4.9. ITC Data Summary

Table S8. Summary of thermodynamics and kinetics of boronate ester system

Diol	K_a (M^{-1})	k_{off} (s^{-1})
mPEG-GA	2200 ± 100	0.26 ±0.11
Glucose (1)	6.2 ± 3.2	1.47 ± 0.88
Dyphylline (2)	26.0 ± 3.8	238 ± 69
Tris (3)	310 ± 60	164 ± 39
Capecitabine (4)	450 ± 20	131.8 ± 14
Crosslinker analogue (5)	4530 ± 60	0.105 ± 0.005
Dopamine (6)	6700 ±600	0.06 ± 0.01

5. Rheology

5.1. Formation of Hydrogels:

To synthesize dynamic hydrogels for the boronate ester system, 10 weight/volume percent (w/v%) solutions of 4PEG-phenylboronic acid and 4PEG-GA were prepared in 0.1 M HEPES buffer at pH 7.4 ± 0.1. We avoided PBS buffer due to its potential to form competing complexes via Lewis adduct formation between phosphates and boronic acid.[18] Upon mixing the 10 w/v% solutions, hydrogels formed within 3 seconds. For the inhibited gels, 4PEG-GA was dissolved at 10 w/v% and pre-mixed with the diol competitor to prevent pre-association with the boronic acid. To ensure the gels were fully equilibrated a time sweep at 10 rad/s at 1% strain for 300 seconds was performed to show there was constant modulus. See pictured gels in figure S39.

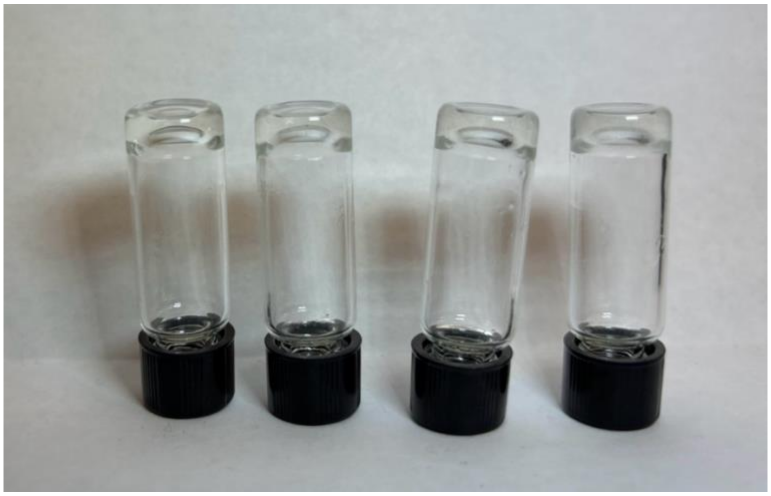

Figure S39. Photograph of boronate ester hydrogels with 0, 20, 30, and 40 mM (from left to right) of competitor (2). All gels were optically clear.

A similar procedure was used for the hydrazone network. Initially, 4PEG-Ar-CHO and 4PEG-Hz were prepared as 0.01 M PBS buffer solutions at pH 7.4 ± 0.1. However, due to the inherent basic pH of the MeHz competitor (e.g. the pH of 18mM MeHz in PBS 1X buffer is about 9.0), pH adjustment is required to keep the hydrogel at a constant pH of 7.4 ± 0.1. The stock solution of MeHz was prepared at 180 mM in PBS 1X, with a pH of about 9.60. Reagent grade HCl was used to adjust the pH to 7.4 ± 0.1. Then, sodium chloride solution (0.037 M in PBS 1X) was added, so that the total saline ionic concentration (excluding PBS 1X) is 0.195 M. The ionic contribution of the PBS 1X is ignored and assumed to be constant throughout the solutions given that it is used as a diluting solvent for all solutions, including the sodium chloride solution. Finally, the volume of MeHz is diluted to 180 mM using PBS 1X. It is important to limit variations in the procedure for preparing hydrogels to achieve consistent rheological measurements.

For the hydrazone system 200 µL gels (72 mM functional groups for both in a final saline concentration of 0.0975 M NaCl in PBS 1X) were prepared by separately dissolving 4PEG-Ar-CHO 18 mM (in 100 µL PBS 1X) and 4PEG-Hz 18 mM (in 100 µL 0.195 M NaCl solution in PBS 1X) followed by mixing and vortexing the solutions. Gels were then allowed to sit at room temperature until gelation occurred. For the competitive hydrogel, the MeHz competitor was added to the solution of 4APEG-Hz. The ionic saline concentration of this 4PEG-Hz solution (0.195 M NaCl) is always kept constant by compensating for the dilution with PBS buffer by adding NaCl solution (0.195 M). For example, MeHz (27 mM with 0.195 M NaCl in PBS 1X) will need 30 µL of MeHz stock solution (180 mM MeHz with 0.195 M NaCl in PBS 1X) and compensated with 70 µL of dilute NaCl solution (0.195 M), yielding a final 100 µL of 0.195 M NaCl concentration.

The 4PEG-hydrazine and 4PEG-Ar-CHO were prepared as 9 w/v% solutions and mixed, with gelation occurring in a minute. The gels were allowed to equilibrate overnight before measurements because the slow kinetics of the hydrazone formation.

5.2. Zero Competitor

Table S9. Summary of 0 competitor rheology results.

Run	Gp (kPa)	Cross Freq (rad /s)	τ (s) from crossover
1	21.5	1.35	0.741
2	21.6	1.34	0.746
3	21.9	1.44	0.694
Average	21.7	1.38	0.727
std	0.192	0.0565	0.029

5.3. Glucose (1)

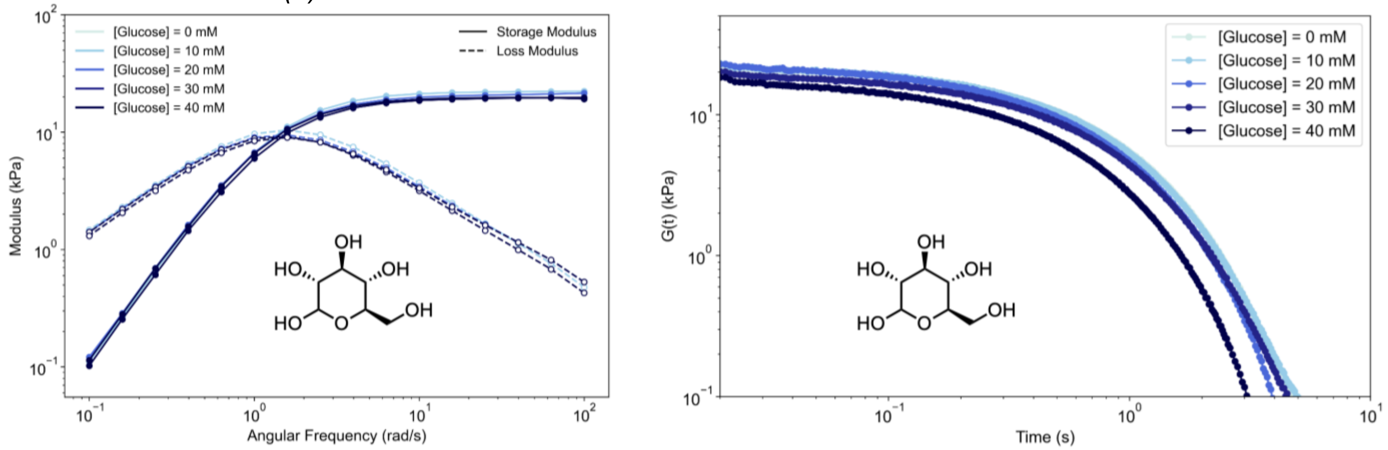

Figure S40. Frequency-dependent storage (solid line) and loss modulus (dashed line) from 0.1 rad/s – 100 rad/s, 1% strain and stress relaxation profiles at 5% strain of glucose competitor (1) added to boronate ester hydrogel at concentrations 0 – 40 mM at 25 °C.

Table S10. Summary of glucose rheology results.

Glucose	10 mM		
Run	Gp (kPa)	Cross Freq (rad /s)	τ (s) from crossover
1	21.2	1.48	0.676
2	20.6	1.41	0.709
3	21.6	1.38	0.725
Average	21.1	1.42	0.704
std	0.481	0.0494	0.025
Glucose	20 mM		
Run	Gp (kPa)	Cross Freq (rad /s)	τ (s) from crossover
1	20.1	1.38	0.725
2	21.1	1.37	0.730
3	21.0	1.49	0.671
Average	20.7	1.41	0.709
std	0.566	0.0667	0.033
Glucose	30 mM		
Run	Gp (kPa)	Cross Freq (rad /s)	τ (s) from crossover
1	19.6	1.42	0.704
2	17.9	1.46	0.685
3	20.5	1.44	0.694
Average	19.3	1.44	0.694
std	1.35	0.0216	0.010
Glucose	40 mM		
Run	Gp (kPa)	Cross Freq (rad /s)	τ (s) from crossover
1	20.5	1.36	0.735
2	21.7	1.80	0.556
3	18.6	1.77	0.565
Average	20.2	1.65	0.606
std	1.55	0.247	0.101

5.4. *Dyphylline (2)*

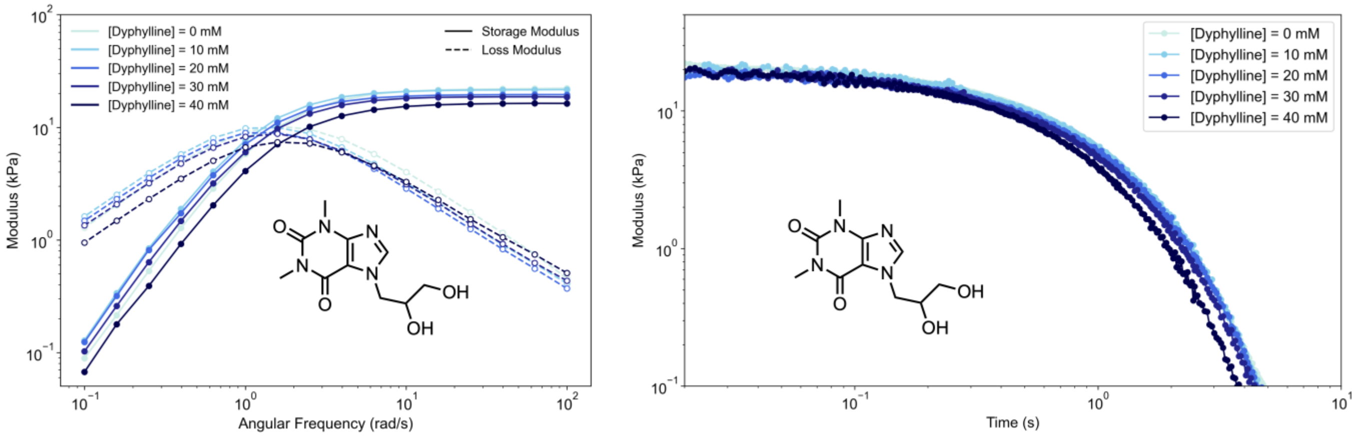

Figure S41. Frequency-dependent storage (solid line) and loss modulus (dashed line) from 0.1 rad/s – 100 rad/s, 1% strain and stress relaxation profiles at 5% strain of dyphylline competitor (2) added to boronate ester hydrogel at concentrations 0 – 40 mM at 25 °C.

Table S11. Summary of dyphylline rheology results.

Dyphylline	10 mM		
Run	Gp (kPa)	Cross Freq (rad /s)	τ (s) from crossover
1	21.6	1.38	0.725
2	20.5	1.28	0.781
3	22.9	1.30	0.769
Average	21.7	1.32	0.758
std	1.22	0.0539	0.030
Dyphylline	20 mM		
Run	Gp (kPa)	Cross Freq (rad /s)	τ (s) from crossover
1	17.5	1.38	0.725
2	20.6	1.30	0.769
3	20.0	1.46	0.685
Average	19.4	1.38	0.725
std	1.68	0.0820	0.042
Dyphylline	30 mM		
Run	Gp (kPa)	Cross Freq (rad /s)	τ (s) from crossover
1	16.7	1.31	0.763
2	19.3	1.41	0.709
3	20.1	1.37	0.730
Average	18.7	1.36	0.735
std	1.77	0.0524	0.027
Dyphylline	40 mM		
Run	Gp (kPa)	Cross Freq (rad /s)	τ (s) from crossover
1	16.2	1.58	0.633
2	18.9	1.71	0.585
3	20.1	1.63	0.613
Average	18.4	1.64	0.610
std	1.98	0.0652	0.024

5.5. *Tris (3)*

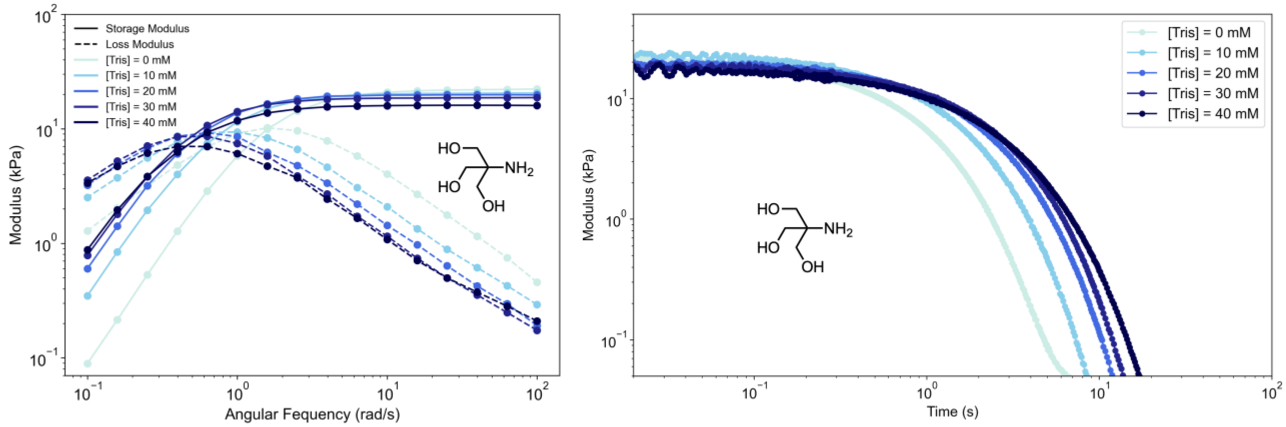

Figure S42. Frequency-dependent storage (solid line) and loss modulus (dashed line) from 0.1 rad/s – 100 rad/s, 1% strain and stress relaxation profiles at 5% strain of tris competitor (3) added to boronate ester hydrogel at concentrations 0 – 40 mM at 25 °C.

Table S12. Summary of tris rheology results.

Tris	10 mM		
Run	Gp (kPa)	Cross Freq (rad /s)	τ (s) from crossover
1	16.9	0.884	1.13
2	19.6	0.796	1.26
3	21.5	0.885	1.13
Average	19.3	0.855	1.17
std	2.29	0.0511	0.07
Tris	20 mM		
Run	Gp (kPa)	Cross Freq (rad /s)	τ (s) from crossover
1	16.2	0.623	1.61
2	19.3	0.583	1.72
3	20.7	0.677	1.48
Average	18.7	0.627	1.59
std	2.31	0.0474	0.12
Tris	30 mM		
Run	Gp (kPa)	Cross Freq (rad /s)	τ (s) from crossover
1	15.6	0.532	1.88
2	18.3	0.497	2.01
3	18.3	0.478	2.09
Average	17.4	0.503	1.99
std	1.58	0.0274	0.11
Tris	40 mM		
Run	Gp (kPa)	Cross Freq (rad /s)	τ (s) from crossover
1	13.4	0.479	2.09
2	15.4	0.453	2.21
3	15.1	0.440	2.27
Average	14.6	0.458	2.18
std	1.10	0.0197	0.09

5.6. Capecitabine (4)

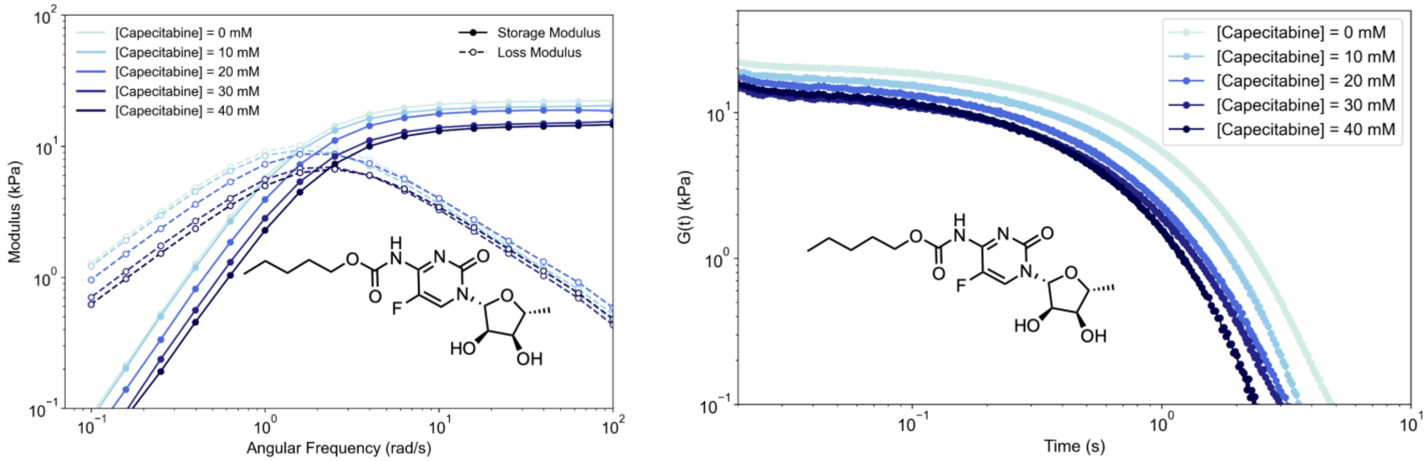

Figure S43. Frequency-dependent storage (solid line) and loss modulus (dashed line) from 0.1 rad/s – 100 rad/s, 1% strain and stress relaxation profiles at 5% strain of capecitabine competitor (4) added to boronate ester hydrogel at concentrations 0 – 40 mM at 25 °C.

Table S13. Summary of capecitabine rheology results.

Capecitabine	10 mM		
Run	Gp (kPa)	Cross Freq (rad /s)	τ (s) from crossover
1	18.6	1.55	0.645
2	18.9	1.60	0.625
3	17.1	1.73	0.578
Average	18.2	1.63	0.613
std	1.01	0.0940	0.034
Capecitabine	20 mM		
Run	Gp (kPa)	Cross Freq (rad /s)	τ (s) from crossover
1	15.7	1.98	0.505
2	15.8	1.59	0.629
3	13.4	1.86	0.538
Average	15.0	1.81	0.552
std	1.32	0.198	0.064
Capecitabine	30 mM		
Run	Gp (kPa)	Cross Freq (rad /s)	τ (s) from crossover
1	11.3	1.89	0.529
2	13.8	2.03	0.493
3	12.5	1.97	0.508
Average	12.5	1.96	0.510
std	1.25	0.0716	0.018
Capecitabine	40 mM		
Run	Gp (kPa)	Cross Freq (rad /s)	τ (s) from crossover
1	12.9	2.28	0.439
2	10.8	2.13	0.469
3	11.0	2.16	0.463
Average	11.6	2.19	0.457
std	1.16	0.0773	0.016

5.7. Crosslink-analogue (cl-ana) (5)

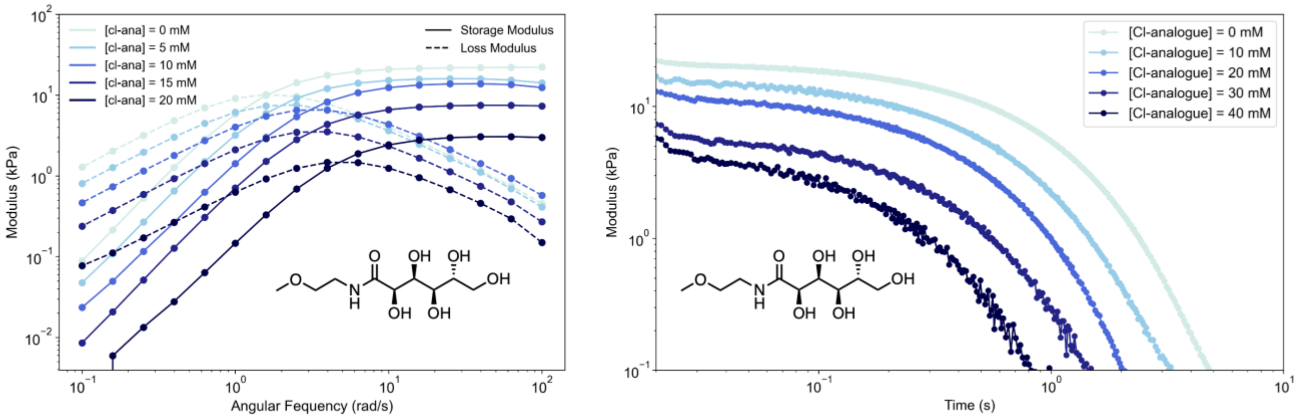

Figure S44. Frequency-dependent storage (solid line) and loss modulus (dashed line) from 0.1 rad/s – 100 rad/s, 1% strain and stress relaxation profiles at 5% strain of cl-ana competitor (5) added to boronate ester hydrogel at concentrations 0 – 20 mM at 25 °C.

Table S14. Summary of cl-ana rheology results.

Cl-ana	5 mM		
Run	Gp (kPa)	Cross Freq (rad /s)	τ (s) from crossover
1	13.5	1.91	0.524
2	11.0	2.02	0.495
3	13.2	2.01	0.498
Average	12.6	1.98	0.505
std	1.40	0.0609	0.016
Cl-ana	10 mM		
Run	Gp (kPa)	Cross Freq (rad /s)	τ (s) from crossover
1	10.6	3.07	0.326
2	9.35	2.71	0.369
3	9.81	2.56	0.391
Average	9.93	2.78	0.360
std	0.654	0.259	0.033
Cl-ana	15 mM		
Run	Gp (kPa)	Cross Freq (rad /s)	τ (s) from crossover
1	4.99	3.37	0.297
2	5.23	3.13	0.319
3	6.60	3.16	0.316
Average	5.61	3.22	0.311
std	0.871	0.131	0.012
Cl-ana	20 mM		
Run	Gp (kPa)	Cross Freq (rad /s)	τ (s) from crossover
1	2.42	4.77	0.210
2	2.54	5.16	0.194
3	2.86	5.49	0.182
Average	2.60	5.14	0.195
std	0.227	0.360	0.014

5.8. *Dopamine (6)*

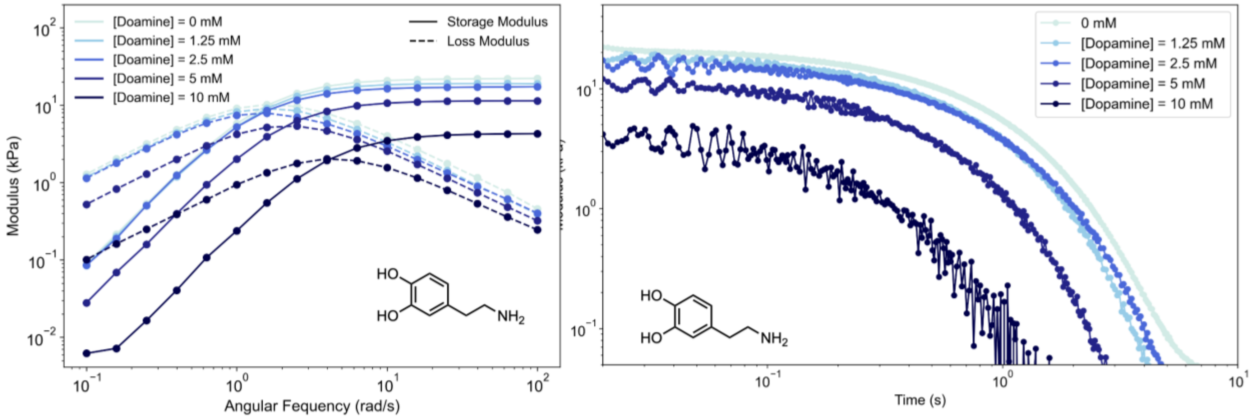

Figure S45. Frequency-dependent storage (solid line) and loss modulus (dashed line) from 0.1 rad/s – 100 rad/s, 1% strain and stress relaxation profiles at 5% strain of dopamine competitor (6) added to boronate ester hydrogel at concentrations 0 – 10 mM at 25 °C.

Table S15. Summary of dopamine rheology results.

Dopamine	1.25 mM		
Run	Gp (kPa)	Cross Freq (rad /s)	τ (s) from crossover
1	13.6	1.41	0.709
2	15.1	1.35	0.741
3	15.3	1.54	0.649
4	16.3	1.46	0.685
Average	15.1	1.44	0.694
std	1.11	0.0808	0.039
Dopamine	2.5 mM		
Run	Gp (kPa)	Cross Freq (rad /s)	τ (s) from crossover
1	16.3	1.61	0.621
2	16.4	1.61	0.621
3	18.2	1.58	0.633
4	17.7	1.56	0.641
Average	17.2	1.59	0.629
std	0.936	0.0229	0.010
Dopamine	5 mM		
Run	Gp (kPa)	Cross Freq (rad /s)	τ (s) from crossover
1	10.1	2.21	0.452
2	10.5	2.20	0.455
3	10.4	2.11	0.474
4	11.2	2.20	0.455
Average	10.6	2.16	0.463
std	0.463	0.0526	0.010
Dopamine	10 mM		
Run	Gp (kPa)	Cross Freq (rad /s)	τ (s) from crossover
1	3.45	4.19	0.239
2	3.50	4.21	0.238
3	3.63	4.04	0.248
Average	3.53	4.15	0.241
std	0.0911	0.0971	0.005

5.9. Addition of Competitive Inhibitor Post-Gelation

To evaluate whether gel properties could be modified after network formation, hydrogels were first formed by mixing equal (100 µL of each) of the polymer solutions to yield an uninhibited gel. Solid competitive inhibitor was then deposited directly onto the gel surface and allowed to dissolve and diffuse into the network.

Addition of 1.2 mg of competitor (6) (Dopamine, a strong inhibitor), corresponding to a final concentration of 32 mM in a 200 µL gel, resulted in rapid dissolution of the solid into the gel, followed by complete network dissolution within approximately 2 minutes. In a second experiment, 2.9 mg of competitor (4) (Capecitabine, a medium inhibitor), corresponding to a 40 mM concentration, was added to a pre-formed gel. The solid dissolved into the gel within ~1 min, after which the sample was transferred to the rheometer for mechanical characterization. The resulting gel exhibited a crossover frequency of 2.28 rad/s and a plateau modulus 11.1 kPa (Fig. S46) – similar to those prepared with 40 M (4) mixed with diol. [MH1.1][C1.2]We anticipate greater variability in this approach as there is more uncertainty weighing out small masses of competitive inhibitors, compared to making stock solutions at the desired concentration.

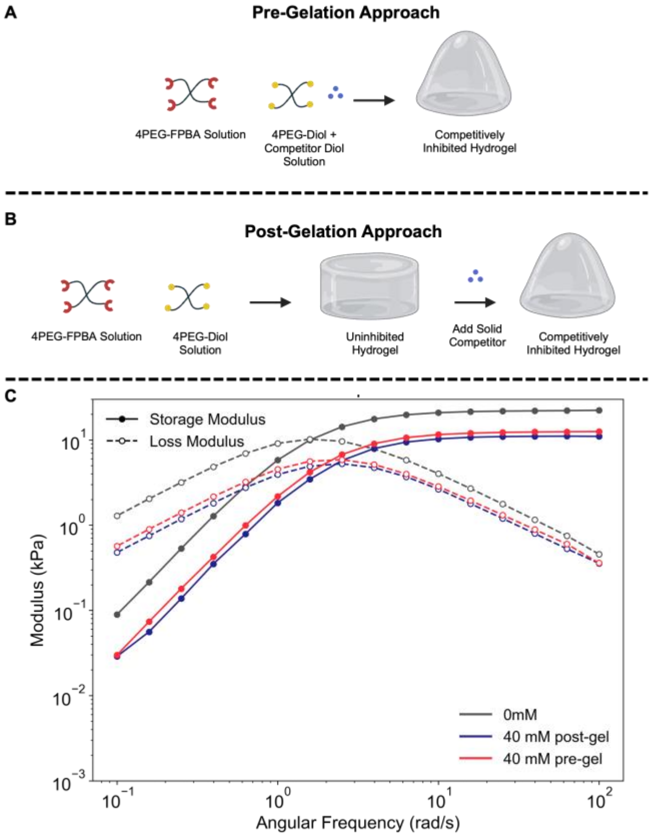

Figure S46. Comparison of pre- and post-gelation strategies for competitive inhibition of boronate ester hydrogels. (A) Pre-gelation inhibition by forming the network in the presence of competitor. (B) Post-gelation inhibition by adding solid competitor (4) to a pre-formed gel. (C) Frequency-dependent storage (solid symbols) and loss (open symbols) moduli for an uninhibited gel (gray), a gel inhibited post-gelation (blue), and a gel inhibited pre-gelation (red) from 0.1 rad/s – 100 rad/s, 1% strain conducted at 25 °C.

6. Modulus Predictions

6.1. Predicting Modulus From K_a Crosslink and K_a of Competitor

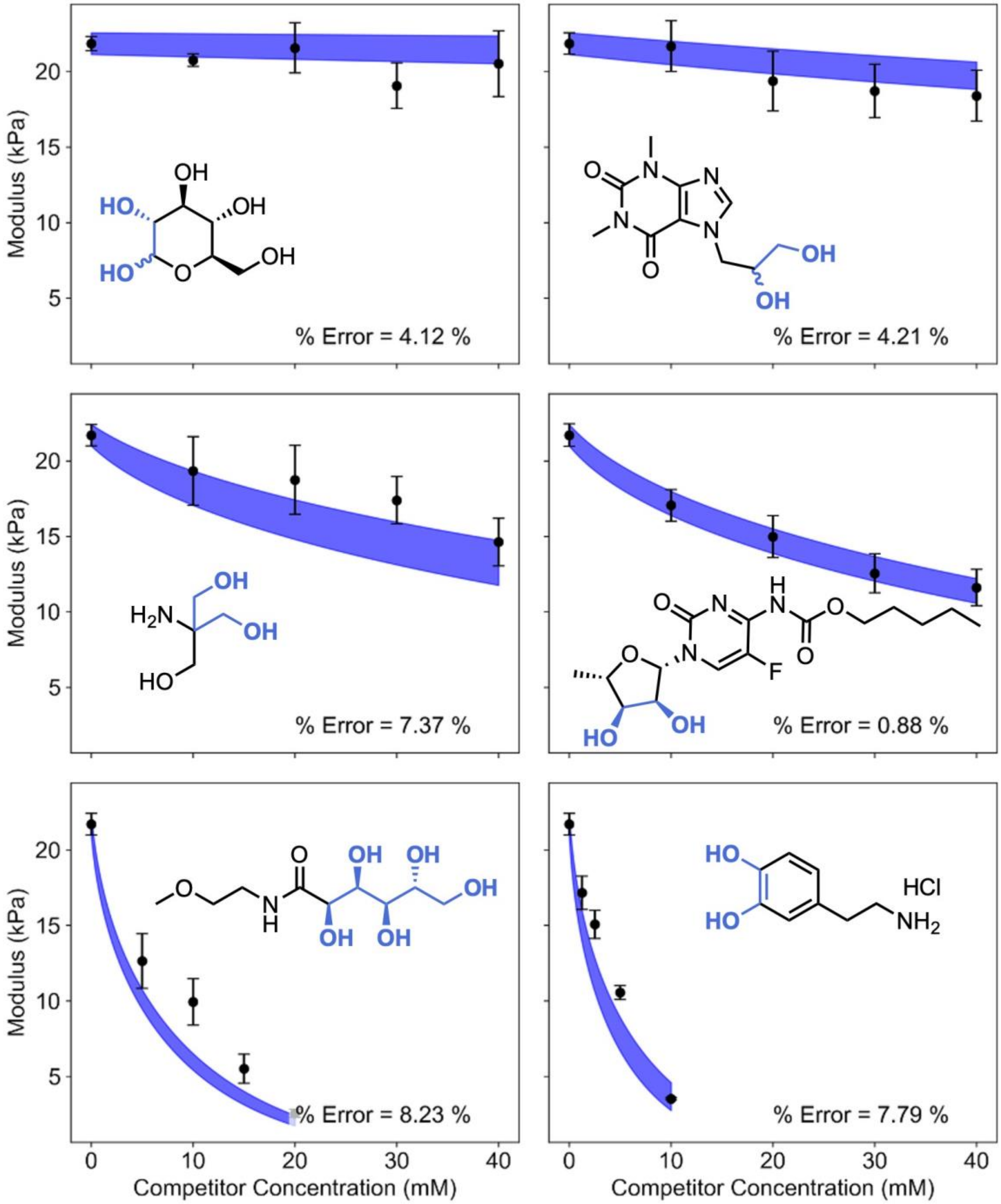

Figure S47. Experimental (black dots with error bars representing standard deviation from triplicate measurements of G_p) and predicted (blue) moduli of boronate ester gels vs concentration of all 6 competitors. The upper and lower limits of the prediction were calculated based on the standard deviation from ITC triplicate measurements.

6.2. Fitting for $K_{a,XL}$ From Known $K_{a,C}$

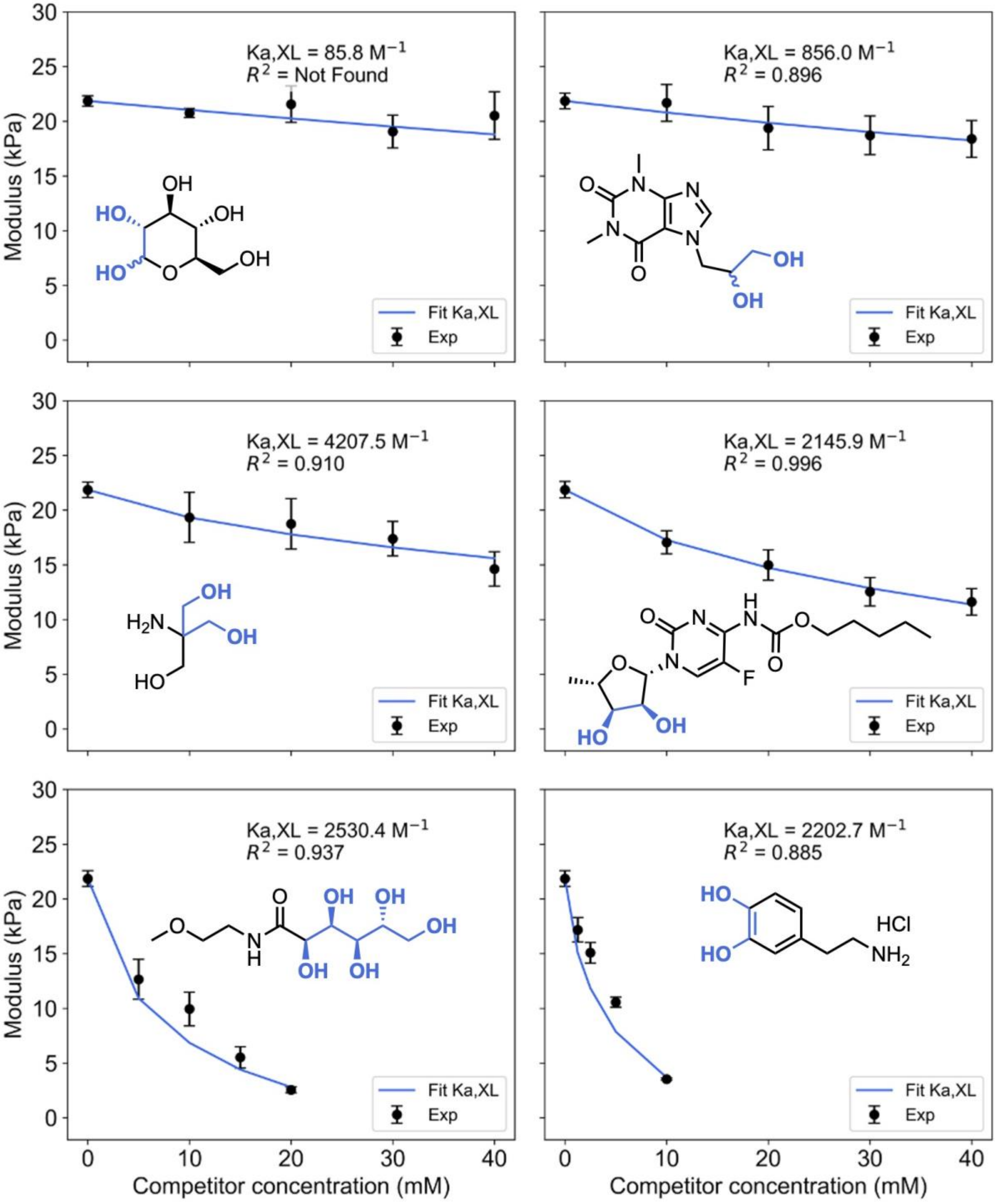

Figure S48. Fitting the modulus vs competitor concentration of all 6 competitors using equations S10, S14, and S20 to fit for the $K_{a,XL}$ of the system.

6.3. Fitting for $K_{a,C}$ From Known $K_{a,XL}$

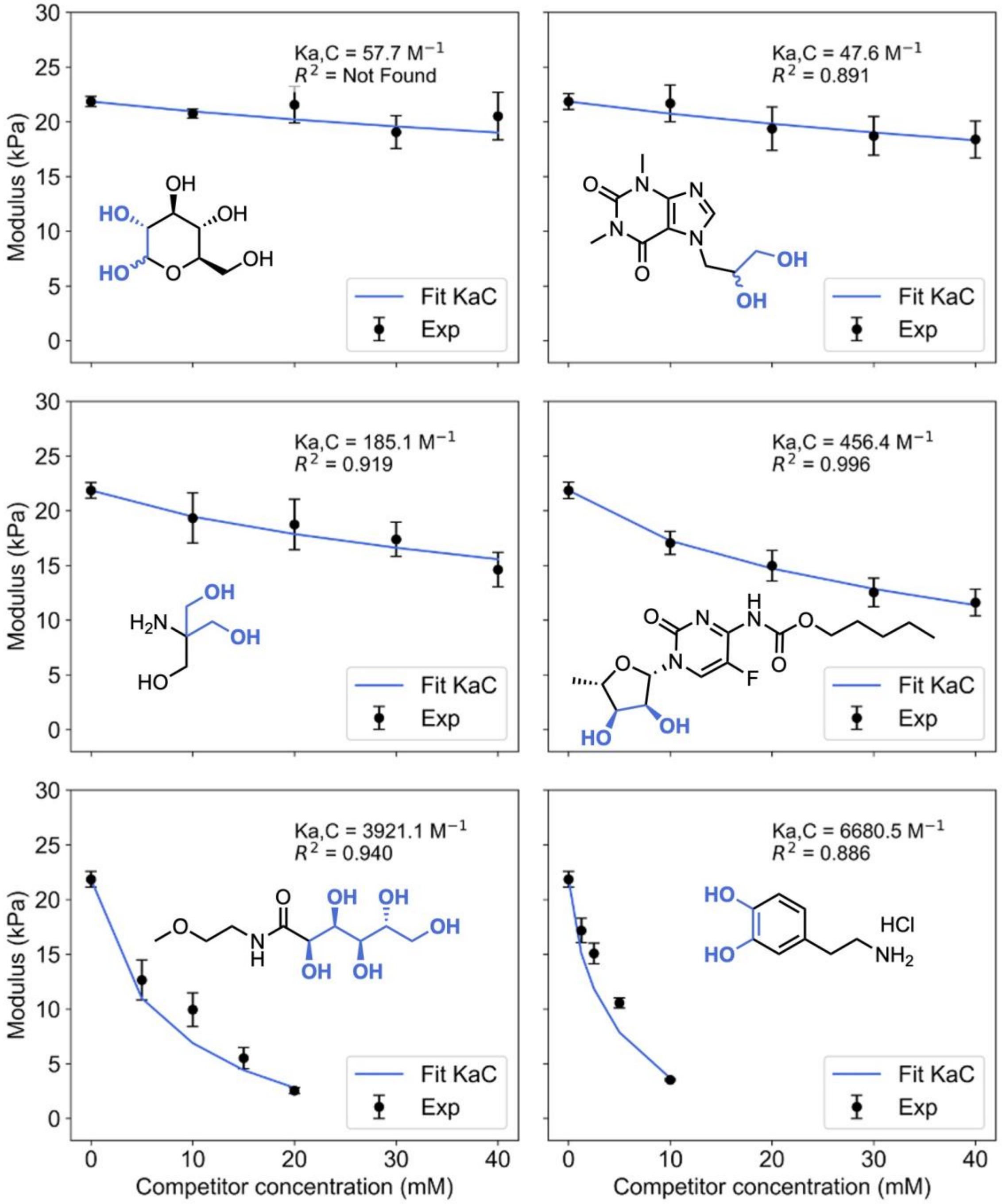

Figure S49. Fitting the modulus vs competitor concentration of all 6 competitors using equations S10, S14, and S20 to fit for the $K_{a,C}$ of the competitor.

7. Tau Predictions

7.1. Representative Stress Relaxation Fits

To find the τ value of our network under various competitor concentrations we compared 3 methods.

7.1.1. Crossover Frequency method[2]

τ can be obtained directly through the crossover frequency (ω), through the relationship:

$$\tau = 1/\,\omega.$$

This method was used to extract tau values for the boronate ester crosslinked networks.

7.1.2. Single Mode Maxwell fit[19]

The normalized relaxation modulus can be fit to a single order Maxwellian fit:

$$G(t)/G_0 = \exp\left(-\frac{t}{\tau}\right).$$

7.1.3. Kohlrausch-Williams-Watts(KWW) fit[20,21]

Finally, the relaxation can be described by the stretched exponential KWW fit:

$$G(t)/G_0 = \exp\left(-\left(\frac{t}{\tau_{kww}}\right)^{\beta}\right).$$

Where β is the stretching exponential. The average τ *value*, <τ>, can be obtained from τ_{kww} and β through:

$$<\tau>= \frac{\tau_{kww}}{\beta}\Gamma\left(\frac{1}{\beta}\right).$$

This method was use for extracting the tau values for the hydrazone crosslinked networks.

Table S16. Summary of dopamine rheology results.

Competitor	Conc (mM)	τ cross freq. (s)	τ maxwell (s)	τ_{kww} (s)	β fit	<τ> (s)
---	0	0.727	0.731	0.769	1.00	0.769
Glucose (1)	10	0.726	0.737	0.737	1.00	0.737
	20	0.694	0.648	0.648	1.00	0.648
	30	0.645	0.548	0.548	1.00	0.548
	40	0.696	0.692	0.692	1.00	0.692
Dyphylline (2)	10	0.758	0.770	0.770	1.00	0.770
	20	0.726	0.777	0.777	1.00	0.777
	30	0.734	0.702	0.702	1.00	0.702
	40	0.610	0.619	0.619	1.00	0.619
Tris (3)	10	1.170	1.136	1.14	0.995	1.14
	20	1.592	1.655	1.66	0.999	1.66
	30	1.989	1.926	1.94	0.977	1.96
	40	2.180	1.989	2.04	0.920	2.12
Capecitabine (4)	10	0.614	0.645	0.645	1.00	0.645
	20	0.552	0.536	0.536	1.00	0.536
	30	0.509	0.508	0.508	1.00	0.508
	40	0.457	0.449	0.449	1.00	0.449
Cl-ana (5)	10	0.505	0.552	0.552	1.00	0.552
	20	0.360	0.411	0.411	1.00	0.411
	30	0.310	0.334	0.334	1.00	0.334
	40	0.194	0.213	0.213	1.00	0.213
Dopamine (6)	10	0.694	0.668	0.668	0.998	0.668
	20	0.629	0.633	0.633	1.00	0.633
	30	0.463	0.471	0.471	1.00	0.471
	40	0.241	0.272	0.272	1.00	0.272

7.2. *Modeling Tau Under Competitive Inhibition*

To demonstrate how our equation for fitting tau is a function of $K_{a,app}$ we added the following expansion of the expression equation 6 in the main text:

$$\tau([C]) = \tau_0 - (\tau_0 - \tau_{min}) \cdot \left(\frac{2v_e}{N_{XL}}\right)$$

Plug in eq S19

$$\tau([C]) = \tau_0 - (\tau_0 - \tau_{min}) \cdot \frac{1}{2}\left(\frac{3}{2} 4P_{out}(1 - P_{out})^3 + 2(1 - P_{out})^4\right) \tag{S21}$$

Plug in eq S16, S17

$$\tau([C]) = \tau_0 - (\tau_0 - \tau_{min}) \cdot \left(\frac{1}{2}\left(6\left[\left(\frac{1}{p} - \frac{3}{4}\right)^{\frac{1}{2}} - \frac{1}{2}\right]\left(1 - \left[\left(\frac{1}{p} - \frac{3}{4}\right)^{\frac{1}{2}} - \frac{1}{2}\right]\right)^3 + 2\left(1 - \left[\left(\frac{1}{p} - \frac{3}{4}\right)^{\frac{1}{2}} - \frac{1}{2}\right]\right)^4\right)\right) \tag{S22}$$

Plug in eq S14

$$\tau([C]) = \tau_0 - (\tau_0 - \tau_{min}) \cdot \frac{1}{2}\left(6\left[\left(\frac{1}{\left(1 + \frac{1}{2N_{XL}K_{a,app}}\right) - \left[\left(1 + \frac{1}{2N_{XL}K_{a,app}}\right)^2 - 1\right]^{1/2}} - \frac{3}{4}\right)^{\frac{1}{2}} - \frac{1}{2}\right]\left(1 - \left[\left(\frac{1}{\left(1 + \frac{1}{2N_{XL}K_{a,app}}\right) - \left[\left(1 + \frac{1}{2N_{XL}K_{a,app}}\right)^2 - 1\right]^{1/2}} - \frac{3}{4}\right)^{\frac{1}{2}} - \frac{1}{2}\right]\right)^3 + 2\left(1 - \left[\left(\frac{1}{\left(1 + \frac{1}{2N_{XL}K_{a,app}}\right) - \left[\left(1 + \frac{1}{2N_{XL}K_{a,app}}\right)^2 - 1\right]^{1/2}} - \frac{3}{4}\right)^{\frac{1}{2}} - \frac{1}{2}\right]\right)^4\right) \tag{S23}$$

To further demonstrate the dependence of tau on competitor binding affinity, we applied linear fits of $\tau([C])$ as a function of [C], and we found that the more strongly associating competitors had steeper linear slopes.

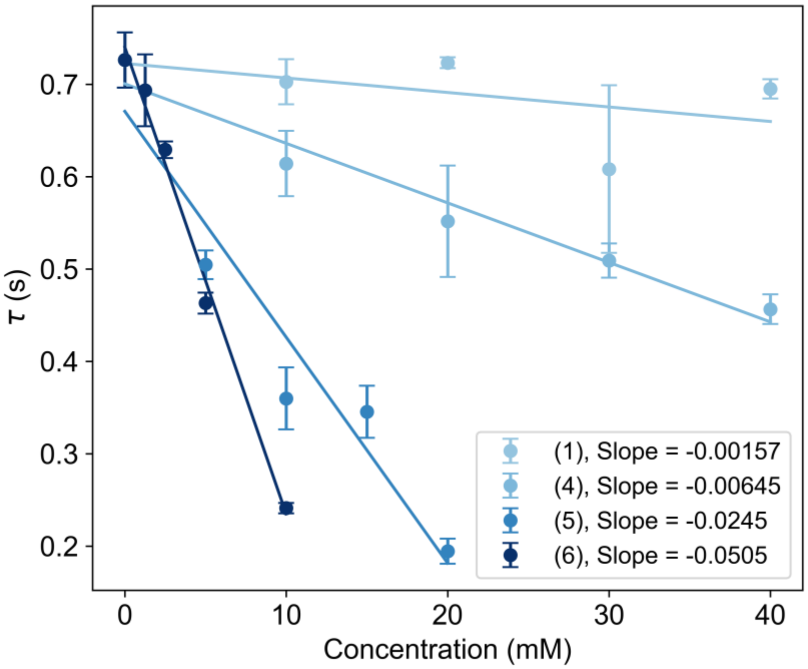

Figure S50. τ vs competitor concentrations of (1), (4), (5), and (6) with a linear fit. As binding affinity for competitor increases from 6.2 M^{-1} (competitor (1) to 6700 M^{-1}(competitor (6)), the magnitude of the slope increases from -0.00157, to -0.0505.

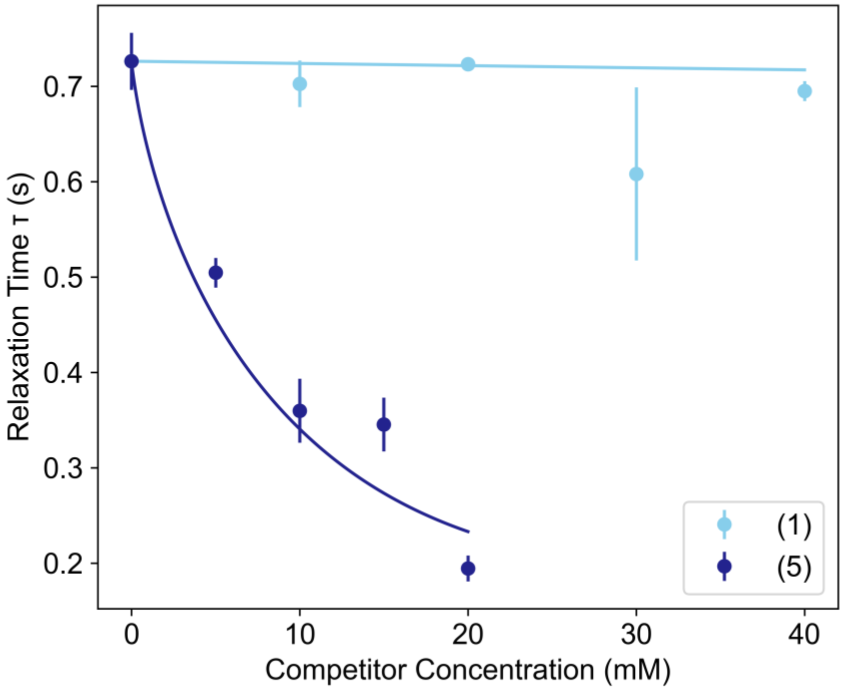

Figure S51. τ vs competitor concentration of , (1) and (5) fitted with Langmuir decay model (equation 6). Where for (1) fitted τ_{min} = 0.001 s, R² = not found and (5) fitted τ_{min} = 0.121 s, R² = 0.9413, with τ values extracted from crossover frequency.

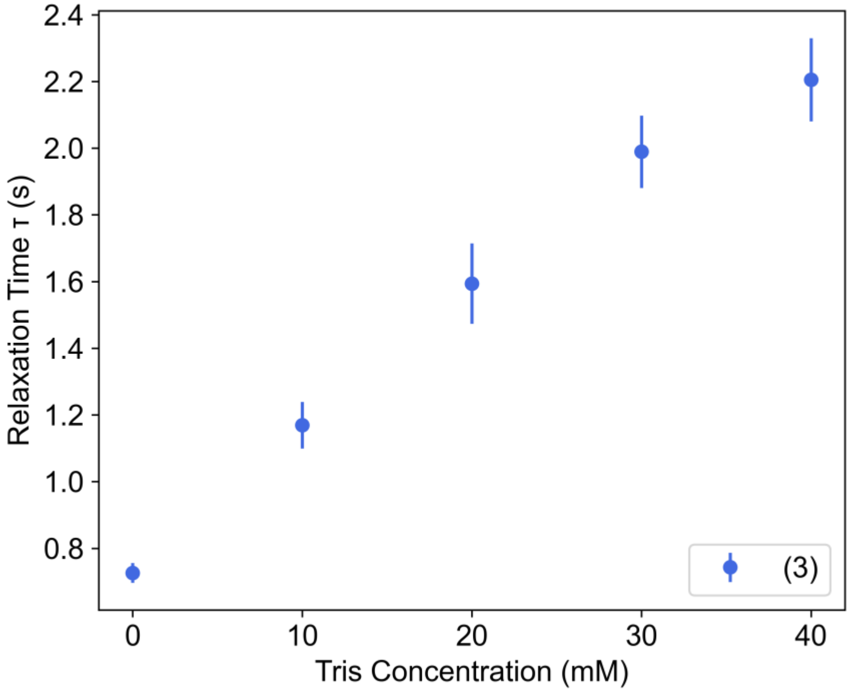

Figure S52. τ vs concentration of (3) with no fit because the τ are increasing, with τ values extracted from crossover frequency.

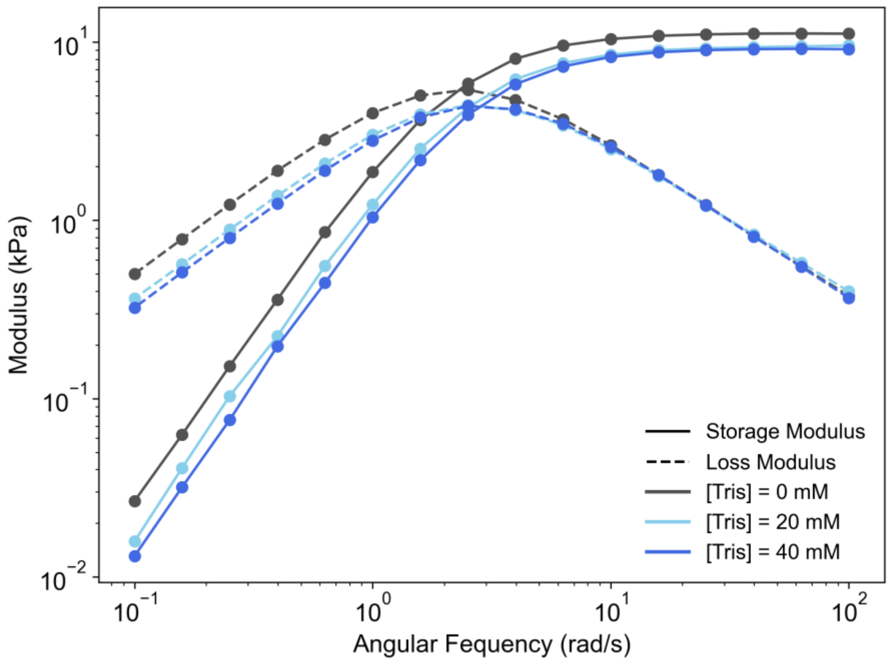

Figure S53. Frequency-dependent storage (solid line) and loss modulus (dashed line) from 0.1 rad/s – 100 rad/s, 1% strain of boronate ester hydrogels with [ion] adjusted to 80 mM with NaCl and pH adjusted to 7.4 with HCl in 0.1 M HEPES. Concentrations range from 0 – 20 mM Tris (4) conducted at 25 °C.

8. Measuring K_a in Hydrazone Crosslinked Hydrogel System

8.1. UV-Vis Titration for MeHz Competitor K_a Determination

For the association constant (K_a) determination, stock solutions of 700 µM 2kDa mPEG-Ar-CHO in PBS 1X and 180 mM MeHz as described above (in 0.195 M NaCl) were made. The desired ratios from 1:1 to 75:1 ([MeHz]:[2kDa mPEG-Ar-CHO]) started from 200 µM with a 3.5 mL total volume per sample. For the hydrazone crosslink, 2kDa mPEG-Hz solution (in 0.195 M NaCl) and 2kDa mPEG-Ar-CHO were titrated in the ratio of 1:1 to 30:1 starting from 50 µM. Each titration was left to equilibrate overnight before the samples were subjected to the UV-Vis experiment. The association constant (K_a) was determined by multiwavelength, non-linear curve fitting using BindFit.[22,23] The spectral region λ = 298 nm – 305 nm was used because this is where the sample absorbs the most. The best fit was obtained using a 1:1 host–guest stoichiometric model.

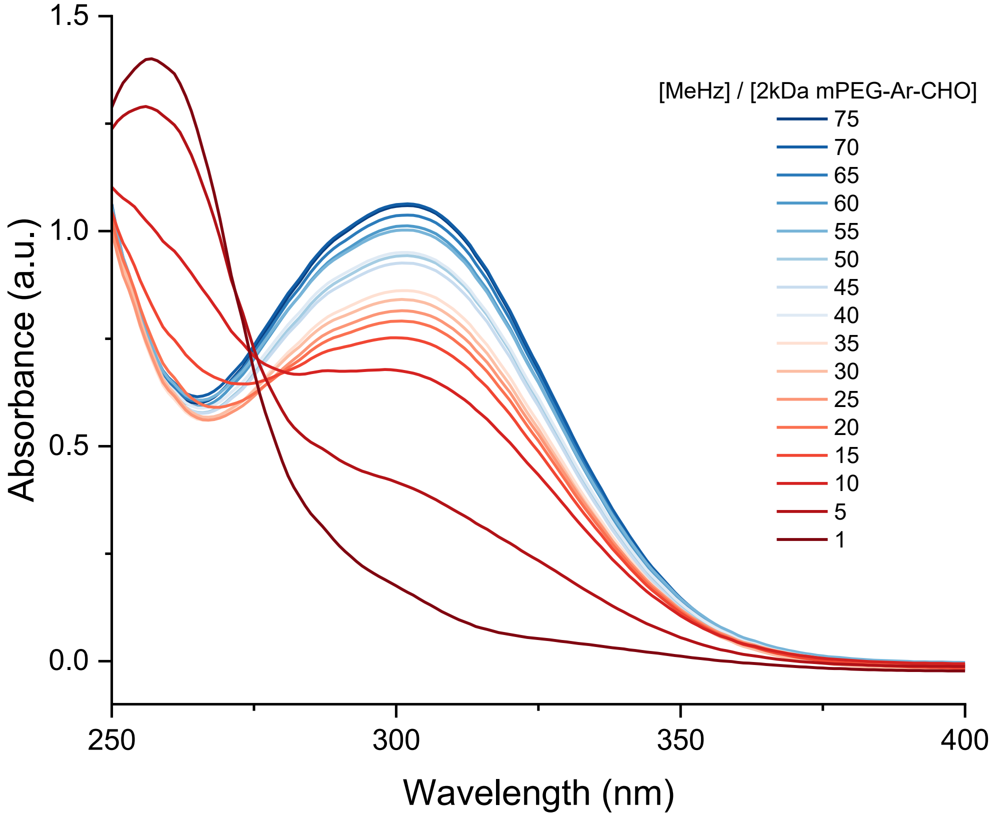

Figure S54. UV-Vis traces stacked in an overlay of all ratios of MeHz to 2kDa mPEG-Ar-CHO in PBS. Replicate 1.

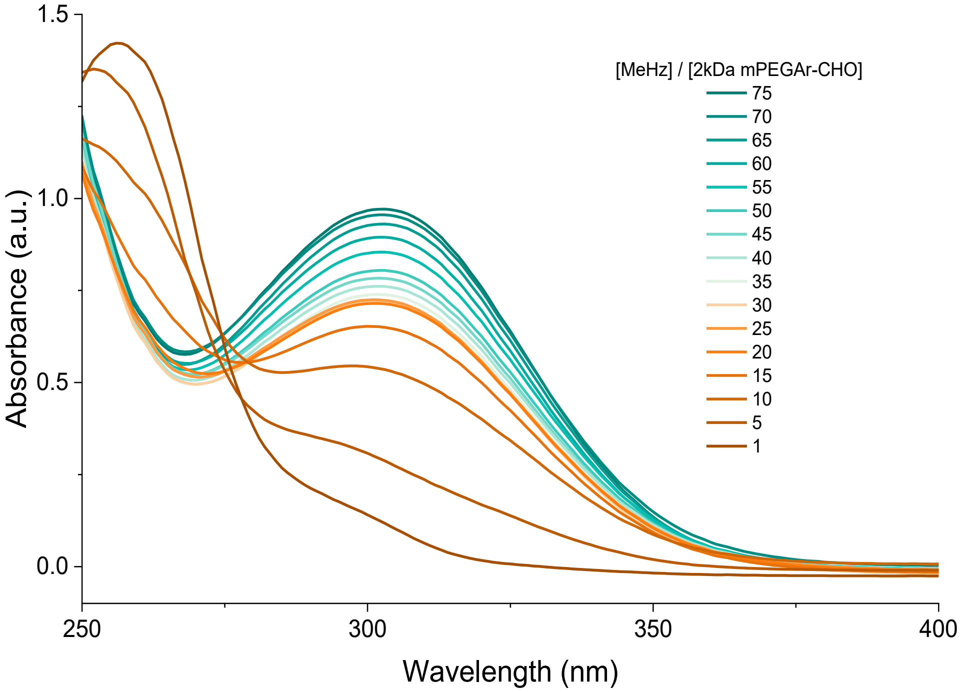

Figure S55. UV-Vis traces stacked in an overlay of all ratios of MeHz to 2kDa mPEG-Ar-CHO in PBS. Replicate 2.

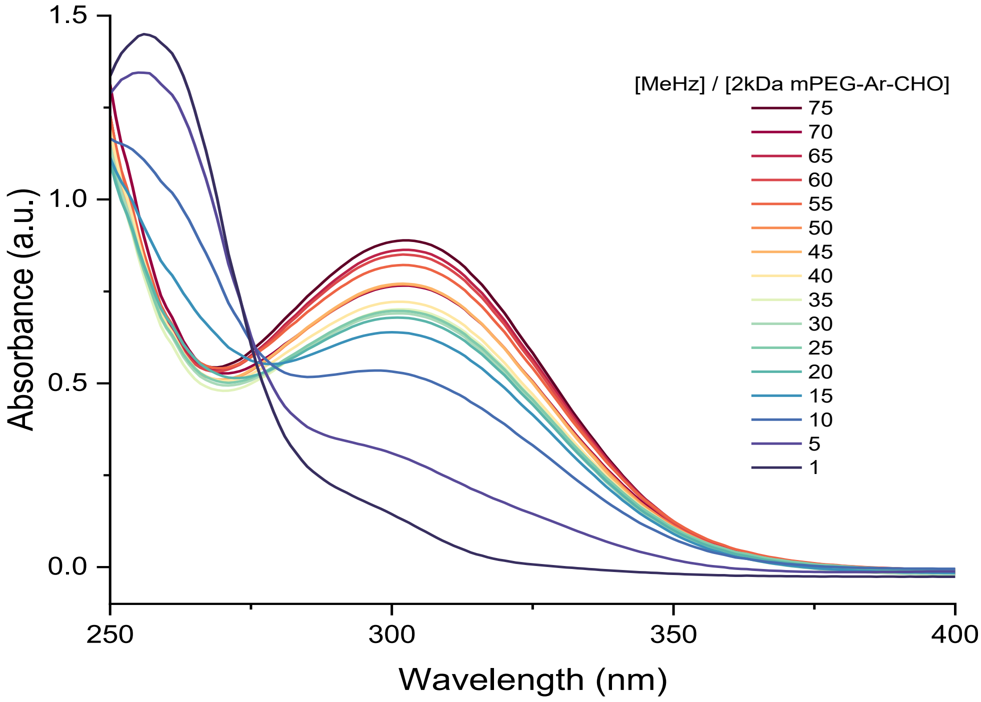

Figure S56. UV-Vis traces stacked in an overlay of all ratios of MeHz to 2kDa mPEG-Ar-CHO in PBS. Replicate 3.

Table S17. Raw λ_{max} absorbance data for each titration.

Ratio ([MeHz]:[2kDa mPEG-Ar-CHO])	Absorbance at λ_{max} (302 nm)		
	Replicate 1	Replicate 2	Replicate 3
1:1	0.160	0.125	0.128
5:1	0.406	0.404	0.299
10:1	0.672	0.538	0.527
15:1	0.750	0.651	0.637
20:1	0.790	0.715	0.678
25:1	0.814	0.724	0.697
30:1	0.840	0.722	0.690
35:1	0.862	0.739	0.701
40:1	0.950	0.761	0.722
45:1	0.926	0.783	0.771
50:1	0.943	0.804	0.769
55:1	1.003	0.854	0.822
60:1	1.012	0.894	0.850
65:1	1.037	0.930	0.863
70:1	1.063	0.955	0.766
75:1	1.060	0.971	0.889

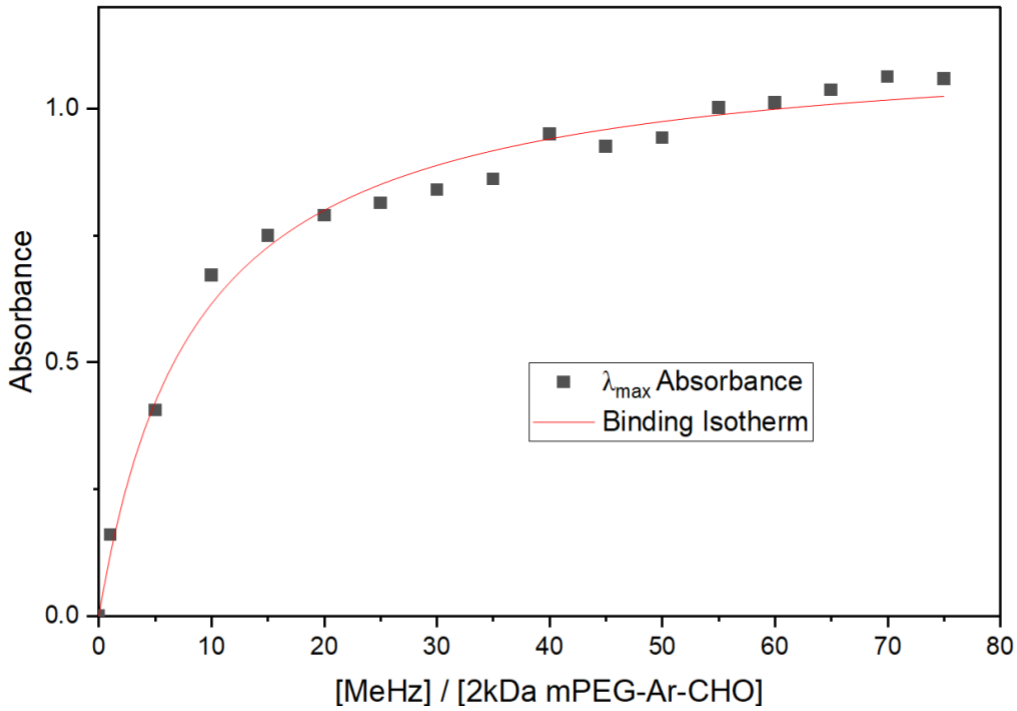

Figure S57. Binding isotherms of all MeHz /2kDa mPEG-Ar-CHO titrations with absorbances obtained from UV-Vis traces. Replicate 1.

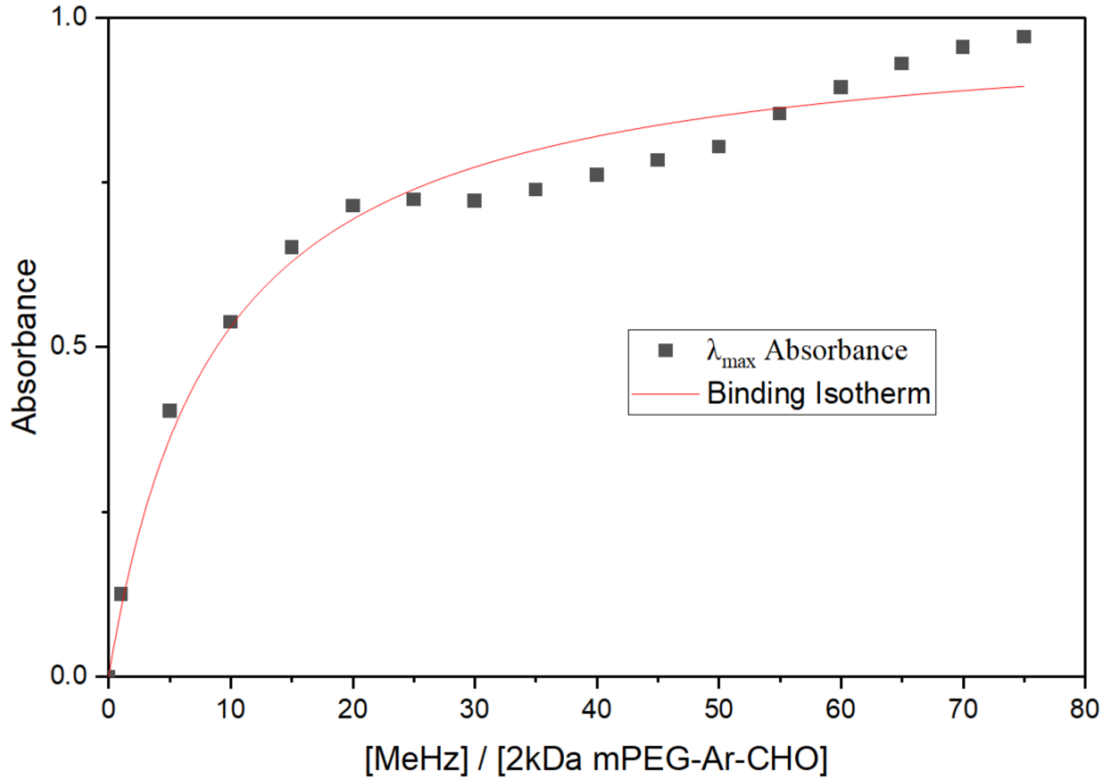

Figure S58. Binding isotherms of all MeHz/2kDa mPEG-Ar-CHO titrations with absorbances obtained from UV-Vis traces. Replicate 2.

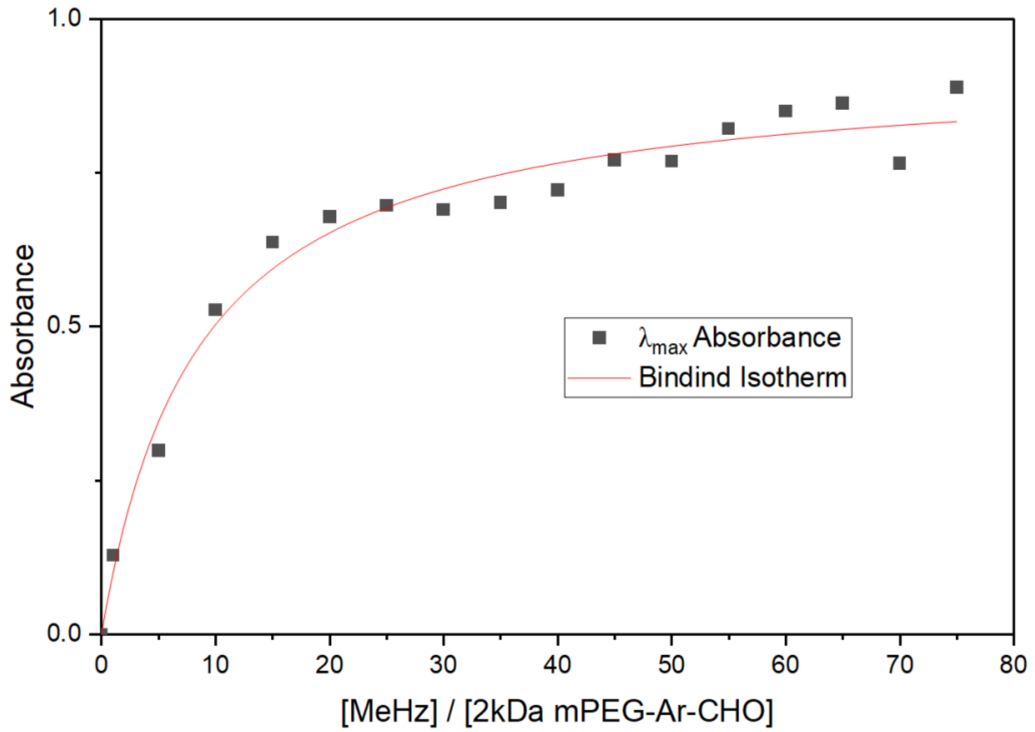

Figure S59. Binding isotherms of all MeHz/2kDa mPEG-Ar-CHO titrations with absorbances obtained from UV-Vis traces. Replicate 3.

Table S18. Summary of K_a and fitting error of MeHz/2kDa mPEG-Ar-CHO from the BindFit using

Replicate 1		Replicate 2		Replicate 3	
K_a (M^{-1})	Fitting error (%)	K_a (M^{-1})	Fitting error (%)	K_a (M^{-1})	Fitting error (%)
505.82	± 3.42	453.34	± 4.91	576.51	± 4.87

Average K_a = 512 ± 61.8 M^{-1} with average fitting error ± 4.40 %

λ = 298-305 nm.

8.2. UV-Vis Titration for Hydrazone Crosslink K_a Determination

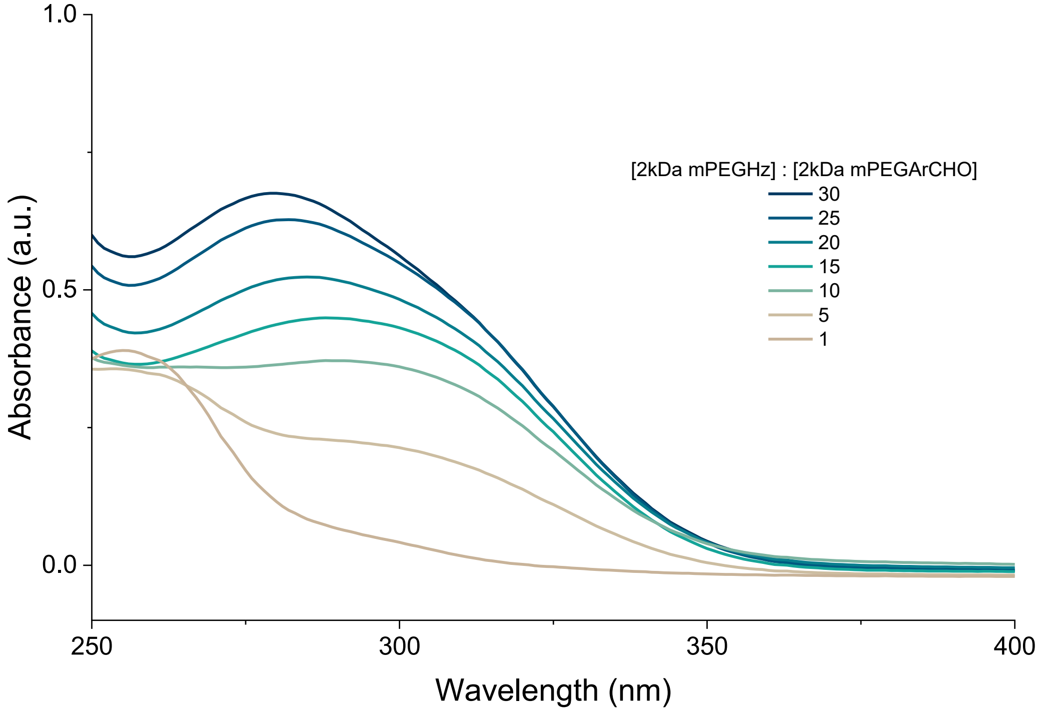

Figure S60. UV-Vis traces stacked in an overlay of all ratios of 2kDa mPEG-Hz to 2kDa mPEG-Ar-CHO in PBS. Replicate 1.

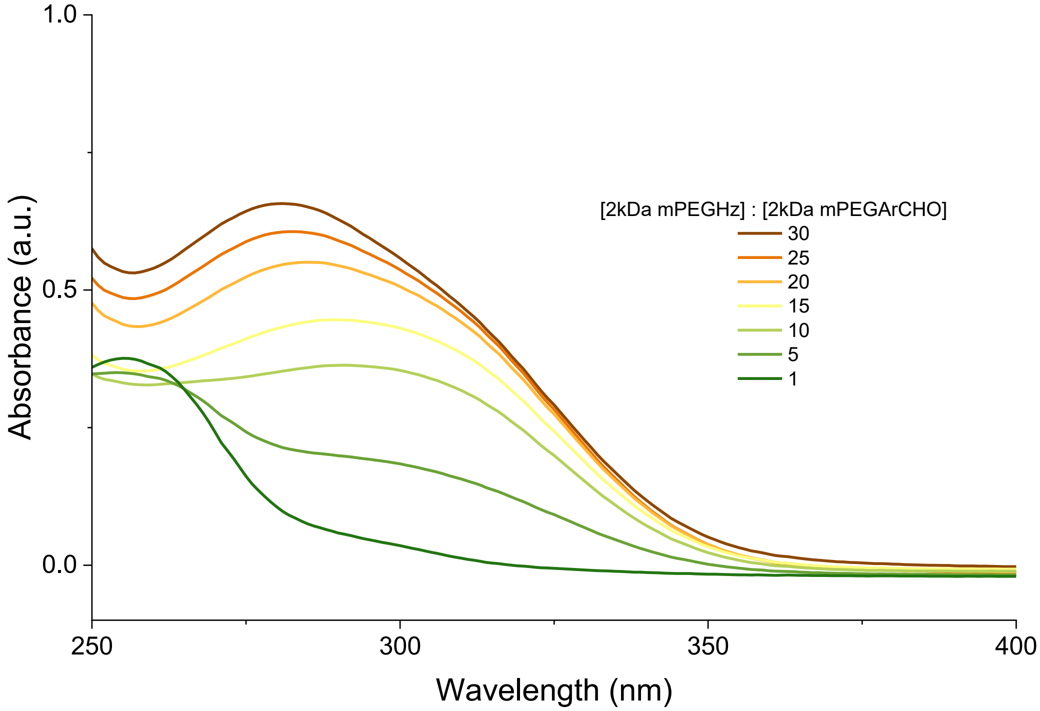

Figure S61. UV-Vis traces stacked in an overlay of all ratios of 2kDa mPEG-Hz to 2kDa mPEG-Ar-CHO in PBS. Replicate 2.

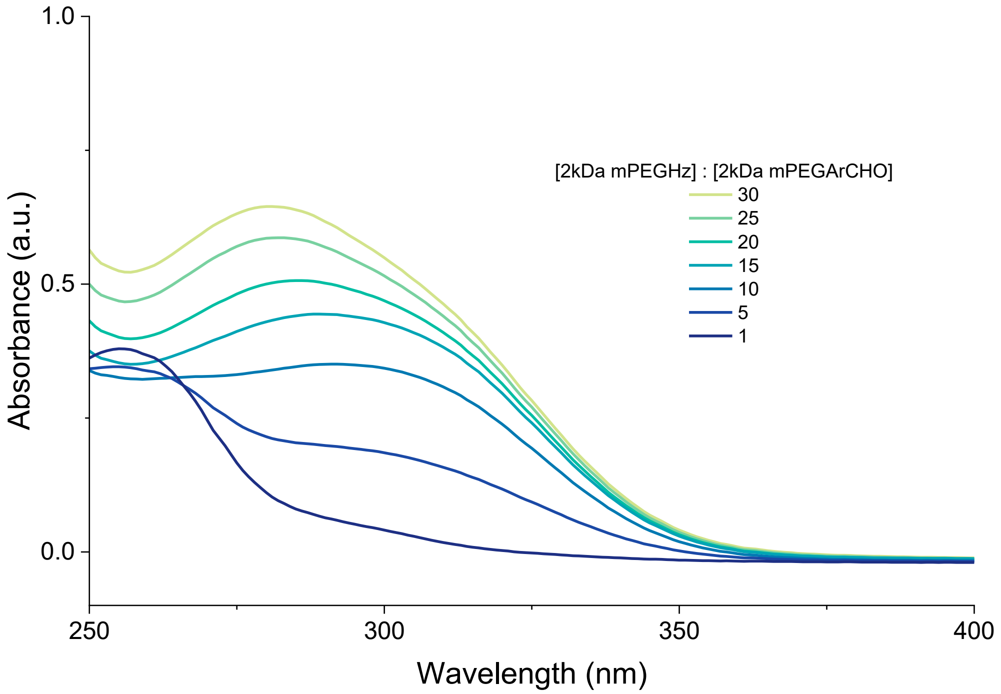

Figure S62. UV-Vis traces stacked in an overlay of all ratios of 2kDa mPEG-Hz to 2kDa mPEG-Ar-CHO in PBS. Replicate 3.

Table S19. Raw λ_{max} absorbance data for each titration.

Ratio ([2kDa mPEG-Hz]:[2kDa mPEG-Ar-CHO])	Absorbance at λ_{max} (302 nm)		
	Replicate 1	Replicate 2	Replicate 3
1:1	0.036	0.030	0.036
5:1	0.209	0.179	0.181
10:1	0.355	0.349	0.338
15:1	0.424	0.424	0.422
20:1	0.473	0.495	0.459
25:1	0.534	0.523	0.502
30:1	0.545	0.541	0.533

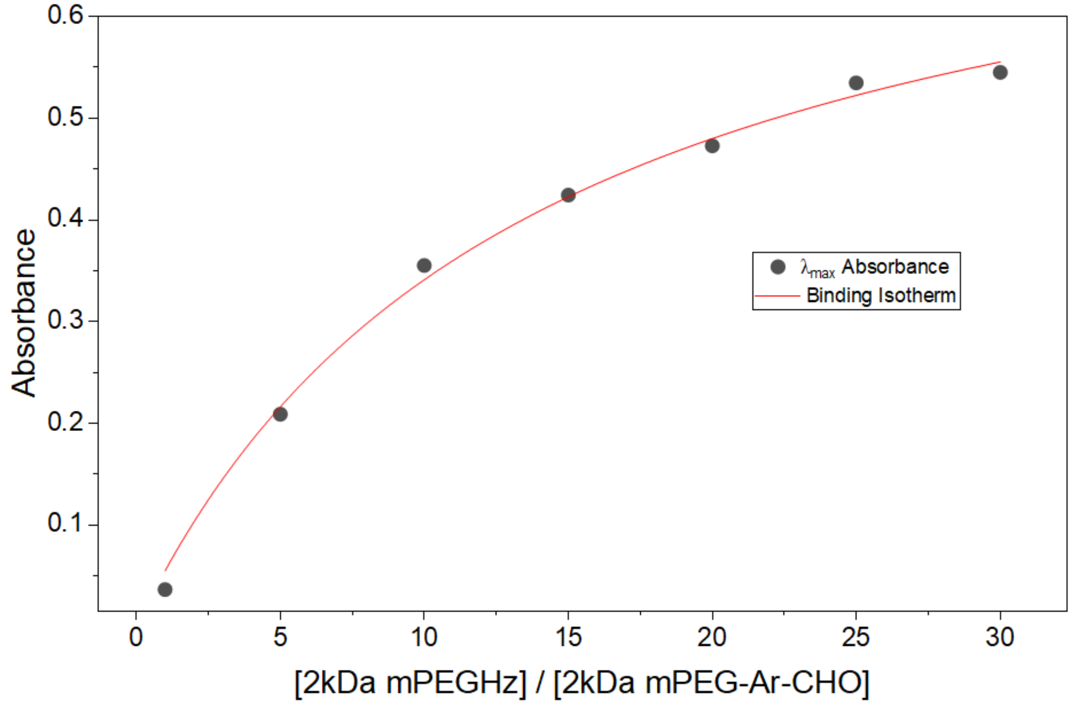

Figure S63. Binding isotherms of all 2kDa mPEG-Hz/2kDa mPEG-Ar-CHO titrations obtained from UV-Vis traces. Replicate 1.

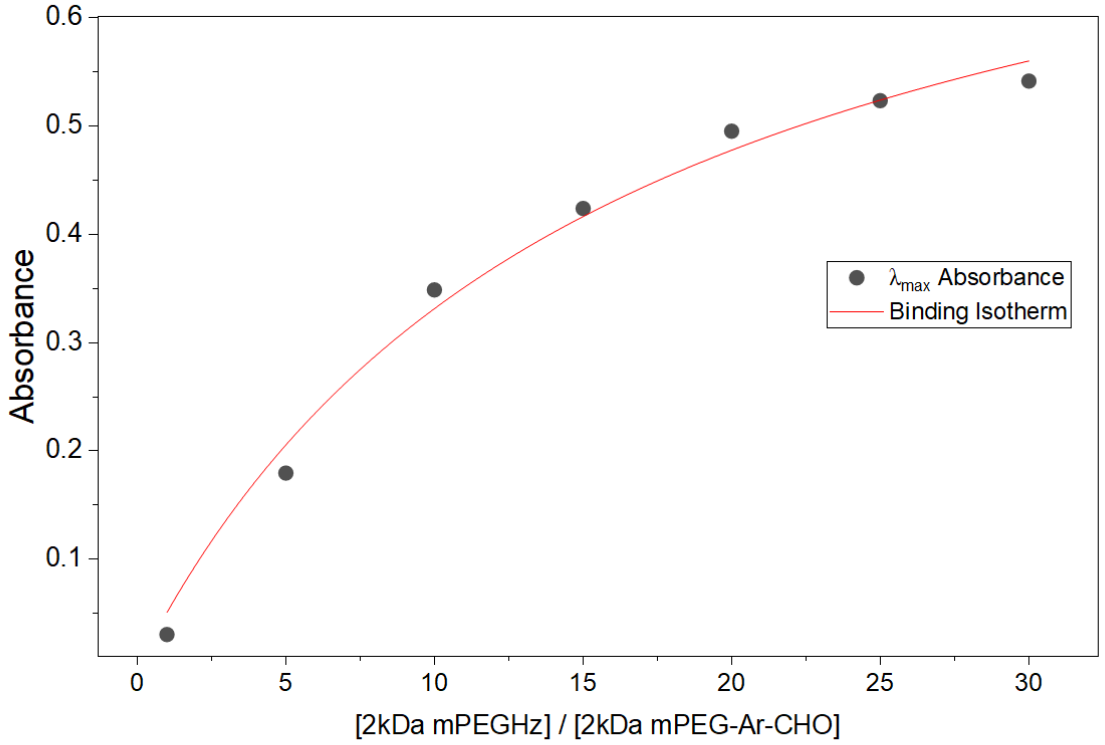

Figure S64. Binding isotherms of all 2kDa mPEG-Hz/2kDa mPEG-Ar-CHO titrations with absorbances obtained from UV-Vis traces. Replicate 2.

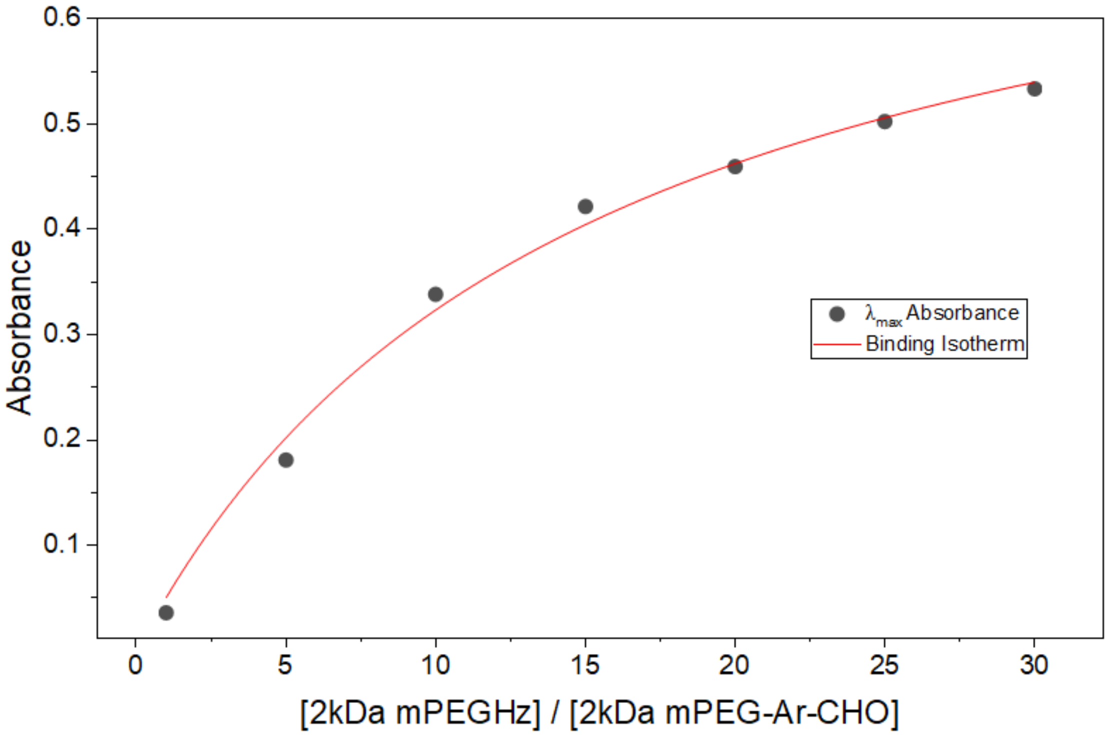

Figure S65. Binding isotherms of all 2kDa mPEG-Hz/2kDa mPEG-Ar-CHO titrations with absorbances obtained from UV-Vis traces. Replicate 3.

Table S20. Summary of K_a and fitting error of 2kDa mPEG-Hz/2kDa mPEG-Ar-CHO from the BindFit using λ = 298-305 nm.

Replicate 1		Replicate 2		Replicate 3	
K_a (M^{-1})	Fitting error (%)	K_a (M^{-1})	Fitting error (%)	K_a (M^{-1})	Fitting error (%)
1474	± 2.22	1235	± 3.11	1328	± 2.52

Average K_a = 1346 ± 120 M^{-1} with average fitting error ± 2.62 %

9. Rheology Values for Hydrazone Gels

Table S21. Values of the rheology represented in figure 5.

[MeHz] mM	Replicate	Modulus (kPa)	τ maxwell (s)	τ fit KWW (s)	β fit	<τ> (s)
0	1	17.5	526	539	0.924	559
	2	16.9	687	729	0.843	797
	3	14.3	664	685	0.912	716
	Avg	16.2	626	651	0.893	691
	Stdev	1.71	87	99	0.044	121
27	1	5.63	390	397	0.941	408
	2	5.47	457	476	0.876	509
	3	5.80	552	561	0.951	574
	Avg	5.63	466	478	0.923	497
	Stdev	0.161	81	82	0.041	83
63	1	2.64	368	379	0.912	396
	2	2.86	521	538	0.912	562
	3	2.25	474	482	0.950	493
	Avg	2.58	454	466	0.925	484
	Stdev	0.309	78	81	0.022	83
81	1	1.81	275	255	0.735	309
	2	2.80	222	171	0.570	276
	3	1.54	255	227	0.669	301
	Avg	2.05	251	218	0.658	295
	Stdev	0.661	27	43	0.083	17

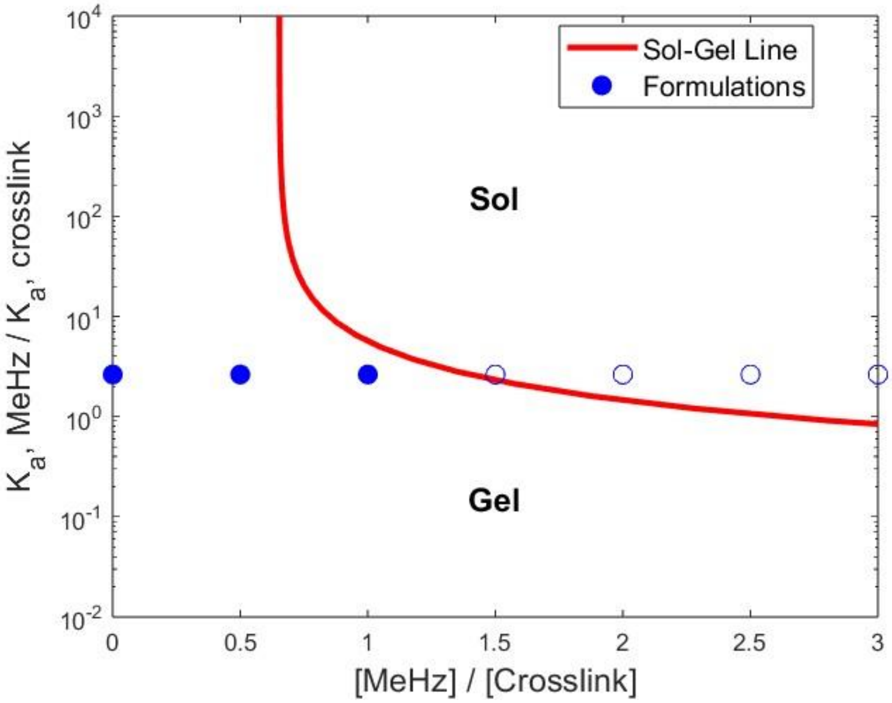

Figure S66. Plot of K_a MeHz / $K_{a,XL}$ of the 4PEG-hydrazone system vs. [MeHz] / [Crosslink]. Solid dots represent formulations that made gels, where open dots represent formulations that did not gel. Phase diagram was calculated based on equations from Heilshorn *et. al.*[24]

10. Swelling Test

The benzyl-hydrazone PEG-based hydrogel 9% w/v (total volume 200 µL) was submerged in PBS 1X 20 mL for 2 days. The swelling ratio is calculated by the equation below:

$$\text{Swelling ratio} = \frac{W_s - W_d}{W_d}$$

where W_s and W_d are the hydrogel weights after swelling and in the dry state, respectively.

The volume fraction ($\boldsymbol{\phi}_s$) of the hydrogel is calculated via the following equation:

$$\boldsymbol{\phi}_s = \left[1 + \frac{\rho_p}{\rho_w}\left(\frac{W_s}{W_d} - 1\right)\right]^{-1}$$

where ρ_p and ρ_w are assumed as the densities of PEG (1.125 g/mL) and water (1.000 g/mL), and W_s and W_d are the hydrogel weights after swelling and in the dry state, respectively.[25]

After 2 days, the PBS was replaced with fresh PBS buffer for another 1 day. Aliquots of PBS after 2 days and fresh PBS after another 1 day were taken for UV-Vis spectroscopy.

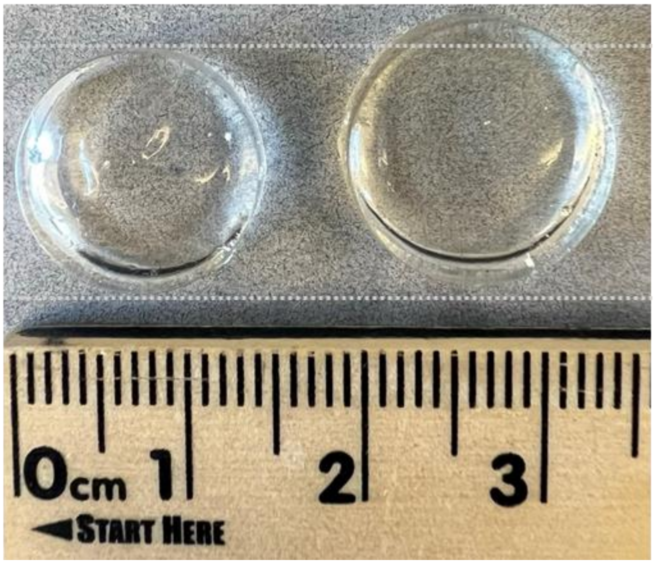

Figure S67. Size comparison of the swollen hydrogel without competitor (left) and with 63 mM MeHz (right).

Table S22. The swelling ratio and volume fraction of the hydrogel with and without MeHz competitor.

w/o or w/ competitor	Swelling ratio	Volume fraction; $\boldsymbol{\phi}_s$
w/o competitor	5.48 ±0.85	0.162 ± 0.034
with 63 mM MeHz	8.15±1.06	0.112 ± 0.014

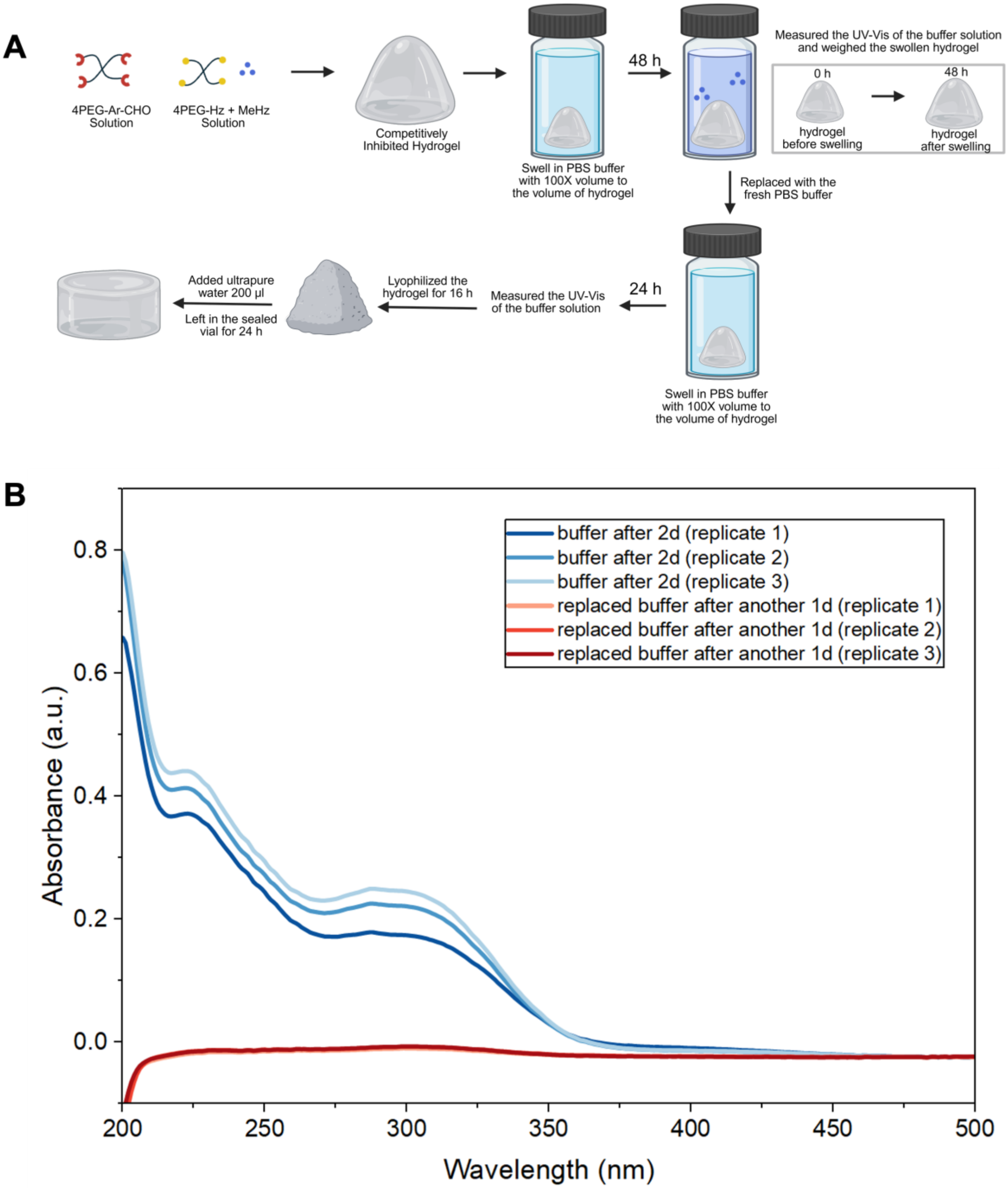

Figure S68. (A) Schematic detailing the swelling, washing, and rehydration of process of hydrazone-crosslinked gels. **(B)** The UV-Vis absorbance of PBS buffer after washing and after replacing with fresh buffer.

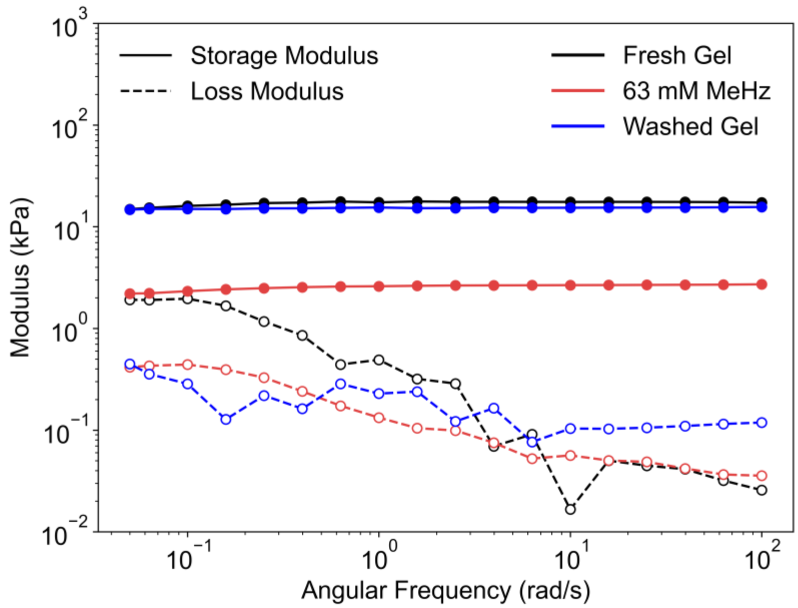

Figure S69. Frequency-dependent storage (solid markers) and loss modulus (open markers) from 0.05 rad/s – 100 rad/s, 1% strain of hydrazone-crosslinked gels after initial preparation with no competitor (black), with 63 mM of competitor (red), and after removing competitor (blue). Conducted at 25 °C.

11. Self-healing Test

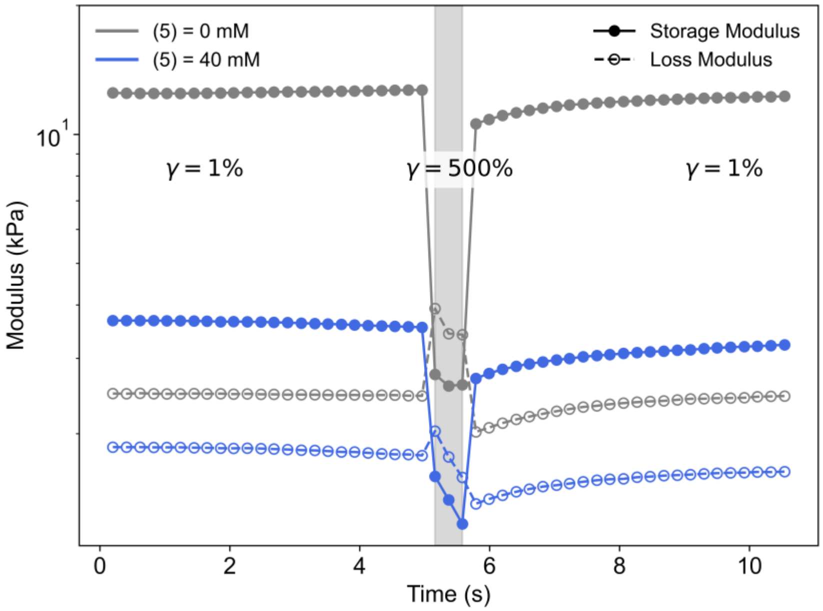

Figure S70. Step-strain measurement of boronate ester gels with and without competitor showing similar recovery of modulus at 10 rad/s going from low strain (1%) to high strain (500%) back to 1% strain. Open dots represent loss modulus and closed dots represent storage modulus.

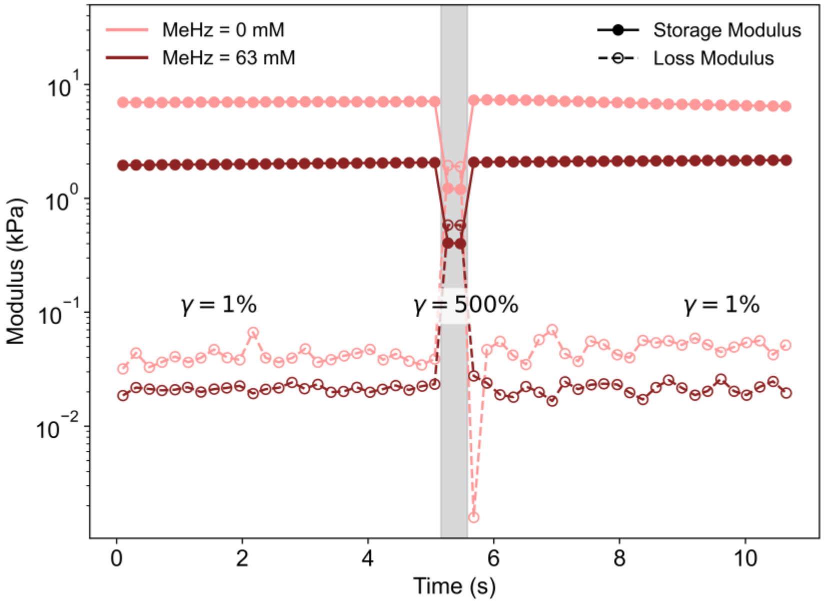

Figure S71. Step strain measurement of hydrazone gels with and without competitor showing similar recovery of modulus at 10 rad/s going from low strain (1%) strain to high strain (500% strain) back to 1% strain.

References

(1) Palmer, T.; Bonner, P. L. Enzyme Inhibition. In *Enzymes*; Elsevier, 2011; pp 126–152. https://doi.org/10.1533/9780857099921.2.126.

(2) Parada, G. A.; Zhao, X. Ideal Reversible Polymer Networks. *Soft Matter* **2018**, *14* (25), 5186–5196. https://doi.org/10.1039/C8SM00646F.

(3) Marco-Dufort, B.; Iten, R.; Tibbitt, M. W. Linking Molecular Behavior to Macroscopic Properties in Ideal Dynamic Covalent Networks. *J. Am. Chem. Soc.* **2020**, *142* (36), 15371–15385. https://doi.org/10.1021/jacs.0c06192.

(4) Cai, P. C.; Krajina, B. A.; Spakowitz, A. J. Brachiation of a Polymer Chain in the Presence of a Dynamic Network. *Phys. Rev. E* **2020**, *102* (2), 020501. https://doi.org/10.1103/PhysRevE.102.020501.

(5) Cai, P. C.; Su, B.; Zou, L.; Webber, M. J.; Heilshorn, S. C.; Spakowitz, A. J. Rheological Characterization and Theoretical Modeling Establish Molecular Design Rules for Tailored Dynamically Associating Polymers. *ACS Cent. Sci.* **2022**, *8* (9), 1318–1327. https://doi.org/10.1021/acscentsci.2c00432.

(6) Bell, G. I. Models for the Specific Adhesion of Cells to Cells: A Theoretical Framework for Adhesion Mediated by Reversible Bonds between Cell Surface Molecules. *Science* **1978**, *200* (4342), 618–627. https://doi.org/10.1126/science.347575.

(7) Miller, D. R.; Macosko, C. W. A New Derivation of Post Gel Properties of Network Polymers. *Macromolecules* **1976**, *9* (2), 206–211. https://doi.org/10.1021/ma60050a004.

(8) Claiborne, Alexander D.; Hill, M. R. InhibNet, 2025. https://github.com/hill-lab-chem/InhibNet.

(9) Akagi, Y.; Gong, J. P.; Chung, U.; Sakai, T. Transition between Phantom and Affine Network Model Observed in Polymer Gels with Controlled Network Structure. *Macromolecules* **2013**, *46* (3), 1035–1040. https://doi.org/10.1021/ma302270a.

(10) Flory, P. J. Theory of Elasticity of Polymer Networks. The Effect of Local Constraints on Junctions. *J. Chem. Phys.* **1977**, *66* (12), 5720–5729. https://doi.org/10.1063/1.433846.

(11) Cho, S.; Hwang, S. Y.; Oh, D. X.; Park, J. Recent Progress in Self-Healing Polymers and Hydrogels Based on Reversible Dynamic B–O Bonds: Boronic/Boronate Esters, Borax, and Benzoxaborole. *J. Mater. Chem. A* **2021**, *9* (26), 14630–14655. https://doi.org/10.1039/D1TA02308J.

(12) Kang, B.; Kalow, J. A. Internal and External Catalysis in Boronic Ester Networks. *ACS Macro Lett.* **2022**, *11* (3), 394–401. https://doi.org/10.1021/acsmacrolett.2c00056.

(13) Xiang, Y.; Xian, S.; Ollier, R. C.; Yu, S.; Su, B.; Pramudya, I.; Webber, M. J. Diboronate Crosslinking: Introducing Glucose Specificity in Glucose-Responsive Dynamic-Covalent Networks. *J. Controlled Release* **2022**, *348*, 601–611. https://doi.org/10.1016/j.jconrel.2022.06.016.

(14) Richardson, B. M.; Walker, C. J.; Macdougall, L. J.; Hoye, J. W.; Randolph, M. A.; Bryant, S. J.; Anseth, K. S. Viscoelasticity of Hydrazone Crosslinked Poly(Ethylene Glycol) Hydrogels Directs Chondrocyte Morphology during Mechanical Deformation. *Biomater. Sci.* **2020**, *8* (14), 3804–3811. https://doi.org/10.1039/D0BM00860E.

(15) McKinnon, D. D.; Domaille, D. W.; Cha, J. N.; Anseth, K. S. Biophysically Defined and Cytocompatible Covalently Adaptable Networks as Viscoelastic 3D Cell Culture Systems. *Adv. Mater.* **2014**, *26* (6), 865–872. https://doi.org/10.1002/adma.201303680.

(16) Burnouf, D.; Ennifar, E.; Guedich, S.; Puffer, B.; Hoffmann, G.; Bec, G.; Disdier, F.; Baltzinger, M.; Dumas, P. kinITC: A New Method for Obtaining Joint Thermodynamic and Kinetic Data by Isothermal Titration Calorimetry. *J. Am. Chem. Soc.* **2012**, *134* (1), 559–565. https://doi.org/10.1021/ja209057d.

(17) Dumas, P.; Ennifar, E.; Da Veiga, C.; Bec, G.; Palau, W.; Di Primo, C.; Piñeiro, A.; Sabin, J.; Muñoz, E.; Rial, J. Extending ITC to Kinetics with kinITC. In *Methods in*

Enzymology; Elsevier, 2016; Vol. 567, pp 157–180. https://doi.org/10.1016/bs.mie.2015.08.026.
(18) Springsteen, G.; Wang, B. A Detailed Examination of Boronic Acid–Diol Complexation. *Tetrahedron* **2002**, *58* (26), 5291–5300. https://doi.org/10.1016/S0040-4020(02)00489-1.
(19) Oglesby, P. L. *Mechanical Properties - Viscoelastic Methods : Progress Report*, 0 ed.; NBS RPT 10294; National Bureau of Standards: Gaithersburg, MD, 1970; p NBS RPT 10294. https://doi.org/10.6028/NBS.RPT.10294.
(20) Chen, Q.; Tudryn, G. J.; Colby, R. H. Ionomer Dynamics and the Sticky Rouse Model. *J. Rheol.* **2013**, *57* (5), 1441–1462. https://doi.org/10.1122/1.4818868.
(21) Tang, S.; Wang, M.; Olsen, B. D. Anomalous Self-Diffusion and Sticky Rouse Dynamics in Associative Protein Hydrogels. *J. Am. Chem. Soc.* **2015**, *137* (11), 3946–3957. https://doi.org/10.1021/jacs.5b00722.
(22) Bindfit. http://supramolecular.org.
(23) Brynn Hibbert, D.; Thordarson, P. The Death of the Job Plot, Transparency, Open Science and Online Tools, Uncertainty Estimation Methods and Other Developments in Supramolecular Chemistry Data Analysis. *Chem. Commun.* **2016**, *52* (87), 12792–12805. https://doi.org/10.1039/C6CC03888C.
(24) Gilchrist, A. E.; Liu, Y.; Klett, K.; Liu, Y.-C.; Ceva, S.; Heilshorn, S. C. Transient Competitors to Modulate Dynamic Covalent Cross-Linking of Recombinant Hydrogels. *Chem. Mater.* **2023**, *35* (21), 8969–8983. https://doi.org/10.1021/acs.chemmater.3c01575.
(25) Richbourg, N. R.; Wancura, M.; Gilchrist, A. E.; Toubbeh, S.; Harley, B. A. C.; Cosgriff-Hernandez, E.; Peppas, N. A. Precise Control of Synthetic Hydrogel Network Structure via Linear, Independent Synthesis-Swelling Relationships. *Sci. Adv.* **2021**, *7* (7), eabe3245. https://doi.org/10.1126/sciadv.abe3245.
