# Supplementary material for: Competitive Inhibition as a Tool to Modulate and Predict Dynamic Hydrogel Mechanics
Source: ACS Cent Sci. 2026 Feb 17;12(2):233–42. doi: 10.1021/acscentsci.5c02130 (PMC12947549; doi:10.1021/acscentsci.5c02130)
Supplement: Supplementary file 3 [file oc5c02130_si_003.pdf]

Supporting Information for

Predicting and Tuning the Mechanics of Dynamic Hydrogels with Competitive Inhibition

Alexander D. Claiborne [1], Sirilak Mekcham [1], Owen A. Lee [1], Megan R. Hill [1]
Department of Chemistry, Colorado State University, 301 W Pitkin, Fort Collins, CO 80521-1872, United States

Table of Contents

Discussion **2**

Equilibrium Constants Under Competitive Inhibition: *2*

Rubber Elasticity Models for Competitively Inhibited Gels: *4*

Design Space Available in Model: *7*

Model Assumptions: *8*

Instrumentation, General Procedures, and Materials **9**

Synthetic Methods **11**

Synthesis of 5 kDa 4PEG-GA: *11*

Synthesis of 2 kDa mPEG-GA: *11*

Synthesis of (2R,3S,4R,5R)-2,3,4,5,6-pentahydroxy-N-(2-methoxyethyl)hexanamide (Cl-ana): *11*

Synthesis of 5 kDa 4PEG-3-fluorophenylboronic acid: *12*

Synthesis of 2 kDa mPEG-3-fluorophenylboronic acid: *12*

Synthesis of 5 kDa 4PEG-benzyl aldehyde (5kDa 4PEG-Ar-CHO): *13*

Synthesis of 2 kDa mPEG-benzyl aldehyde (2kDa mPEG-Ar-CHO): *13*

Synthesis of 5 kDa 4PEG-hydrazine (5kDa 4PEG-Hz): *14*

Synthesis of 2 kDa mPEG-hydrazine (2kDa mPEG-Hz): *15*

ITC **24**

ITC Procedures *24*

Glucose (1) *25*

Dyphylline (2) *29*

Tris (3) *32*

Capecitabine (4) *35*

mPEG-GA *38*

Cl-ana (5) *41*

Dopamine (6) *44*

ITC Data Summary *47*

Rheology **48**

Formation of Hydrogels: ... 48
Zero Competitor ... 49
Glucose (1) ... 50
Dyphylline (2) ... 51
Tris (3) ... 52
Capecitabine (4) ... 53
Crosslink-analogue (cl-ana) (5) ... 54
Dopamine (6) ... 55
Addition of Competitive Inhibitor Post-Gelation: ... 56
Modulus Predictions ... 58
Predicting Modulus From K_a crosslink and K_a of competitor ... 58
Fitting for $K_{a,XL}$ from known $K_{a,C}$ ... 59
Fitting for $K_{a,C}$ from known $K_{a,XL}$ ... 60
Representative Stress Relaxation Fits ... 61
Tau Predictions ... 62
UV-Vis Titration for MeHz K_a determination ... 65
Rheology values for Hydrazone Gels ... 74
Swelling Test ... 75
Self-healing Test ... 78
References ... 79

Discussion

Equilibrium Constants Under Competitive Inhibition:

To model the competitive inhibition of a crosslink, we used assumptions from Michaelis Menton kinetics. This section summarizes our assumptions for determining the equilibrium constant of a crosslink under competition.

Found below are the derived expressions, all of which are based on competing, dynamic polymer networks where $A + B \leftrightarrow N_{XL}$ is the crosslinking reaction and $A + C \leftrightarrow AC$ is the competitor (C) reacting with half of the crosslink (A or B). The concentration of the active crosslinks, N_{XL}, can be solved for from the association constant eq. (S1),

$$K_{a,XL} = \frac{N_{XL}}{[A][B]} = \frac{k_{on}}{k_{off}} \text{ (S1)}$$

$$N_{XL} = K_{a,XL}[A][B] \text{ (S2)}$$

And the equilibrium constants and concentration of interaction for the competing interaction similarly can be defined as:

$$[AC] = K_{a,C}[A][C] \quad (S3)$$

The total concentration of A can be expressed by:

$$[A_{total}] = [A] + N_{XL} + [AC] \quad (S4)$$

Substituting eq (S2) and eq (S3) into eq (S4) and simpifying gives:

$$[A] = \frac{[A_{total}]}{1+K_{a,XL}[B]+K_{a,C}[C]} \quad (S5)$$

By substituting free crosslink concentration [A] into eq (S2), the concentration of active crosslink N_{XL} is defined by the K values and concentrations of each species:

$$N_{XL} = \frac{K_{a,XL}[A_{total}][B]}{1+K_{a,XL}[B]+K_{a,C}[C]} \quad (S6)$$

However, eq (S6) is difficult to use practically as instantaneously one need to know the concentration of every bound and unbound species in the system. To adjust for this, several assumptions were taken from chemical biology.

Utilizing how chemical biologists consider competitive inhibition equilibrium, we created a framework for assuming how the equilibrium constant changes. A key assumption in Michaelis Menten kinetics is that the Michaelis constant (K_m), which reflects the substrate concentration required to reach half of the maximum reaction rate, and thus serves as a measure of enzyme-substrate affinity, changes as a function of concentration of competitor ($[C]_0$) and the equilibrium constant of the competitor with the crosslink $K_{a,C}$ (expressed as the dissociation equilibrium, K_d, rather than the association equilibrium used here where $K_a = 1/K_d$).[1] The apparent change in K_m or the dissociation of an enzyme to a substrate, as a function competitor concentration and affinity is shown in eq (S7).

$$K_m^{app} = K_m\left(1 + \frac{[C]_0}{K_{a,C}}\right) \quad (S7)$$

In enzyme binding an enzyme binds with a substrate, the substrate is then transformed into a product, as seen in eq (S8)

$$S + E \underset{k_{off}}{\overset{k_{on}}{\rightleftarrows}} ES \xrightarrow{k_{cat}} E + P \qquad (S8)$$

Where S is the substrate, E is the enzyme and P is the product. The values k_{on} and k_{off} are the rates of association and dissociation of the complex, and k_{cat} is the rate of formation of product. The Michaelis constant can be defined by $K_m = \frac{k_{off}+k_{cat}}{k_{on}}$, however, in dynamic networks no product is being formed so the variable k_{cat} is removed and $K_m = \frac{k_{off}}{k_{on}} = K_d$. So, to utilize this relationship, K_m must be replaced with the dissociative equilibrium constant, K_d where, $K_d = \frac{[A][B]}{N_{XL}} = \frac{k_{off}}{k_{on}}$. The K_d is then replaced the associative equilibrium constant as $K_A = 1/K_d$ in eq (9)

$$\frac{1}{K_{a,app}} = \frac{1}{K_{a,XL}}\left(1 + K_{a,C}[C]_0\right) \quad (S9)$$

So, if the inverse of both sides is taken and we simplify, to get,

$$K_{a,app} = \frac{K_{a,XL}}{1+K_{a,C}[C]_0} \qquad \text{(S10)}$$

In Eq (S10) all that is needed to estimate how changes in $K_{a,XL}$ with competitor is eq (S1) $K_{a,XL}$ without competitor, eq (S2) $K_{a,C}$ and eq (S3) the initial concentration of the competitor, $[C]_0$. Most importantly, we do not need to know the instantaneous fraction of bound or unbound crosslinks, which is a function of $K_{a,XL}$.

Rubber Elasticity Models for Competitively Inhibited Gels:

To predict Gp, a variety of theoretical approaches have been reported. For example, Spakowitz *et. al,* developed a brachiation model in which network mechanics emerge from stochastic association and dissociation events along polymer chains, with bound-state statistics treated using a Poisson framework.[2,3] In parallel, Zhao *et. al* developed a mean-field, Bell-type description for reversible ideal polymer networks that links molecular association equilibria to macroscopic viscoelastic response.[4,5]

In this work, we adopt the Zhao framework as an initial approximation because it provides a direct, analytically tractable connection between equilibrium binding parameters and macroscopic mechanics, making it well-suited for incorporating $K_{a,app}$, introduced here. The competitive inhibition formalism introduced here is general, and we anticipate that $K_{a,app}$ can be applied to a variety of network elasticity models. In this work, we demonstrate its implementation for ideal polymer networks assuming either affine or phantom elasticity.

With a method to calculate $K_{a,app}$ in hand, we then sought to utilize this value in rubber elasticity models developed previously.[4,6] The shear modulus of a polymer network (G_p, relating to stiffness or network "strength") is defined by:

$$G_p = \upsilon_e k_B T \quad \text{(S11)}$$

for the affine network model (applicable to crosslink dense systems) or

$$G_p = (\upsilon_e - \mu) k_B T \quad \text{(S12)}$$

for the phantom network model (applicable to dilute systems lose to the overlap concentration like this work). Where υ_e is the density of elastically active chains, which refer to chains that have three or more crosslinks to the network, and μ is the concentration of cross-links. To calculate these values for any ideal, reversible polymer network, the conversion of crosslinks must first be estimated. Since the crosslinks exist in an equilibrium, the conversion (*p*) is dependent on the equilibrium constant, $K_{a,XL}$, and the concentration of the reactive chain-ends N_{XL}.[4,7] The overall conversion of the crosslinks with no competitor (p_0) is therefore defined by:

$$p_0 = \left(1 + \frac{1}{2N_{XL}K_{a,XL}}\right) - \left[\left(1 + \frac{1}{2N_{XL}K_{a,XL}}\right)^2 - 1\right]^{1/2} \quad \text{(S13)}$$

To determine the conversion of crosslinks as a function of inhibitor we can replace $K_{a,XL}$ with $K_{a,app}$ to predict how the conversion of crosslinks will change as a function of competitor concentration and binding to the crosslink.

$$p = \left(1 + \frac{1}{2N_{XL}K_{a,app}}\right) - \left[\left(1 + \frac{1}{2N_{XL}K_{a,app}}\right)^2 - 1\right]^{1/2} \quad \text{(S14)}$$

For an ideal network employing a tetra-arm macromer, υ_e can then be related to conversion (p) through the probability of the macromers to make three or four crosslinks within the network (thereby contributing to network elasticity). Thus, we can calculate the probability that one arm leads to a dangling end P_{out} as a function of the conversion of crosslinks:

$$P_{out} = \left(\frac{1}{p} - \frac{3}{4}\right)^{\frac{1}{2}} - \frac{1}{2} \qquad \text{(S15)}$$

which can be applied to a mean-field approximation to determine the probability of a three arm (P_3) or four arm (P_4) connections to the percolated network. Where a three arm junction represents a macromer connected to three distinct macromers, and a four arm connection is a macromer that is connected to four distinct macromers. As each connection is shared between two macromers, each crosslink contributes ½ elastically active chain, so P_3 contributes 1.5, and P_4 contributes 2. A P_2 connection contributes a loop, so it is not considered elastically active, and P_1 and P_0 connections represent dangling ends, and are not treated as elastically active.[8]

$$P_3 = 4P_{out}(1 - P_{out})^3 \quad \text{(S16)}$$

$$P_4 = (1 - P_{out})^4 \quad \text{(S17)}$$

Understanding that a three-arm junction contributes 1.5 elastically active chains, while a four-arm junction contributes 2 elastically active chains (i.e. each macromer arm is one half of the network strands that connect macromer junctions), we can write:

$$\mu = \frac{N_{XL}}{4}(P_3 + P_4) \qquad \text{(S18)}$$

$$\upsilon_e = \frac{N_{XL}}{4}\left(\frac{3}{2}P_3 + 2P_4\right) \quad \text{(S19)}$$

Substituting values derived from eq (s18) and eq (S19) into eq (S12) and simplifying (described in detail in reference 5) the shear modulus for a reversible ideal network utilizing the phantom network model can be predicted by eq (S20).

$$G_0/k_BT = \frac{N_{XL}}{16}\left(3 - \sqrt{\frac{4}{p} - 3}\right)^3 \left(\sqrt{\frac{4}{p} - 3} + 1\right) \quad \text{(S20)}$$

Utilizing equation (20) with $K_{a,app}$ determined via our Michaelis-Menton type approximation, we can predict how the modulus will change with competitor concentration and binding affinity a fit to the experimental modulus change is shown in figure 6. It is worth noting that the same procedure could be applied to the affine model (equation (11)), however given that the materials studied here are reasonably close to their overlap concentration (~10w/v %), we found that the phantom network model yielded better results. However, given that the phantom and affine network models represent two extremes of rubber elasticity, it is reasonable that the phantom chain model

underestimates some of the experimental data. To view these models as a tool we have published code in our GitHub repository, these models can be run as a web app where users can change different input parameters.[9]

Design Space Available in Model:

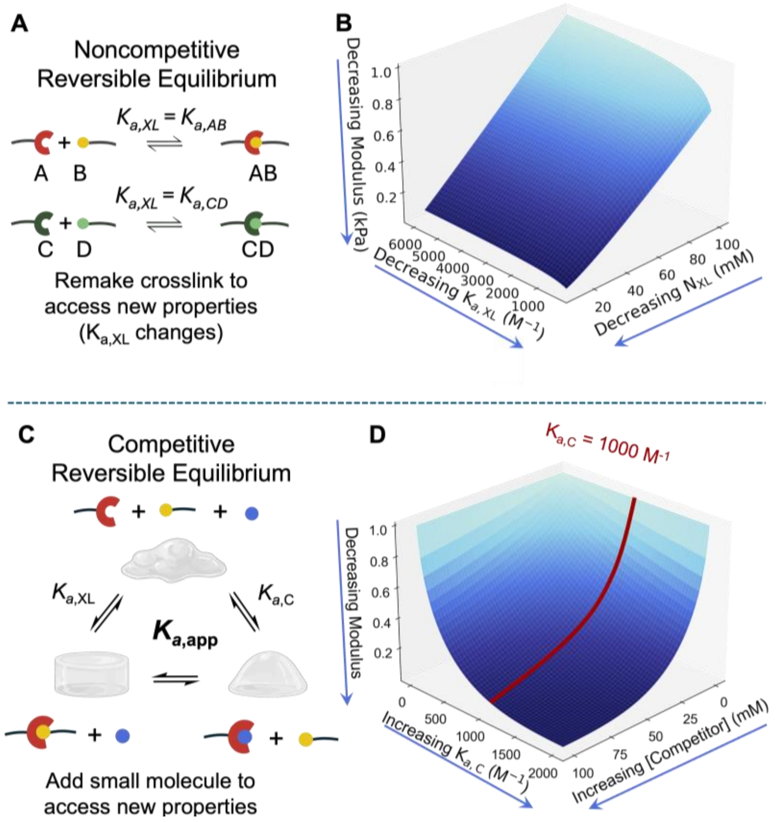

Figure S1: (A) In the noncompetitive case to change the material properties the concentration of crosslinks can be changed, or the crosslink can be remade to give different properties. Here is an example how two different sets of crosslinkers (AB and CD) give different crosslink association constants ($K_{a,XL}$) as shown in previous work.[4,6] (B) Visualization of the design space for a phantom network in the noncompetitive case, modulus can be modified by changing $K_{a,XL}$ or the concentration of the crosslink. Each slice of $K_{a,XL}$ represents a material that needs to be resynthesized. To change N_{XL} the material can just be prepared at different weight percents. (C) For the competitive reversible equilibrium case there is a ternary equilibrium between unbound, crosslink associated with the competitor and crosslink formation. We assume that the association of the crosslink is a function of both $K_{a,XL}$, $K_{a,C}$ and [C] (eq S1 – S10). (D) To visualize the design space in the noncompetitive case, modulus decreases as a function of increasing competitor association constant, $K_{a,C}$, and increasing concentration of competitor [C]. Note that in this case the crosslinking chemistry stays constant. To visualize how one competitor can change modulus, the red line indicates how a competitor with an association constant of 1000 M^{-1} changes the modulus.

Model Assumptions:

1. Affine and Phantom Network Models

We were inspired by this work for this section.[10] In this study we assume that these dilute gels undergo phantom network deformation which includes a mean field approximation of the gel, as shown in figure 2.[11] However, within our model on GitHub we inputted the option for changing the model to affine deformation instead, which would be better for a gel with a higher concentration of crosslinks.[12] However we do not capture behavior in-between the phantom and affine network, but we posit more complex network models could incorporate the $K_{a,app}$.

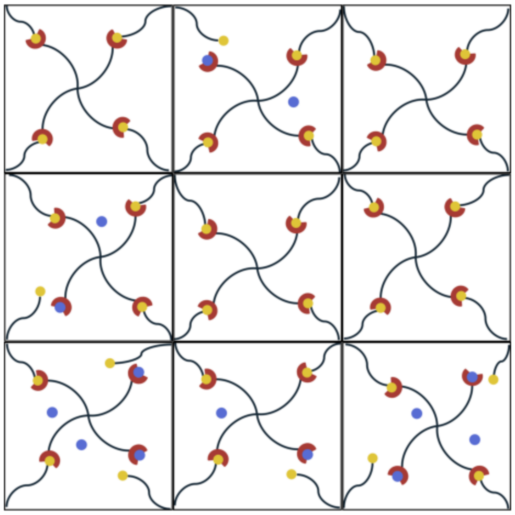

Figure S2: Scheme illustrating the mean field approximation for polymer network under competitive inhibition. The network is split into a mean unit (*e.g.* the unit within a single box). The mean field assumption for dynamic polymer networks calculates the probability of forming different numbers of junctions. We apply the mean field assumptions to the entire polymer network.

2. K_a is concentration independent

We assume that the association constants of both the crosslinks and the competitors remain unchanged across different concentrations. Although the concentrations used to measure crosslink association constants differ from those present in the gel, the measured values still apply reliably to the gel environment.

3. mPEG binding approximates 4PEG binding

This study uses functionalized mPEG with crosslink functionalities to study the binding of crosslinks and competitors. This assumes that this is a good approximation of how the individual strands of 4PEG macromers bind.

Instrumentation, General Procedures, and Materials

Instrumentation

Proton Nuclear Magnetic Resonance (^{1}H-NMR) spectra and Fluorine Nuclear Magnetic Resonance (^{19}F-NMR) spectra were acquired at 25 °C on a Bruker US400 (400 MHz) NMR instrument. Matrix Assisted Laser Desorption/Ionization Time-of-Flight Mass Spectrometry (MALDI-TOF MS) spectra were acquired on a Bruker UltrafleXtreme MALDI-TOF instrument. Rheology spectra were acquired on a Discovery Hybrid Rheometer 20 at 25 °C with either 8 mm or 20 mm parallel plates with a gap height set at 100 mm for the 8 mm plate and 300 mm for the 20 mm plate. Isothermal Titration Calorimetry (ITC) spectra were acquired on a TA Instruments Affinity ITC at 25 °C, with 30 injections of 2 μL. UV-Vis Spectrophotometer (UV/Vis) spectra were acquired on a Cary 3500 Multicell UV-Vis Spectrophotometer. Scans were acquired from 200.00 - 800.00 nm with an average time of 0.020 s. An Instron 68TM-10 Mechanical Tester was used to measure the injectability profiles. The Instron was equipped with a 500 N load cell with 1 kN pneumatic grips with serrated jaw faces. For the samples containing competitors there is an onset period before the force increases. This onset period arises from the initial compression of the gel within the syringe prior to flow through the needle. Once the gel is fully compressed and injection begins, the force reaches a plateau corresponding to the steady-state force required to inject the gel.

General MALDI procedure:

A 20 mg/mL solution of the polymer product was prepared in THF. A 60 mg/mL solution of DCTB in THF was prepared as a matrix solution. A 15 mg/mL solution of NaTFA in THF was used as the salt solution. To prepare the sample on the plate, 1 µL of sample solution, 20 µL of matrix solution, and 1 µL of salt solution was used. The instrument was set to reflector mode; 1000 shots were fired at a time at 200 Hz.

General rheology procedure:

Boronate Ester Crosslinked Gels

10 w/v % 4arm-PEG-FPBA and 10 w/v % 4arm-PEG-GA were prepared by using 10x HEPES buffer. To prepare the samples 100 µL of each PEG solution were combined in a vial. Gels would form within 3 seconds. The samples were then placed on an 8 mm or 20 mm parallel plate geometry. The trim gap was set to 130 mm for 8 mm plates or 330 for 20 mm plates. Excess gel was trimmed, and the geometry was set to 100 mm for the 8 mm plate and 300 mm for the 20 mm plate. Following this mineral oil was applied around the sides of the plates to limit evaporation. The instrument was set to 25 °C. To ensure thermodynamic equilibrium a time sweep was recorded at 10 rad/s, 1% strain, for 300 s. If the modulus of the gel was consistent a frequency sweep was collected from 100 rad/s to 0.1 rad/s at 1% strain. For the stress relaxation experiments, 5% strain steps were measured over 1000 s.

For the rheology experiments, we ensured the material was in equilibrium by initially performing a time sweep at 10 rad/s at 1 % strain for 300 seconds at 25 ºC. Following the time sweep, we performed frequency sweeps from 100 rad/s to 0.1 rad/s at 1% strain, and 25 ºC, collecting 5 points per decade, at varying identify and concentration of competitor. Finally, a stress relaxation experiment was run at 1% strain for 1000 seconds.

Over longer timescales, the materials are bench-stable with or without competitor, remaining in the gel phase for at least six months after formation. However, when placed in excess water, the hydrogels are not water-stable and dissolve within 24 h, consistent with previous work.[13] As a result, reversibility upon competitor removal by dilution is limited under these conditions. However, this behavior may be advantageous in contexts where triggered dissolution or clearance is

desired, such as drug delivery or injectable applications. Long-term stability would require additional network stabilization strategies.

Hydrazone Crosslinked gels

The hydrogels were allowed to set at ambient temperature for at least 12 h. Experiments were performed at a gap of 0.3 mm. The Oscillation time experiment was run at 25 ºC, 1% strain, Angular frequency 10.0 rad/s for 300 s, and a frequency sweep experiment was then performed from 0.1 to 100 rad/s with 1% strain at 25 ºC. After that stress relaxation experiment was performed with 10% strain at 37 ºC for 2000 s.

Instron injectability test:

10 w/v % 4arm-PEG-FPBA and 10 w/v % 4arm-PEG-GA with varying amounts of competitor were prepared by using 10x HEPES buffer. To prepare the samples 100 µL of each PEG solution were combined in a vial. Gels would form within 3 seconds. The gels were top loaded into 1 mL Air-Tite 2-Part Luer Lock Syringe with a PrecisionGlide 21G x 1" TW needle equipped. The grips were set to be 78 mm apart from each other. The grips were set to compress at a pressure of 15 psi, with one grip on the plunger of the syringe with the other on the screw of the Luer lock. After the syringe was secured in the center of the grips the compression test started at a rate of 0.05 mm/s. The test was set to end after a displacement of 5 mm occurred. The force vs displacement graphs were outputted to excel.

Materials:

Chemicals:

Triethylamine (TEA) – Fisher (121-44-8)
o-Benzotriazol-1-yl-tetramethyluronium hexafluorophosphate (HBTU) – Chem Impex Intl (94790-37-1)
1-Hydroxybenzotriazole hydrate (HOBt) – Sigma Aldrich (123333-53-9)
Glucono-delta-lactone – Chem Impex Intl (90-80-2)
Tetra-PEG-Amine (5kDa) – JenKem
(3-Formyloxyphenyl)boronic acid – TCI (25487-66-5)
Formylbenzoic acid- Aldrich Chemical (619-66-9)
4-Methylmorpholine- Alfa Aesar (109-02-4)
Tri-Boc-hydrazinoacetic acid- Chem Impex (261380-41-0)
(1-[Bis(dimethylamino)methylene]-1H-1,2,3-triazolo[4,5b]pyridinium3-oxidhexafluorophosphate) (HATU)- Sigma Aldrich (148893-10-1)
Methyl Hydrazine-Aldrich (60-34-4)
Dopamine hydrochloride – Thermo-scientific (62-31-7)
D(+) Glucose anhydrous – Thermo-scientific (50-99-7)
Dyphylline – Thermo-scientific (479-18-5)
Tris – Matrix Scientific (77-86-1)
Capecitabine – Sigma Aldrich (154361-50-9)
2-Methoxyethylamine – Sigma Aldrich (109-85-3)
4-(2-hydroxyethyl)-1-piperazineethanesulfonic acid (HEPES) – Sigma Aldrich (7365-45-9)
PBS Tablets – MP Biomedicals (N/A)
and were used as received unless otherwise noted.

Synthetic Methods

Synthesis of 5 kDa 4PEG-GA:

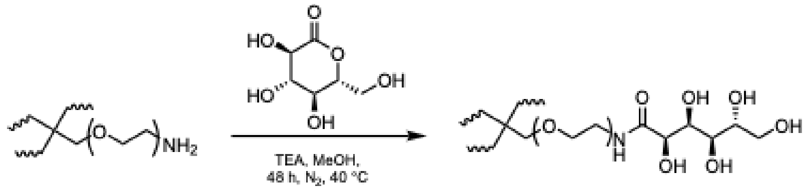

In a procedure adapted from Kang, and Kalow,[14] 5 kDa 4PEG-Amine HCl salt (1.000 g, 0.8000 mmol of amine, 1.000 eq), Glucono-delta-lactone (1.143 g, 0.8005 mmol, 8.020 eq), TEA (0.4946 g, 4.888 mmol, 6.11 eq) and a stir bar were placed in a 20 mL scintillation vial filled with 10 mL of methanol. The vial was heated and stirred at 40 °C for 48 hours under nitrogen. The reaction was then precipitated directly into 100 mL of -40 °C diethyl ether. The solution was filtered using a fine fritted funnel and dried under vacuum overnight. After seeing impurities in the ^{1}H NMR, the sample was redissolved in water and dialyzed against water for 3 days, with the water being changed twice a day. The sample was lyophilized to yield a stark white powder (1.012 g, 88.74 %). d = 7.46 (bs, 1H), 4.30 (s, 1H), 4.13 (s, 1H), 3.83 (s, 2H), 3.64 (brs, 114H), 1.25 (s, 1H).

Synthesis of 2 kDa mPEG-GA:

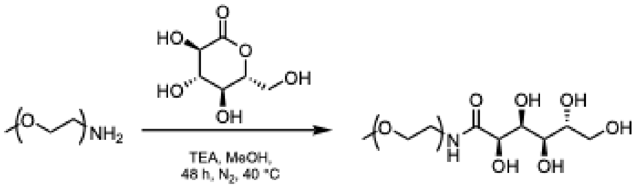

In a procedure adapted from Kang, and Kalow,[14] 2 kDa methoxyPEG-Amine HCl salt (0.5000 g, 0.2500 mmol of amine, 1.0 eq), Glucono-delta-lactone (0.1781 g, 0.9998 mmol, 3.999 eq), TEA (0.0761 g, 0.7525 mmol, 3.010 eq) and a stir bar were placed in a 20 mL scintillation vial filled with 10 mL of methanol. The vial was heated and stirred at 40 °C for 48 hours under nitrogen. The reaction was then precipitated directly into 100 mL of -40 °C diethyl ether. The solution was filtered using a fine fritted funnel and dried under vacuum overnight. After seeing impurities in the ^{1}H NMR, the sample was redissolved in water and dialyzed against water for 3 days, with the water being changed twice a day. The sample was lyophilized to yield a stark white powder (0.1834 g, 36.80%). d = 7.46 (bs, 1H), 4.30 (s, 1H), 4.13 (s, 1H), 3.83 (s, 2H), 3.64 (brs, 114H), 1.25 (s, 1H).

Synthesis of (2R,3S,4R,5R)-2,3,4,5,6-pentahydroxy-N-(2-methoxyethyl)hexanamide (CI-ana):

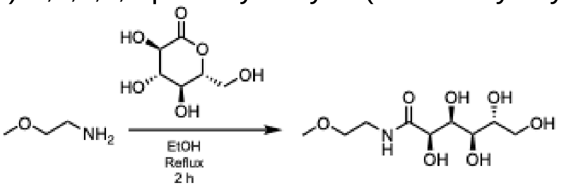

In a procedure adapted from Kang, and Kalow,[14] Glucono-delta-lactone (1 eq, 1.00 g, 5.6 mmol) was placed in a 100 mL round bottom flask. 2-Methoxyethylamine (1.1eq, 0.46 g, 6.2 mmol) was added dropwise to the flask. A condenser was attached to the round bottom flask and the ethanol was refluxed for 2 hours. Following this the ethanol was removed in vacuo. Following this the white powder was placed in a fine fritted funnel. The white powder was then washed with 10 mL of DCM 3 times. The white powder was then placed in a scintillation vial and dried overnight to yield the final product. The NMR was prepared in DMSO-D6 f(0.52 g, 36 %). ^{1}H-NMR d = 7.58 (t,

1H), 5.41 (d, 1H), 4.53 (brs, 1H), 4.47 (brs, 1H), 4.40 (d, 1H), 4.33 (t, 1H), 4.00(t, 1H), 3.90 (brs, 1H), 3.58 (brs, 1H), 3.47 (brs, 2H), 3.35 (m, 4H), 3.30 (t, 1H), 3.25 (s, 3H).

Synthesis of 5 kDa 4PEG-3-fluorophenylboronic acid:

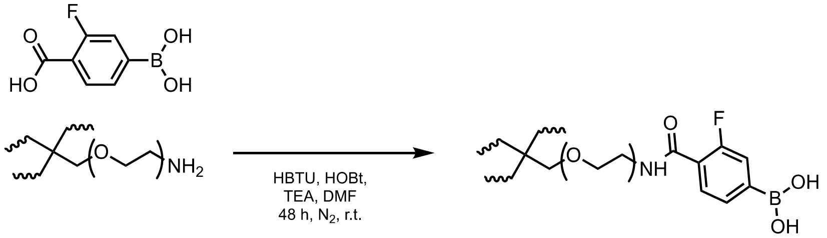

In a procedure adapted from Xiang et. al.,[15] 5kDa 4PEG-Amine (2.0 g, 0.4 mmol, 1.0 eq), HOBt (0.2450, 3.2 mmol, 8 eq), 4-Carboxy-3-flurophenylboronic acid (0.5886 g, 3.2 mmol, 8 eq) and TEA (0.446 mL, 3.200 mmol, 8.0 eq) were placed in a 100 mL flame dried round bottom flask and dissolved in 20 mL of DMF. The reaction mixture was then stirred for 5 minutes . Following this HBTU (1.2136 g, 3.2 mmol, 8 eq) was added. The reaction was stirred at room temperature for 48 hours under nitrogen. The reaction mixture was then dialyzed against MeOH for 1 day followed by dialysis against DI water for 2 days, with the solvent being changed being changed twice a day. The sample was lyophilized to yield a white powder. When the sample was prepared in DMSO-D6 gelation occurred, likely due to formation of a borazine crosslink under dry conditions, to analyze the sample 1 drop of DI water was added. (1.7478 g, 77.15 %). ^{1}H-NMR d = 8.39 (s, 2H), 8.27(brt, 1H), 7.53 – 7.26 (m, 4H), 3.64 (brs, 114H) ^{1}F-NMR d = 116.1 (s, 1F).

Synthesis of 2 kDa mPEG-3-fluorophenylboronic acid:

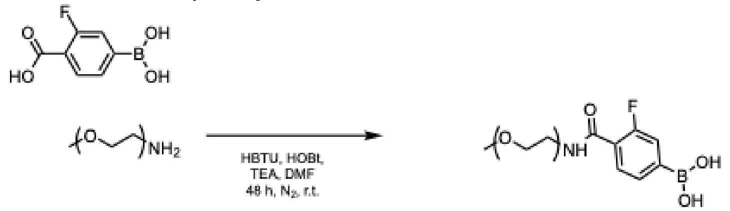

In a procedure adapted from Xiang et. al.,[15] 5kDa 4PEG-Amine (2.0 g, 0.4 mmol, 1.0 eq), HOBt (0.2450, 1.6 mmol, 8 eq), 4-Carboxy-3-flurophenylboronic acid (0.5886 g, 3.2 mmol, 8 eq) and TEA (0.446 mL, 3.200 mmol, 8.0 eq) were placed in a 100 mL flame dried round bottom flask and dissolved in 20 mL of DMF. The reaction mixture was then stirred for 5 minutes . Following this HBTU (1.2136 g, 3.2 mmol, 8 eq) was added. The reaction was stirred at room temperature for 48 hours under nitrogen. The reaction mixture was then dialyzed against MeOH for 1 day followed by dialysis against DI water for 2 days, with the solvent being changed being changed twice a day. The sample was lyophilized to yield a white powder (1.7478 g, 77.15 %). ^{1}H-NMR d = 8.39 (s, 2H), 8.27(brt, 1H), 7.53 – 7.26 (m, 4H), 3.64 (brs, 181H) ^{1}F-NMR d = 116.1 (s, 1F)

Synthesis of 5 kDa 4PEG-benzyl aldehyde (5kDa 4PEG-Ar-CHO):

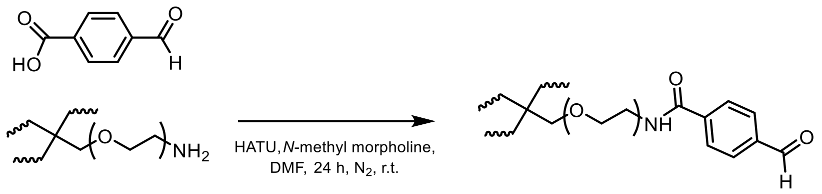

The benzyl PEG aldehyde was synthesized following the reported procedure.[16] 4-formylbenzoic acid (264 mg, 1.76 mmol, 2.20 eq. to NH_2 groups) was activated with HATU (1-[Bis(dimetylamino)methylene]-1H-1,2,3-triazolo[4,5-b]pyridinium 3-oxid hexafluorophosphate) (608 mg, 1.60 mmol, 2.00 eq. to NH_2 groups) and 4-methylmorpholine (0.440 mL, 4.00 mmol, 5.00 eq. to NH_2 groups) in dimethylformamide (DMF) 7.00 mL under N_2 for 10 min. In a separate vessel, 4-methylmorpholine (0.440 mL, 4.00 mmol, 5.00 eq. to NH_2 groups was added to 4-arm PEG-NH_2, HCl salt (M_n 5000 g/mol) (1.00 g, 0.200 mmol, 1.00 eq.) in DMF 3.00 mL. The 4-arm PEG-NH_2, HCl salt containing 4-methylmorpholine was slowly added to the first solution under N_2. The reaction was allowed to proceed at room temperature for 24 h. The reaction mixture was concentrated and precipitated in cold diethyl ether (-25 °C). The crude product was concentrated under reduced pressure, dissolved in DI water, transferred to regenerated cellulose membranes (Spectra/Por) with a molecular weight cut-off of 2,000 g/mol, and dialyzed against DI water for 2 days. The product was lyophilized to give an off-white powder (83 % yield, Figure S6). ^{1}H NMR (400 MHz, $CDCl_3$): δ= 10.1 (s, H, C*H*O), 8.12-7.88 (m, 4H, C_6H_4-), 3.83-3.41 (m, 113.5H, -O-CH_2-CH_2-O-).

Synthesis of 2 kDa mPEG-benzyl aldehyde (2kDa mPEG-Ar-CHO):

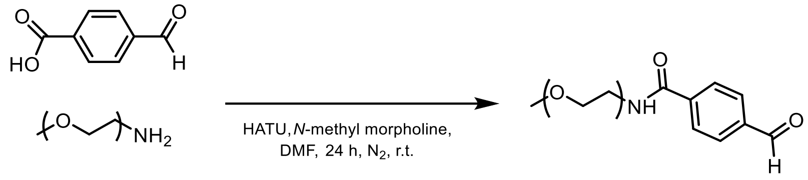

The (mono) functionalization of PEG with an aryl aldehyde was performed following the reported procedure.[16] 4-formylbenzoic acid (430 mg, 1.10 mmol, 2.20 eq. to NH_2 groups) was activated with HATU (1-[Bis(dimetylamino)methylene]-1H-1,2,3-triazolo[4,5-b]pyridinium 3-oxid hexafluorophosphate) (380 mg, 1.00 mmol, 2.00 eq. to NH_2 groups) and 4-methylmorpholine (0.270 mL, 2.50 mmol, 5.00 eq. to NH_2 groups) in dimethylformamide (DMF) 7.00 mL under N_2 for 10 min. In a separate vessel, 4-methylmorpholine (0.270 mL, 2.50 mmol, 5.00 eq. to NH_2 groups was added to poly(ethylene glycol) methyl ether amine (mPEG-NH_2) (M_n 2000 g/mol) (1.00 g, 0.500 mmol, 1.00 eq.) in DMF 5.00 mL. The mPEG-NH_2 containing 4-methylmorpholine was slowly added to the first solution under N_2. The reaction was allowed to proceed at room temperature for 24 h. The reaction mixture was concentrated and precipitated in cold diethyl ether (-25 °C). The crude product was concentrated under reduced pressure, dissolved in DI water, transferred to regenerated cellulose membranes (Spectra/Por) with a molecular weight cut-off of 2,000 g/mol, and dialyzed against DI water for 2 days. The product was lyophilized to give an off-white powder (75 % yield, Figure S7). ^{1}H NMR (400 MHz, $CDCl_3$): δ= 10.1 (s, H, C*H*O), 8.12-7.88 (m, 4H, C_6H_4-), 3.83-3.41 (m, 181H, -O-CH_2-CH_2-O-)

Synthesis of 5 kDa 4PEG-hydrazine (5kDa 4PEG-Hz):

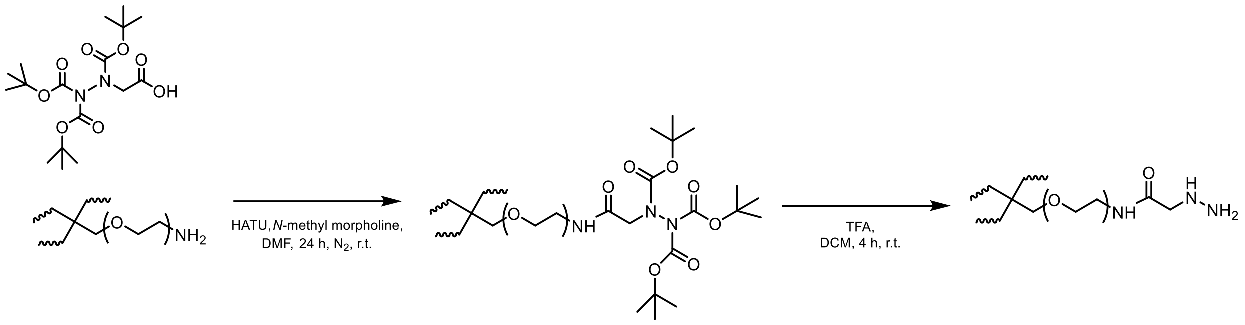

5 kDa 4PEG hydrazine was synthesized following the reported procedure.[17] Tri-boc-hydrazinoacetic acid (1.37 g, 3.52 mmol, 2.20 eq. to NH_2 groups) was activated with 4-methylmorpholine (0.880 mL, 8.00 mmol, 5.00 eq. to NH_2 groups) and HATU (1.22 g, 3.20 mmol, 2.00 eq. to NH_2 groups) in DMF 10.0 mL. In a separate vessel, 4-methylmorpholine (0.88 mL, 8.00 mmol, 5.00 eq. to NH_2 groups was added to 4-arm PEG-NH_2, HCl salt (M_n 5000 g/mol) (2.00 g, 0.400 mmol, 1.00 eq.) in DMF 5.00 mL. The 4-arm PEG-NH_2, HCl salt containing 4-methylmorpholine was slowly added to the first solution under N_2. The reaction was allowed to proceed at room temperature (25 °C) for 24 h. The reaction mixture was concentrated and precipitated in cold diethyl ether (-25 °C), filtered and dried to obtain 5k4APEG-NBoc-NBoc2 (Figure S8). ^{1}H NMR (400 MHz, $CDCl_3$): δ = 3.83-3.41 (m, 113.5H, -O-CH_2-CH_2-O-), 1.55-1.50 (d, 18H, -OC(CH_3)$_3$), δ = 1.48-1.42 (d, 9H, -OC(CH_3)$_3$).

After that, the 5k4PEG-NBoc-NBoc2 was dissolved in a 50:50 mixture of trifluoroacetic acid (TFA) and DCM. The reaction proceeded for 4 h and then precipitated into cold diethyl ether. The crude product was filtered and concentrated under reduced pressure, dissolved in DI water, transferred to regenerated cellulose membranes (Spectra/Por) with a molecular weight cut-off of 2,000 g/mol, and dialyzed against DI water for 1 day. The product was lyophilized to give a yellow powder (42 % yield, Figure S9). ^{1}H NMR (400 MHz, $CDCl_3$): δ= 3.83-3.41 (m, 113.5H, -O-CH_2-CH_2-O-).

Synthesis of 2 kDa mPEG-hydrazine (2kDa mPEG-Hz):

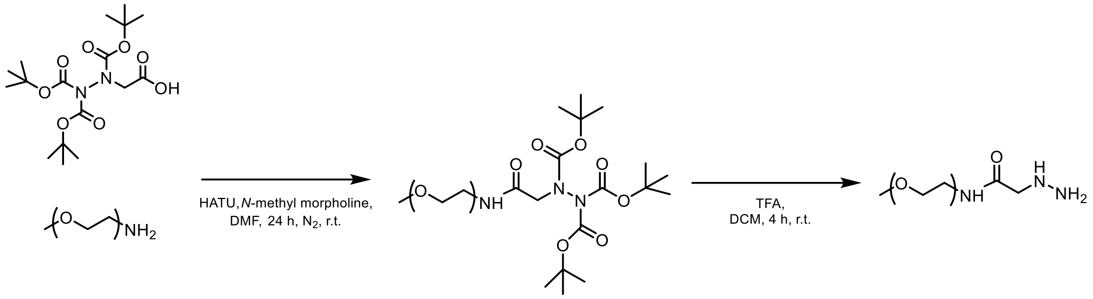

m-PEG hydrazine was synthesized following the reported procedure.[17] Tri-boc-hydrazinoacetic acid (430 mg, 1.10 mmol, 2.20 eq. to NH_2 groups) was activated with 4-methylmorpholine (0.270 mL, 2.50 mmol, 5.00 eq. to NH_2 groups) and HATU (380 mg, 1.0 mmol, 2.00 eq. to NH_2 groups) in DMF 10.0 mL. In a separate vessel, 4-methylmorpholine (0.270 mL, 2.50 mmol, 5.00 eq. to NH_2 groups was added to mPEG-NH_2 (M_n 2000 g/mol) (1.00 g, 0.500 mmol, 1.00 eq.) in DMF 5.00 mL. The mPEG-NH_2 containing 4-methylmorpholine was slowly added to the first solution under N2. The reaction was allowed to proceed at room temperature (25 °C) for 24 h. The reaction mixture was concentrated and precipitated in cold diethyl ether (-25 °C), filtered and dried to obtain 5k4PEG-NBoc-NBoc2 (Figure S10). ^{1}H NMR (400 MHz, $CDCl_3$): δ = 3.84-3.37 (m, 181H, -O-CH_2-CH_2-O-), 1.57-1.48 (d, 18H, -OC(CH_3)$_3$), δ = 1.48-1.41 (d, 9H, -OC(CH_3)$_3$).

After that, the 2 kDa mPEG-NBoc-NBoc2 was then dissolved in a 50:50 mixture of trifluoroacetic acid (TFA) and DCM. The reaction was allowed to proceed for 4 h and then precipitated into cold diethyl ether again. The crude product was filtered and concentrated under reduced pressure, dissolved in DI water, transferred to regenerated cellulose membranes (Spectra/Por) with a molecular weight cut-off of 2,000 g/mol, and dialyzed against DI water for 1 day. The product was lyophilized to give a yellow powder (22 % yield, Figure S11). ^{1}H NMR (400 MHz, $CDCl_3$): δ= 3.84-3.37 (m, 181H, -O-CH_2-CH_2-O-).

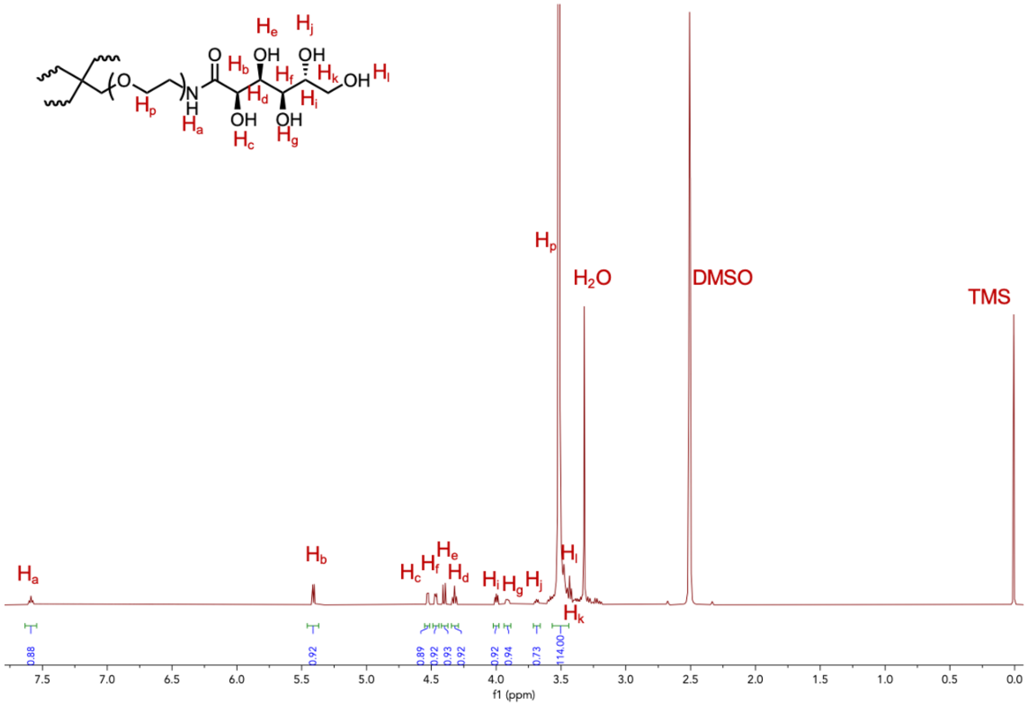

Figure S3: 1D ^{1}H NMR of 5 kDa 4PEG-GA (400 MHz, DMSO-d6, 25 °C).

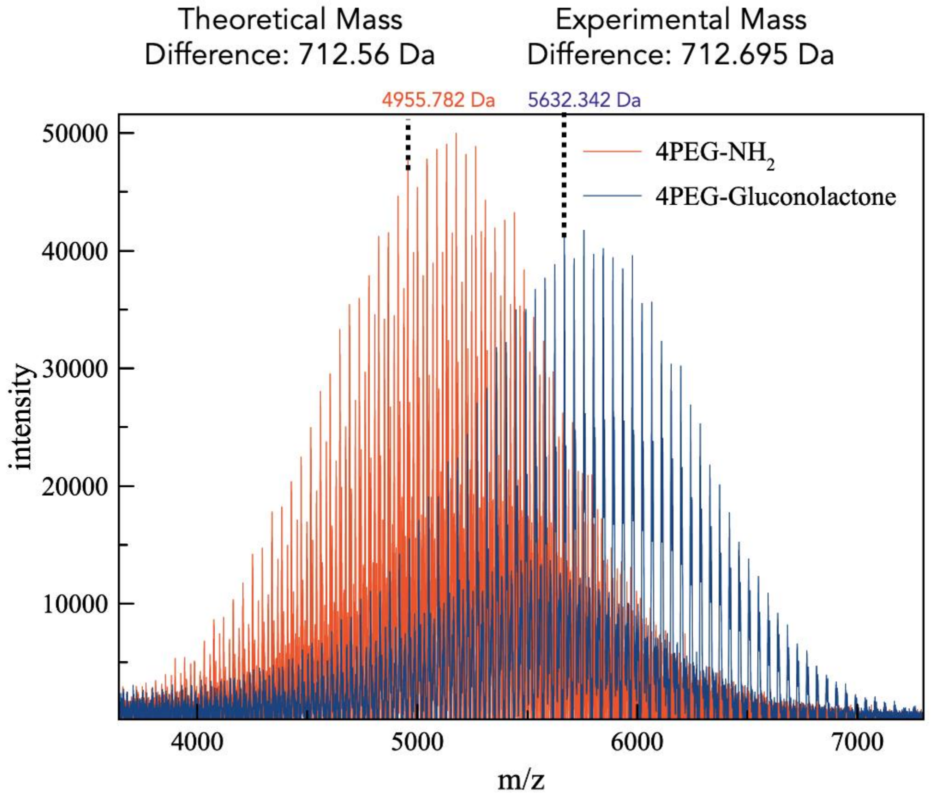

Figure S4: MALDI-TOF Analysis of 5 kDa 4PEG-GA.

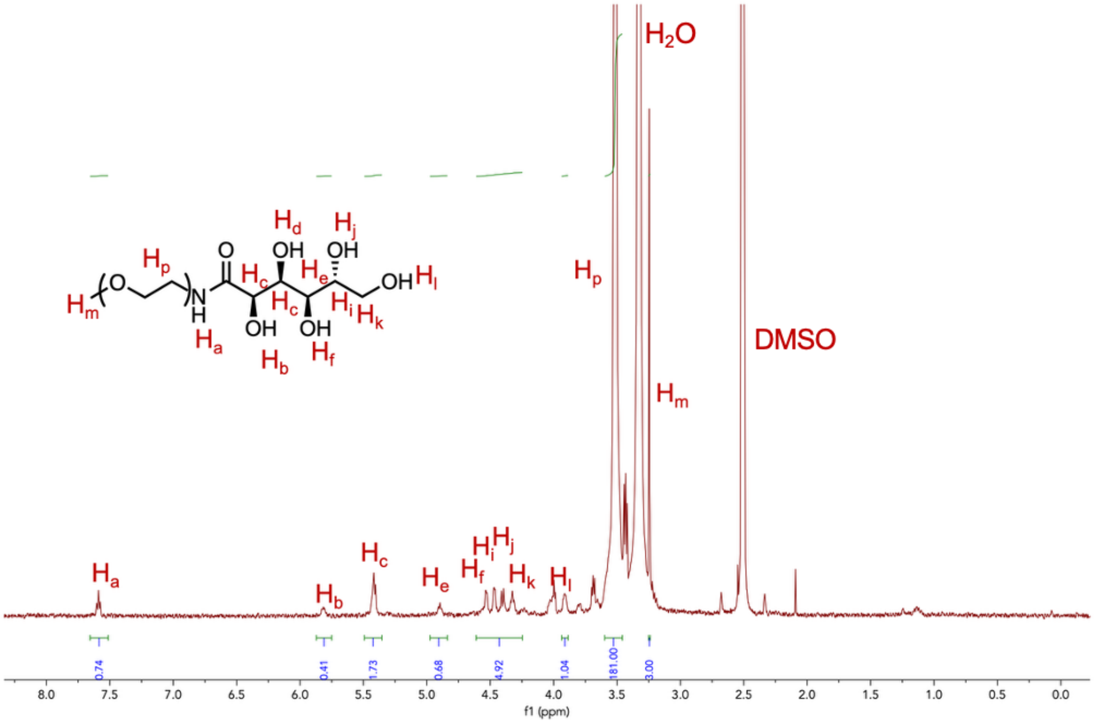

Figure S5: 1D ^{1}H NMR of 2 kDa mPEG-GA (400 MHz, DMSO-d6, 25 °C).

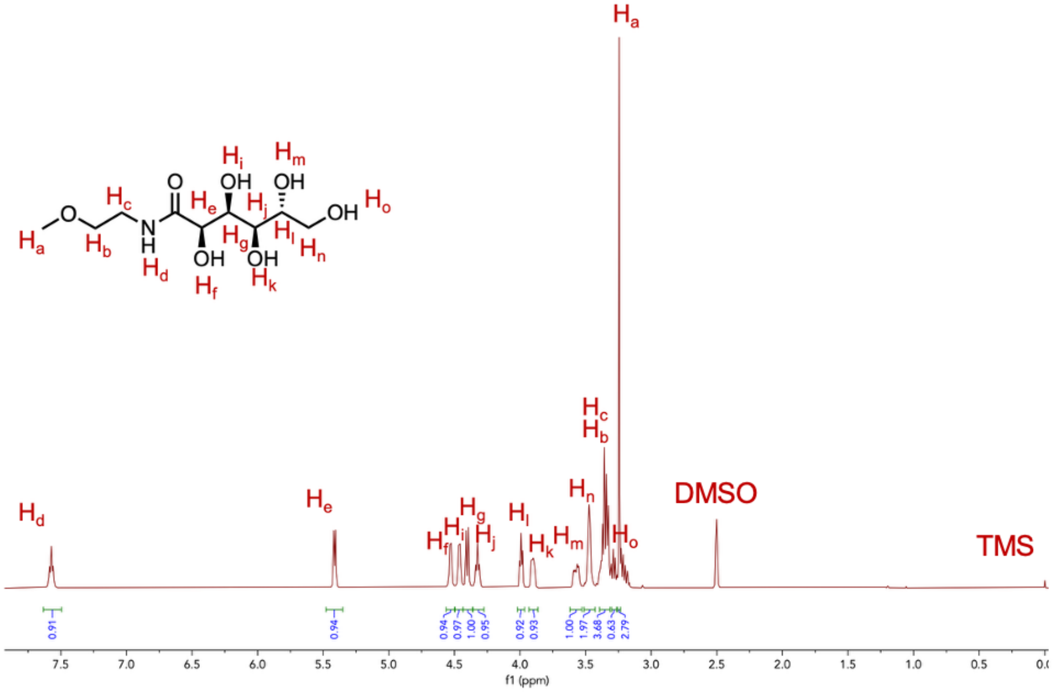

Figure S6: 1D ^{1}H NMR of (2R,3S,4R,5R)-2,3,4,5,6-pentahydroxy-N-(2-methoxyethyl)hexanamide (Cl-ana) (400 MHz, DMSO-d6, 25 °C).

.

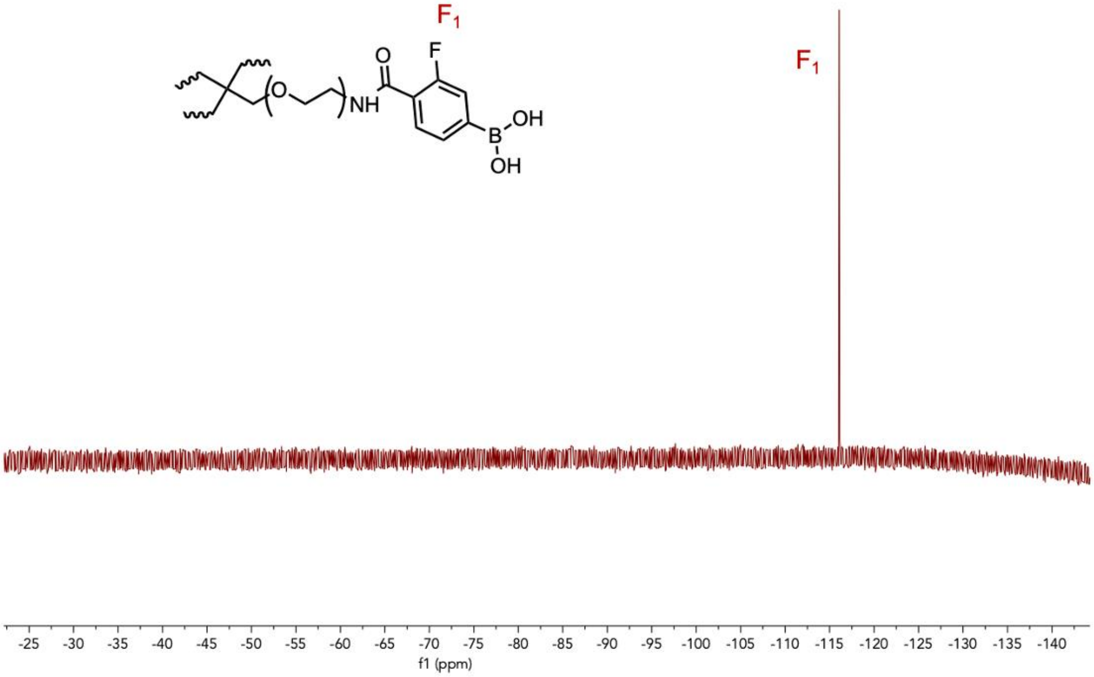

Figure S7: 1D ^{19}F NMR of 5 kDa 4PEG-FPBA (400 MHz, DMSO-d6, 25 °C).

.

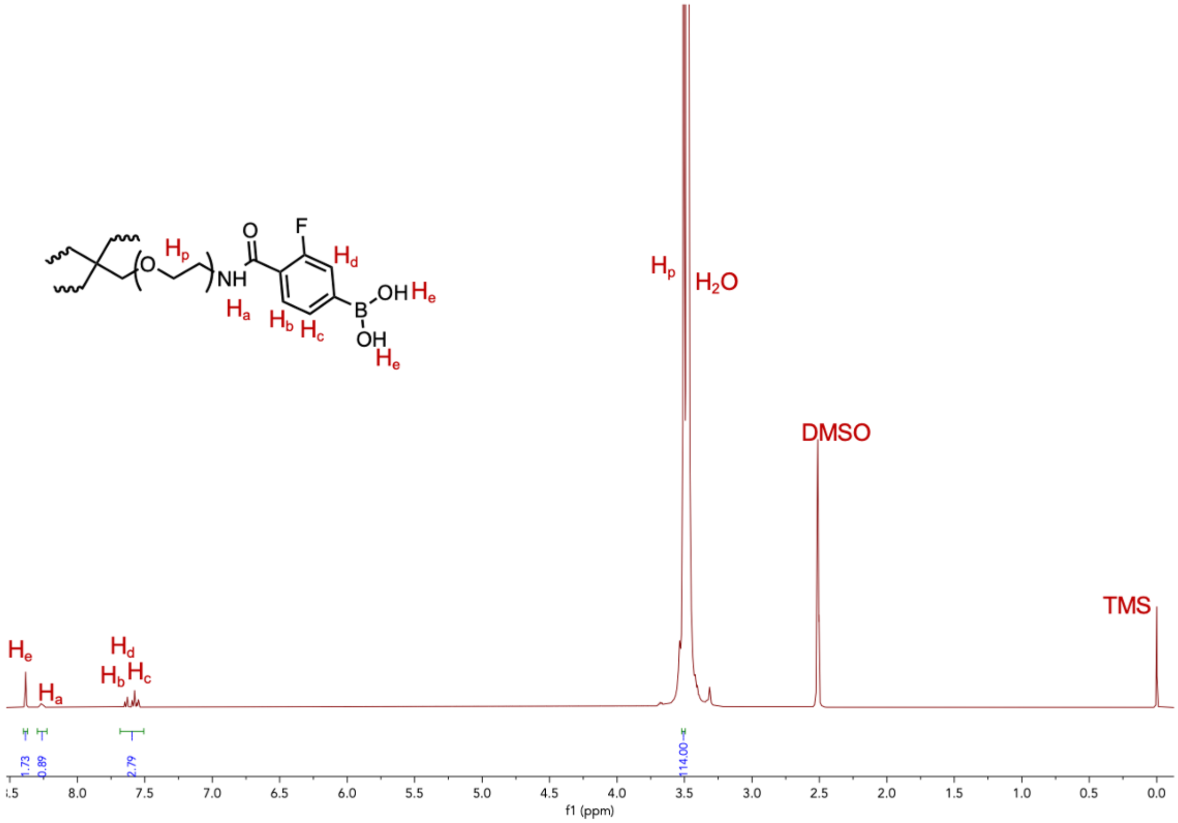

Figure S8: 1D ^{1}H NMR of 5 kDa 4PEG-FPBA (400 MHz, DMSO-d6, 25 °C).

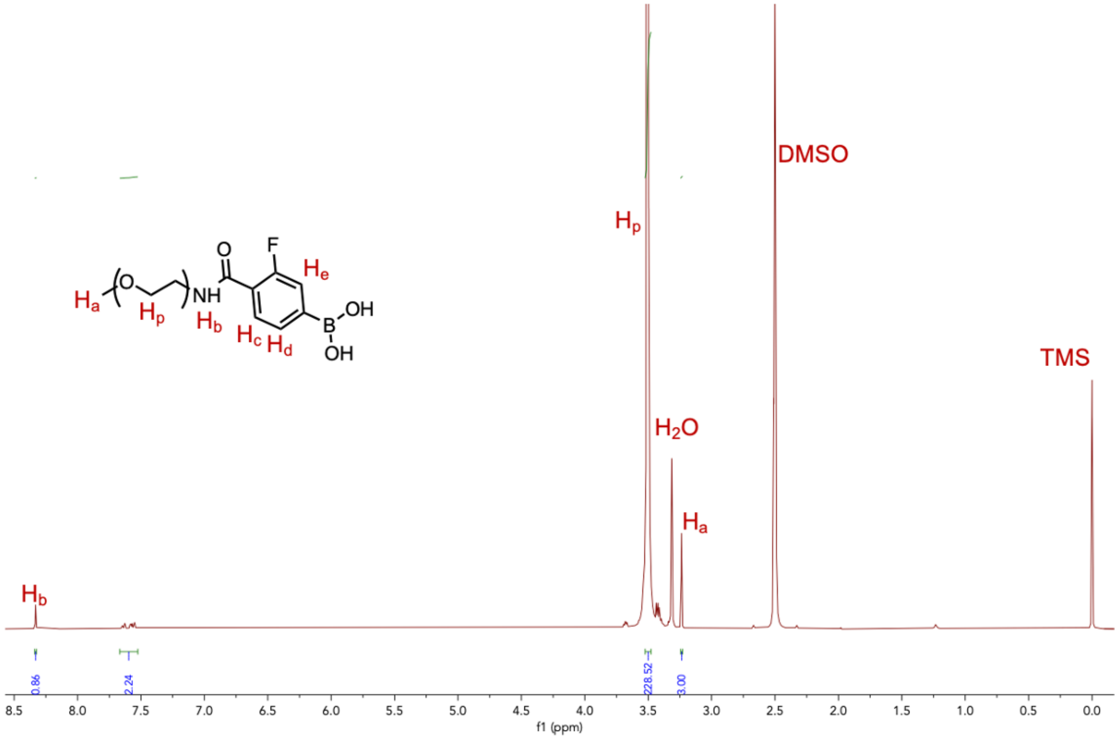

Figure S9: 1D ^{1}H NMR of 2 kDa mPEG-FPBA (400 MHz, DMSO-d6, 25 °C).

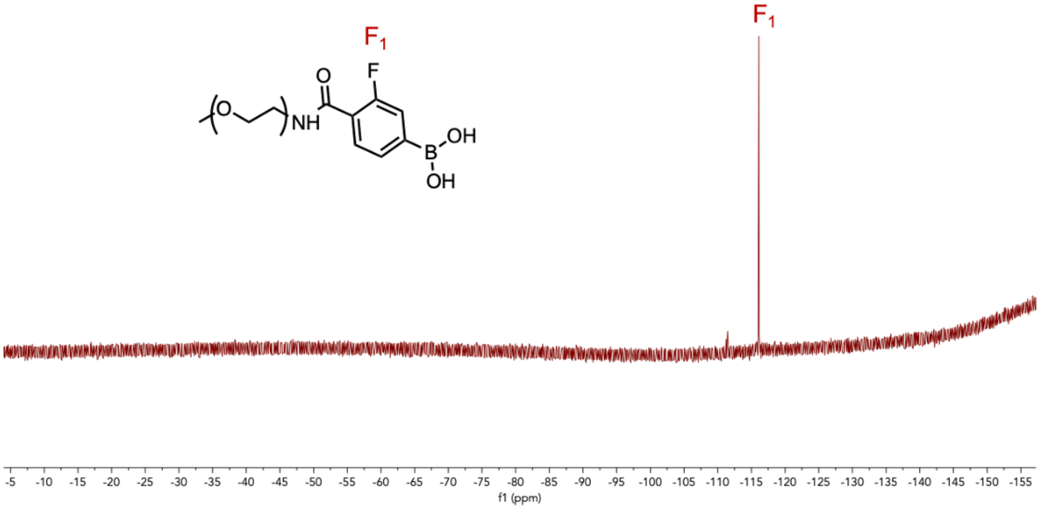

Figure S10: 1D ^{1}H NMR of 2 kDa mPEG-FPBA (400 MHz, DMSO-d6, 25 °C).

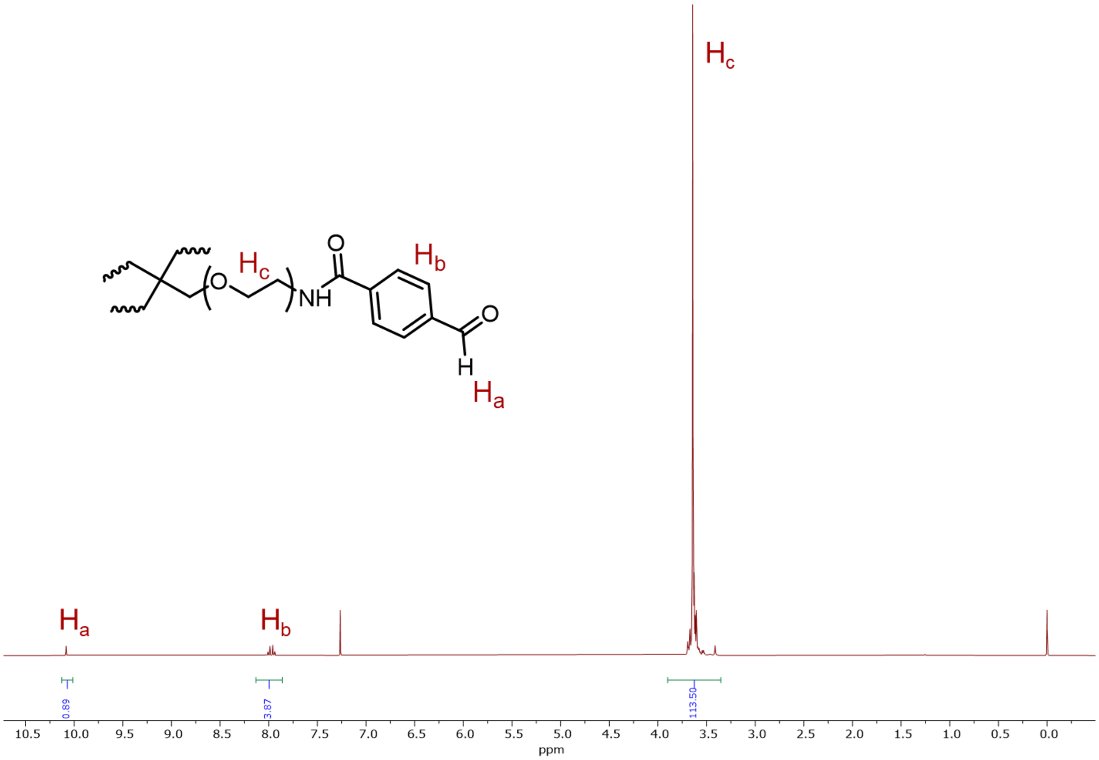

Figure S11. ^{1}H NMR spectrum of 5kDa 4PEG-Ar-CHO (400 MHz, $CDCl_3$, 25 °*C*).

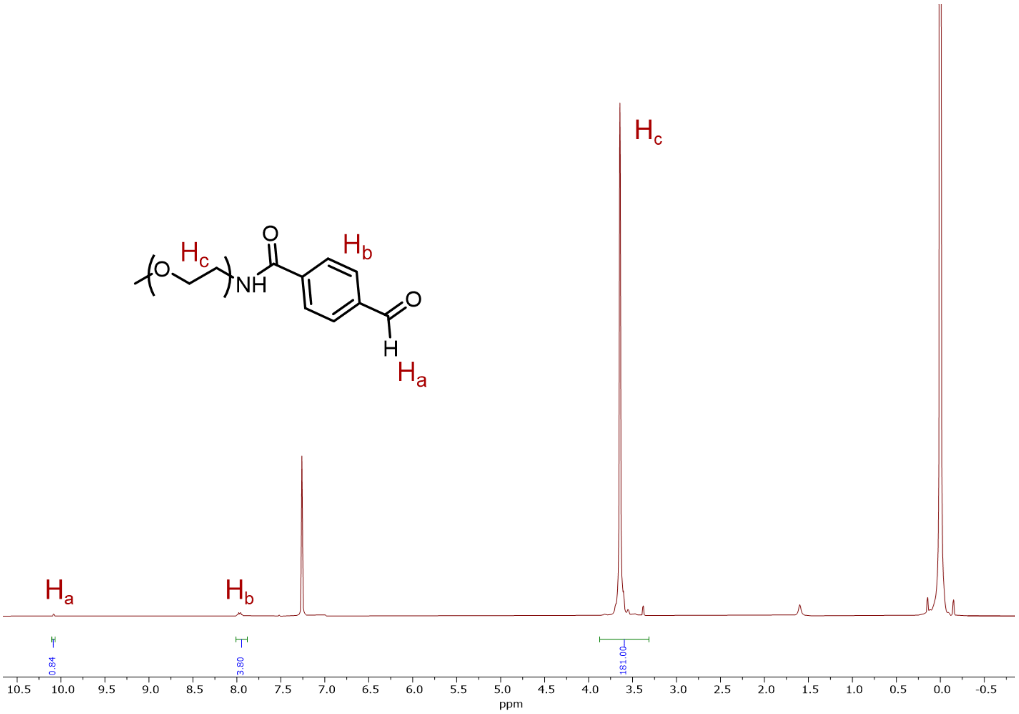

Figure S12. ^{1}H NMR spectrum of 2kDa mPEG-Ar-CHO (400 MHz, $CDCl_3$, 25 °C).

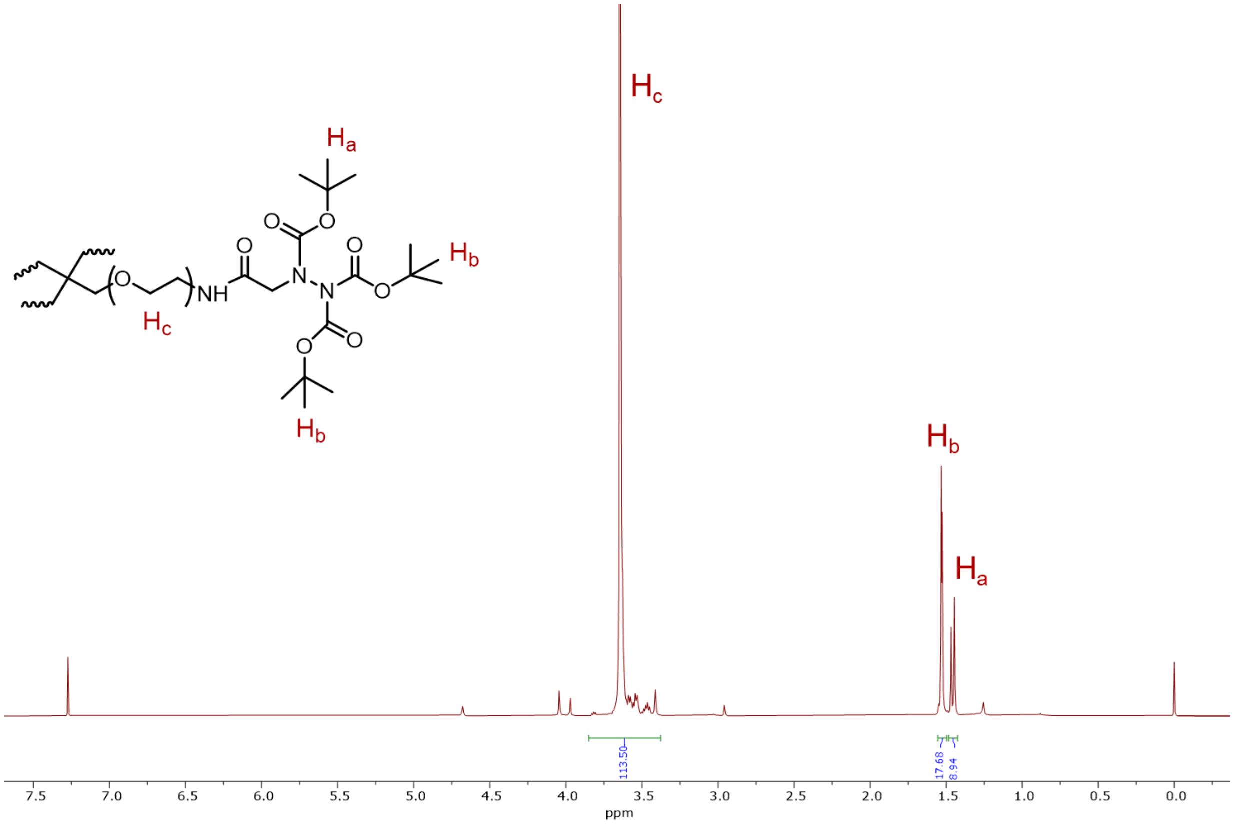

Figure S13. ^{1}H NMR spectrum of 5kDa 4PEG-NBoc-NBoc$_2$ (400 MHz, $CDCl_3$, 25 °C).

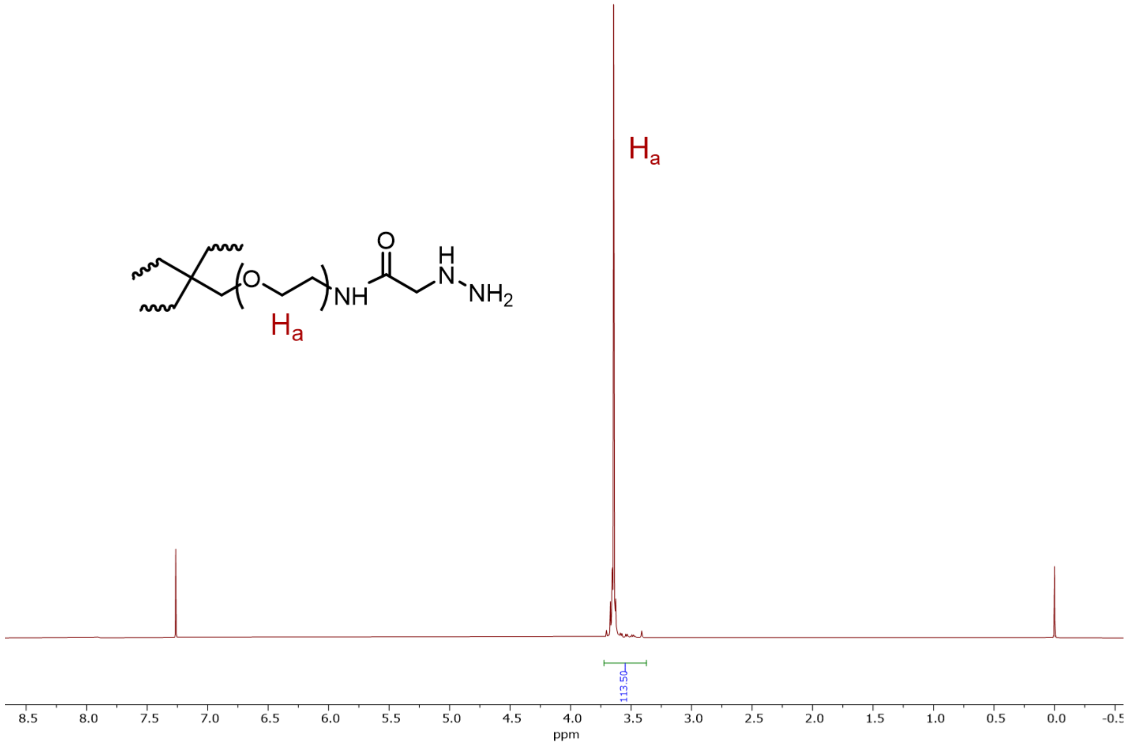

Figure S14. ^{1}H NMR spectrum of 5kDa 4PEG-Hz (400 MHz, $CDCl_3$, 25 °C).

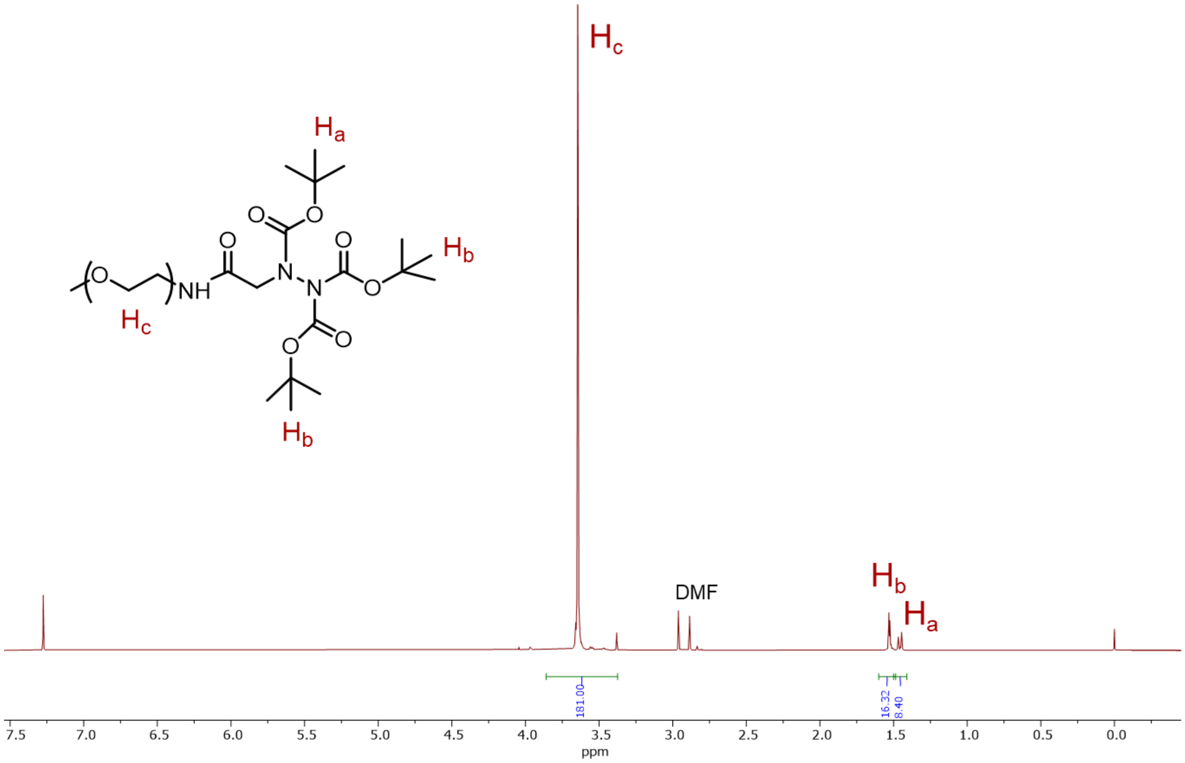

Figure S15. ^{1}H NMR spectrum of 2kDa mPEG-NBoc-NBoc$_2$ (400 MHz, $CDCl_3$, 25 °C).

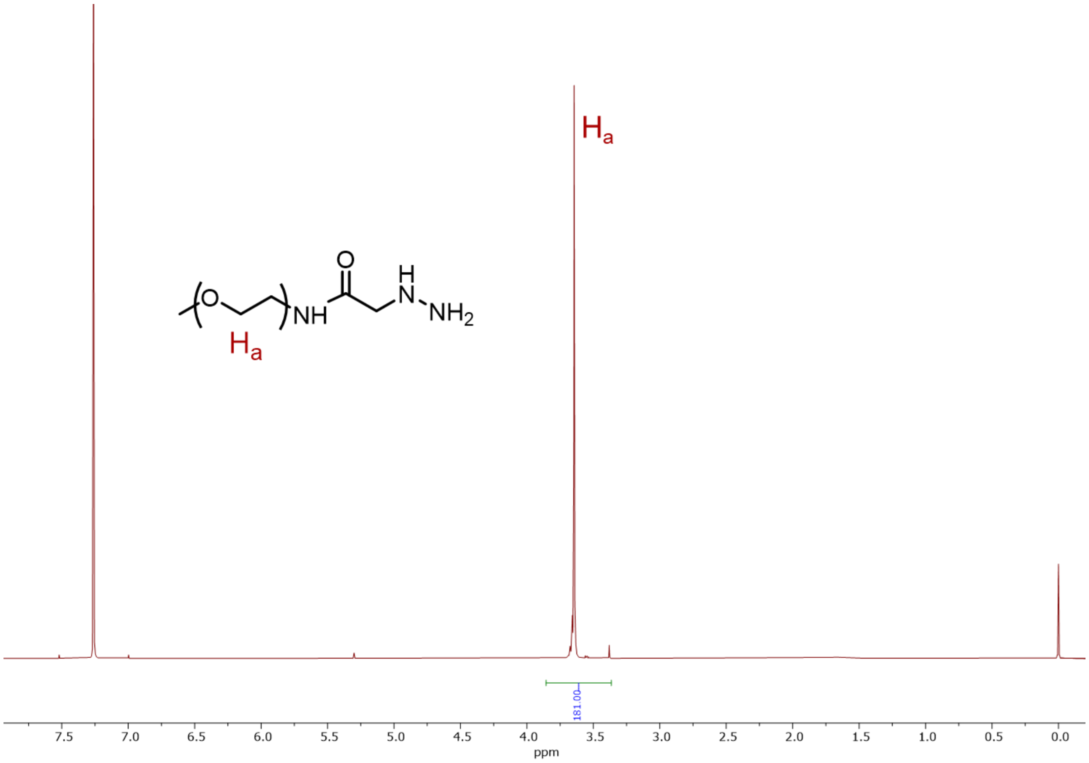

Figure S16. ^{1}H NMR spectrum of 2kDa mPEG-Hz (400 MHz, $CDCl_3$, 25 °C).

ITC

ITC Procedures**:**

For the boronate ester system, ITC experiments were conducted at 25 °C with 30 injections. The syringe contained 27.5 mM mPEG–phenylboronic acid, while the cell was filled with 4 mM of a diol species (either a competitor or the mPEG-based crosslinker). All association constants were determined through the kinITC software.[18]

The heats of injection were plotted as a binding isotherm to determine the Ka values. The koff values were determined by extracting the equilibration time between each injection and fitting these values as a function of molar ratio. When determining the koff values the key assumptions that the software makes are:

(1) The instrument gives a single response time
(2) The time between each injection starting, and returning to the baseline can be extracted, plotted against the molar ratio of the interaction and fit in a parabolic curve to return the rate constants of the binding interaction.

For more details regarding the kinITC process for determining the rate constants of the interactions please refer to the corresponding publications.[18,19]

Triplicate measurements were performed for each competitor, and fit-derived residuals and are reported (Ka std, koff std).

Glucose (1)

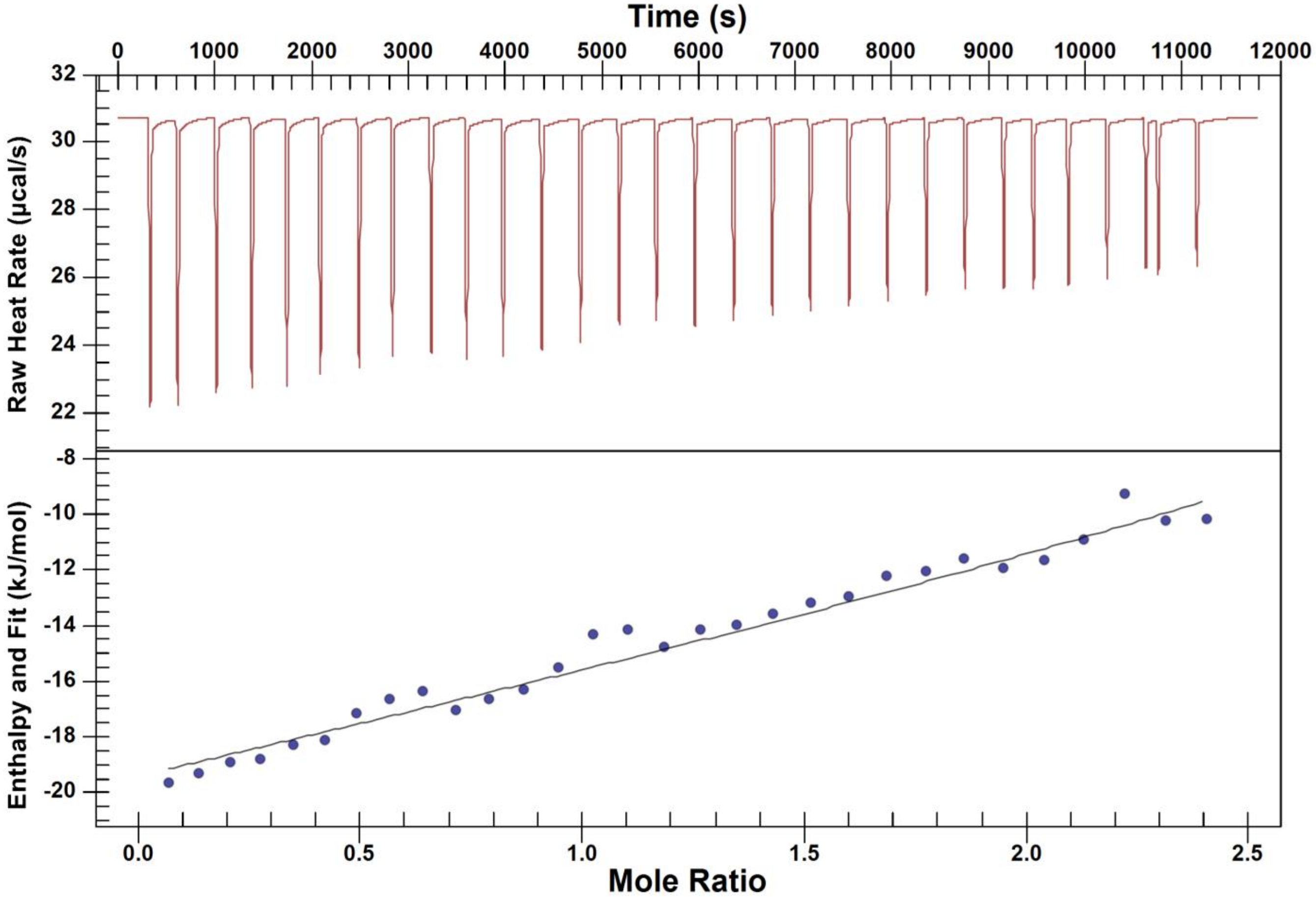

Figure S17: ITC heats of injection and binding isotherm of Glucose titrated with mPEG-FPBA Trial 1 conducted at 25 °C.

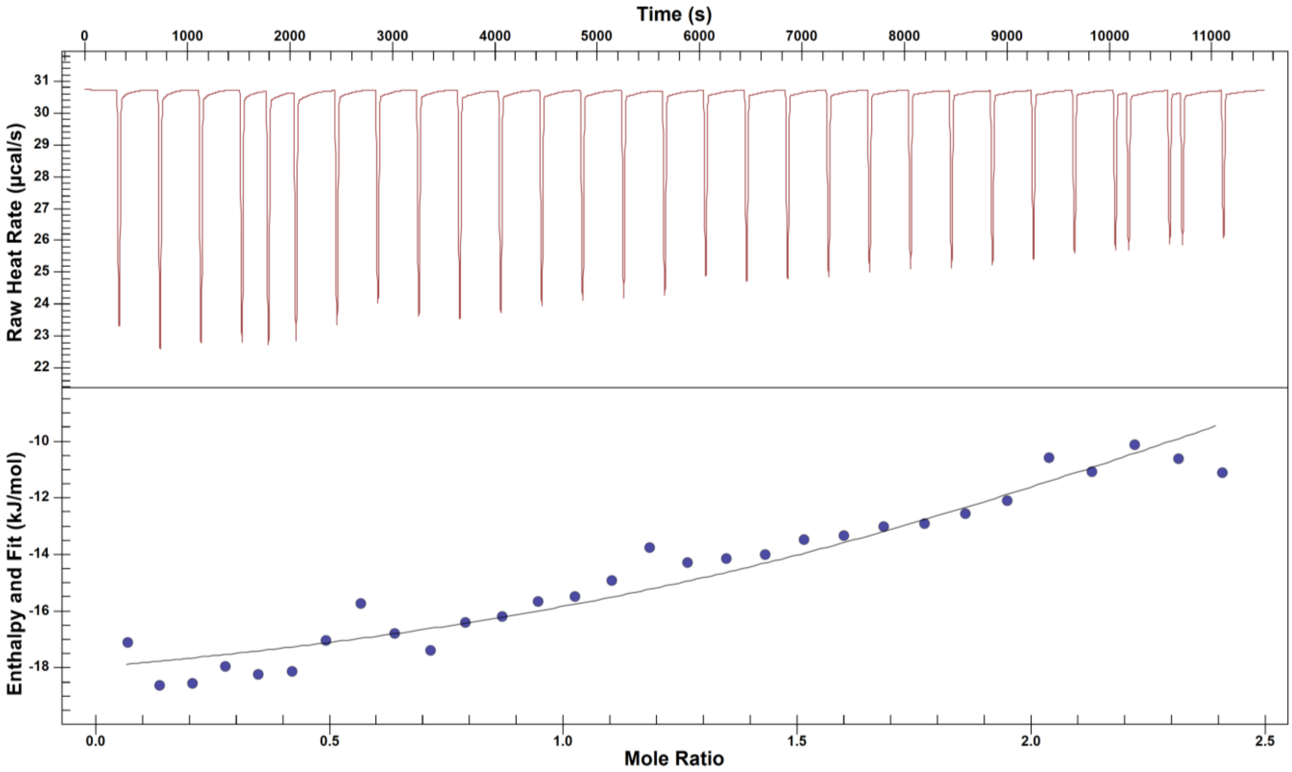

Figure S18: ITC heats of injection and binding isotherm of Glucose titrated with mPEG-FPBA Trial 2 conducted at 25 ^{o}C.

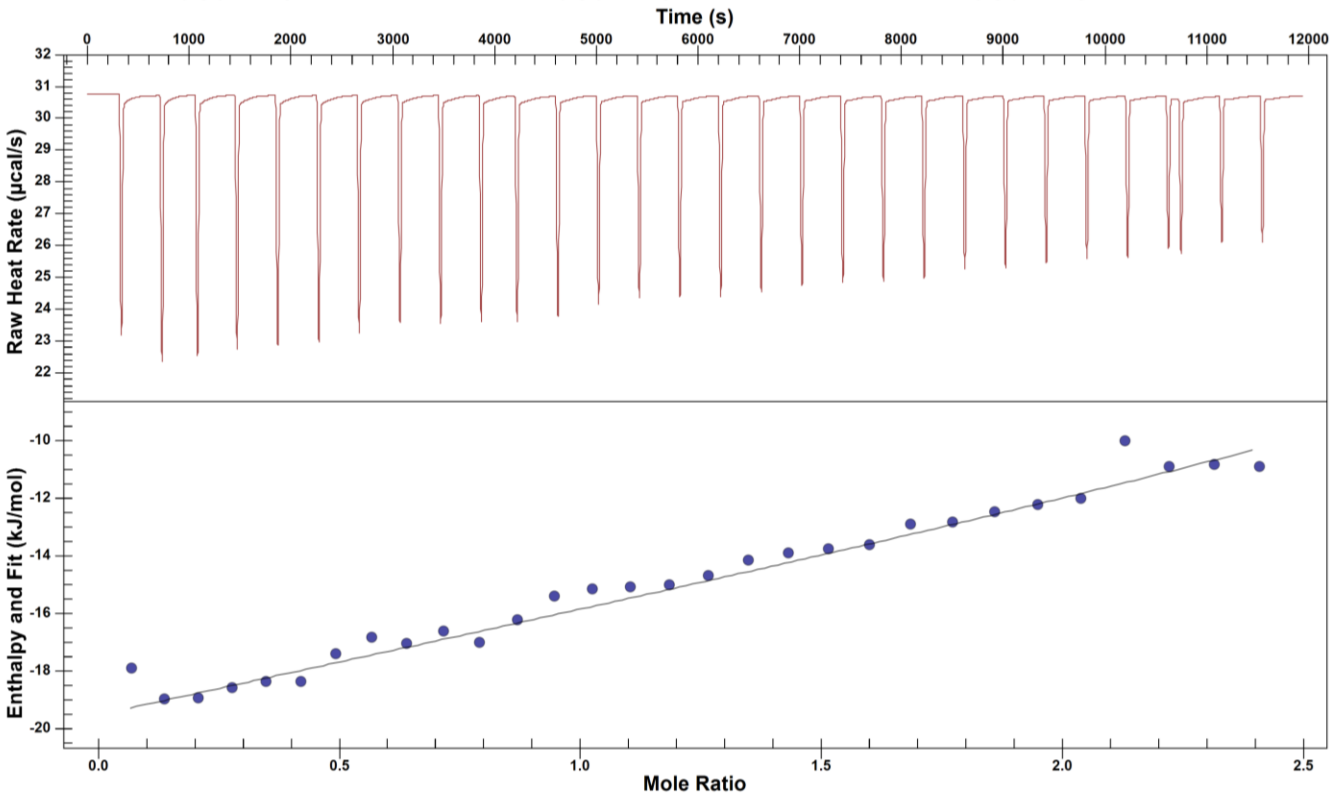

Figure S19: ITC heats of injection and binding isotherm of Glucose titrated with mPEG-FPBA Trial 3 conducted at 25 ^{o}C.

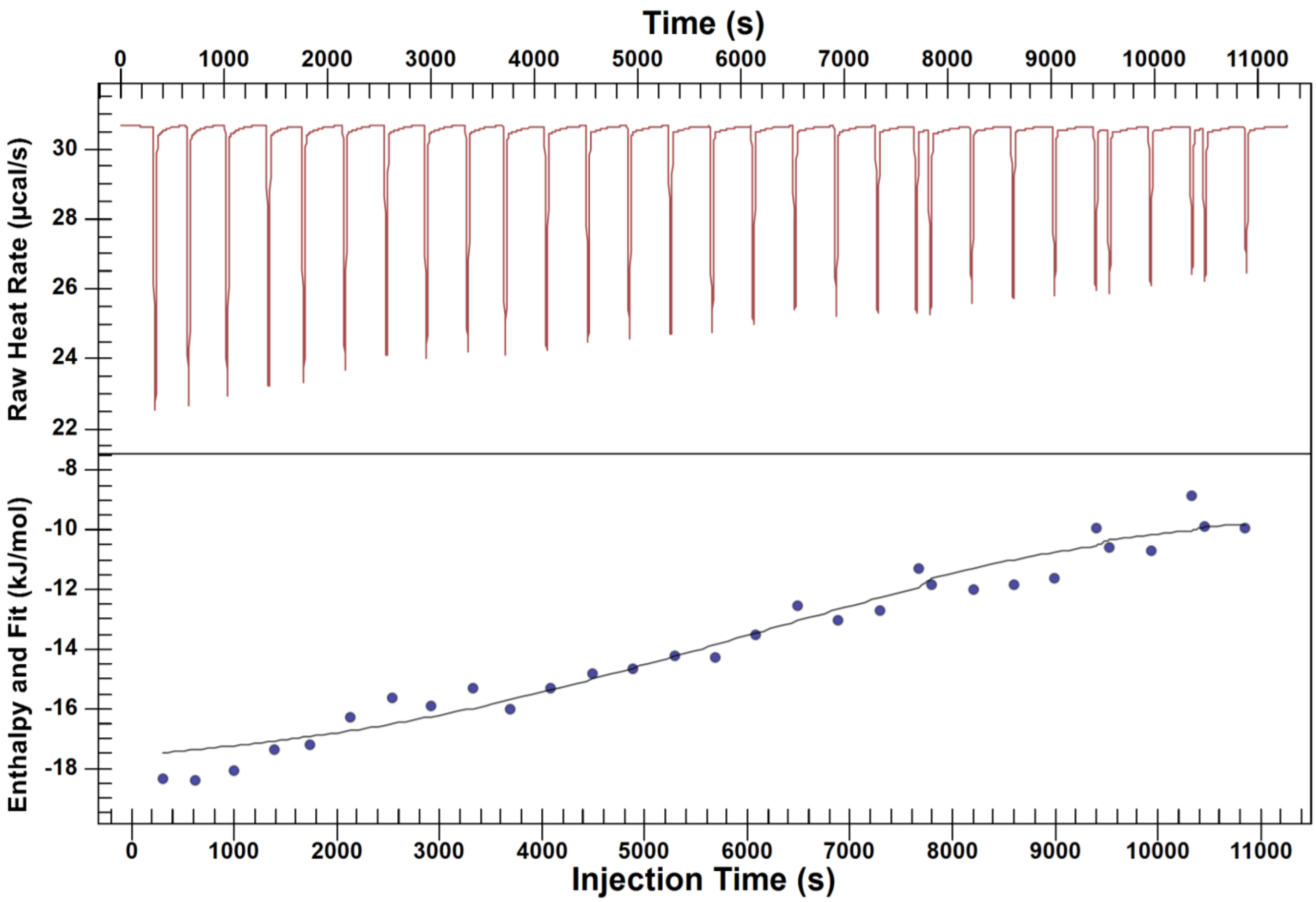

Figure S20: ITC heats of injection and binding isotherm of Dyphylline titrated with mPEG-FPBA Trial 4 conducted at 25 ^{o}C.

Table S1: Summary of ITC fit from glucose runs determined by kinITC[3].

KinITC	**Glucose**						
Trial	n	Kd (M)	Ka (M^{-1})	Ka std	koff ($M^{-1}s^{-1}$)	koff std	kon
1	1.01E+0	1.06E-1	9.40E+0	6.57E+0	9.83E-1	1.40 E+0	9.24E+0
2	7.85E-1	1.35E-1	7.40E+0	2.07E+0	1.36E+2	2.49 E+2	1.01E+3
3	1.93E+0	3.34E-1	3.00E+0	9.59E+0	9.54E-1	1.98 E+0	2.86E+0
4	1.05E+0	1.65E-1	6.06E+0	2.35E+0	2.49E+0	2.29 E+0	1.51E+1
avg	1.19E+0	1.85E-1	6.46E+0		3.51E+1		2.59E+2
std	5.03E-1	1.02E-1	2.69E+0		6.73E+1		4.99E+2

Dyphylline (2)

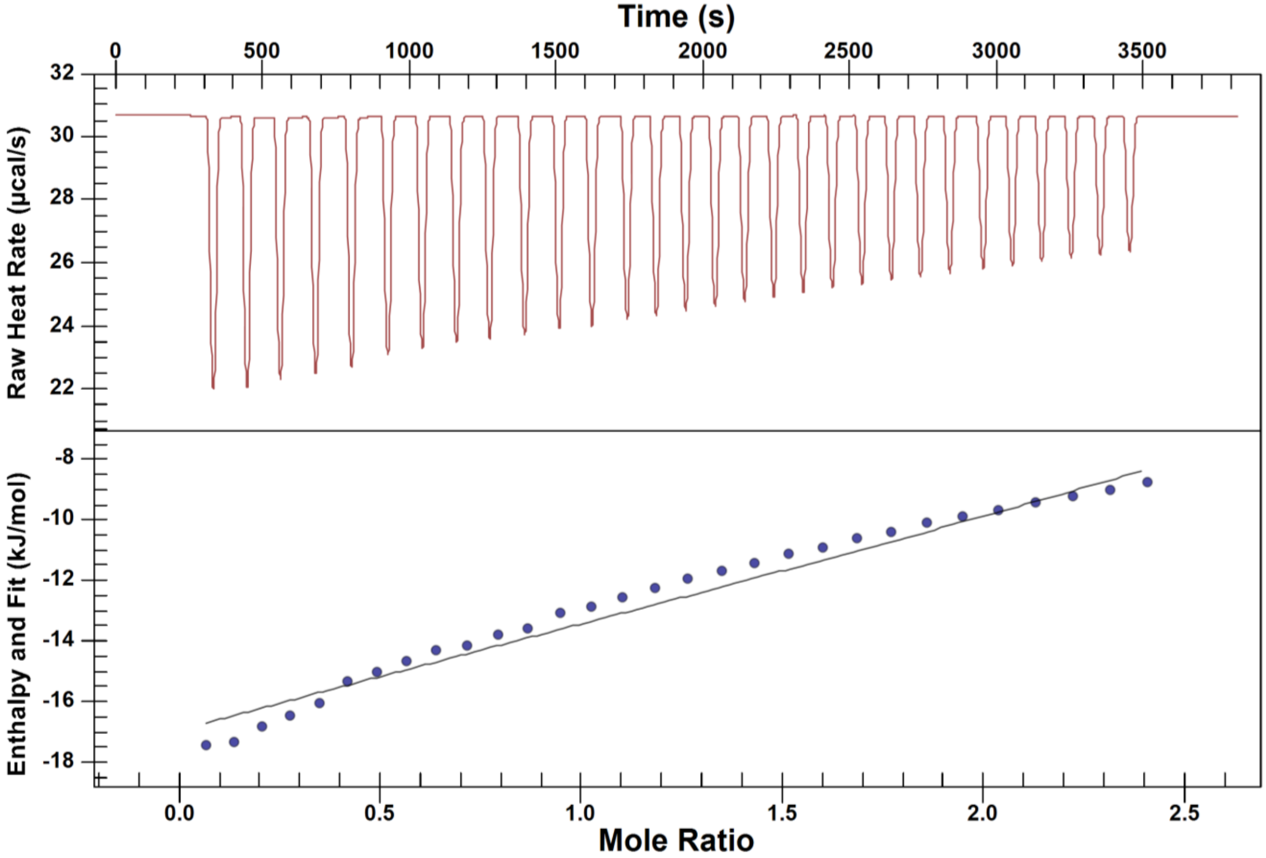

Figure S21: ITC heats of injection and binding isotherm of Dyphylline titrated with mPEG-FPBA Trial 1 conducted at 25 °C.

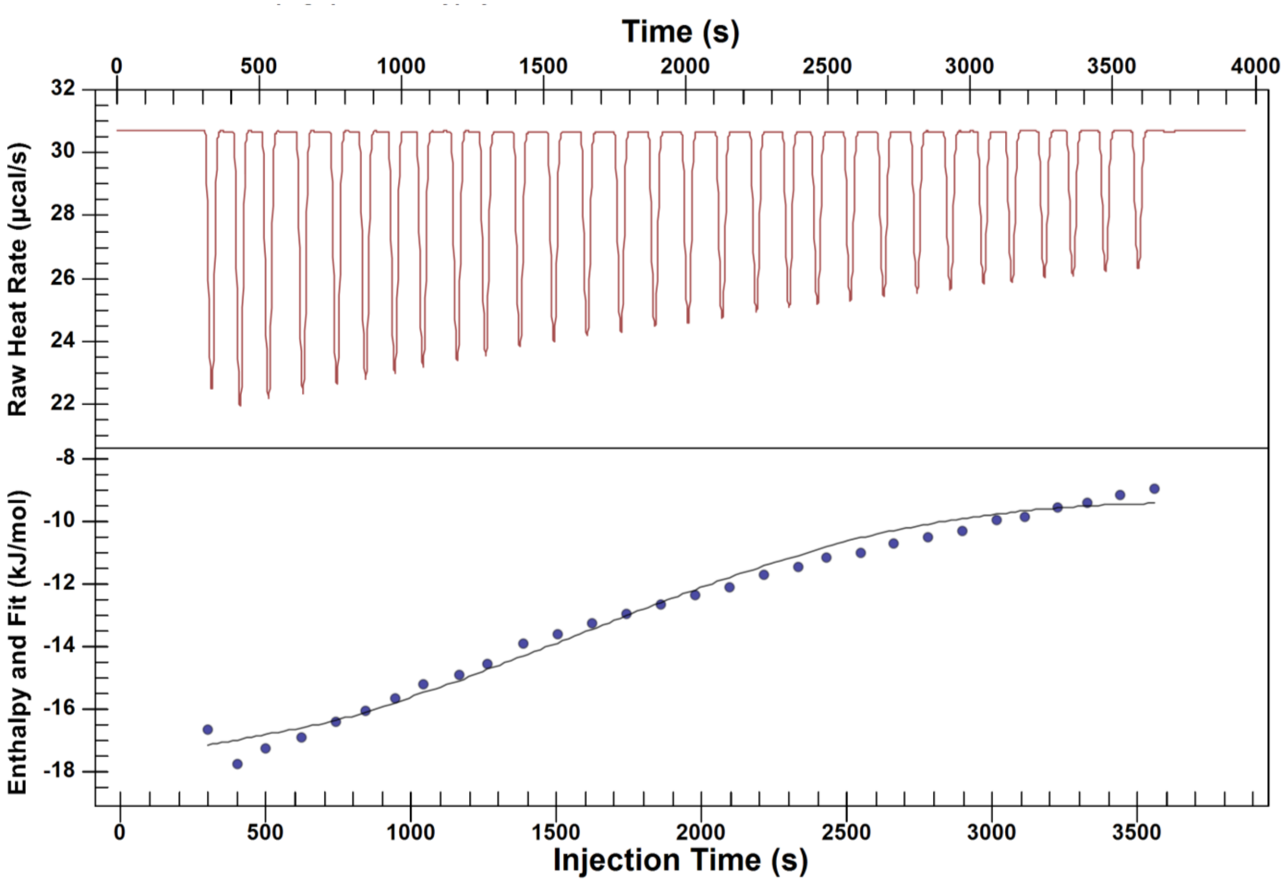

Figure S22: ITC heats of injection and binding isotherm of Dyphylline titrated with mPEG-FPBA Trial 2 conducted at 25 ^{o}C.

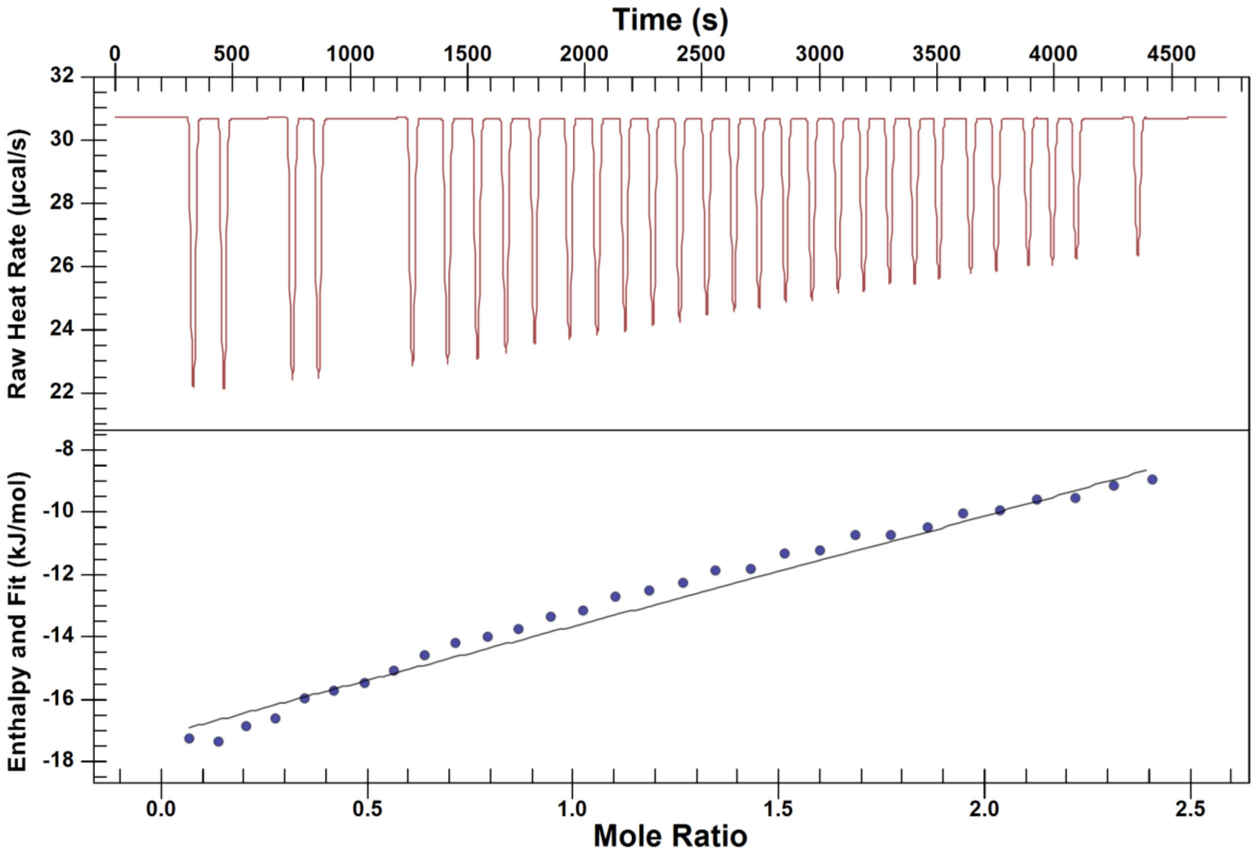

Figure S23: ITC heats of injection and binding isotherm of Dyphylline titrated with mPEG-FPBA Trial 3 conducted at 25 °C.

Table S2: Summary of ITC fits from dyphylline runs determined by kinITC[3].

KinITC	Dyphylline						
Trial	n	Kd (M)	Ka (M^{-1})	Ka std	koff ($M^{-1}s^{-1}$)	koff std	kon
1	9.87E-1	3.53E-2	2.83E+1	3.77E+0	2.69E+2	1.74E+1	7.62E+3
2	7.90E-1	3.55E-2	2.82E+1	3.38E+0	2.94E+2	1.21E+1	8.29E+3
3	1.00E+0	4.62E-2	2.16E+1	3.91E+0	2.33E+2	2.35E+1	5.03E+3
avg	9.26E-1	3.90E-2	2.60E+1		2.65E+2		6.98E+3
std	1.17E-1	6.25E-3	3.82E+0		3.09E+1		1.72E+3

Tris (3)

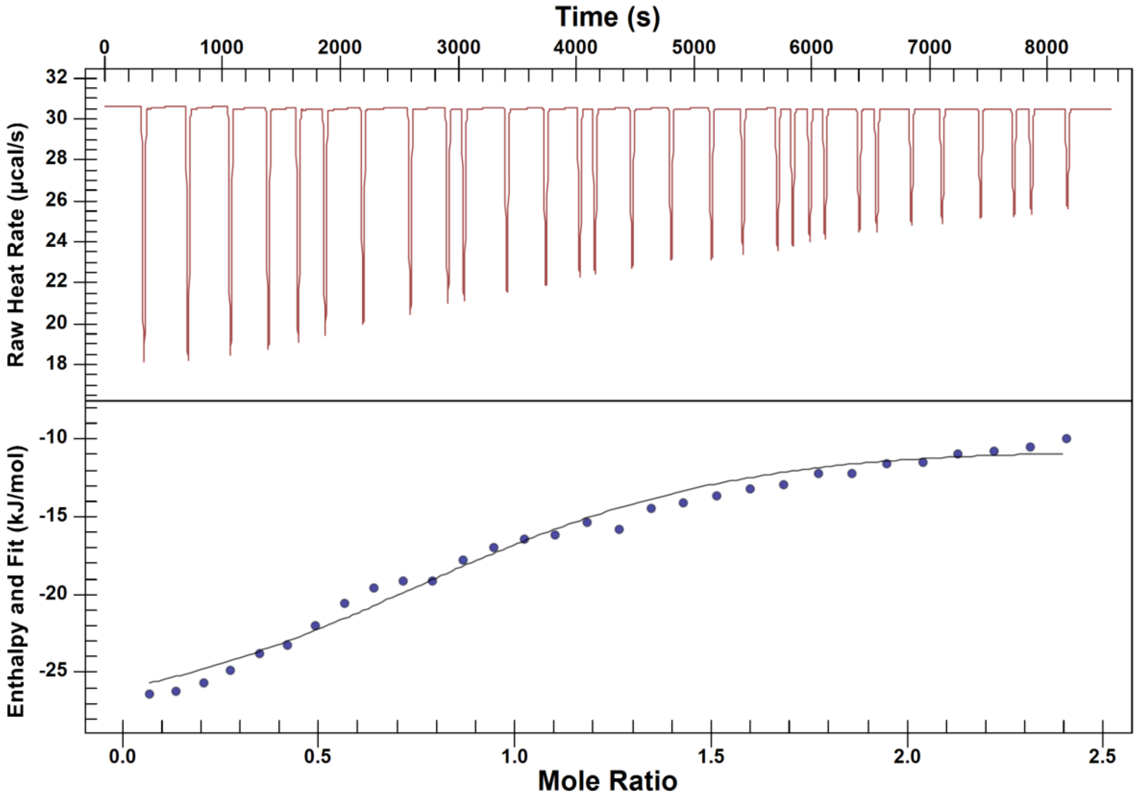

Figure S24: ITC heats of injection and binding isotherm of Tris titrated with mPEG-FPBA Trial 1 conducted at 25 ^{o}C.

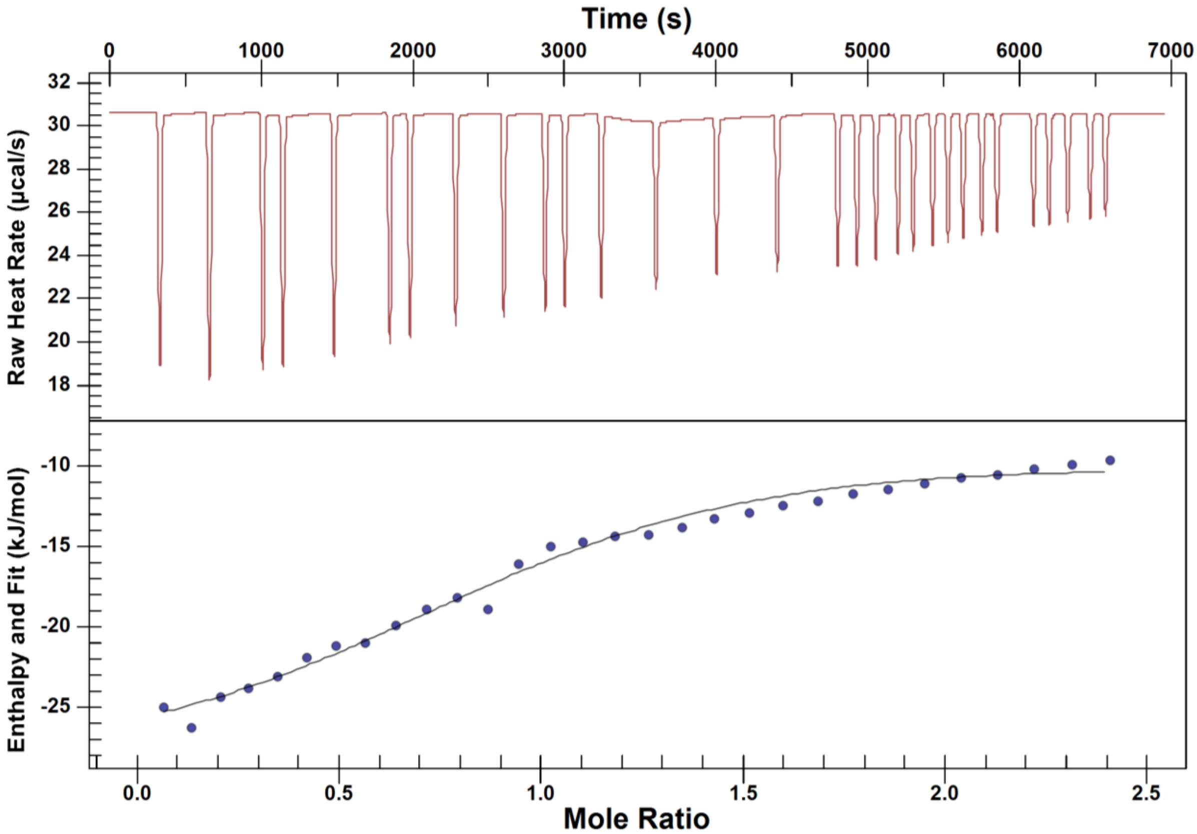

Figure S25: ITC heats of injection and binding isotherm of Tris titrated with mPEG-FPBA Trial 2 conducted at 25 °C.

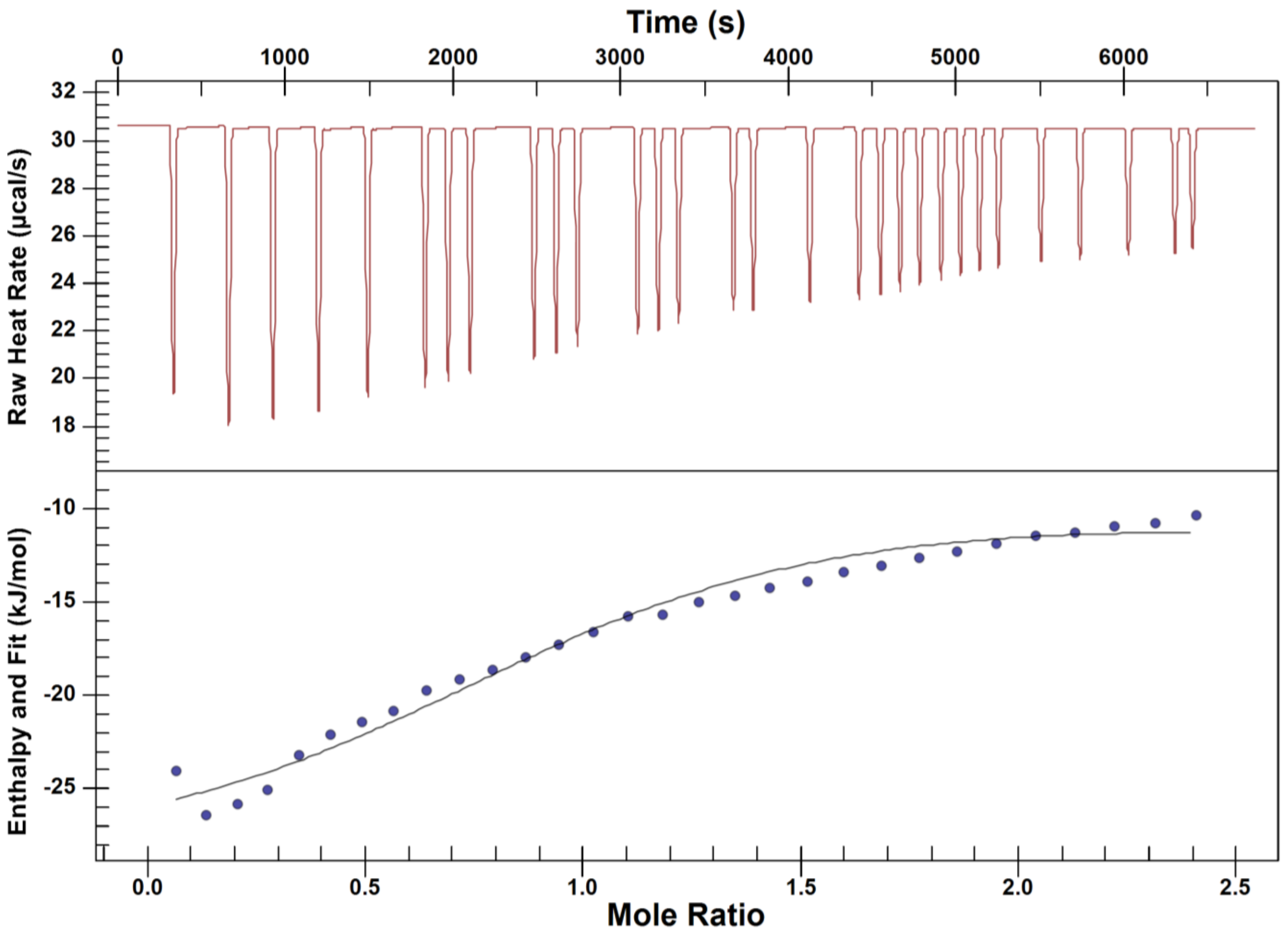

Figure S26: ITC heats of injection and binding isotherm of Tris titrated with mPEG-FPBA Trial 3 conducted at 25 °C.

Table S3: Summary of ITC fits from tris runs determined by kinITC[3].

KinITC	Tris						
Trial	n	Kd (M)	Ka (M^{-1})	Ka std	koff ($M^{-1}s^{-1}$)	koff std	kon
1	7.15E-1	2.65E-3	3.77E+2	5.04E+1	2.07E+2	2.45E+1	1.40E+5
2	9.02E-1	3.81E-3	2.63E+2	4.94E+1	1.55E+2	3.81E+1	4.07E+4
3	1.03E+0	3.18E-3	3.15E+2	3.57E+1	1.31E+2	2.77E+1	3.36E+4
avg	8.81E-1	3.21E-3	3.18E+2		1.64E+2		7.13E+4
std	1.57E-1	5.78E-4	5.72E+1		3.86E+1		5.93E+4

Capecitabine (4)

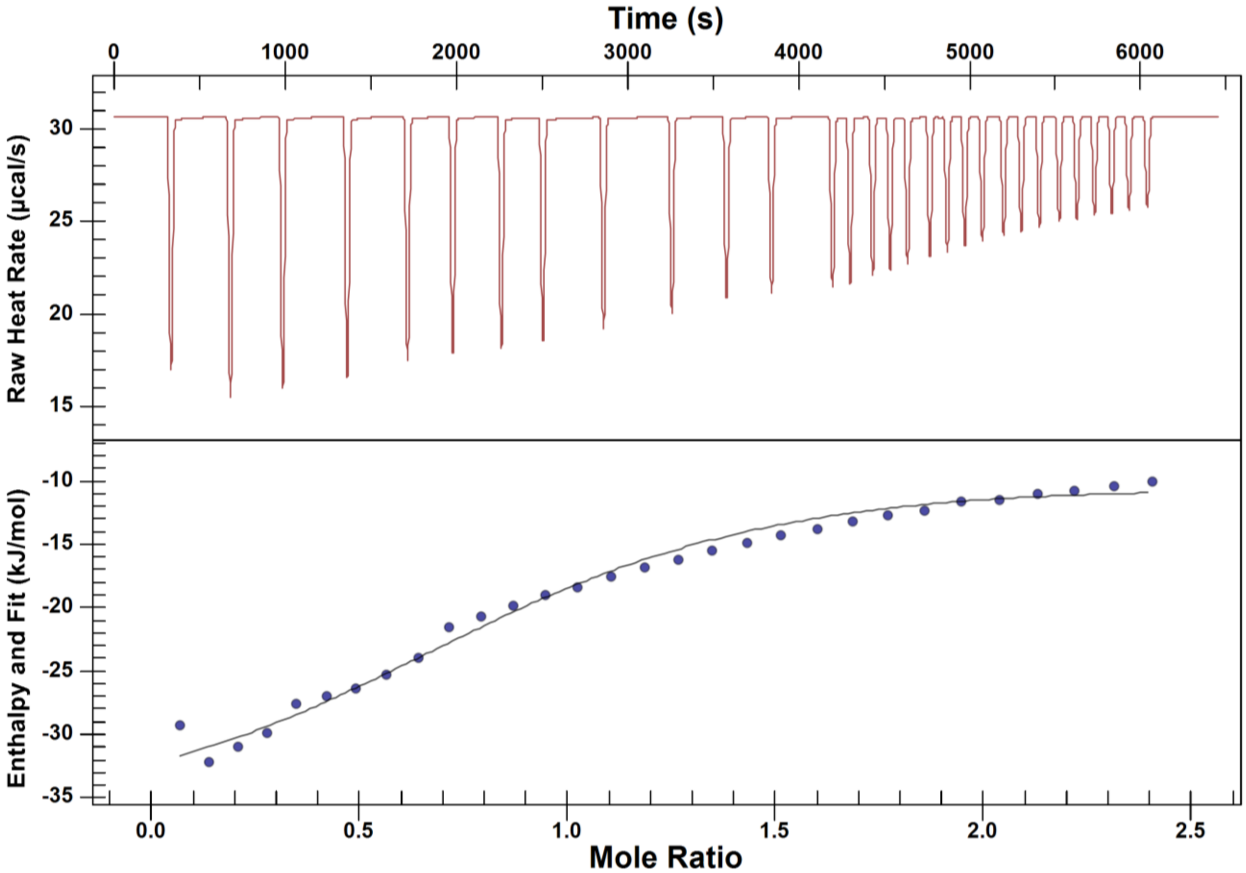

Figure S27: ITC heats of injection and binding isotherm of Capecitabine titrated with mPEG-FPBA Trial 1 conducted at 25 °C.

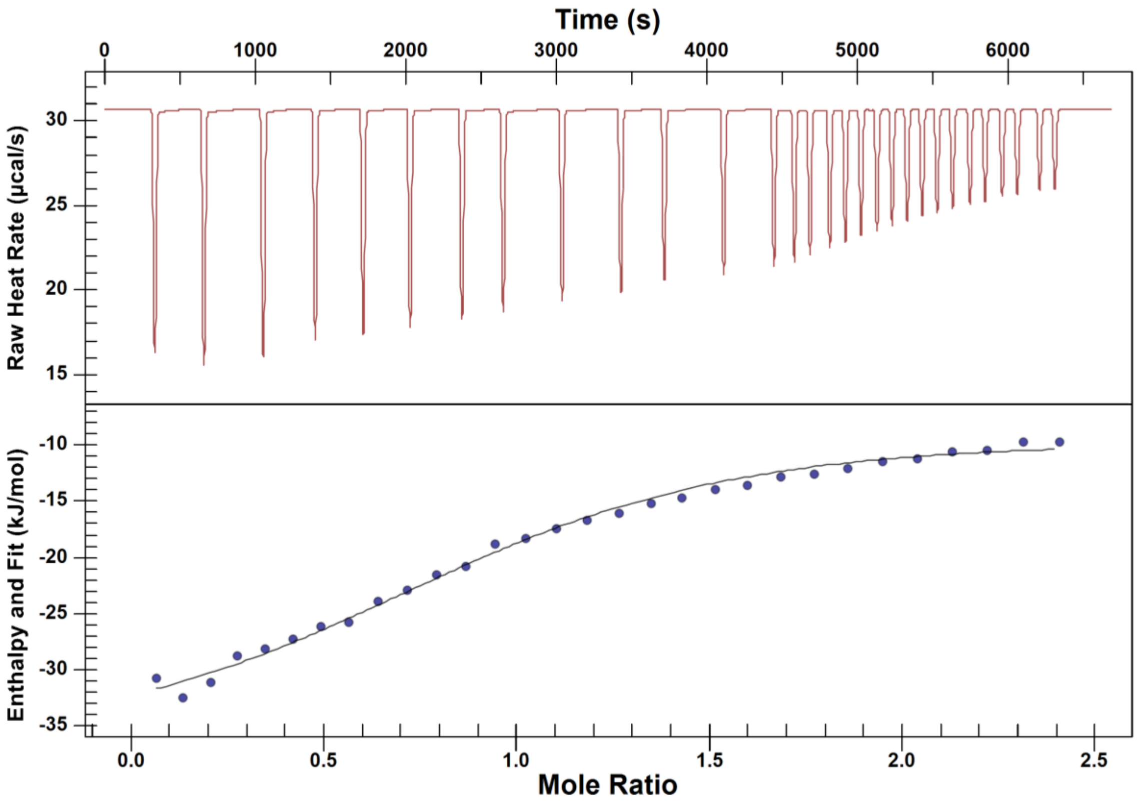

Figure S28: ITC heats of injection and binding isotherm of Capecitabine titrated with mPEG-FPBA Trial 2 conducted at 25 ^{o}C.

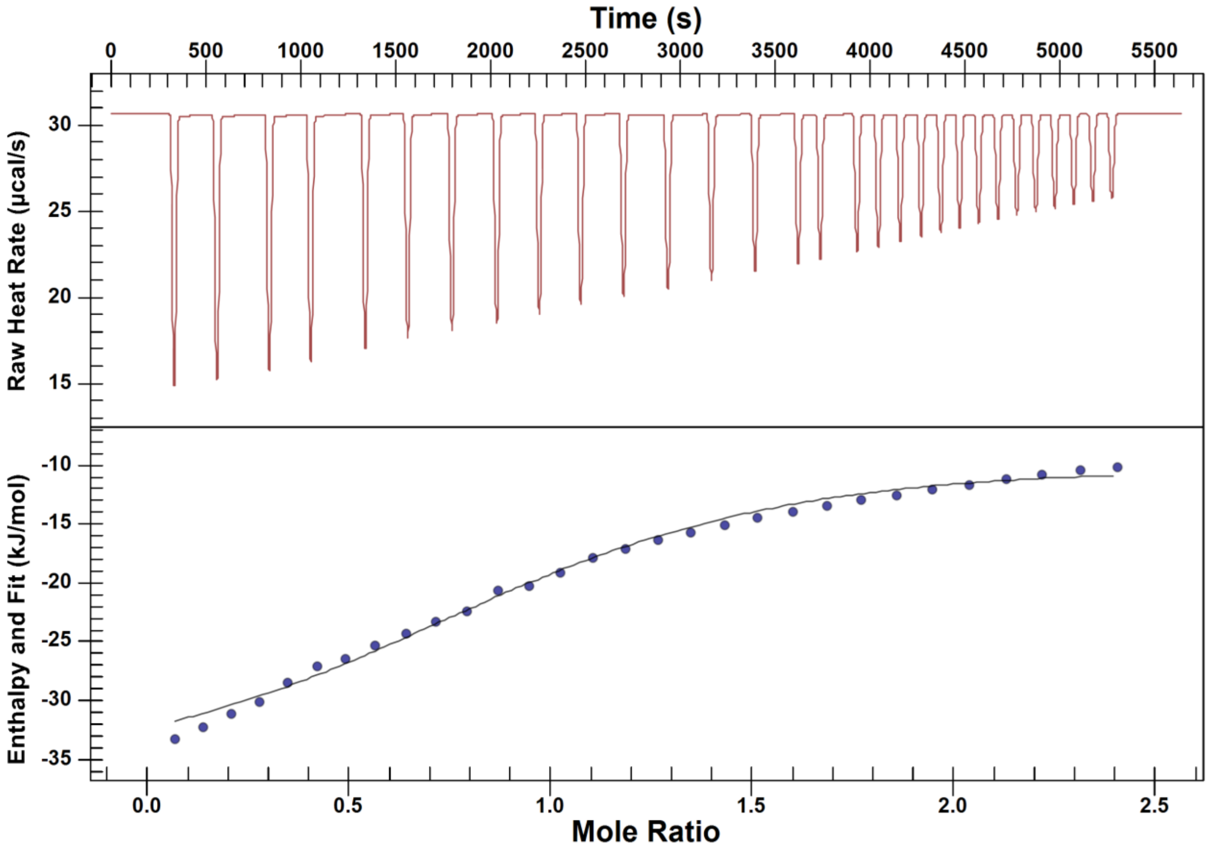

Figure S29: ITC heats of injection and binding isotherm of Capecitabine titrated with mPEG-FPBA Trial 3 conducted at 25 °C.

Table S4: Summary of ITC fits from capecitabine runs determined by kinITC[3].

KinITC	**Capecitabine**						
Trial	n	Kd (M)	Ka (M^{-1})	Ka std	koff ($M^{-1}s^{-1}$)	koff std	kon
1	1.00E+0	2.20E-3	4.55E+2	2.34E+1	1.22E+2	3.23E+1	5.53E+4
2	9.51E-1	2.15E-3	4.64E+2	1.79E+1	1.47E+2	1.76E+1	6.84E+4
3	9.79E-1	2.33E-3	4.28E+2	2.38E+1	1.27E+2	1.87E+1	5.42E+4
avg	9.77E-1	2.23E-3	4.49E+2		1.32E+2		5.93E+4
std	2.54E-2	9.43E-5	1.87E+1		1.37E+1		7.92E+3

mPEG-GA

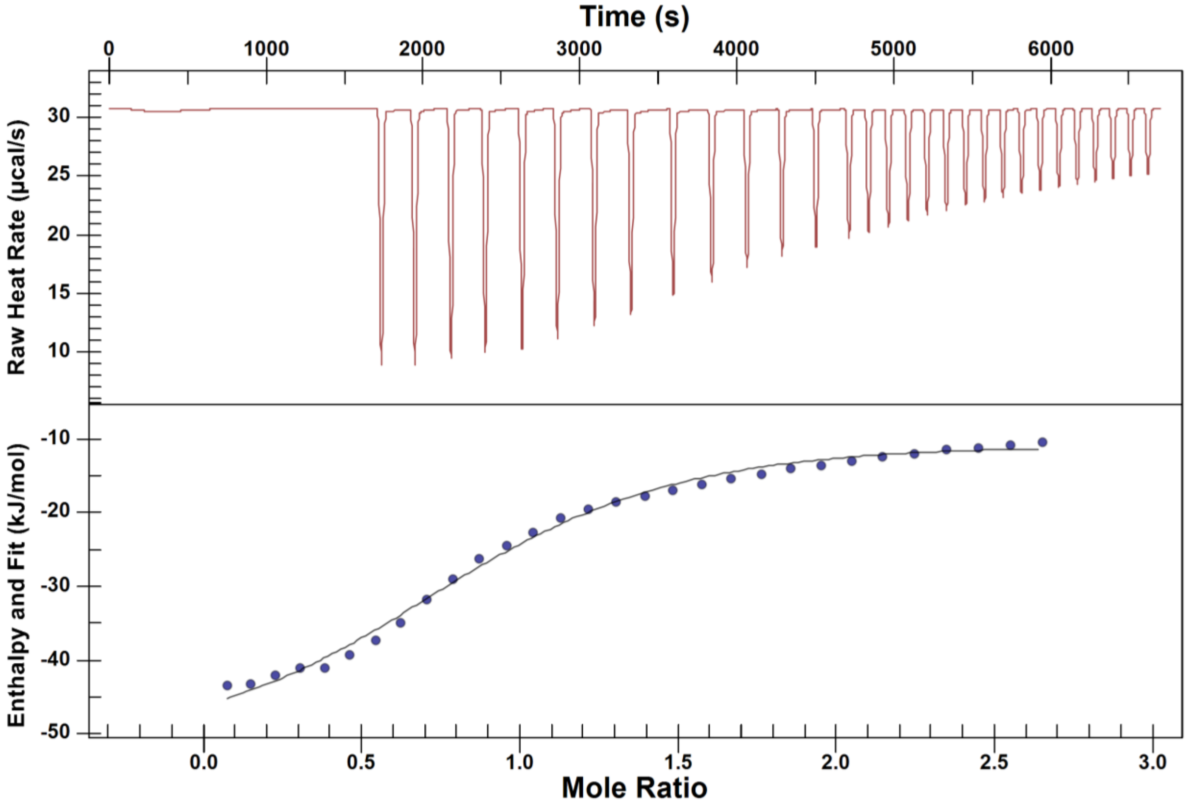

Figure S30: ITC heats of injection and binding isotherm of mPEG-GA titrated with mPEG-FPBA Trial 1 conducted at 25 ^{o}C.

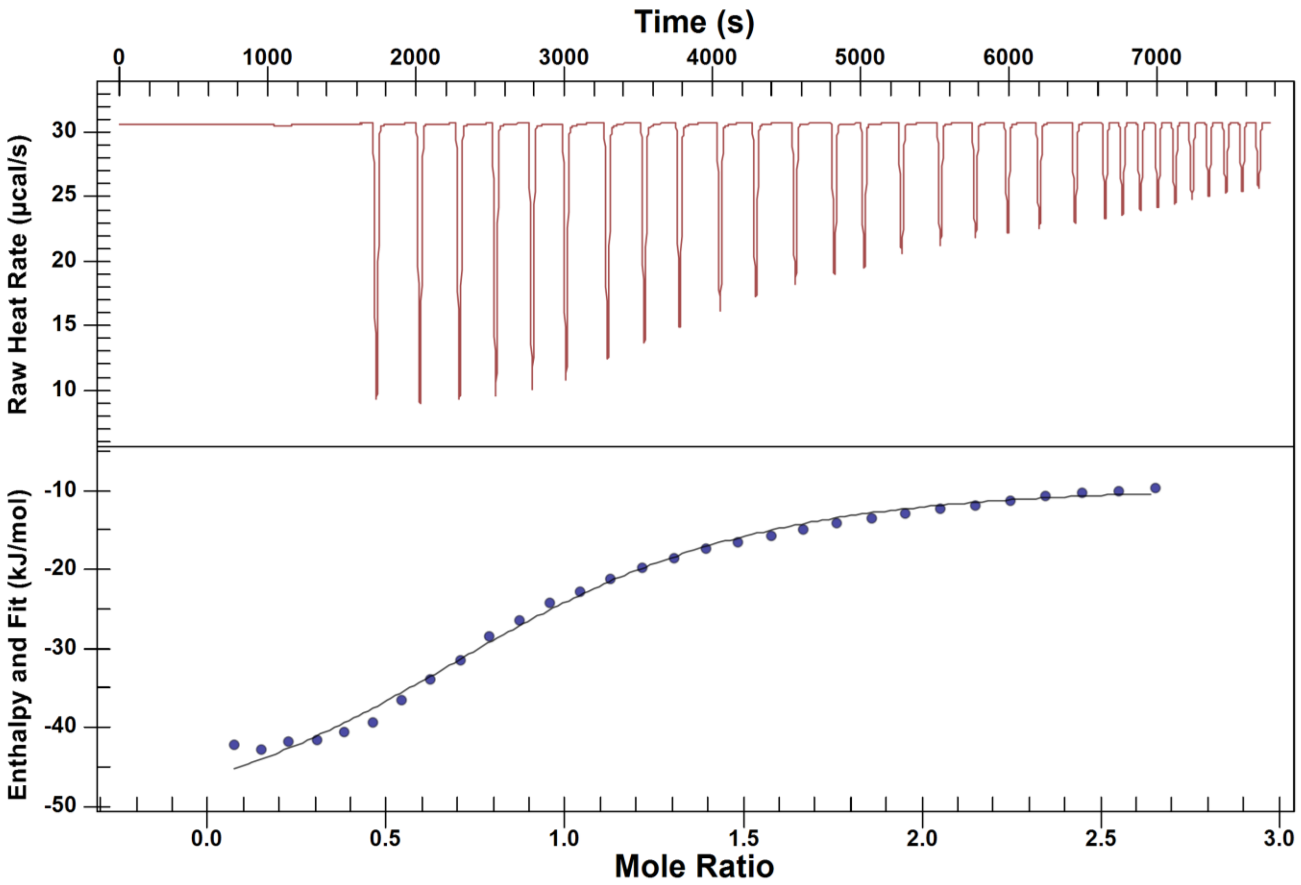

Figure S31: ITC heats of injection and binding isotherm of mPEG-GA titrated with mPEG-FPBA Trial 2 conducted at 25 ^{o}C.

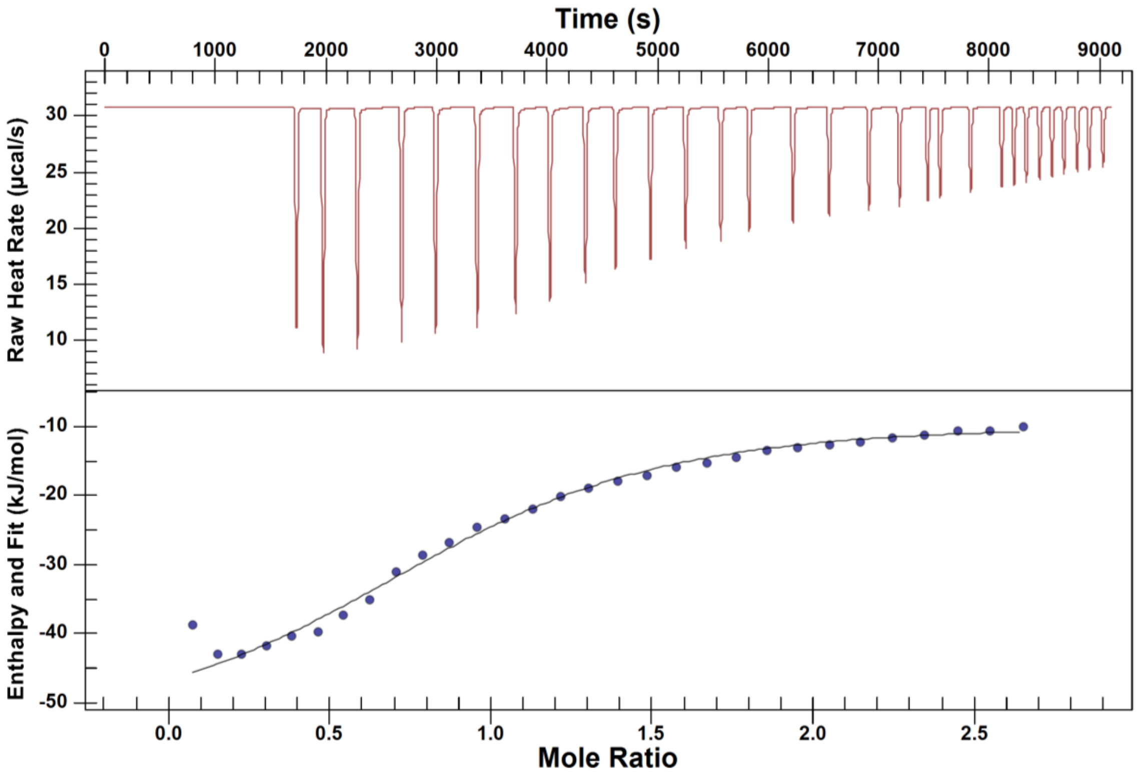

Figure S32: ITC heats of injection and binding isotherm of mPEG-GA titrated with mPEG-FPBA Trial 3 conducted at 25 ^{o}C.

Table S5: Summary of ITC fits from mPEG-GA runs determined by kinITC[3].

KinITC	mPEG-*GA*						
Trial	n	Kd (M)	Ka (M^{-1})	Ka std	koff ($M^{-1}s^{-1}$)	koff std	kon
1	8.77E-1	4.67E-4	2.14E+3	1.40E+1	2.04E-1	2.41E-2	4.36E+2
2	8.92E-1	4.35E-4	2.30E+3	1.91E+1	1.86E-1	7.05E-2	4.28E+2
3	1.01E+0	4.73E-4	2.11E+3	3.66E+1	3.85E-1	6.99E-2	8.15E+2
avg	9.25E-1	4.58E-4	2.18E+3		2.58E-1		5.60E+2
std	7.06E-2	2.06E-5	1.01E+2		1.10E-1		2.21E+2

Cl-ana (5)

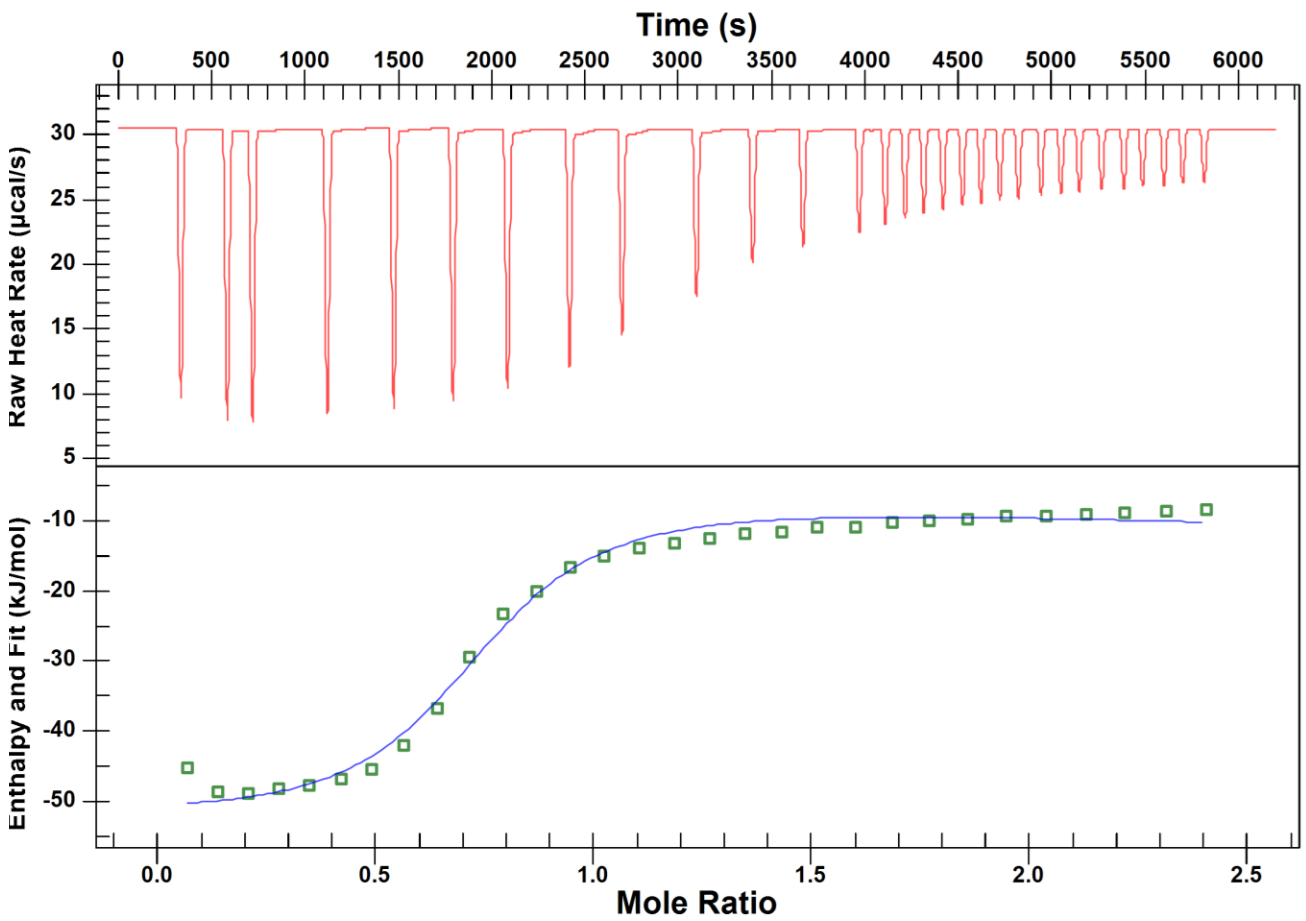

Figure S33: ITC heats of injection and binding isotherm of cl-ana titrated with mPEG-FPBA Trial 1 conducted at 25 °C.

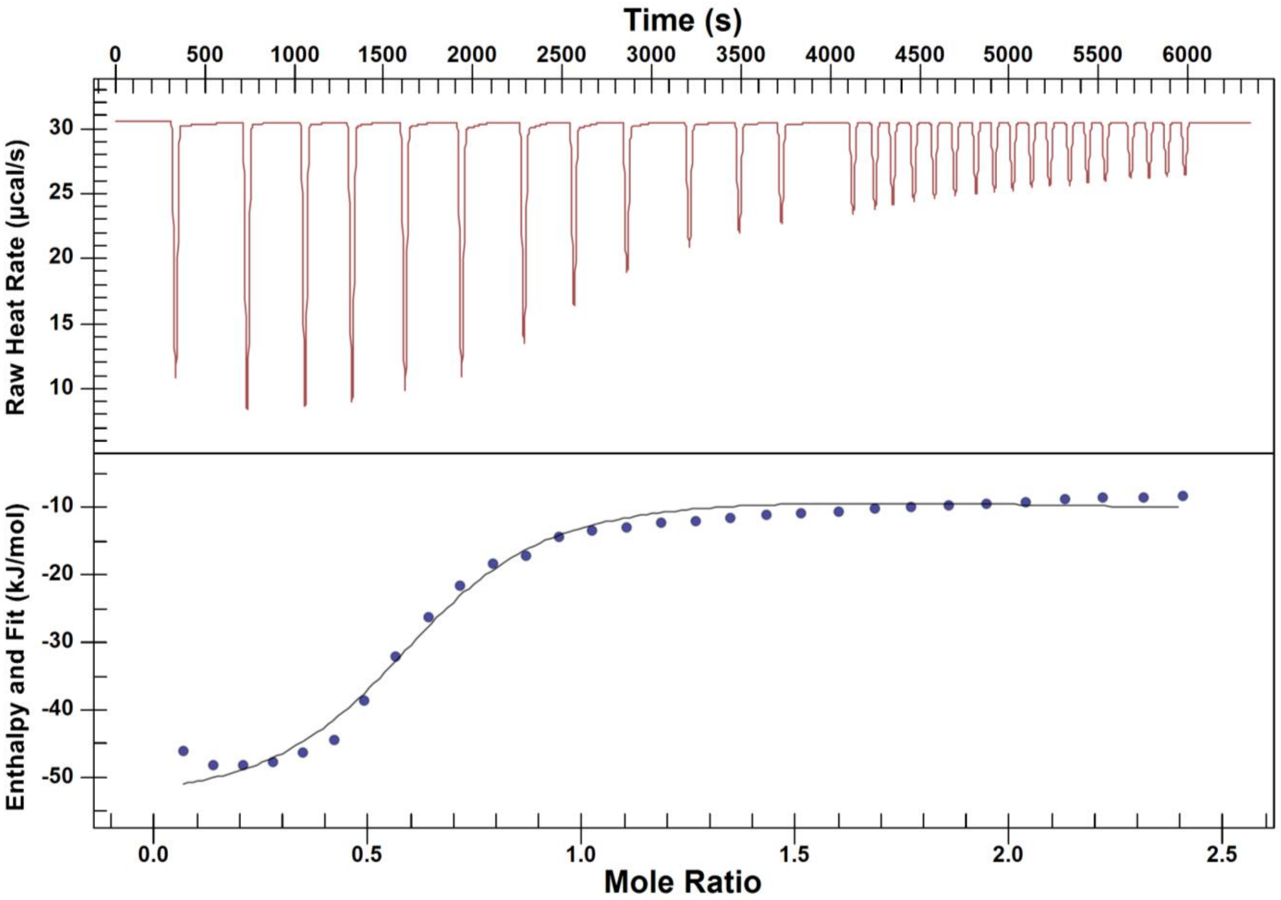

Figure S34: ITC heats of injection and binding isotherm of cl-ana titrated with mPEG-FPBA Trial 2 conducted at 25 °C.

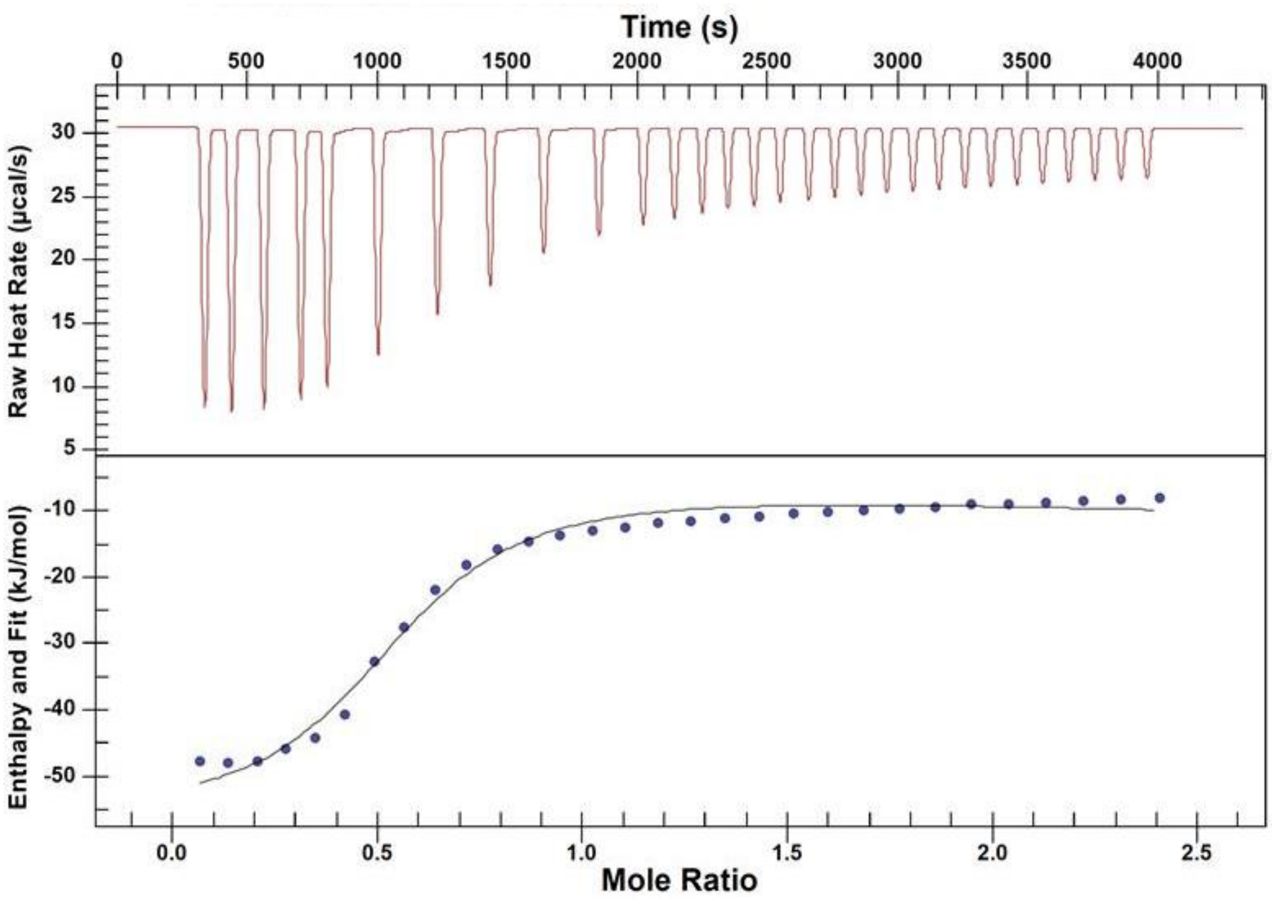

Figure S35: ITC heats of injection and binding isotherm of cl-ana titrated with mPEG-FPBA Trial 3 conducted at 25 °C.

Table S6: Summary of ITC fits from cl-ana runs determined by kinITC[3].

KinITC	*cl-ana*						
Trial	n	Kd (M)	Ka (M^{-1})	Ka std	koff ($M^{-1}s^{-1}$)	koff std	kon
1	7.28E-1	2.74E-4	3.65E+3	4.52E+1	1.11E-1	1.96E-2	4.04E+2
2	5.19E-1	1.96E-4	5.09E+3	9.30E+1	1.04E-1	2.24E-2	5.28E+2
3	6.74E-1	2.07E-4	4.83E+3	7.44E+1	1.01E-1	1.48E-2	4.89E+2
avg	6.40E-1	2.26E-4	4.52E+3		1.05E-1		4.74E+2
std	1.09E-1	4.21E-5	7.69E+2		4.92E-3		6.34E+1

Dopamine (6)

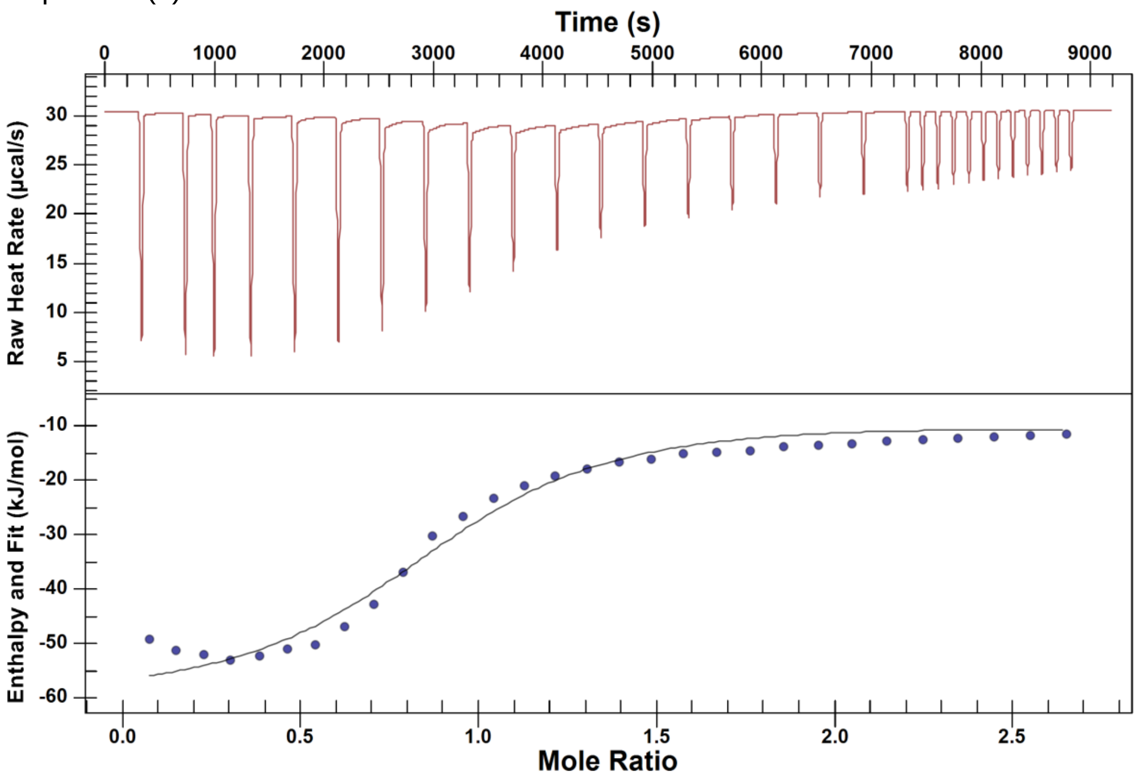

Figure S36: ITC heats of injection and binding isotherm of Dopamine titrated with mPEG-FPBA Trial 1conducted at 25 °C.

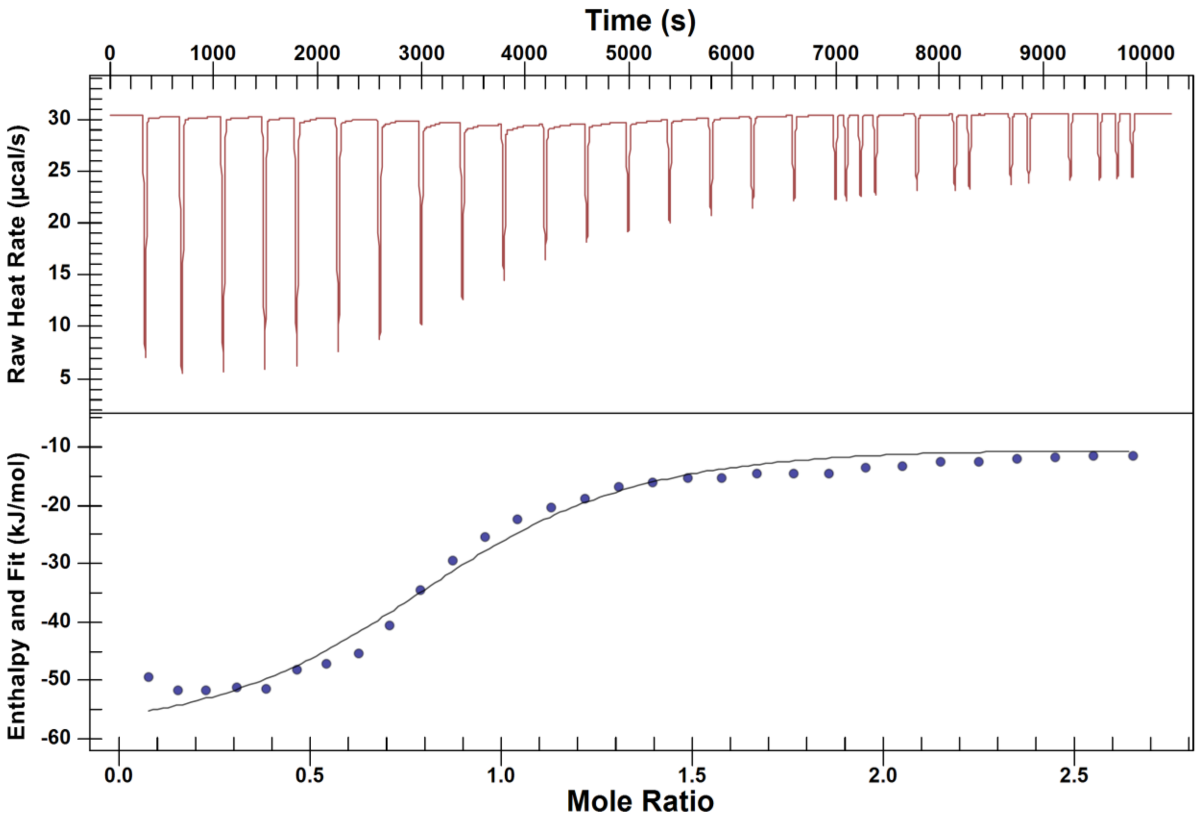

Figure S37: ITC heats of injection and binding isotherm of Dopamine titrated with mPEG-FPBA Trial 2 conducted at 25 °C.

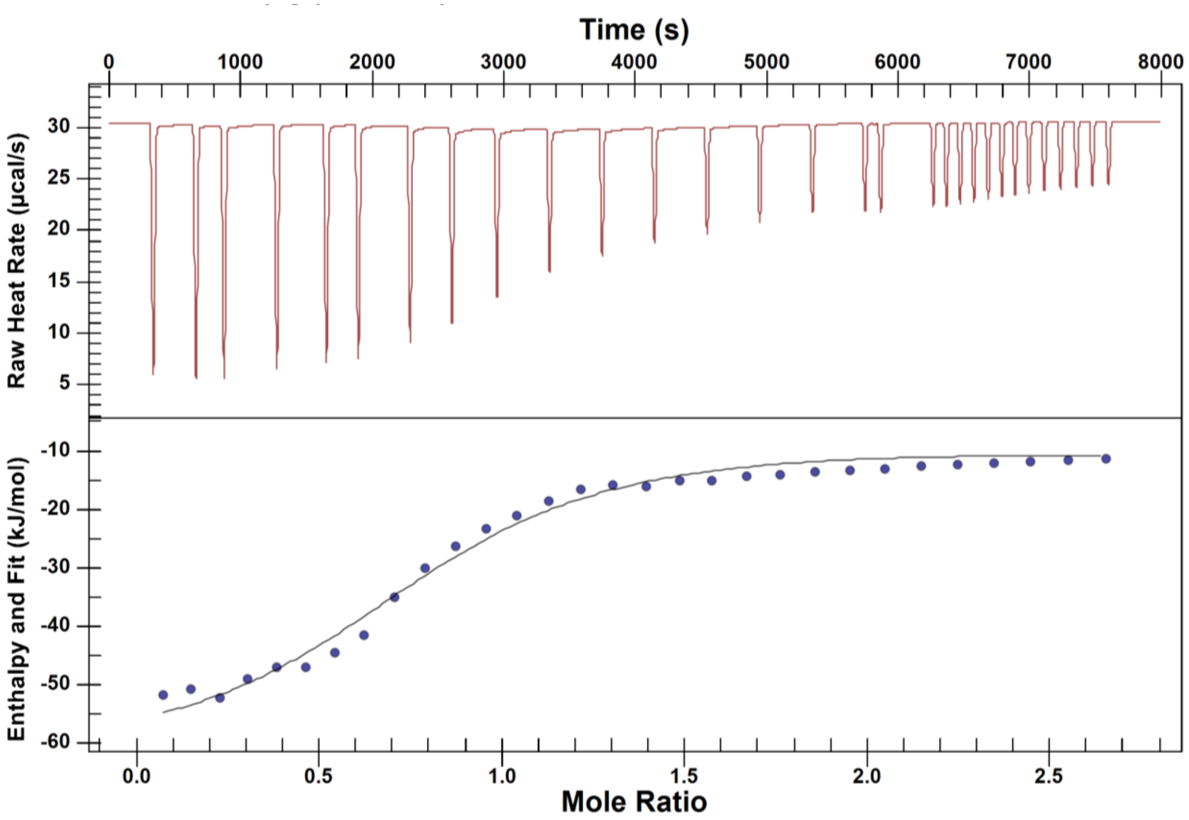

Figure S38: ITC heats of injection and binding isotherm of Dopamine titrated with mPEG-FPBA Trial 3 conducted at 25 ^{o}C.

Table S7: Summary of ITC fits from dopamine runs determined by kinITC[3].

KinITC	**Dopamine**						
Trial	n	Kd (M)	Ka (M^{-1})	Ka std	koff ($M^{-1}s^{-1}$)	koff std	kon
1	6.87E-1	1.34E-4	7.47E+3	7.38E+1	7.64E-2	1.05E-2	5.71E+2
2	7.46E-1	1.62E-4	6.17E+3	1.51E+2	6.08E-2	1.81E-2	3.76E+2
3	7.18E-1	1.52E-4	6.58E+3	1.25E+2	4.52E-2	5.88E-3	2.98E+2
avg	7.17E-1	1.49E-4	6.74E+3		6.08E-2		4.15E+2
std	2.93E-2	1.42E-5	6.63E+2		1.56E-2		1.41E+2

ITC Data Summary

Table S8: Summary of thermodynamics and kinetics of boronate ester system

Diol	K_a (M^{-1})	k_{off} (s^{-1})
mPEG-GA	2200 ± 100	0.26 ±0.11
Glucose (1)	6.2 ± 3.2	1.47 ± 0.88
Dyphylline (2)	26.0 ± 3.8	238 ± 69
Tris (3)	310 ± 60	164 ± 39
Capecitabine (4)	450 ± 20	131.8 ± 14
Crosslinker analogue (5)	4530 ± 60	0.105 ± 0.005
Dopamine (6)	6700 ±600	0.06 ± 0.01

Rheology

Formation of Hydrogels:

To synthesize dynamic hydrogels for the boronate ester system, 10 weight/volume percent (w/v%) solutions of 4PEG-phenylboronic acid and 4PEG-GA were prepared in 0.1 M HEPES buffer at pH 7.4 ± 0.1. We avoided PBS buffer due to its potential to form competing complexes via Lewis adduct formation between phosphates and boronic acid.[20] Upon mixing the 10 w/v% solutions, hydrogels formed within 3 seconds. For the inhibited gels, 4PEG-GA was dissolved at 10 w/v% and pre-mixed with the diol competitor to prevent pre-association with the boronic acid. To ensure the gels were fully equilibrated a time sweep at 10 rad/s at 1% strain for 300 seconds was performed to show there was constant modulus. See pictured gels in figure S39.

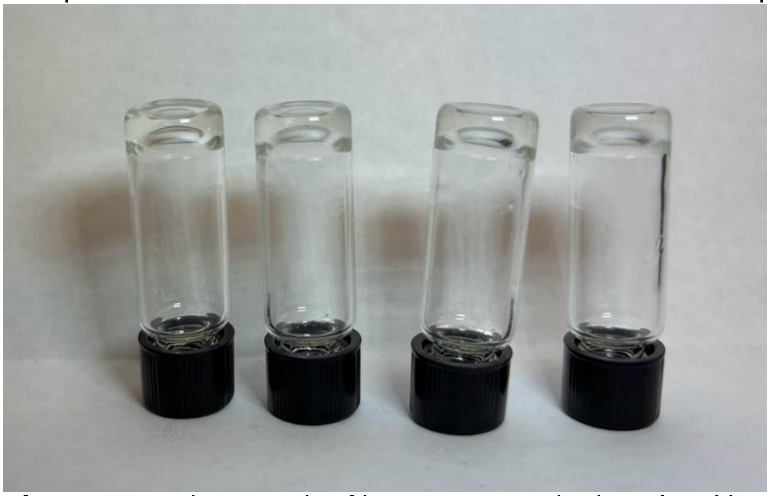

Figure S39: Photograph of boronate ester hydrogels with 0, 20, 30, and 40 mM (from left to right) of competitor (2). All gels were optically clear.

A similar procedure was used for the hydrazone network. Initially, 4PEG-Ar-CHO and 4PEG-Hz were prepared as 0.01 M PBS buffer solutions at pH 7.4 ± 0.1. However, due to the inherent basic pH of the MeHz competitor (e.g. the pH of 18mM MeHz in PBS 1X buffer is about 9.0), pH adjustment is required to keep the hydrogel at a constant pH of 7.4 ± 0.1. The stock solution of MeHz was prepared at 180 mM in PBS 1X, with a pH of about 9.60. Reagent grade HCl was used to adjust the pH to 7.4 ± 0.1. Then, sodium chloride solution (0.037 M in PBS 1X) was added, so that the total saline ionic concentration (excluding PBS 1X) is 0.195 M. The ionic contribution of the PBS 1X is ignored and assumed to be constant throughout the solutions given that it is used as a diluting solvent for all solutions, including the sodium chloride solution. Finally, the volume of MeHz is diluted to 180 mM using PBS 1X. It is important to limit variations in the procedure for preparing hydrogels to achieve consistent rheological measurements.

For the hydrazone system 200 µL gels (72 mM functional groups for both in a final saline concentration of 0.0975 M NaCl in PBS 1X) were prepared by separately dissolving 4PEG-Ar-CHO 18 mM (in 100 µL PBS 1X) and 4PEG-Hz 18 mM (in 100 µL 0.195 M NaCl solution in PBS 1X) followed by mixing and vortexing the solutions. Gels were then allowed to sit at room temperature until gelation occurred. For the competitive hydrogel, the MeHz competitor was added to the solution of 4APEG-Hz. The ionic saline concentration of this 4PEG-Hz solution (0.195 M NaCl) is always kept constant by compensating for the dilution with PBS buffer by adding NaCl solution (0.195 M). For example, MeHz (27 mM with 0.195 M NaCl in PBS 1X) will need 30 µL of MeHz stock solution (180 mM MeHz with 0.195 M NaCl in PBS 1X) and compensated with 70 µL of dilute NaCl solution (0.195 M), yielding a final 100 µL of 0.195 M NaCl concentration. The 4PEG-hydrazine and 4PEG-Ar-CHO were prepared as 9 w/v% solutions and mixed, with

gelation occurring in a minute. The gels were allowed to equilibrate overnight before measurements because the slow kinetics of the hydrazone formation.

Zero Competitor

Table S9: Summary of 0 competitor rheology results.

Run	Gp (kPa)	Cross Freq (rad /s)	τ (s) from crossover
1	21.5	1.35	0.741
2	21.6	1.34	0.746
3	21.9	1.44	0.694
Average	21.7	1.38	0.727
std	0.192	0.0565	0.029

Glucose (1)

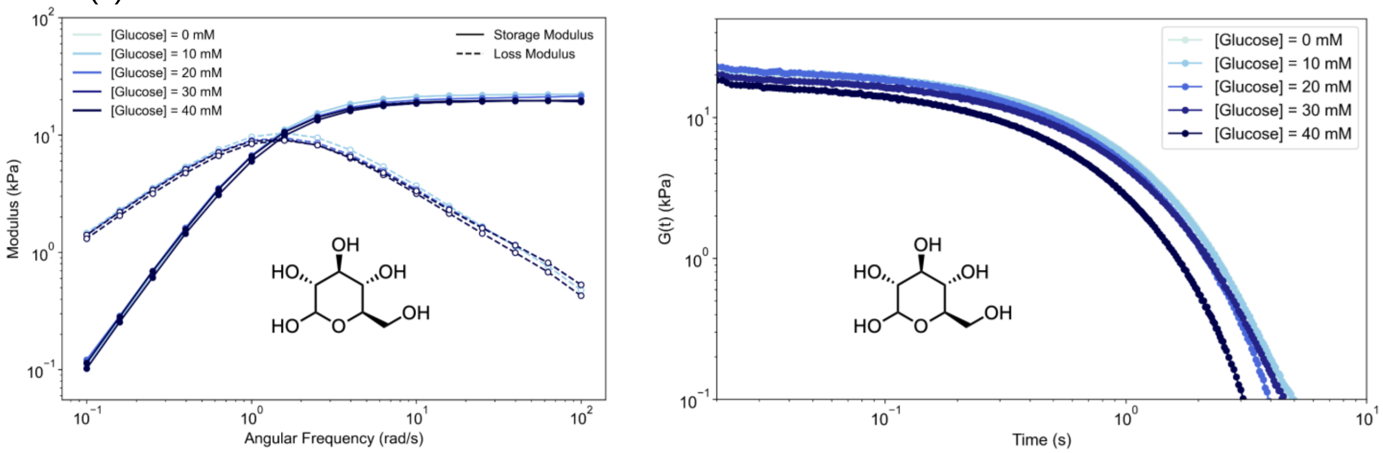

Figure S40: Frequency-dependent storage (solid line) and loss modulus (dashed line) from 0.1 rad/s – 100 rad/s, 1% strain and stress relaxation profiles at 5% strain of glucose competitor (1) added to boronate ester hydrogel at concentrations 0 – 40 mM at 25 °C.

Table S10: Summary of glucose rheology results.

Glucose	10 mM		
Run	Gp (kPa)	Cross Freq (rad /s)	τ (s) from crossover
1	21.2	1.48	0.676
2	20.6	1.41	0.709
3	21.6	1.38	0.725
Average	21.1	1.42	0.704
std	0.481	0.0494	0.025
Glucose	20 mM		
Run	Gp (kPa)	Cross Freq (rad /s)	τ (s) from crossover
1	20.1	1.38	0.725
2	21.1	1.37	0.730
3	21.0	1.49	0.671
Average	20.7	1.41	0.709
std	0.566	0.0667	0.033
Glucose	30 mM		
Run	Gp (kPa)	Cross Freq (rad /s)	τ (s) from crossover
1	19.6	1.42	0.704
2	17.9	1.46	0.685
3	20.5	1.44	0.694
Average	19.3	1.44	0.694
std	1.35	0.0216	0.010
Glucose	40 mM		
Run	Gp (kPa)	Cross Freq (rad /s)	τ (s) from crossover
1	20.5	1.36	0.735
2	21.7	1.80	0.556
3	18.6	1.77	0.565
Average	20.2	1.65	0.606
std	1.55	0.247	0.101

Dyphylline (2)

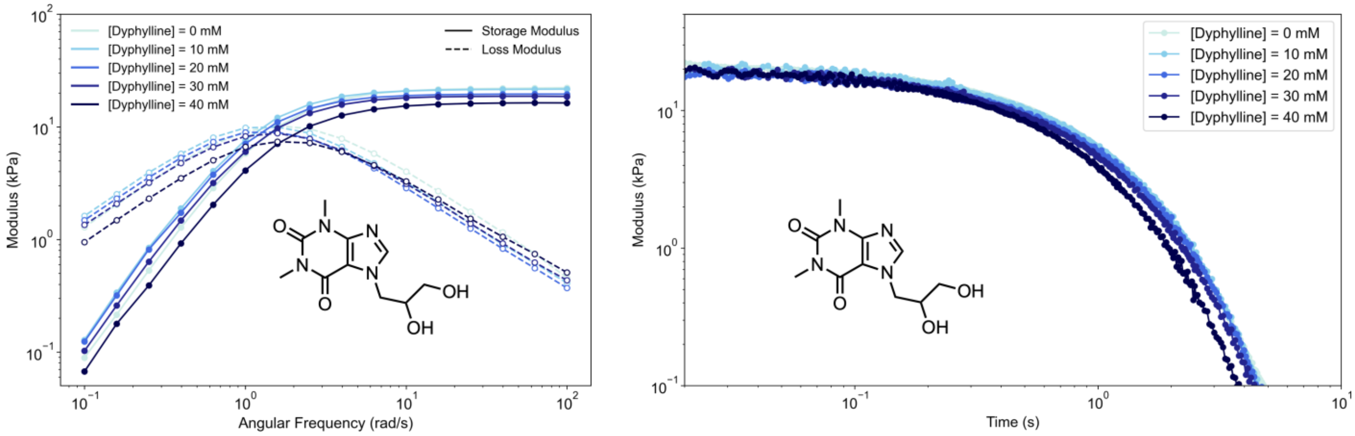

Figure S41: Frequency-dependent storage (solid line) and loss modulus (dashed line) from 0.1 rad/s – 100 rad/s, 1% strain and stress relaxation profiles at 5% strain of dyphylline competitor (2) added to boronate ester hydrogel at concentrations 0 – 40 mM at 25 °C.

Table S11: Summary of dyphylline rheology results.

Dyphylline	10 mM		
Run	Gp (kPa)	Cross Freq (rad /s)	τ (s) from crossover
1	21.6	1.38	0.725
2	20.5	1.28	0.781
3	22.9	1.30	0.769
Average	21.7	1.32	0.758
std	1.22	0.0539	0.030
Dyphylline	20 mM		
Run	Gp (kPa)	Cross Freq (rad /s)	τ (s) from crossover
1	17.5	1.38	0.725
2	20.6	1.30	0.769
3	20.0	1.46	0.685
Average	19.4	1.38	0.725
std	1.68	0.0820	0.042
Dyphylline	30 mM		
Run	Gp (kPa)	Cross Freq (rad /s)	τ (s) from crossover
1	16.7	1.31	0.763
2	19.3	1.41	0.709
3	20.1	1.37	0.730
Average	18.7	1.36	0.735
std	1.77	0.0524	0.027
Dyphylline	40 mM		
Run	Gp (kPa)	Cross Freq (rad /s)	τ (s) from crossover
1	16.2	1.58	0.633
2	18.9	1.71	0.585
3	20.1	1.63	0.613
Average	18.4	1.64	0.610
std	1.98	0.0652	0.024

Tris (3)

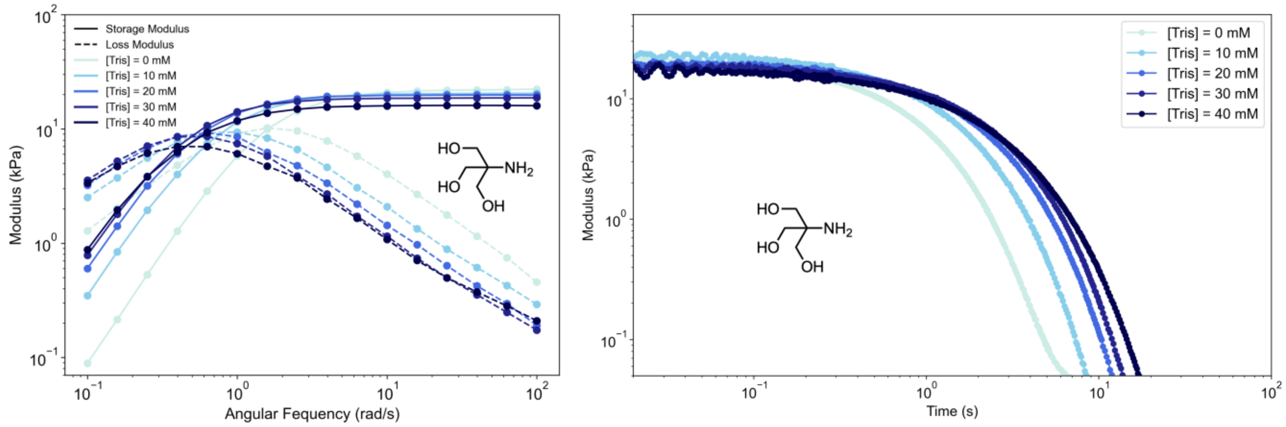

Figure S42: Frequency-dependent storage (solid line) and loss modulus (dashed line) from 0.1 rad/s – 100 rad/s, 1% strain and stress relaxation profiles at 5% strain of tris competitor (3) added to boronate ester hydrogel at concentrations 0 – 40 mM at 25 °C.

Table S12: Summary of tris rheology results.

Tris	10 mM		
Run	Gp (kPa)	Cross Freq (rad /s)	τ (s) from crossover
1	16.9	0.884	1.13
2	19.6	0.796	1.26
3	21.5	0.885	1.13
Average	19.3	0.855	1.17
std	2.29	0.0511	0.07
Tris	20 mM		
Run	Gp (kPa)	Cross Freq (rad /s)	τ (s) from crossover
1	16.2	0.623	1.61
2	19.3	0.583	1.72
3	20.7	0.677	1.48
Average	18.7	0.627	1.59
std	2.31	0.0474	0.12
Tris	30 mM		
Run	Gp (kPa)	Cross Freq (rad /s)	τ (s) from crossover
1	15.6	0.532	1.88
2	18.3	0.497	2.01
3	18.3	0.478	2.09
Average	17.4	0.503	1.99
std	1.58	0.0274	0.11
Tris	40 mM		
Run	Gp (kPa)	Cross Freq (rad /s)	τ (s) from crossover
1	13.4	0.479	2.09
2	15.4	0.453	2.21
3	15.1	0.440	2.27
Average	14.6	0.458	2.18
std	1.10	0.0197	0.09

Capecitabine (4)

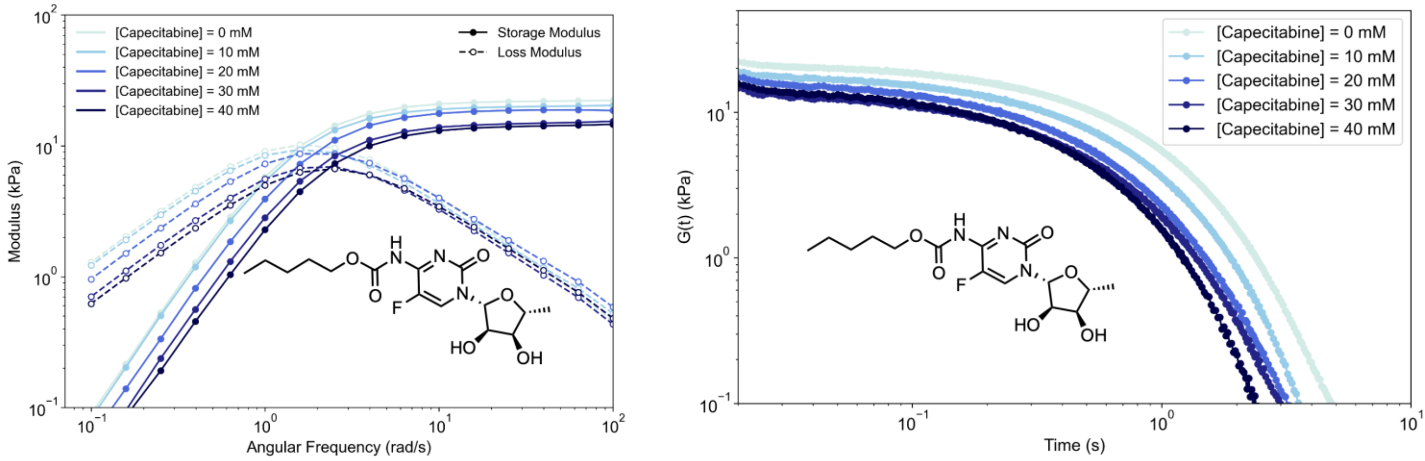

Figure S43: Frequency-dependent storage (solid line) and loss modulus (dashed line) from 0.1 rad/s – 100 rad/s, 1% strain and stress relaxation profiles at 5% strain of capecitabine competitor (4) added to boronate ester hydrogel at concentrations 0 – 40 mM at 25 °C.

Table S13: Summary of capecitabine rheology results.

Capecitabine	10 mM		
Run	Gp (kPa)	Cross Freq (rad /s)	τ (s) from crossover
1	18.6	1.55	0.645
2	18.9	1.60	0.625
3	17.1	1.73	0.578
Average	18.2	1.63	0.613
std	1.01	0.0940	0.034
Capecitabine	20 mM		
Run	Gp (kPa)	Cross Freq (rad /s)	τ (s) from crossover
1	15.7	1.98	0.505
2	15.8	1.59	0.629
3	13.4	1.86	0.538
Average	15.0	1.81	0.552
std	1.32	0.198	0.064
Capecitabine	30 mM		
Run	Gp (kPa)	Cross Freq (rad /s)	τ (s) from crossover
1	11.3	1.89	0.529
2	13.8	2.03	0.493
3	12.5	1.97	0.508
Average	12.5	1.96	0.510
std	1.25	0.0716	0.018
Capecitabine	40 mM		
Run	Gp (kPa)	Cross Freq (rad /s)	τ (s) from crossover
1	12.9	2.28	0.439
2	10.8	2.13	0.469
3	11.0	2.16	0.463
Average	11.6	2.19	0.457
std	1.16	0.0773	0.016

Crosslink-analogue (cl-ana) (5)

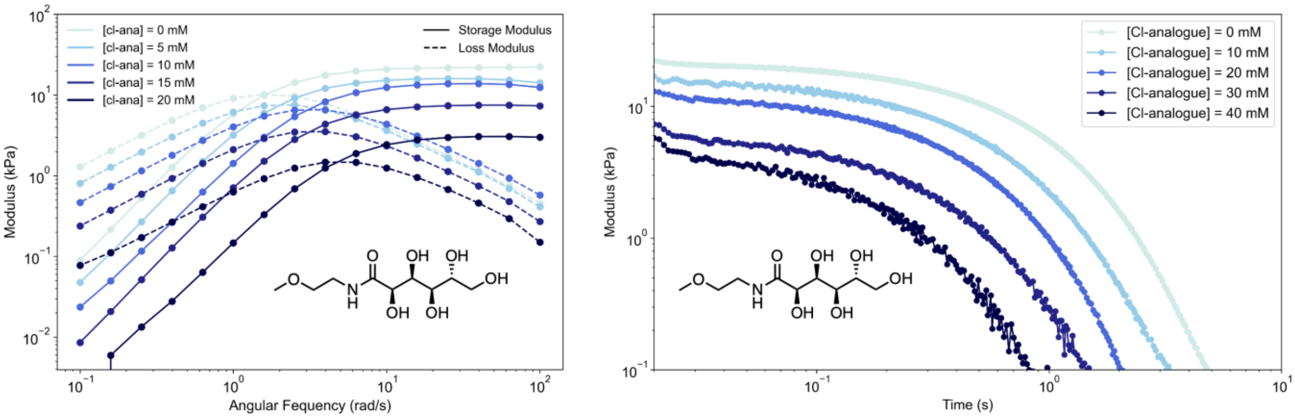

Figure S44: Frequency-dependent storage (solid line) and loss modulus (dashed line) from 0.1 rad/s – 100 rad/s, 1% strain and stress relaxation profiles at 5% strain of cl-ana competitor (5) added to boronate ester hydrogel at concentrations 0 – 20 mM at 25 °C.

Table S14: Summary of cl-ana rheology results.

Cl-ana	5 mM		
Run	Gp (kPa)	Cross Freq (rad /s)	τ (s) from crossover
1	13.5	1.91	0.524
2	11.0	2.02	0.495
3	13.2	2.01	0.498
Average	12.6	1.98	0.505
std	1.40	0.0609	0.016
Cl-ana	10 mM		
Run	Gp (kPa)	Cross Freq (rad /s)	τ (s) from crossover
1	10.6	3.07	0.326
2	9.35	2.71	0.369
3	9.81	2.56	0.391
Average	9.93	2.78	0.360
std	0.654	0.259	0.033
Cl-ana	15 mM		
Run	Gp (kPa)	Cross Freq (rad /s)	τ (s) from crossover
1	4.99	3.37	0.297
2	5.23	3.13	0.319
3	6.60	3.16	0.316
Average	5.61	3.22	0.311
std	0.871	0.131	0.012
Cl-ana	20 mM		
Run	Gp (kPa)	Cross Freq (rad /s)	τ (s) from crossover
1	2.42	4.77	0.210
2	2.54	5.16	0.194
3	2.86	5.49	0.182
Average	2.60	5.14	0.195
std	0.227	0.360	0.014

Dopamine (6)

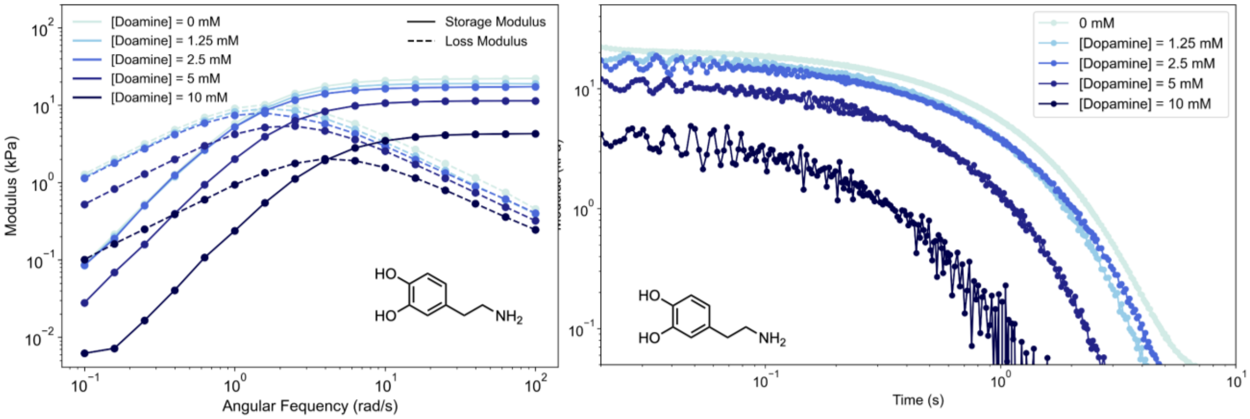

Figure S45: Frequency-dependent storage (solid line) and loss modulus (dashed line) from 0.1 rad/s – 100 rad/s, 1% strain and stress relaxation profiles at 5% strain of dopamine competitor (6) added to boronate ester hydrogel at concentrations 0 – 10 mM at 25 °C.

Table S15: Summary of dopamine rheology results.

Dopamine	1.25 mM		
Run	Gp (kPa)	Cross Freq (rad /s)	τ (s) from crossover
1	13.6	1.41	0.709
2	15.1	1.35	0.741
3	15.3	1.54	0.649
4	16.3	1.46	0.685
Average	15.1	1.44	0.694
std	1.11	0.0808	0.039
Dopamine	2.5 mM		
Run	Gp (kPa)	Cross Freq (rad /s)	τ (s) from crossover
1	16.3	1.61	0.621
2	16.4	1.61	0.621
3	18.2	1.58	0.633
4	17.7	1.56	0.641
Average	17.2	1.59	0.629
std	0.936	0.0229	0.010
Dopamine	5 mM		
Run	Gp (kPa)	Cross Freq (rad /s)	τ (s) from crossover
1	10.1	2.21	0.452
2	10.5	2.20	0.455
3	10.4	2.11	0.474
4	11.2	2.20	0.455
Average	10.6	2.16	0.463
std	0.463	0.0526	0.010
Dopamine	10 mM		
Run	Gp (kPa)	Cross Freq (rad /s)	τ (s) from crossover
1	3.45	4.19	0.239
2	3.50	4.21	0.238
3	3.63	4.04	0.248
Average	3.53	4.15	0.241
std	0.0911	0.0971	0.005

Addition of Competitive Inhibitor Post-Gelation:

To test whether the material properties of the gel could be altered after the formation of a gel we first formed a gel by mixing 100 µL of each polymer solution. Following this competitor was deposited on top of the gel.

1.2 mg (in 200 µL gives concentration of 32 mM) of (6) was weighed and placed in the vial. Within 1 minute the solid was dissolved into the gel. Within 2 minutes the gel was completely dissolved and in the solution phase.

2.9 mg (in 200 µL gives concentration of 40 mM) of (4) was weighed and placed in the vial. Within 1 minute the solid was dissolved into the gel. The gel was then taken to the rheometer. The crossover frequency was found to be 2.28 rad/s, and the G_p was found to be 11.1 kPa (figure S45). We anticipate greater variability in this approach as there is more uncertainty weighing out small masses of competitive inhibitors, compared to making stock solutions at the desired concentration.

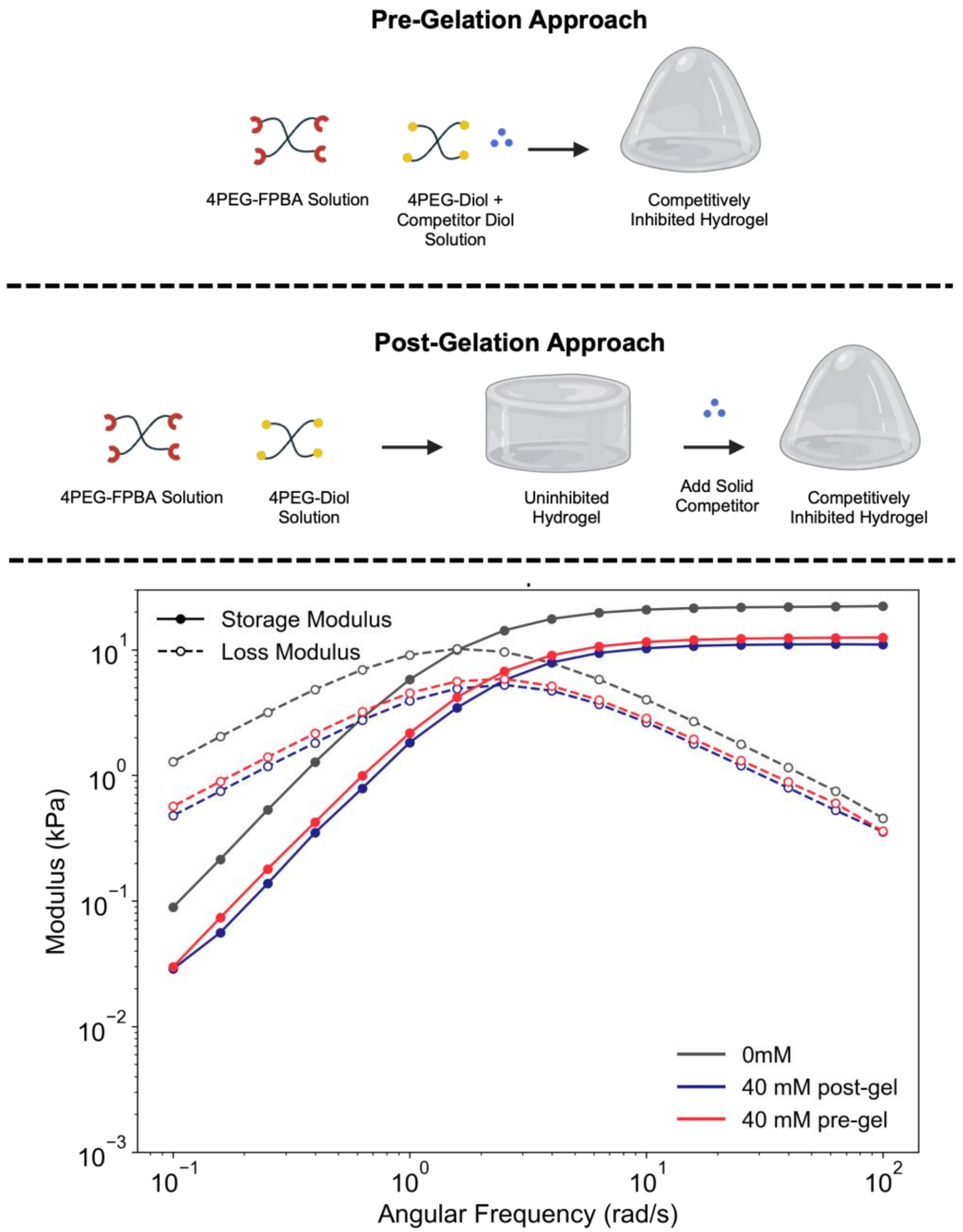

Figure S46: Comparison of pre- and post-gelation strategies for competitive inhibition of boronate ester hydrogels. (A) Pre-gelation inhibition: the hydrogel is formed by dissolving 4PEG-diol in a stock solution containing the competitor prior to mixing and network formation. (B) Post-gelation inhibition: an uninhibited gel is first formed, after which solid competitor is placed on top of the gel and allowed to dissolve and diffuse into the network. (C) Frequency-dependent storage (solid symbols) and loss (open symbols) moduli for an uninhibited gel (gray), a gel inhibited post-gelation (blue), and a gel inhibited pre-gelation (red) from 0.1 rad/s – 100 rad/s, 1% strain conducted at 25 ^{o}C.

Modulus Predictions

Predicting Modulus From K_a crosslink and K_a of competitor

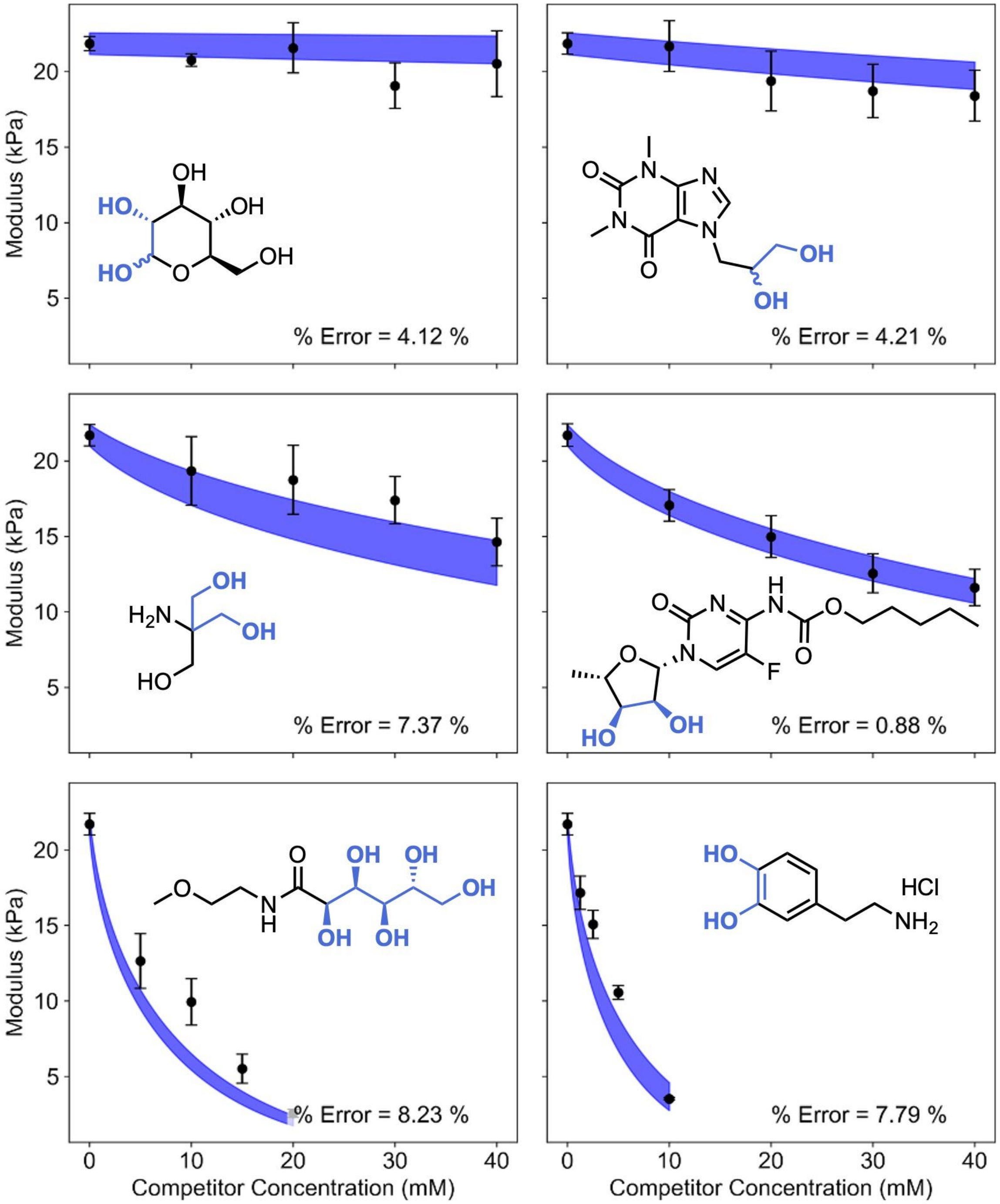

Figure S47: Experimental (black dots with error bars representing standard deviation from triplicate measurements of G_p) and predicted (blue) moduli of boronate ester gels vs concentration of all 6 competitors. The upper and lower limits of the prediction were calculated based on the standard deviation from ITC triplicate measurements.

Fitting for $K_{a,XL}$ from known $K_{a,C}$

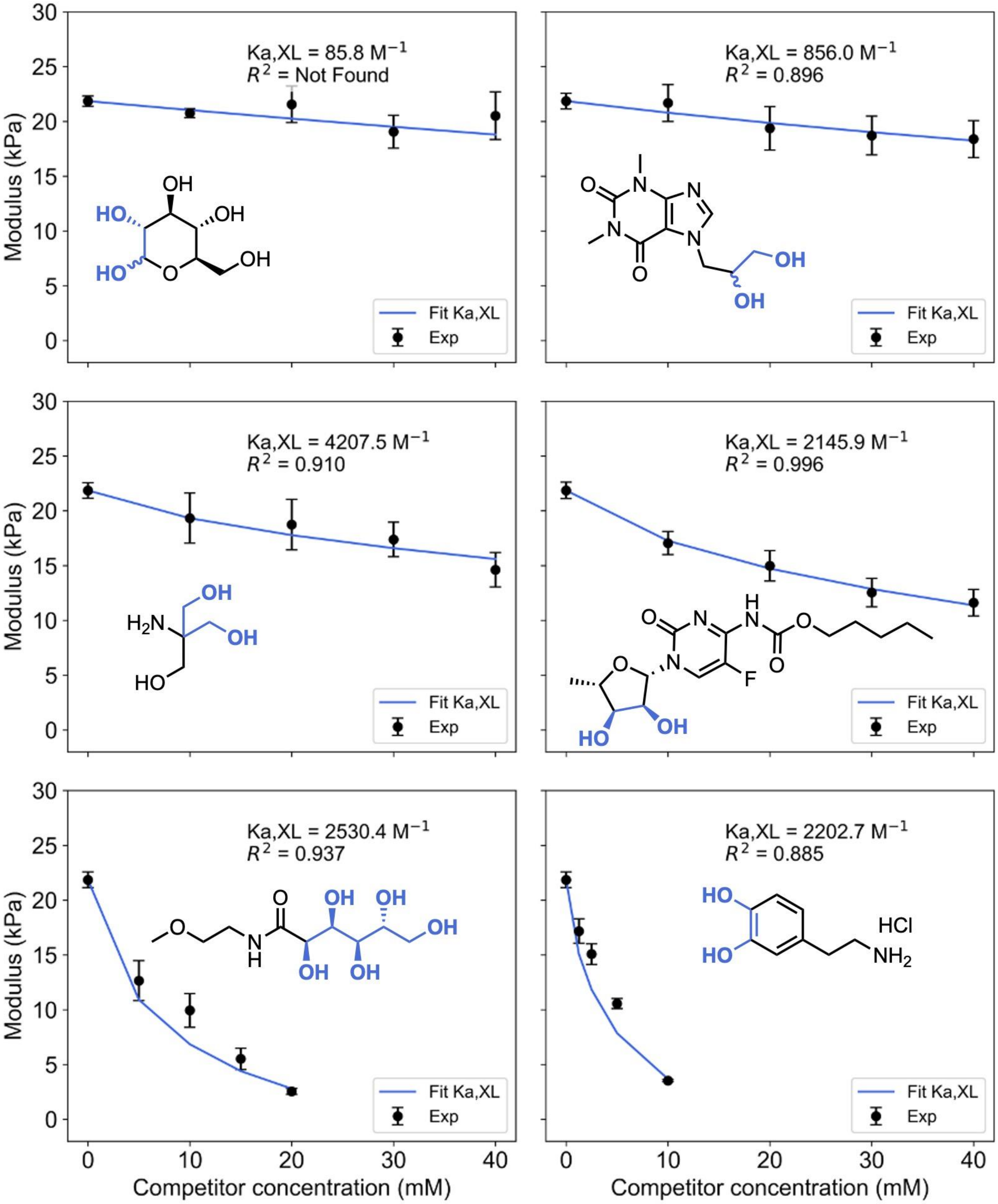

Figure S48: Fitting the modulus vs competitor concentration of all 6 competitors using equations S10, S14, and S20 to fit for the $K_{a,XL}$ of the system.

Fitting for $K_{a,C}$ from known $K_{a,XL}$

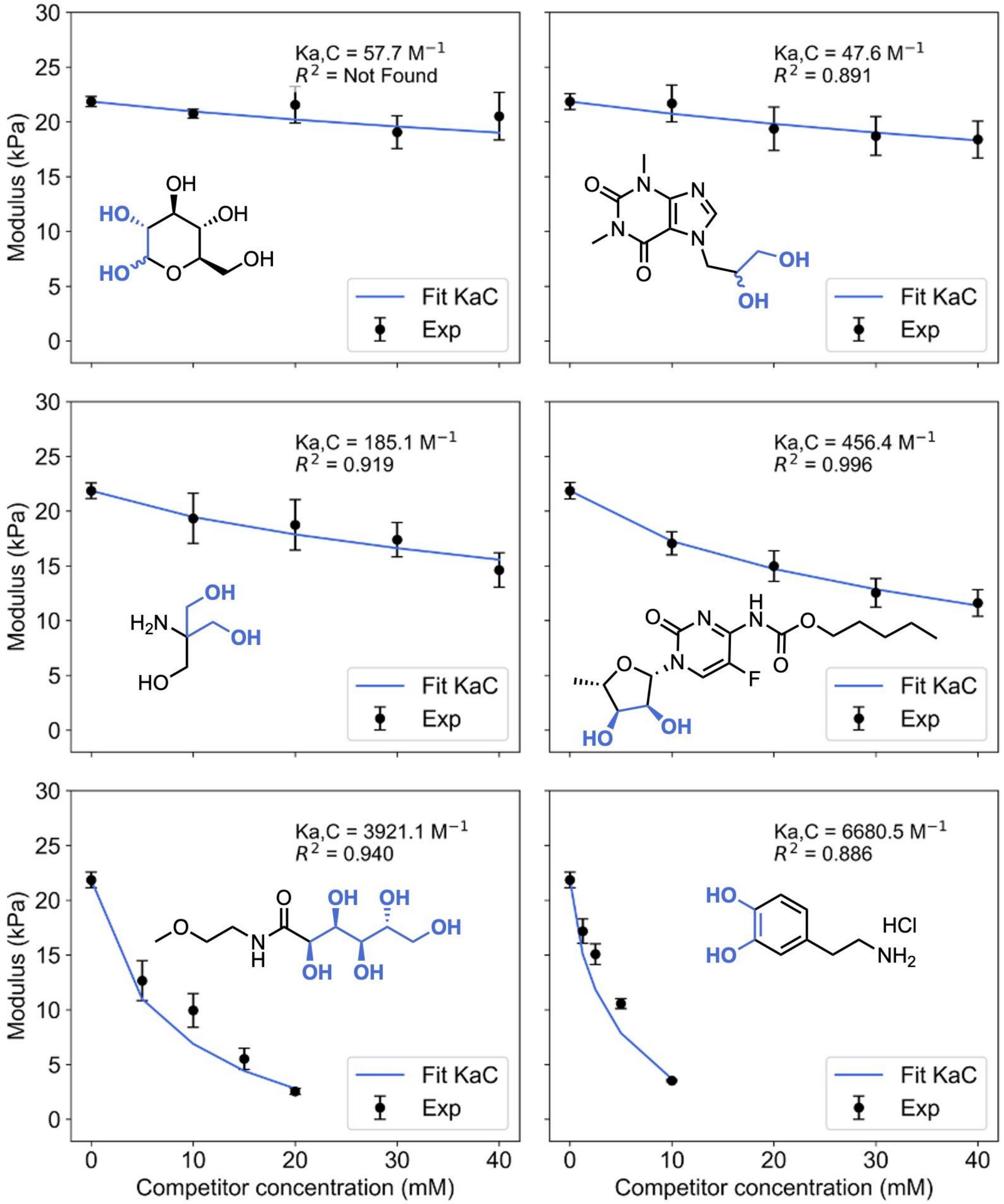

Figure S49: Fitting the modulus vs competitor concentration of all 6 competitors using equations S10, S14, and S20 to fit for the $K_{a,C}$ of the competitor.

Representative Stress Relaxation Fits

To find the τ value of our network under various competitor concentrations we compared 3 methods.

1. Crossover Frequency method[4]

τ can be obtained directly through the crossover frequency (ω), through the relationship:

$$\tau = 1/\,\omega.$$

This method was used to extract tau values for the boronate ester crosslinked networks.

2. Single Mode Maxwell fit[21]

The normalized relaxation modulus can be fit to a single order Maxwellian fit:

$$G(t)/G_0 = \exp\left(-\frac{t}{\tau}\right).$$

3. Kohlrausch-Williams-Watts(KWW) fit[22,23]

Finally, the relaxation can be described by the stretched exponential KWW fit:

$$G(t)/G_0 = \exp\left(-\left(\frac{t}{\tau_{kww}}\right)^{\beta}\right).$$

Where β is the stretching exponential. The average τ *value,* $<\tau>$, can be obtained from τ_{kww} and β through:

$$<\tau>= \frac{\tau_{kww}}{\beta}\Gamma\left(\frac{1}{\beta}\right).$$

This method was use for extracting the tau values for the hydrazone crosslinked networks.

Table S16: Summary of dopamine rheology results.

Competitor	Conc (mM)	τ cross freq. (s)	τ maxwell (s)	τ_{kww} (s)	β fit	$<\tau>$ (s)
---	0	0.727	0.731	0.769	1.00	0.769
Glucose (1)	10	0.726	0.737	0.737	1.00	0.737
	20	0.694	0.648	0.648	1.00	0.648
	30	0.645	0.548	0.548	1.00	0.548
	40	0.696	0.692	0.692	1.00	0.692
Dyphylline (2)	10	0.758	0.770	0.770	1.00	0.770
	20	0.726	0.777	0.777	1.00	0.777
	30	0.734	0.702	0.702	1.00	0.702
	40	0.610	0.619	0.619	1.00	0.619
Tris (3)	10	1.170	1.136	1.14	0.995	1.14
	20	1.592	1.655	1.66	0.999	1.66
	30	1.989	1.926	1.94	0.977	1.96
	40	2.180	1.989	2.04	0.920	2.12
Capecitabine (4)	10	0.614	0.645	0.645	1.00	0.645
	20	0.552	0.536	0.536	1.00	0.536
	30	0.509	0.508	0.508	1.00	0.508
	40	0.457	0.449	0.449	1.00	0.449
Cl-ana (5)	10	0.505	0.552	0.552	1.00	0.552
	20	0.360	0.411	0.411	1.00	0.411
	30	0.310	0.334	0.334	1.00	0.334
	40	0.194	0.213	0.213	1.00	0.213
Dopamine (6)	10	0.694	0.668	0.668	0.998	0.668
	20	0.629	0.633	0.633	1.00	0.633
	30	0.463	0.471	0.471	1.00	0.471
	40	0.241	0.272	0.272	1.00	0.272

Tau Predictions

To demonstrate how our equation for fitting tau is a function of $K_{a,app}$ we added the following expansion of the expression:

$$\tau([C]) = \tau_0 - (\tau_0 - \tau_{min}) \cdot \left(\frac{2v_e}{N_{XL}}\right)$$

Plug in eq S19

$$\tau([C]) = \tau_0 - (\tau_0 - \tau_{min}) \cdot \frac{1}{2}\left(\frac{3}{2}4P_{out}(1-P_{out})^3 + 2(1-P_{out})^4\right)$$

Plug in eq S16, S17

$$\tau([C]) = \tau_0 - (\tau_0 - \tau_{min}) \cdot \left(\frac{1}{2}\left(6\left[\left(\frac{1}{p}-\frac{3}{4}\right)^{\frac{1}{2}}-\frac{1}{2}\right]\left(1-\left[\left(\frac{1}{p}-\frac{3}{4}\right)^{\frac{1}{2}}-\frac{1}{2}\right]\right)^3 + 2\left(1-\left[\left(\frac{1}{p}-\frac{3}{4}\right)^{\frac{1}{2}}-\frac{1}{2}\right]\right)^4\right)\right)$$

Plug in eq S14

$$\tau([C]) = \tau_0 - (\tau_0 - \tau_{min}) \cdot \frac{1}{2}\left(6\left[\left(\frac{1}{\left(1+\frac{1}{2N_{XL}K_{a,app}}\right)-\left[\left(1+\frac{1}{2N_{XL}K_{a,app}}\right)^2-1\right]^{1/2}}-\frac{3}{4}\right)^{\frac{1}{2}}-\frac{1}{2}\right]\left(1-\left[\left(\frac{1}{\left(1+\frac{1}{2N_{XL}K_{a,app}}\right)-\left[\left(1+\frac{1}{2N_{XL}K_{a,app}}\right)^2-1\right]^{1/2}}-\frac{3}{4}\right)^{\frac{1}{2}}-\frac{1}{2}\right]\right)^3 + 2\left(1-\left[\left(\frac{1}{\left(1+\frac{1}{2N_{XL}K_{a,app}}\right)-\left[\left(1+\frac{1}{2N_{XL}K_{a,app}}\right)^2-1\right]^{1/2}}-\frac{3}{4}\right)^{\frac{1}{2}}-\frac{1}{2}\right]\right)^4\right)$$

To further demonstrate the dependence of tau on competitor binding affinity, we applied linear fits of $\tau([C])$ as a function of [C], and we found that the more strongly associating competitors had steeper linear slopes.

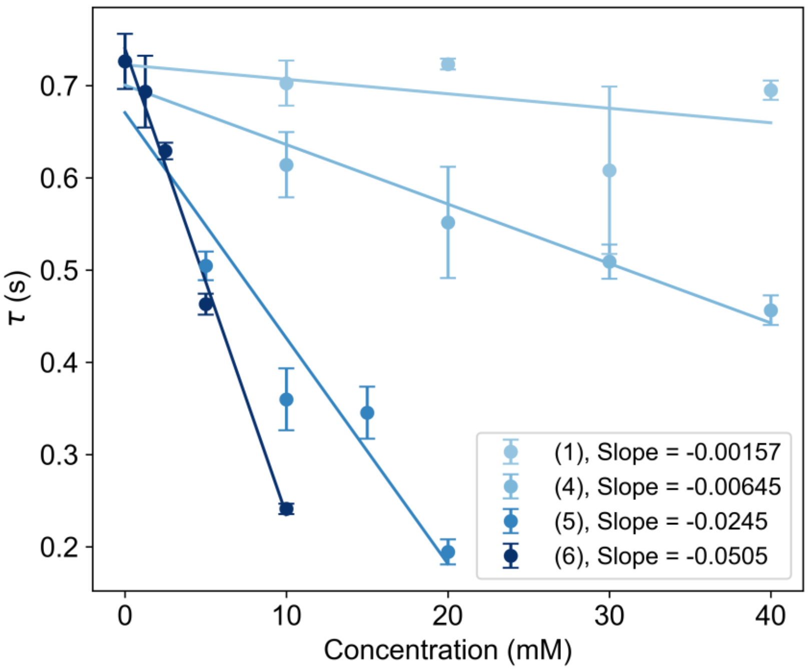

Figure S50: τ vs competitor concentrations of (1), (4), (5), and (6) with a linear fit. As binding affinity for competitor increases from 6.2 M^{-1} (competitor (1) to 6700 M^{-1}(competitor (6)), the magnitude of the slope increases from -0.00157, to -0.0505.

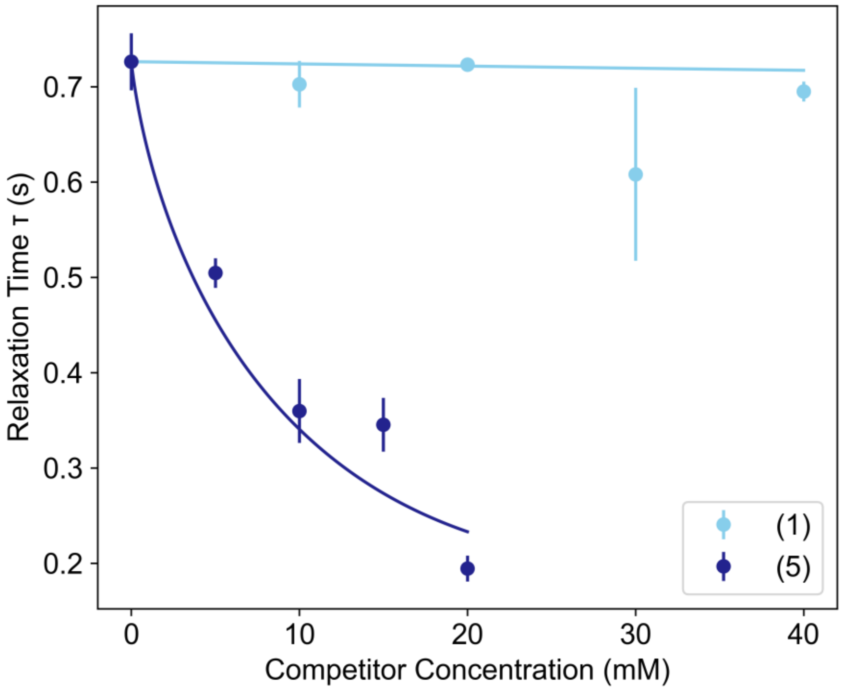

Figure S51: τ vs competitor concentration of , (1) and (5) fitted with Langmuir decay model (equation 6). Where for (1) fitted τ_{min} = 0.001 s, R^2 = not found and (5) fitted τ_{min} = 0.121 s, R^2 = 0.9413, with τ values extracted from crossover frequency.

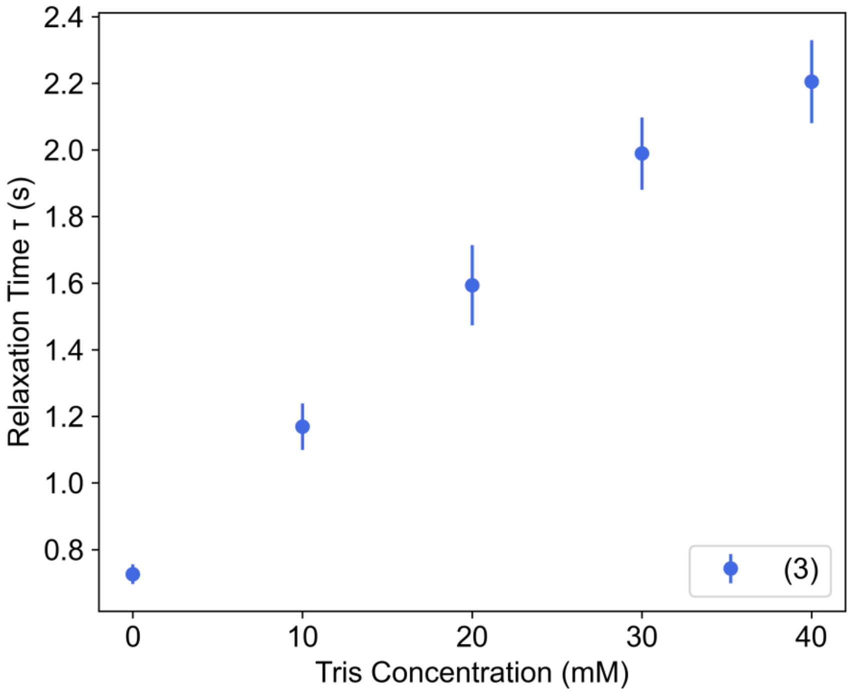

Figure S52: τ vs concentration of (3) with no fit because the τ are increasing, with τ values extracted from crossover frequency.

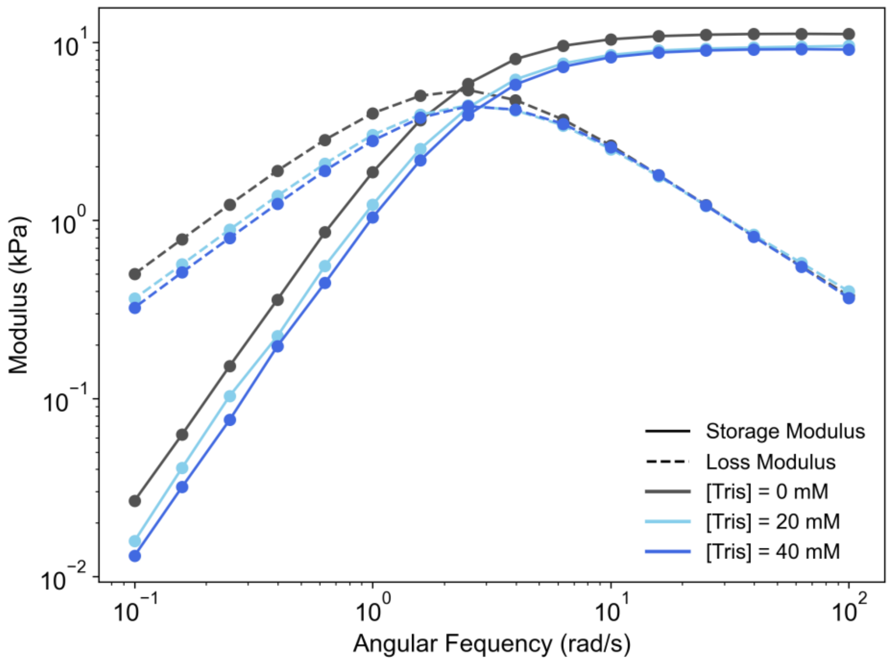

Figure S53: Frequency-dependent storage (solid line) and loss modulus (dashed line) from 0.1 rad/s – 100 rad/s, 1% strain of boronate ester hydrogels with [ion] adjusted to 80 mM with NaCl and pH adjusted to 7.4 with HCl in 0.1 M HEPES. Concentrations range from 0 – 20 mM Tris (4) conducted at 25 °C.

UV-Vis Titration for MeHz K_a determination

For the association constant (K_a) determination, stock solutions of 700 µM 2kDa mPEG-Ar-CHO in PBS 1X and 180 mM MeHz as described above (in 0.195 M NaCl) were made. The desired ratios from 1:1 to 75:1 ([MeHz]:[2kDa mPEG-Ar-CHO]) started from 200 µM with a 3.5 mL total volume per sample. For the hydrazone crosslink, 2kDa mPEG-Hz solution (in 0.195 M NaCl) and 2kDa mPEG-Ar-CHO were titrated in the ratio of 1:1 to 30:1 starting from 50 µM. Each titration was left to equilibrate overnight before the samples were subjected to the UV-Vis experiment. The association constant (K_a) was determined by multiwavelength, non-linear curve fitting using BindFit.[24,25] The spectral region λ = 298 nm – 305 nm was used because this is where the sample absorbs the most. The best fit was obtained using a 1:1 host–guest stoichiometric model.

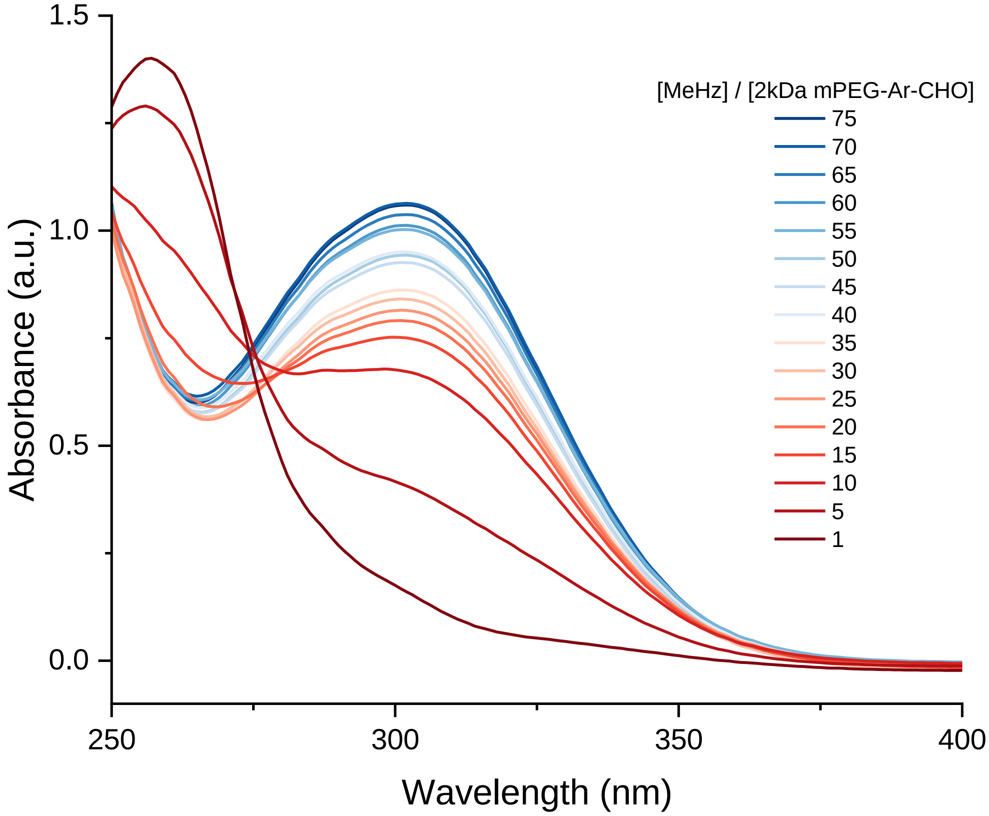

Figure S54. UV-Vis traces stacked in an overlay of all ratios of MeHz to 2kDa mPEG-Ar-CHO in PBS. Replicate 1.

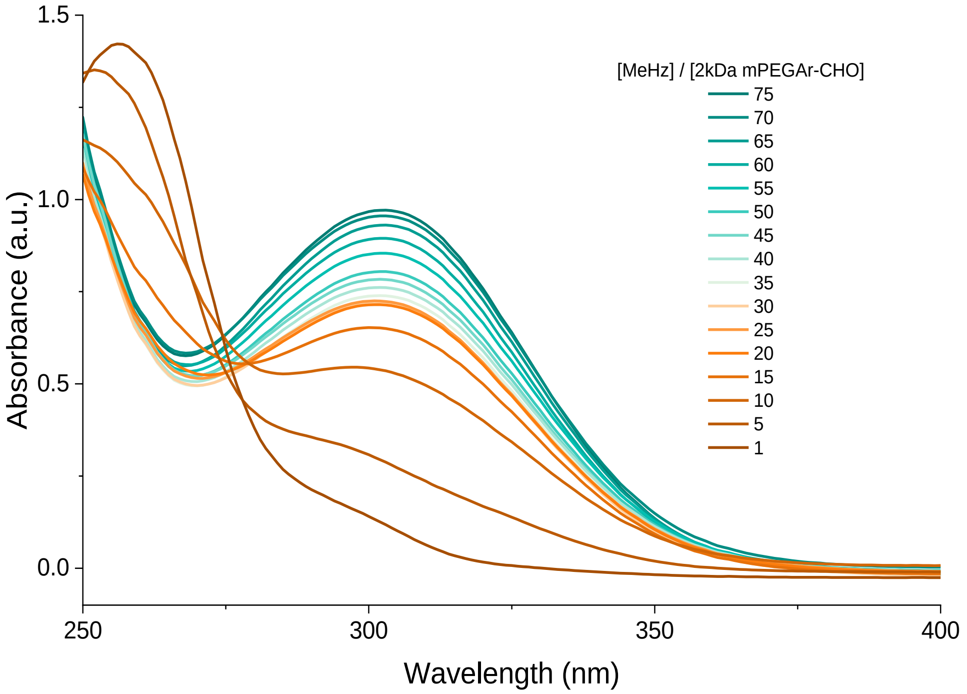

Figure S55. UV-Vis traces stacked in an overlay of all ratios of MeHz to 2kDa mPEG-Ar-CHO in PBS. Replicate 2.

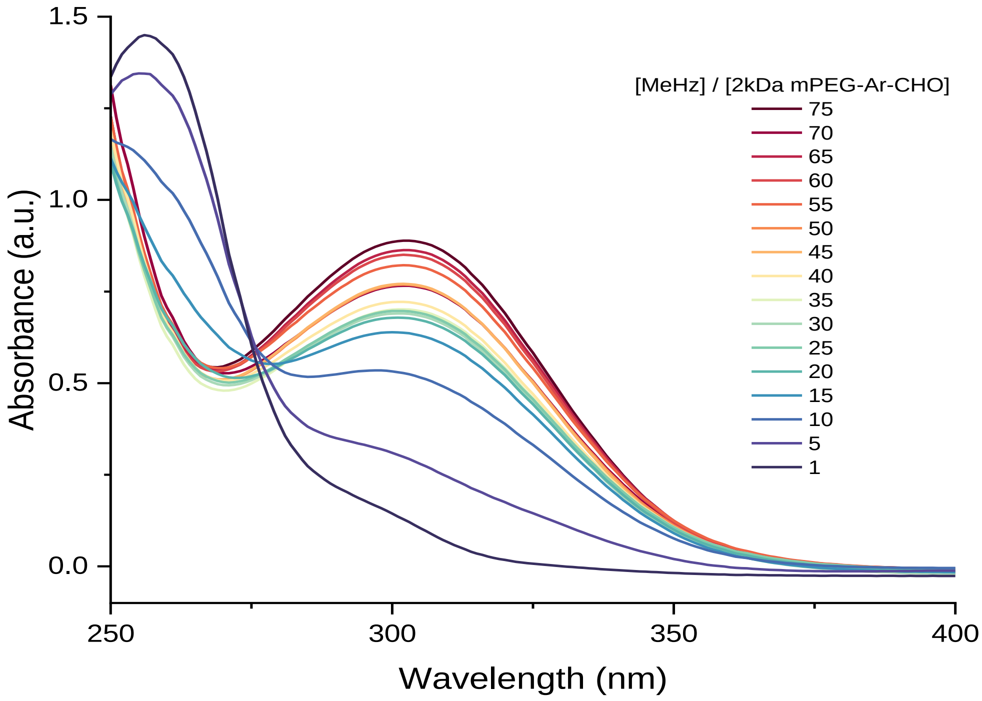

Figure S56. UV-Vis traces stacked in an overlay of all ratios of MeHz to 2kDa mPEG-Ar-CHO in PBS. Replicate 3.

Table S17. Raw λ_{max} absorbance data for each titration.

Ratio ([MeHz]:[2kDa mPEG-Ar-CHO])	Absorbance at λ_{max} (302 nm)		
	Replicate 1	Replicate 2	Replicate 3
1:1	0.160	0.125	0.128
5:1	0.406	0.404	0.299
10:1	0.672	0.538	0.527
15:1	0.750	0.651	0.637
20:1	0.790	0.715	0.678
25:1	0.814	0.724	0.697
30:1	0.840	0.722	0.690
35:1	0.862	0.739	0.701
40:1	0.950	0.761	0.722
45:1	0.926	0.783	0.771
50:1	0.943	0.804	0.769
55:1	1.003	0.854	0.822
60:1	1.012	0.894	0.850
65:1	1.037	0.930	0.863
70:1	1.063	0.955	0.766
75:1	1.060	0.971	0.889

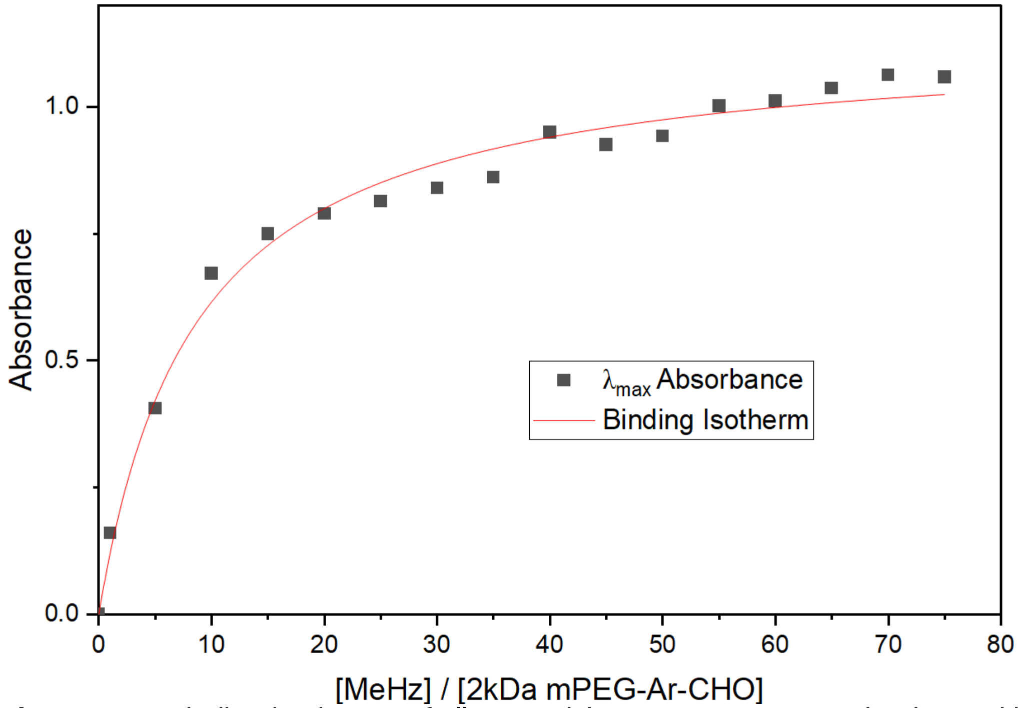

Figure S57. Binding isotherms of all MeHz /2kDa mPEG-Ar-CHO titrations with absorbances obtained from UV-Vis traces. Replicate 1.

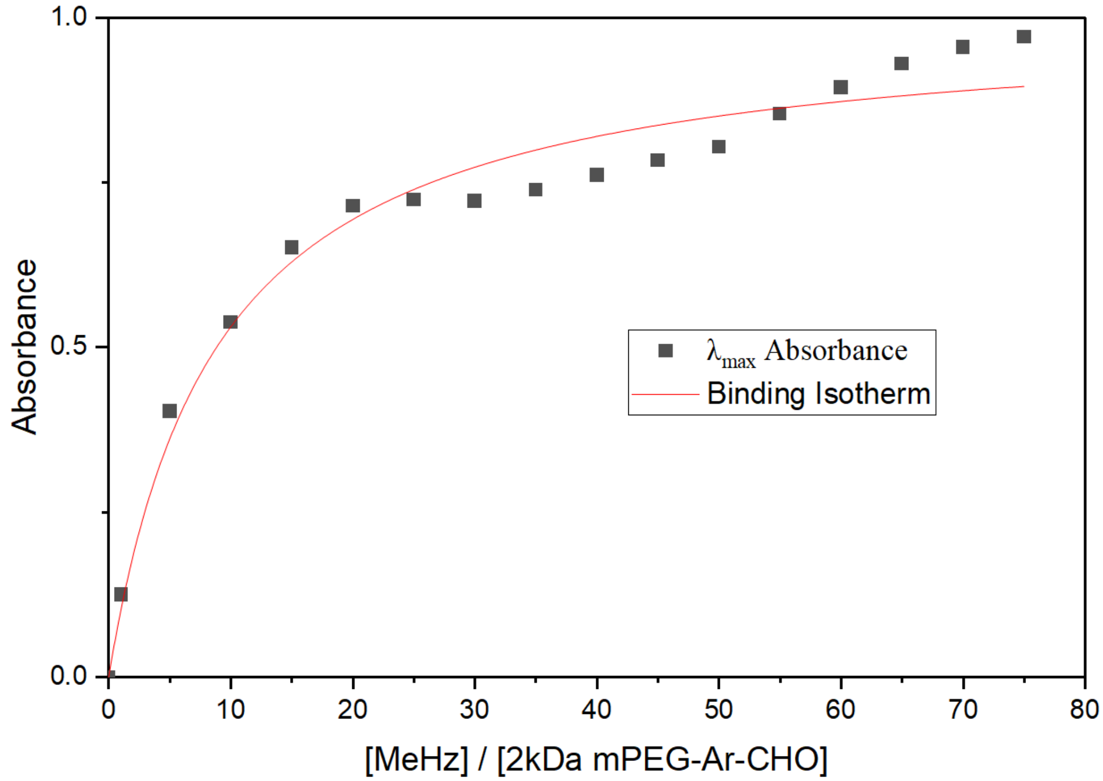

Figure S58. Binding isotherms of all MeHz/2kDa mPEG-Ar-CHO titrations with absorbances obtained from UV-Vis traces. Replicate 2.

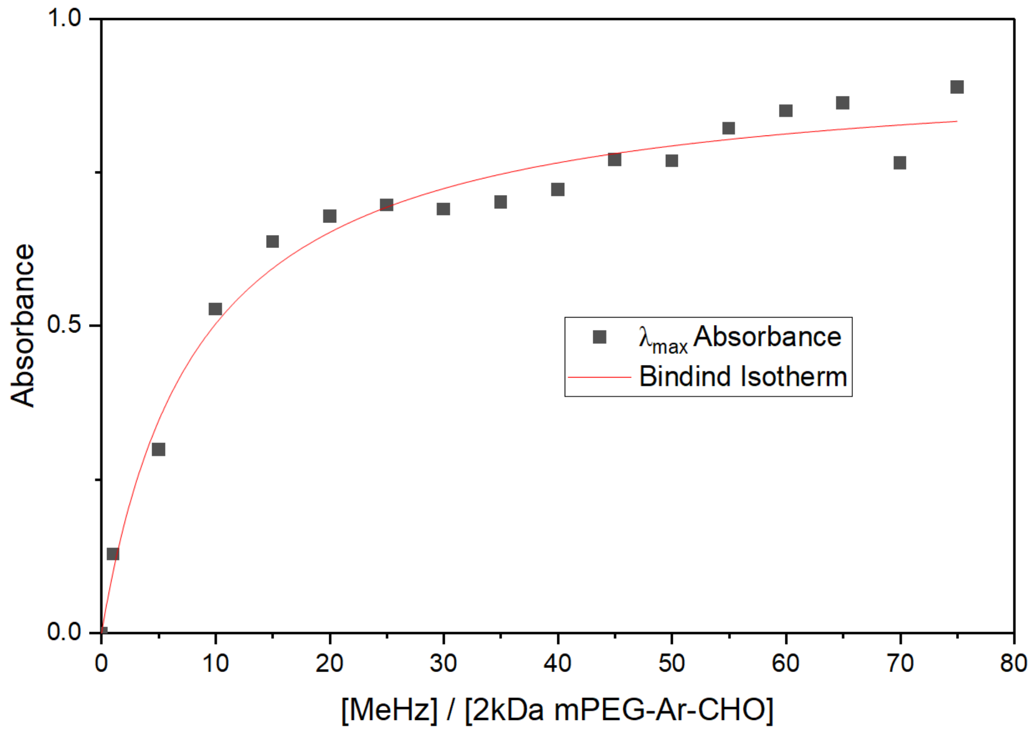

Figure S59. Binding isotherms of all MeHz/2kDa mPEG-Ar-CHO titrations with absorbances obtained from UV-Vis traces. Replicate 3.

Table S18. Summary of K_a and fitting error of MeHz/2kDa mPEG-Ar-CHO from the BindFit using

Replicate 1		Replicate 2		Replicate 3	
K_a (M^{-1})	Fitting error (%)	K_a (M^{-1})	Fitting error (%)	K_a (M^{-1})	Fitting error (%)
505.82	± 3.42	453.34	± 4.91	576.51	± 4.87

Average K_a = 512 ± 61.8 M^{-1} with average fitting error ± 4.40 %

λ = 298-305 nm.

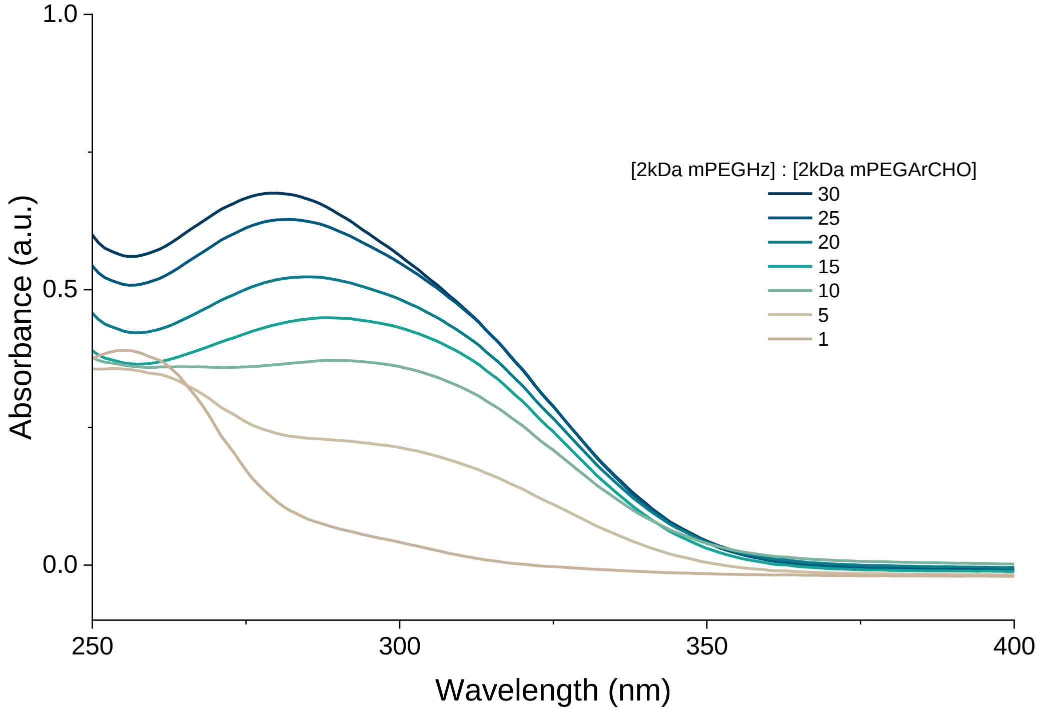

Figure S60. UV-Vis traces stacked in an overlay of all ratios of 2kDa mPEG-Hz to 2kDa mPEG-Ar-CHO in PBS. Replicate 1.

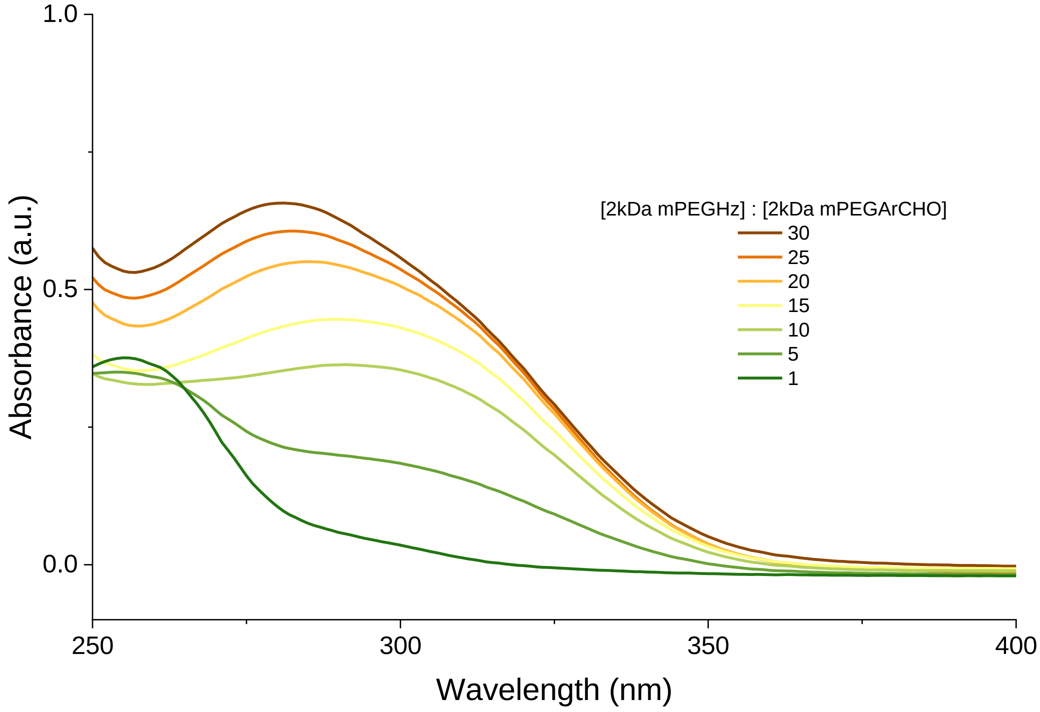

Figure S61. UV-Vis traces stacked in an overlay of all ratios of 2kDa mPEG-Hz to 2kDa mPEG-Ar-CHO in PBS. Replicate 2.

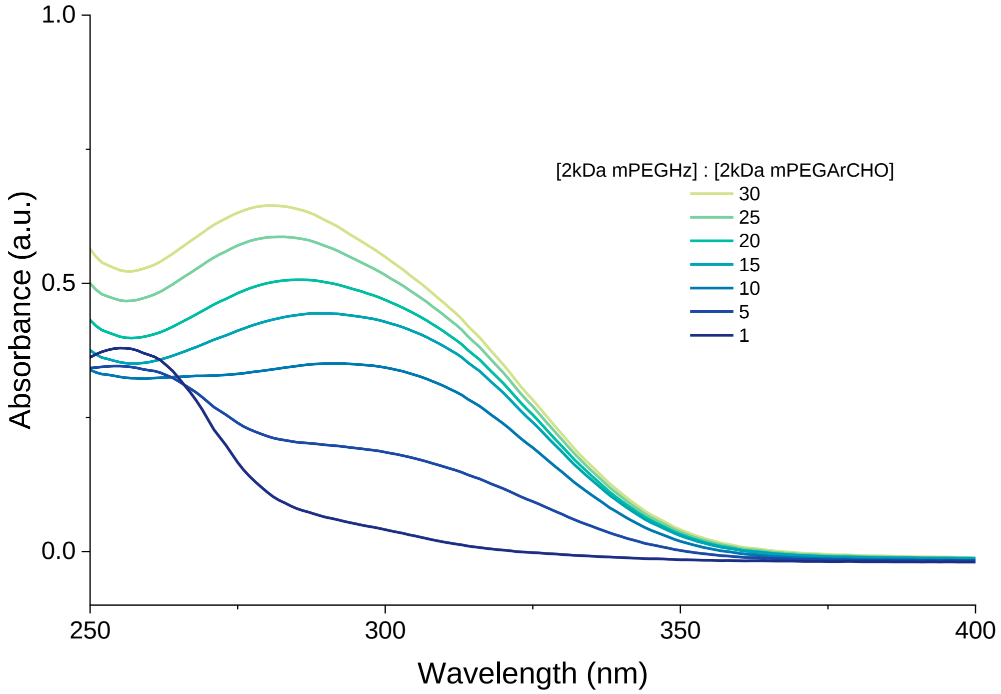

Figure S62. UV-Vis traces stacked in an overlay of all ratios of 2kDa mPEG-Hz to 2kDa mPEG-Ar-CHO in PBS. Replicate 3.

Table S19. Raw λ_{max} absorbance data for each titration.

Ratio ([2kDa mPEG-Hz]:[2kDa mPEG-Ar-CHO])	Absorbance at λ_{max} (302 nm)		
	Replicate 1	Replicate 2	Replicate 3
1:1	0.036	0.030	0.036
5:1	0.209	0.179	0.181
10:1	0.355	0.349	0.338
15:1	0.424	0.424	0.422
20:1	0.473	0.495	0.459
25:1	0.534	0.523	0.502
30:1	0.545	0.541	0.533

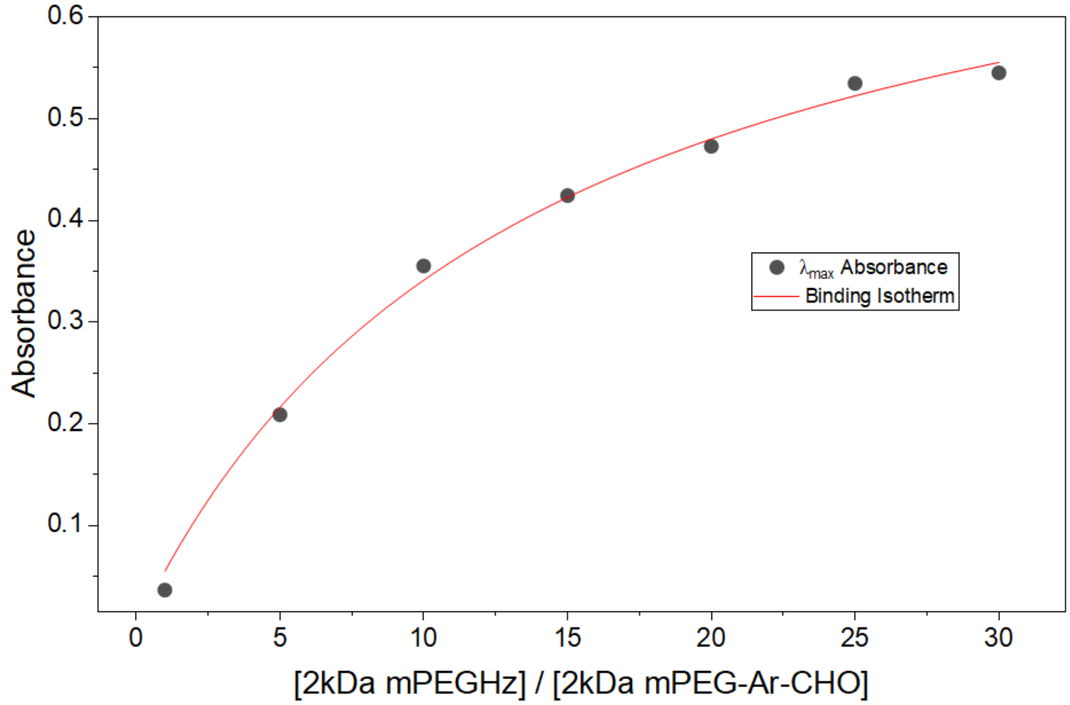

Figure S63. Binding isotherms of all 2kDa mPEG-Hz/2kDa mPEG-Ar-CHO titrations obtained from UV-Vis traces. Replicate 1.

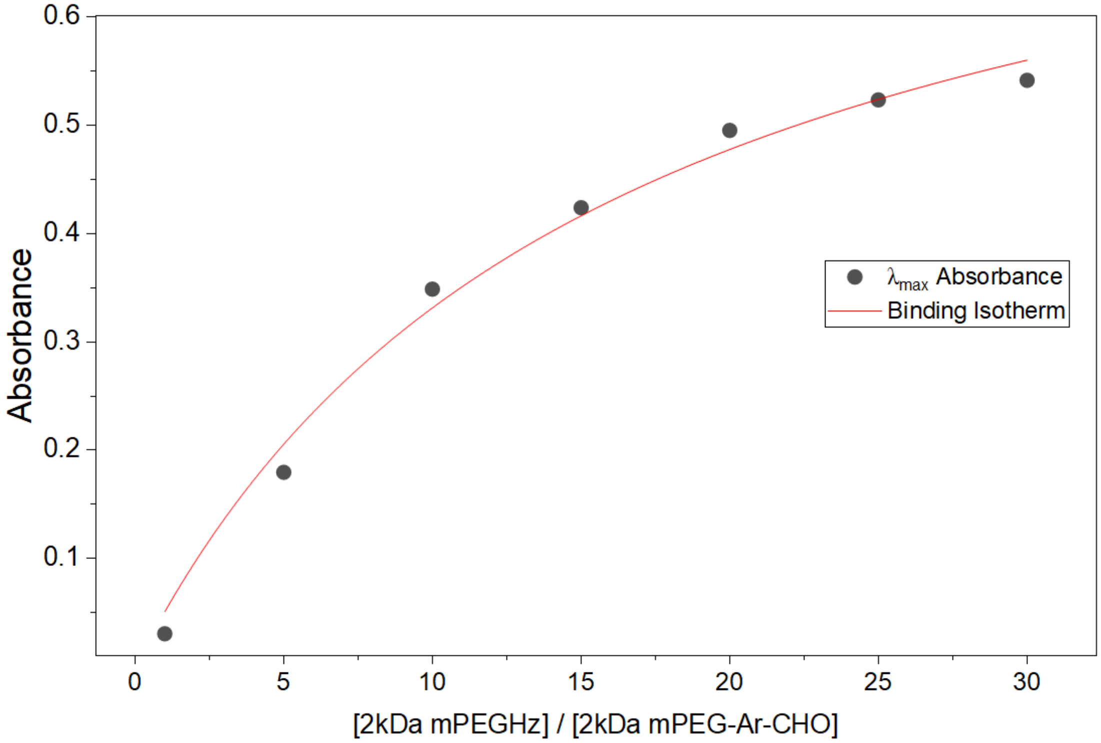

Figure S64. Binding isotherms of all 2kDa mPEG-Hz/2kDa mPEG-Ar-CHO titrations with absorbances obtained from UV-Vis traces. Replicate 2.

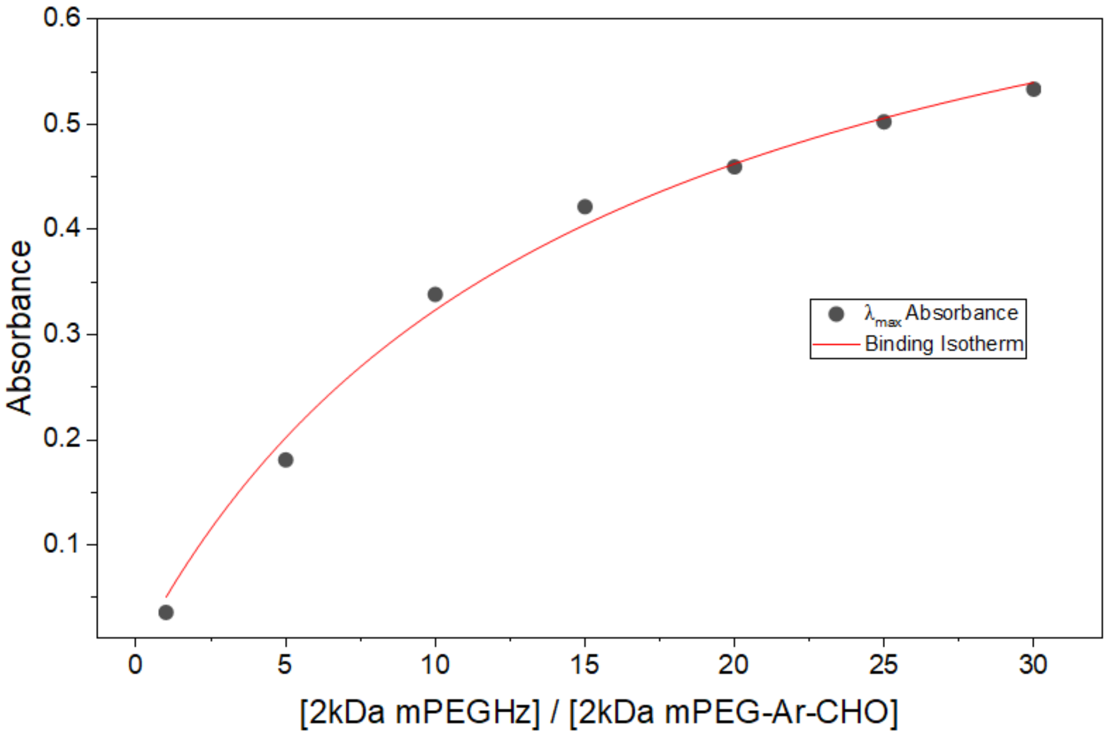

Figure S65. Binding isotherms of all 2kDa mPEG-Hz/2kDa mPEG-Ar-CHO titrations with absorbances obtained from UV-Vis traces. Replicate 3.

Table S20. Summary of K_a and fitting error of 2kDa mPEG-Hz/2kDa mPEG-Ar-CHO from the BindFit using λ = 298-305 nm.

Replicate 1		Replicate 2		Replicate 3	
K_a (M^{-1})	Fitting error (%)	K_a (M^{-1})	Fitting error (%)	K_a (M^{-1})	Fitting error (%)
1474	± 2.22	1235	± 3.11	1328	± 2.52

Average K_a = 1346 ± 120 M^{-1} with average fitting error ± 2.62 %

Rheology values for Hydrazone Gels

Table S21. Values of the rheology represented in figure 5.

[MeHz] mM	Replicate	Modulus (kPa)	τ maxwell (s)	τ fit KWW (s)	β fit	<τ> (s)
0	1	17.5	526	539	0.924	559
	2	16.9	687	729	0.843	797
	3	14.3	664	685	0.912	716
	Avg	16.2	626	651	0.893	691
	Stdev	1.71	87	99	0.044	121
27	1	5.63	390	397	0.941	408
	2	5.47	457	476	0.876	509
	3	5.80	552	561	0.951	574
	Avg	5.63	466	478	0.923	497
	Stdev	0.161	81	82	0.041	83
63	1	2.64	368	379	0.912	396
	2	2.86	521	538	0.912	562
	3	2.25	474	482	0.950	493
	Avg	2.58	454	466	0.925	484
	Stdev	0.309	78	81	0.022	83
81	1	1.81	275	255	0.735	309
	2	2.80	222	171	0.570	276
	3	1.54	255	227	0.669	301
	Avg	2.05	251	218	0.658	295
	Stdev	0.661	27	43	0.083	17

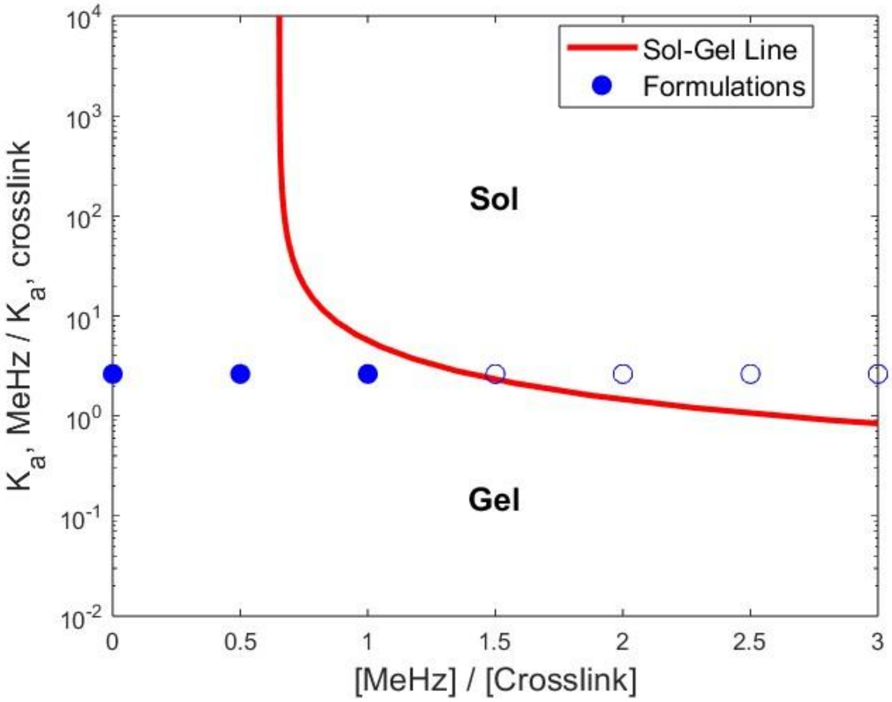

Figure S66: Plot of K_a MeHz / $K_{a,XL}$ of the 4PEG-hydrazone system vs. [MeHz] / [Crosslink]. Solid dots represent formulations that made gels, where open dots represent formulations that did not gel. Phase diagram was calculated based on equations from Heilshorn *et. al*.[26]

Swelling Test
The benzyl-hydrazone PEG-based hydrogel 10% w/v (total volume 200 µL) was submerged in PBS 1X 20 mL for 2 days. The swelling ratio is calculated by the equation below:

Swelling ratio = $\frac{W_s - W_d}{W_d}$

where W_s and W_d are the hydrogel weights after swelling and in the dry state, respectively.

The volume fraction ($\boldsymbol{\phi}_s$) of the hydrogel is calculated via the following equation:

$$\boldsymbol{\phi}_s = [1 + \frac{\rho_p}{\rho_w}\left(\frac{W_s}{W_d} - 1\right)]^{-1}$$

where ρ_p and ρ_w are assumed as the densities of PEG (1.125 g/mL) and water (1.000 g/mL), and W_s and W_d are the hydrogel weights after swelling and in the dry state, respectively.[27]

After 2 days, the PBS was replaced with fresh PBS buffer for another 1 day. Aliquots of PBS after 2 days and fresh PBS after another 1 day were taken for UV-Vis spectroscopy.

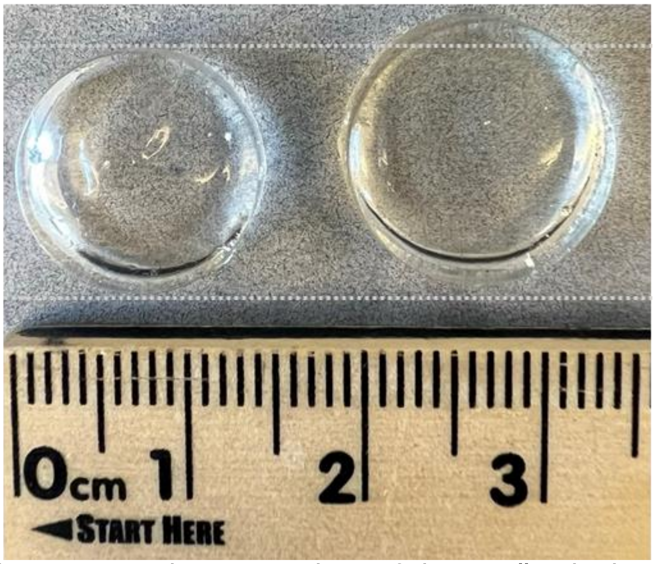

Figure S67: Size comparison of the swollen hydrogel without competitor (left) and with 63 mM MeHz (right).

Table S22. The swelling ratio and volume fraction of the hydrogel with and without MeHz competitor.

w/o or w/ competitor	Swelling ratio	Volume fraction; $\boldsymbol{\phi}_s$
w/o competitor	5.48 ±0.85	0.162 ± 0.034
with 63 mM MeHz	8.15±1.06	0.112 ± 0.014

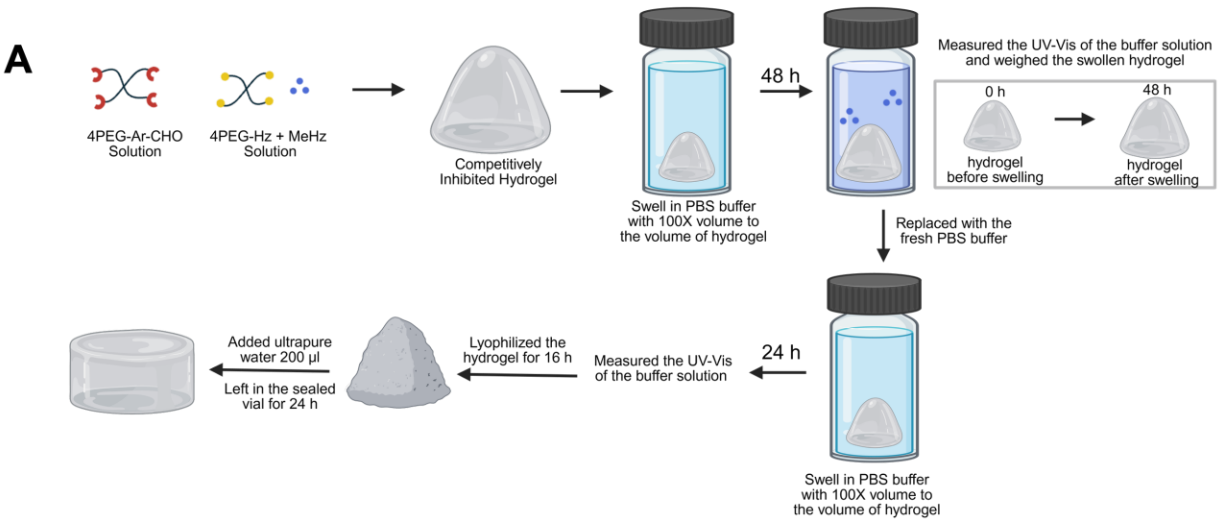

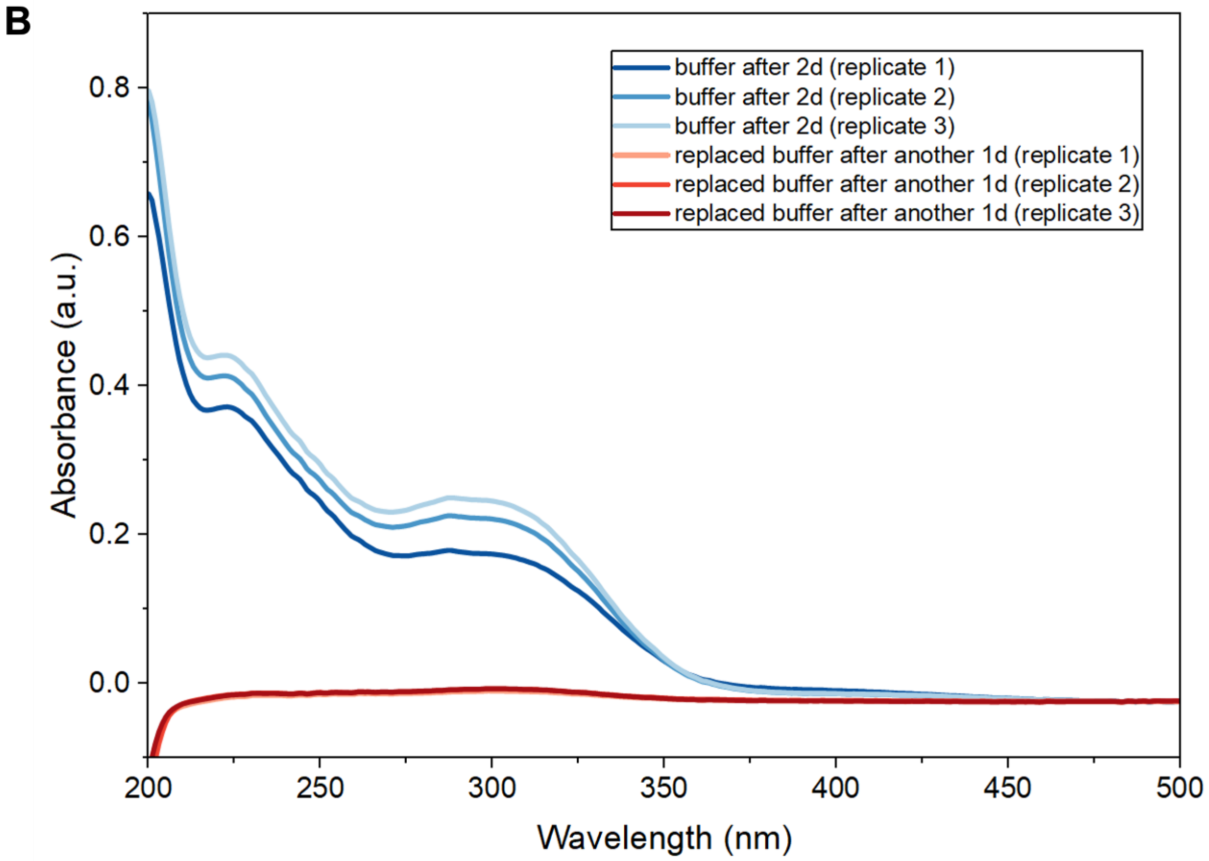

Figure S68: (A) Schematic detailing the swelling, washing, and rehydration of process of hydrazone-crosslinked gels. **(B)** The UV-Vis absorbance of PBS buffer after washing and after replacing with fresh buffer.

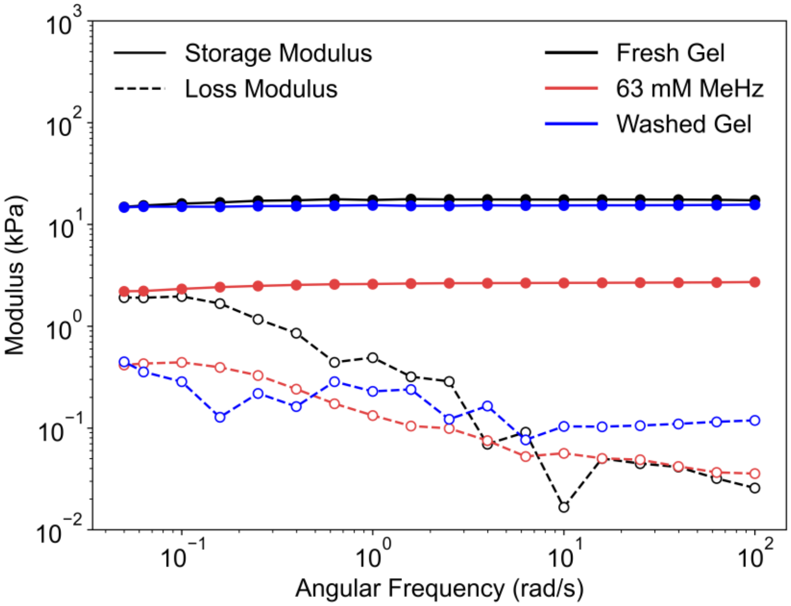

Figure S69: Frequency-dependent storage (solid markers) and loss modulus (open markers) from 0.05 rad/s – 100 rad/s, 1% strain of hydrazone-crosslinked gels after initial preparation with no competitor (black), with 63 mM of competitor (red), and after removing competitor (blue). Conducted at 25 °C.

Self-healing Test

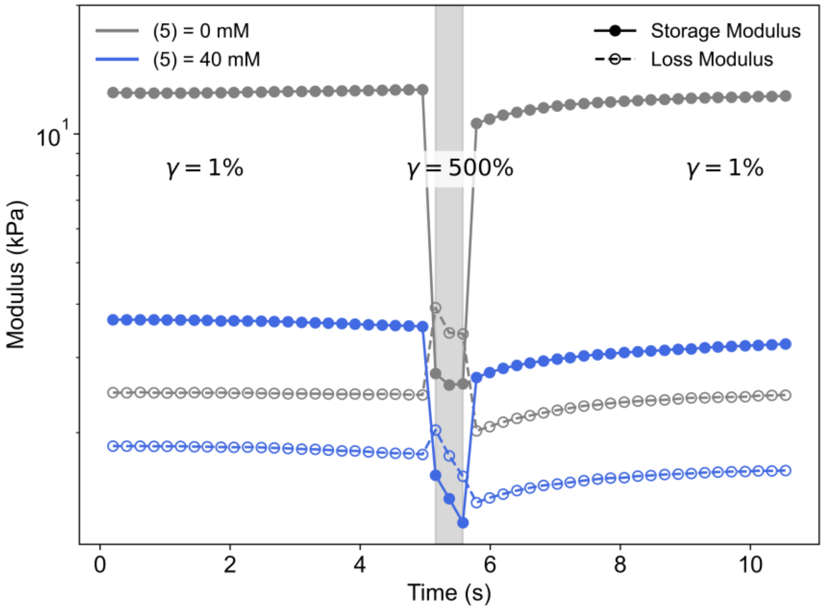

Figure S70: Step-strain measurement of boronate ester gels with and without competitor showing similar recovery of moduls at 10 rad/s going from low strain (1%) to high strain (500%) back to 1% strain. Open dots represent loss modulus and closed dots represent storage modulus.

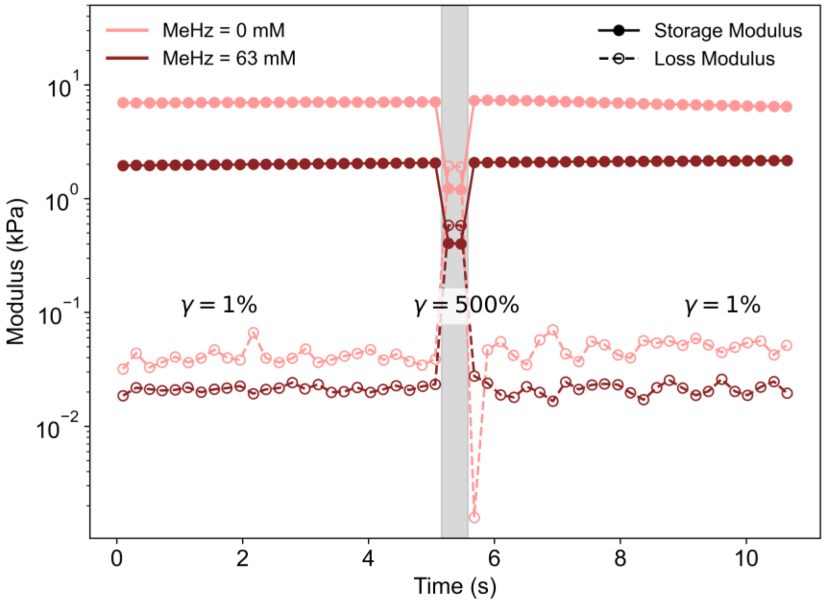

Figure S71: Step strain measurement of hydrazone gels with and without competitor showing similar recovery of modulus at 10 rad/s going from low strain (1%) strain to high strain (500% strain) back to 1% strain.

References

(1) Palmer, T.; Bonner, P. L. Enzyme Inhibition. In *Enzymes*; Elsevier, 2011; pp 126–152. https://doi.org/10.1533/9780857099921.2.126.

(2) Cai, P. C.; Krajina, B. A.; Spakowitz, A. J. Brachiation of a Polymer Chain in the Presence of a Dynamic Network. *Phys. Rev. E* **2020**, *102* (2), 020501. https://doi.org/10.1103/PhysRevE.102.020501.

(3) Cai, P. C.; Su, B.; Zou, L.; Webber, M. J.; Heilshorn, S. C.; Spakowitz, A. J. Rheological Characterization and Theoretical Modeling Establish Molecular Design Rules for Tailored Dynamically Associating Polymers. *ACS Cent. Sci.* **2022**, *8* (9), 1318–1327. https://doi.org/10.1021/acscentsci.2c00432.

(4) Parada, G. A.; Zhao, X. Ideal Reversible Polymer Networks. *Soft Matter* **2018**, *14* (25), 5186–5196. https://doi.org/10.1039/C8SM00646F.

(5) Marco-Dufort, B.; Iten, R.; Tibbitt, M. W. Linking Molecular Behavior to Macroscopic Properties in Ideal Dynamic Covalent Networks. *J. Am. Chem. Soc.* **2020**, *142* (36), 15371–15385. https://doi.org/10.1021/JACS.0C06192.

(6) Marco-Dufort, B.; Iten, R.; Tibbitt, M. W. Linking Molecular Behavior to Macroscopic Properties in Ideal Dynamic Covalent Networks. *J. Am. Chem. Soc.* **2020**, *142* (36), 15371–15385. https://doi.org/10.1021/jacs.0c06192.

(7) Jolly, R. D. Mannosidosis of Angus Cattle: A Prototype Control Program for Some Genetic Diseases. *Adv. Vet. Sci. Comp. Med.* **1975**, *19*, 1–21.

(8) Miller, D. R.; Macosko, C. W. A New Derivation of Post Gel Properties of Network Polymers. *Macromolecules* **1976**, *9* (2), 206–211. https://doi.org/10.1021/ma60050a004.

(9) Claiborne, Alexander D.; Hill, M. R. InhibNet, 2025. https://github.com/hill-lab-chem/InhibNet.

(10) Hydrogel Design. https://hydrogeldesign.org/the-model/rubberlike-elasticity-theory/.

(11) Flory, P. J. Theory of Elasticity of Polymer Networks. The Effect of Local Constraints on Junctions. *J. Chem. Phys.* **1977**, *66* (12), 5720–5729. https://doi.org/10.1063/1.433846.

(12) Akagi, Y.; Gong, J. P.; Chung, U.; Sakai, T. Transition between Phantom and Affine Network Model Observed in Polymer Gels with Controlled Network Structure. *Macromolecules* **2013**, *46* (3), 1035–1040. https://doi.org/10.1021/ma302270a.

(13) Cho, S.; Hwang, S. Y.; Oh, D. X.; Park, J. Recent Progress in Self-Healing Polymers and Hydrogels Based on Reversible Dynamic B–O Bonds: Boronic/Boronate Esters, Borax, and Benzoxaborole. *J. Mater. Chem. A* **2021**, *9* (26), 14630–14655. https://doi.org/10.1039/D1TA02308J.

(14) Kang, B.; Kalow, J. A. Internal and External Catalysis in Boronic Ester Networks. *ACS Macro Lett.* **2022**, *11* (3), 394–401. https://doi.org/10.1021/acsmacrolett.2c00056.

(15) Xiang, Y.; Xian, S.; Ollier, R. C.; Yu, S.; Su, B.; Pramudya, I.; Webber, M. J. Diboronate Crosslinking: Introducing Glucose Specificity in Glucose-Responsive Dynamic-Covalent Networks. *J. Controlled Release* **2022**, *348*, 601–611. https://doi.org/10.1016/j.jconrel.2022.06.016.

(16) Richardson, B. M.; Walker, C. J.; Macdougall, L. J.; Hoye, J. W.; Randolph, M. A.; Bryant, S. J.; Anseth, K. S. Viscoelasticity of Hydrazone Crosslinked Poly(Ethylene Glycol) Hydrogels Directs Chondrocyte Morphology during Mechanical Deformation. *Biomater. Sci.* **2020**, *8* (14), 3804–3811. https://doi.org/10.1039/D0BM00860E.

(17) McKinnon, D. D.; Domaille, D. W.; Cha, J. N.; Anseth, K. S. Biophysically Defined and Cytocompatible Covalently Adaptable Networks as Viscoelastic 3D Cell Culture Systems. *Adv. Mater.* **2014**, *26* (6), 865–872. https://doi.org/10.1002/adma.201303680.

(18) Burnouf, D.; Ennifar, E.; Guedich, S.; Puffer, B.; Hoffmann, G.; Bec, G.; Disdier, F.; Baltzinger, M.; Dumas, P. kinITC: A New Method for Obtaining Joint Thermodynamic and Kinetic Data by Isothermal Titration Calorimetry. *J. Am. Chem. Soc.* **2012**, *134* (1), 559–565. https://doi.org/10.1021/ja209057d.

(19) Dumas, P.; Ennifar, E.; Da Veiga, C.; Bec, G.; Palau, W.; Di Primo, C.; Piñeiro, A.; Sabin, J.; Muñoz, E.; Rial, J. Extending ITC to Kinetics with kinITC. In *Methods in Enzymology*; Elsevier, 2016; Vol. 567, pp 157–180. https://doi.org/10.1016/bs.mie.2015.08.026.
(20) Springsteen, G.; Wang, B. A Detailed Examination of Boronic Acid–Diol Complexation. *Tetrahedron* **2002**, *58* (26), 5291–5300. https://doi.org/10.1016/S0040-4020(02)00489-1.
(21) Oglesby, P. L. *Mechanical Properties - Viscoelastic Methods : Progress Report*, 0 ed.; NBS RPT 10294; National Bureau of Standards: Gaithersburg, MD, 1970; p NBS RPT 10294. https://doi.org/10.6028/NBS.RPT.10294.
(22) Chen, Q.; Tudryn, G. J.; Colby, R. H. Ionomer Dynamics and the Sticky Rouse Model. *J. Rheol.* **2013**, *57* (5), 1441–1462. https://doi.org/10.1122/1.4818868.
(23) Tang, S.; Wang, M.; Olsen, B. D. Anomalous Self-Diffusion and Sticky Rouse Dynamics in Associative Protein Hydrogels. *J. Am. Chem. Soc.* **2015**, *137* (11), 3946–3957. https://doi.org/10.1021/jacs.5b00722.
(24) Bindfit. http://supramolecular.org.
(25) Brynn Hibbert, D.; Thordarson, P. The Death of the Job Plot, Transparency, Open Science and Online Tools, Uncertainty Estimation Methods and Other Developments in Supramolecular Chemistry Data Analysis. *Chem. Commun.* **2016**, *52* (87), 12792–12805. https://doi.org/10.1039/C6CC03888C.
(26) Gilchrist, A. E.; Liu, Y.; Klett, K.; Liu, Y.-C.; Ceva, S.; Heilshorn, S. C. Transient Competitors to Modulate Dynamic Covalent Cross-Linking of Recombinant Hydrogels. *Chem. Mater.* **2023**, *35* (21), 8969–8983. https://doi.org/10.1021/acs.chemmater.3c01575.
(27) Richbourg, N. R.; Wancura, M.; Gilchrist, A. E.; Toubbeh, S.; Harley, B. A. C.; Cosgriff-Hernandez, E.; Peppas, N. A. Precise Control of Synthetic Hydrogel Network Structure via Linear, Independent Synthesis-Swelling Relationships. *Sci. Adv.* **2021**, *7* (7), eabe3245. https://doi.org/10.1126/sciadv.abe3245.
